# Inflammatory lipid biomarkers and transplant-free mortality risk in hepatitis B-related cirrhosis and hepatic encephalopathy

**DOI:** 10.3389/fmed.2025.1528733

**Published:** 2025-01-23

**Authors:** Ke Shi, Xiaojing Wang, Zhang Yi, Yanqiu Li, Ying Feng, Xianbo Wang

**Affiliations:** Center of Integrative Medicine, Beijing Ditan Hospital, Capital Medical University, Beijing, China

**Keywords:** hepatitis B virus, hepatic encephalopathy, model end-stage liver disease score, inflammatory, high density lipoprotein cholesterol

## Abstract

**Objective:**

Inflammatory reactions and dyslipidemia are associated with the pathogenesis and prognosis of hepatitis B virus-related cirrhosis. We aimed to assess the predictive ability of these parameters in patients with hepatitis B virus-related cirrhosis and overt hepatic encephalopathy (HBV-related OHE).

**Design:**

We conducted an analysis of 1,404 participants diagnosed with HBV-related OHE between January 2008 and July 2023. The prognostic significance of the neutrophil-to-high-density lipoprotein cholesterol (HDL-C) ratio (NHR), lymphocyte-to-HDL-C ratio (LHR), and monocyte-to-HDL-C ratio (MHR) was evaluated using the area under the receiver operating characteristic curve (AUC). Restrictive cubic splines (RCS) were employed to explore the relationship between NHR and 12-month transplant-free (TF) mortality. This study included a prospective test cohort of 328 patients.

**Results:**

NHR was identified as an independent risk factor for 12-month TF mortality. The AUC for NHR (0.776) was similar to that of the model end-stage liver disease (MELD) score (AUC: 0.777). In the test cohort, NHR demonstrated AUC values comparable to MELD, with significantly higher AUCs than LHR and MHR (both *p* < 0.05). Based on cutoff values for NHR and MELD, patients were classified into four risk subgroups: very-low (NHR < 10 and MELD <18), low (NHR ≥ 10 and MELD <18), moderate (NHR < 10 and MELD ≥18), and high (NHR ≥ 10 and MELD ≥18). The 12-month TF mortality rates in the training cohort were 7.2, 23.5, 30.8, and 51.4%, respectively, for these subgroups, while in the test cohort, the rates were 8.7, 20.5, 30.7, and 46.0%.

**Conclusion:**

NHR is a valuable and accessible prognostic indicator for 12-month TF mortality in patients with HBV-related OHE. Patients with both NHR ≥ 10 and MELD ≥18 are at the highest risk of mortality.

## Introduction

Chronic hepatitis B virus (HBV) infection is the leading cause of cirrhosis in China, often progressing to severe liver failure and life-threatening complications (1). Hepatic encephalopathy (HE), a complex neurological manifestation of cirrhosis, is characterized by high recurrence and mortality rates (2). This condition significantly diminishes patients’ quality of life and imposes a substantial burden on healthcare systems (3). Clinical studies have identified overt HE (OHE) as a major driver of hospital admissions and readmissions among cirrhotic patients (4, 5). Given its considerable impact on morbidity and mortality, it is crucial to identify high-risk patients using reliable biomarkers to enable closer monitoring and timely intervention, thereby preventing the progression of HBV-related OHE.

Decompensated cirrhosis is frequently complicated by infections and carries a high risk of mortality due to dysregulated innate immune responses, which result in both inflammation and immune paralysis (6). Research has highlighted the role of systemic inflammation and lipid abnormalities in the pathogenesis and progression of HE (7, 8). Neutrophils, as key mediators of inflammation, release cytotoxic substances that contribute to the body’s defense against pathogens (9). High-density lipoprotein cholesterol (HDL-C), on the other hand, has protective roles in anti-infection, anti-oxidation, and antithrombotic processes (10). HDL-C also functions as an endogenous inhibitor of inflammation, showing inverse correlations with inflammatory markers (11, 12). Reduced HDL-C levels are strongly linked to poor prognoses in various diseases (13, 14). However, relying on inflammatory or lipid markers alone may not provide sufficient prognostic accuracy. Recent studies have focused on the interplay between inflammatory and lipid markers and their combined predictive value in chronic liver diseases. Ratios such as neutrophil-to-HDL-C (NHR) (14, 15), lymphocyte-to-HDL-C (LHR) (16), and monocyte-to-HDL-C (MHR) (17, 18) have been explored as predictors for conditions including stroke, Parkinson’s disease, cardiovascular disease, and metabolic syndrome. Nevertheless, limited evidence exists regarding the prognostic significance of these biomarkers in predicting 12-month transplant-free (TF) mortality among patients with HBV-related OHE.

This study aims to evaluate the predictive capabilities of NHR, LHR, and MHR for 12-month TF mortality and develop a risk stratification strategy for patients with HBV-related OHE.

## Materials and methods

### Participants

From January 2008 to July 2023, a total of 2,534 hospitalized patients with OHE aged 18–75 years were screened at Beijing Ditan Hospital. Chronic hepatitis B was diagnosed by the presence of hepatitis B surface antigen for more than six months (19). OHE diagnosis was confirmed based on the West Haven Criteria (20). Cirrhosis was identified through liver biopsy, endoscopic or radiological evidence of portal hypertension (e.g., ultrasound, computed tomography, or magnetic resonance imaging), and/or complications of cirrhosis, including ascites, HE, or upper gastrointestinal bleeding (21).

Exclusion criteria included co-infections (e.g., HIV, hepatitis A, C, D, or E), malignant tumors, liver transplantation, neuropsychiatric disorders, use of psychotropic drugs, minimal HE, and other liver diseases such as alcoholic liver disease, autoimmune liver disease, metabolic-associated fatty liver disease (MASLD), drug-induced liver injury, cholestatic liver disorders, or inherited conditions. Patients with incomplete clinical data were also excluded. For patients with comorbidities such as hypertension and diabetes, these conditions were not exclusion criteria. However, MASLD was excluded based on clinical and imaging findings (e.g., liver ultrasound or MRI), and histopathological evaluation was performed when necessary to ensure the liver disease was primarily attributable to HBV-related cirrhosis.

**Figure 1:**
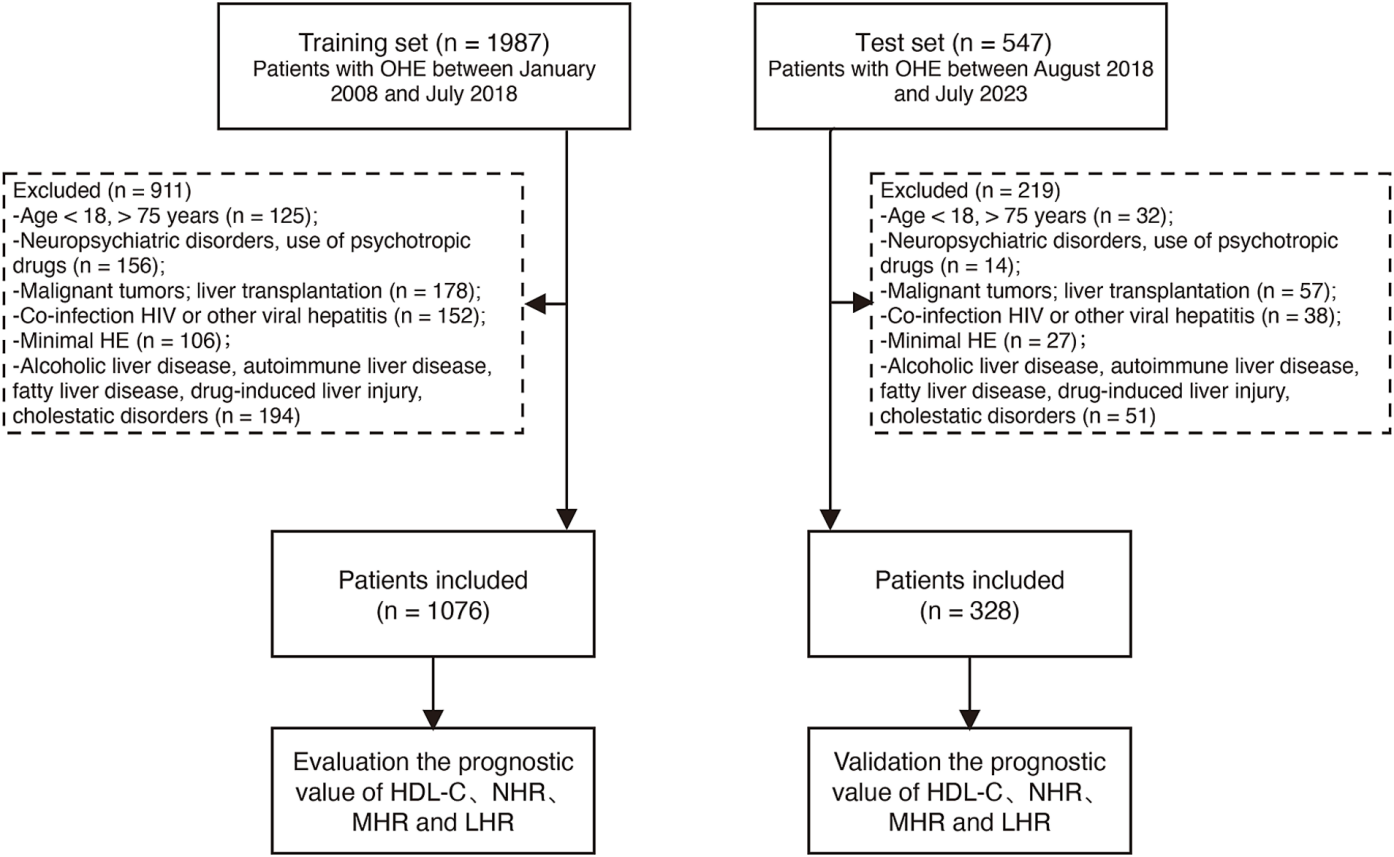
Participant flow in the study. HBV, hepatitis B virus; OHE, overt hepatic encephalopathy; HDL-C, high-density lipoprotein cholesterol; NHR, neutrophil-to-high-density lipoprotein cholesterol ratio; LHR, lymphocyte-to-high-density lipoprotein cholesterol ratio; MHR, monocyte-to-high-density lipoprotein cholesterol ratio.

Ultimately, 1,076 patients met the inclusion criteria and formed the training cohort. An additional 328 patients treated between August 2018 and July 2023 were included as the test cohort (Figure 1). The index date was the time of OHE diagnosis, and outcomes were assessed as TF mortality or survival at the end of the 12-month follow-up period. The study complied with the Declaration of Helsinki Ethical Guidelines and was approved by the Ethics Committee of Beijing Ditan Hospital.

### Treatment of OHE

Management of OHE was tailored according to the clinical classification of HE, the severity of symptoms, and the underlying liver disease status. Standard treatment included a combination of L-ornithine L-aspartate, lactulose, and/or rifaximin. L-ornithine L-aspartate was administered to enhance ammonia metabolism, lactulose was used to reduce ammonia levels by promoting its excretion, and rifaximin provided antimicrobial effects by decreasing ammonia production from gut bacteria. The selection and combination of therapies were guided by the severity of encephalopathy, the presence of comorbidities, and the patient’s response to treatment. This individualized approach aimed to optimize outcomes by addressing the multifactorial nature of OHE.

### Data collection

Baseline characteristics and biochemical data were obtained from the medical record system. These included patient demographics (age, sex), complications, and laboratory parameters such as alanine aminotransferase (ALT), aspartate aminotransferase (AST), total bilirubin (TBIL), albumin (ALB), platelet count, international normalized ratio (INR), creatinine (Cr), neutrophils, lymphocytes, monocytes, total cholesterol (TC), triglycerides (TG), high-density lipoprotein cholesterol (HDL-C), and low-density lipoprotein cholesterol (LDL-C). Ratios such as NHR, LHR, and MHR were calculated by dividing neutrophil, lymphocyte, and monocyte counts by HDL-C levels. The severity of liver disease was assessed using the Model for End-Stage Liver Disease (MELD) score.

### Statistical analysis

Continuous variables were summarized as mea*n* ± standard deviation (SD) or median with interquartile range (IQR), and comparisons between two groups were performed using the T-test or Mann–Whitney U test. Categorical variables were presented as frequencies and percentages, with group differences evaluated using chi-square or Fisher’s exact tests. Multivariate Cox regression analysis included variables with clinical relevance or statistical significance in univariate analysis (*p* < 0.05). A stepwise backward elimination approach was used to finalize the model, retaining only significant predictors. Adjusted hazard ratios (aHR) and 95% confidence intervals (CIs) were calculated, with significance defined as *p* < 0.05 (two-tailed). Statistical analyses were conducted using SPSS (version 25.0; SPSS Inc., Chicago, IL, USA).

The discriminatory power of NHR, LHR, MHR, and MELD scores was evaluated using the area under the receiver operating characteristic curve (AUC). Comparative analyses at 1, 3, and 12 months were performed using the DeLong test (22). A restricted cubic spline (RCS) with four knots (10th, 50th, 70th, and 90th percentiles of NHR) was employed to assess potential nonlinear relationships between NHR and prognosis (23). The importance of predictive indicators was analyzed using random forest models with tenfold cross-validation (24). Optimal cutoff values for NHR and MELD scores were determined using X-tile software. Survival rates were estimated with Kaplan–Meier curves using R software (version 4.2.2; The R Foundation, Vienna, Austria).

## Results

### Baseline characteristics

Among 1,404 patients, 1,132 deaths (80.6%) were attributed to liver-related causes, while 84 deaths (6.0%) were non-liver-related, with the remainder unclassified. Liver-related mortality included upper gastrointestinal bleeding and hemorrhagic shock (416 cases, 29.6%), multiple organ failure (351 cases, 25%), hepatic encephalopathy (116 cases, 8.3%), toxic shock from infection (212 cases, 15.1%), and liver failure (225 cases, 16%). Non-liver-related causes of death included heart failure (56 cases, 4%) and respiratory failure (28 cases, 2%).

**Figure 2:**
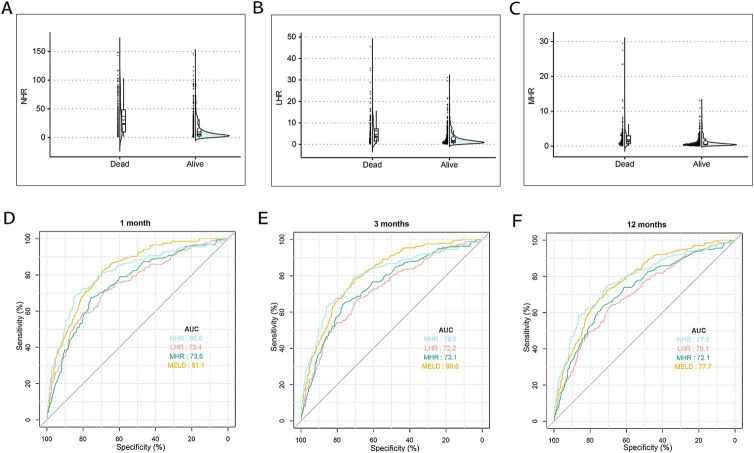
The differences and discrimination ability in NHR, LHR, and MHR between the surviving and deceased patients in the training cohort. **(A)** The difference in NHR between surviving and deceased patients. **(B)** The difference in LHR between surviving and deceased patients. **(C)** The difference in MHR between surviving and deceased patients. **(D–F)** The discrimination ability in NHR, MHR, LHR, and MELD score for TF mortality. ROC curves of NHR, MHR, LHR, and MELD score predicting 1 **(D)**, 3 **(E)**, and 12 months **(F)** TF mortality. MELD, model for end-stage liver disease; HDL-C, high-density lipoprotein cholesterol; NHR, neutrophil to HDL-C ratio; MHR, monocyte to HDL-C ratio; LHR, lymphocyte to HDL-C ratio; TF, transplant-free; ROC, receiver operating characteristic.

Baseline clinical data for the training and test cohorts are shown in Table 1. The median age of patients was 55 years (IQR: 48–64), with 71.2% being male (1,000/1,404), and 25.2% having diabetes (358/1,404). Median NHR, LHR, and MHR were 7.5, 1.7, and 0.7, respectively. The 12-month TF mortality rates were 25.6% (276/1,076) in the training cohort and 24.3% (80/328) in the test cohort. Deceased patients were older, had a higher prevalence of variceal bleeding, infection, and ascites, and demonstrated elevated MELD scores, ALT, AST, TBIL, INR, Cr, NHR, LHR, and MHR levels, with significantly lower TC, ALB, LDL-C, and HDL-C levels (all *p* < 0.001, Table 2). Figures 2A–C illustrate differences in NHR, LHR, and MHR between survivors and deceased patients (all *p* < 0.001).

**Table 1:** Baseline demographics and clinical characteristics of patients with hepatitis B virus-related cirrhosis and overt hepatic encephalopathy in the training and test cohorts.

Variables	All patients (*n* = 1,404)	Training cohort (*n* = 1,076)	Test cohort (*n* = 328)	*p*-value
Age (y)	55.0 (48.0–64.0)	56.0 (48.0–65.0)	54.0 (48.0–62.8)	0.586
Sex (male)	1,000 (71.2)	752 (69.9)	248 (75.6)	0.055
Hypertension (%)	303 (21.6)	231 (21.5)	72 (22.0)	0.852
Diabetes (%)	358 (25.5)	263 (24.4)	95 (29.0)	0.116
Variceal bleeding (%)	485 (34.5)	372 (34.6)	113 (34.5)	0.968
Infection (%)	245 (17.4)	184 (17.1)	61(17.1)	0.532
Ascites (%)	870 (62.0)	670 (62.3)	200 (60.9)	0.673
HE (%)				0.092
II	945 (67.3)	747 (69.4)	198 (60.4)	
III	350 (24.9)	258 (24.0)	92 (28.0)	
IV	109 (7.8)	71 (6.6)	38 (11.6)	
MELD score	16.1 (12.3–23.5)	16.2 (12.4–23.4)	15.9 (12.2–23.8)	0.878
ALT (U/L)	30.7 (19.1–56.7)	30.5 (19.3–57.0)	30.9 (19.1–55.7)	0.858
AST (U/L)	49.5 (31.5–99.8)	48.9 (31.3–100.9)	51.4 (31.8–98.3)	0.700
TBIL (μmol/L)	53.1 (26.4–146.7)	51.7 (26.4–147.9)	58.6(31.8–143.5)	0.610
ALB (g/L)	28.3 ± 5.0	28.2 ± 5.0	28.5 ± 4.9	0.798
PLT (×10^9^/L)	69.0 (46.0–102.0)	69.0 (46.5–102.7)	66.1 (45.1–97.7)	0.495
INR	1.5 (1.3–1.9)	1.5 (1.3–1.9)	1.5 (1.3–1.9)	0.753
Cr (μmol/L)	70.3 (56.6–97.9)	69.2 (56.5–96.7)	72.1 (56.8–103.7)	0.675
Neutrophils (×10^9^/L)	3.8 (2.3–6.3)	3.8 (2.3–6.3)	3.8 (2.2–6.4)	0.378
Lymphocytes (×10^9^/L)	0.9 (0.6–1.3)	0.9 (0.6–1.3)	0.9 (0.6–1.4)	0.393
Monocytes (×10^9^/L)	0.4 (0.3–0.5)	0.4 (0.3–0.5)	0.4 (0.3–0.5)	0.637
TC (mmol/L)	2.4 (1.9–3.1)	2.4 (1.9–3.0)	2.4 (1.9–3.1)	0.395
TG (mmol/L)	0.6 (0.4–0.8)	0.6 (0.4–0.8)	0.6 (0.4–0.9)	0.418
HDL-C (mmol/L)	0.5 (0.3–0.9)	0.5 (0.3–0.8)	0.5 (0.3–0.9)	0.433
LDL-C (mmol/L)	1.1 (0.8–1.6)	1.1 (0.8–1.6)	1.1 (0.8–1.7)	0.316
NHR	7.5 (3.2–22.0)	7.4 (3.2–21.7)	7.6 (3.4–23.6)	0.630
MHR	0.7 (0.4–1.5)	0.7 (0.4–1.5)	0.8 (0.4–1.5)	0.719
LHR	1.7 (0.9–4.0)	1.7 (0.9–4.0)	1.8 (0.9–4.0)	0.677

Data are presented as mean ± SD or *n* (%) or median (interquartile range). HE, hepatic encephalopathy; MELD, model for end-stage liver disease; ALT, alanine aminotransferase; AST, aspartate aminotransferase; TBIL, total bilirubin; ALB, albumin; PLT, platelet count; INR, international normalized ratio; Cr, creatinine; TC, total cholesterol; TG, triglyceride; HDL-C, high-density lipoprotein cholesterol; LDL-C, low-density lipoprotein cholesterol; NHR, neutrophil to HDL-C ratio; MHR, monocyte to HDL-C ratio; LHR, lymphocyte to HDL-C ratio.

**Table 2:** Baseline characteristics of survival and death patients in the training cohort.

Variables	Survived (*n* = 800)	Death (*n* = 276)	*P*-value
Age (y)	53.0 (46.0–61.0)	55.0 (47.0–64.0)	0.003
Sex (male)	575 (71.9)	177 (64.1)	0.002
Hypertension (%)	153 (17.2)	78 (28.3)	< 0.001
Diabetes (%)	195 (24.9)	68 (24.6)	0.378
Variceal bleeding (%)	251 (32.5)	128 (46.4)	< 0.001
Infection (%)	100 (12.5)	84 (30.4)	< 0.001
Ascites (%)	460 (57.4)	210 (70.6)	< 0.001
HE (%)			0.015
II	564 (66.5)	183 (66.3)	
III	188 (26.0)	70 (25.4)	
IV	48 (7.5)	23 (8.3)	
MELD score	15.0 (11.6–19.9)	24.8 (18.1–29.5)	< 0.001
ALT (U/L)	29.2 (19.0–47.8)	53.8 (25.7–148.7)	< 0.001
AST (U/L)	45.9 (30.8–84.4)	102.7(47.0–243.3)	< 0.001
TBIL (μmol/L)	45.8 (24.9–99.8)	174.3 (59.3–397.3)	< 0.001
ALB (g/L)	28.7 ± 5.0	26.9 ± 5.6	< 0.001
PLT (×10^9^/L)	69.0 (46.5–103.0)	64.0 (45.3–101.3)	0.560
INR	1.5 (1.3–1.8)	1.9 (1.6–2.5)	< 0.001
Cr (μmol/L)	67.9 (55.8–88.1)	91.5(61.6–150.6)	< 0.001
Neutrophils (×10^9^/L)	3.5 (2.2–5.8)	5.6 (3.6–9.1)	< 0.001
Lymphocytes (×10^9^/L)	0.9 (0.6–1.3)	1.0 (0.6–1.3)	0.939
Monocytes (×10^9^/L)	0.4 (0.3–0.5)	0.4 (0.3–0.5)	0.141
TC (mmol/L)	2.4 (2.0–3.2)	2.0 (1.4–2.4)	< 0.001
TG (mmol/L)	0.6 (0.4–0.8)	0.6 (0.4–0.8)	0.598
HDL-C (mmol/L)	0.6 (0.4–0.9)	0.2 (0.1–0.5)	< 0.001
LDL-C (mmol/L)	1.2 (0.9–1.6)	0.9 (0.6–1.1)	< 0.001
NHR	6.4 (2.7–15.9)	24.2 (11.1–49.7)	< 0.001
MHR	0.6 (0.4–1.2)	1.4 (0.7–3.1)	< 0.001
LHR	1.5 (0.8–3.2)	3.8 (1.6–7.2)	< 0.001

Data are presented as mean ± SD or n (%) or median (interquartile range). HE, hepatic encephalopathy; MELD, model for end-stage liver disease; ALT, alanine aminotransferase; AST, aspartate aminotransferase; TBIL, total bilirubin; ALB, albumin; PLT, platelet count; INR, international normalized ratio; Cr, creatinine; TC, total cholesterol; TG, triglyceride; HDL-C, high-density lipoprotein cholesterol; LDL-C, low-density lipoprotein cholesterol; NHR, neutrophil to HDL-C ratio; MHR, monocyte to HDL-C ratio; LHR, lymphocyte to HDL-C ratio.

### Predictors of 12 months TF mortality

Univariate analysis identified significant associations between 12-month TF mortality and variables such as age, sex, variceal bleeding, infection, ascites, MELD score, NHR, LHR, MHR, ALT, AST, ALB, and TC (all *p* < 0.05). Multivariate Cox regression analysis revealed that age (HR = 1.015, 95% CI: 1.005–1.026; *p* = 0.009), MELD score (HR = 1.092, 95% CI: 1.077–1.107; *p* < 0.001), NHR (HR = 1.006, 95% CI: 1.002–1.011; *p* = 0.003), LHR (HR = 1.013, 95% CI: 1.001–1.025; *p* = 0.033), and ALB (HR = 0.970, 95% CI: 0.945–0.996; *p* = 0.024) were independent predictors of 12-month TF mortality (Table 3).

**Table 3:** Univariate and multivariate Cox hazards analysis for 12 months transplant-free mortality among patients with hepatitis B virus-related cirrhosis and overt hepatic encephalopathy.

Variables	Univariate analysis HR (95%CI)	*P*-value	Multivariate analysis HR (95%CI)	*P*-value
Age (y)	1.015 (1.005–1.026)	0.003	1.015 (1.005–1.026)	0.009
Sex (male)	0.590 (0.461–0.755)	< 0.001		
Hypertension (%)	1.551 (0.993–2.016)	0.821		
Diabetes (%)	0.991 (0.752–1.303)	0.946		
Variceal bleeding (%)	1.446 (1.140–1.834)	0.002		
Infection (%)	2.375 (1.837–3.071)	0.001		
Ascites (%)	1.397 (1.016–1.763)	< 0.001		
HE (%)				
II	Reference			
III	1.058 (0.823–1.428)	0.564		
IV	1.393 (0.902–2.149)	0.135		
MELD score	1.010 (1.088–1.114)	< 0.001	1.092 (1.077–1.107)	< 0.001
NHR	1.015 (1.013–1.017)	< 0.001	1.006 (1.002–1.011)	0.003
MHR	1.033 (1.022–1.044)	< 0.001		
LHR	1.025 (1.019–1.031)	< 0.001	1.013 (1.001–1.025)	0.033
ALT (U/L)	1.001 (1.000–1.001)	< 0.001		
AST (U/L)	1.001 (1.001–1.001)	< 0.001		
ALB (g/L)	0.945 (0.922–0.968)	< 0.001	0.970 (0.945–0.996)	0.024
PLT (×10^9^/L)	1.000 (0.998–1.002)	0.918		
TC (mmol/L)	0.737 (0.663–0.823)	0.001		
TG (mmol/L)	1.021 (0.812–1.282)	0.861		

*HE, hepatic encephalopathy; MELD, Model for End-Stage Liver Disease; ALT, alanine aminotransferase; AST, aspartate aminotransferase; ALB, albumin; PLT, platelet count; TC, total cholesterol; TG, triglyceride; NHR, neutrophil to HDL-C ratio; MHR, monocyte to HDL-C ratio; LHR, lymphocyte to HDL-C ratio; HR, Hazard ratio; CI, confidence interval.

### Performance of inflammatory and lipid parameters

**Figure 3:**
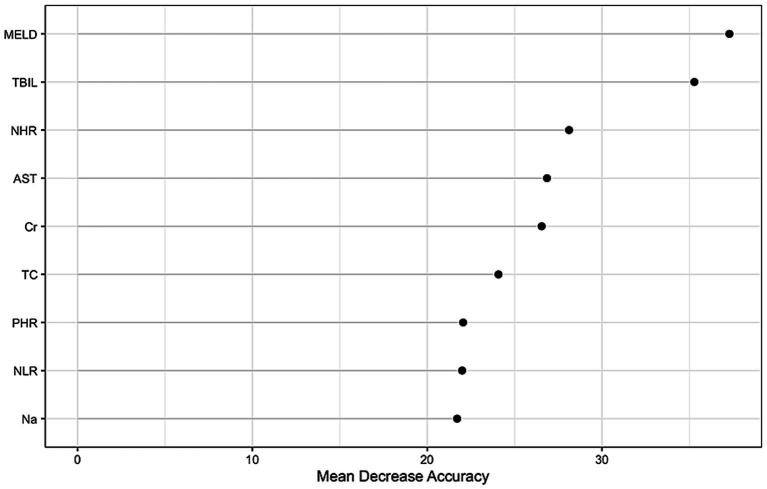
Random forest analysis.

The predictive performance of HDL-C levels, NHR, LHR, MHR, and MELD scores was evaluated using receiver operating characteristic (ROC) curves (Figures 2D–F). At 1, 3, and 12 months in the training set, NHR demonstrated predictive accuracy comparable to the MELD score, with AUC values of 0.800, 0.795, and 0.776 for NHR and 0.811, 0.806, and 0.777 for MELD, respectively. NHR outperformed LHR (AUC: 0.734, 0.722, and 0.701) and MHR (AUC: 0.736, 0.731, and 0.721) across the same time intervals (all *p* < 0.05). Random forest analysis further identified NHR as a key predictive factor, ranking second only to MELD and TBIL levels (Figure 3). These findings underscore NHR’s significance as a reliable prognostic marker.

### Association of NHR and MELD scores with survival

**Figure 4:**
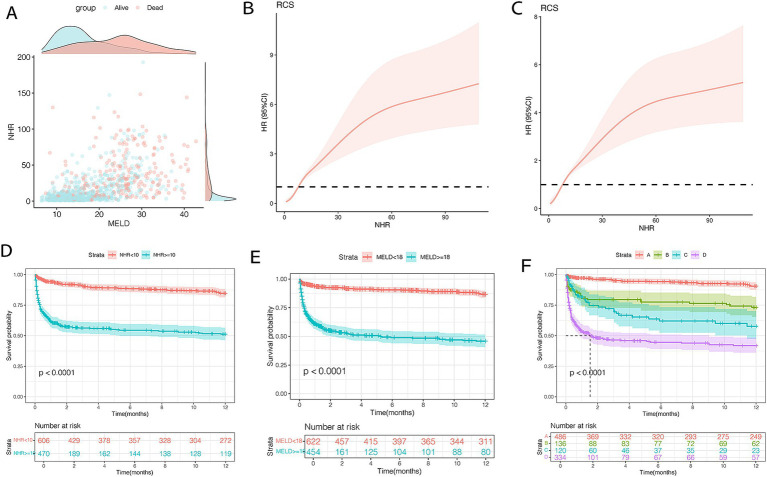
Association between NHR levels and mortality **(A–C)** and survival curves **(D–F)** of patients with HBV-related cirrhosis and OHE in the training cohort (*n* = 1,076). **(A)** The distribution of survival and death patients. **(B)**The association between NHR and 12-month TF mortality (unadjusted). **(C)** The association between NHR and 12-month TF mortality (adjusted). **(D)** Survival probability in patients with NHR < 10 (*n* = 609) and ≥ 10 (*n* = 467). **(E)** Survival probability in patients with MELD < 18 (*n* = 622) and ≥ 18 (*n* = 454). **(F)** Survival probability in the very low- (NHR < 10 and MELD <18, *n* = 486), low- (NHR ≥ 10 and MELD <18, *n* = 136), moderate- (NHR < 10 and MELD ≥18, *n* = 120), and high-risk (NHR ≥ 10 and MELD ≥18, *n* = 334) groups. HBV, hepatitis B virus; NHR, neutrophil to high-density lipoprotein cholesterol ratio; OHE, overt hepatic encephalopathy; MELD, model for end-stage liver disease; TF, transplant-free.

Using X-tile software, the optimal cutoff values for 12-month TF mortality were determined to be 10 for NHR and 18 for MELD scores. Scatter plots illustrated that patients with NHR ≥ 10 and MELD ≥18 had the poorest prognosis (Figure 4A). Patients with MELD scores ≥18 exhibited higher rates of infection and ascites, as well as elevated ALT, AST, TBIL, INR, Cr, TC, NHR, and LHR levels compared to those with MELD <18 (all *p* < 0.001; Table 4). RCS analysis revealed a nonlinear relationship between NHR and 12-month TF mortality risk (unadjusted and adjusted, both *p* < 0.001; Figures 4B,C). Mortality risk was relatively low when NHR was <10 but increased significantly when NHR exceeded 10.

**Table 4:** Clinical characteristics according to MELD scores in the training cohort.

Variables	MELD < 18 (*n* = 622)	MELD ≥ 18 (*n* = 454)	*P*-value
Age (y)	58.0 (49.0–66.0)	53.0 (46.0–61.0)	0.001
Sex (male)	419 (67.3)	333 (73.3)	0.035
Hypertension (%)	135 (21.7)	96 (21.1)	0.826
Diabetes (%)	168 (27.0)	95 (20.9)	0.022
Variceal bleeding (%)	237 (38.1)	135 (29.7)	< 0.001
Infection (%)	70 (11.2)	114 (25.1)	< 0.001
Ascites (%)	334 (53.6)	336 (74.0)	< 0.001
HE (%)			0.124
II	417 (67.0)	330 (72.6)	
III	159 (25.6)	99 (21.8)	
IV	46 (7.4)	25 (5.5)	
ALT (U/L)	24.5 (17.6–38.2)	46.4 (25.7–138.4)	< 0.001
AST (U/L)	38.9 (27.4–59.4)	86.0 (47.4–197.2)	< 0.001
TBIL (μmol/L)	31.7 (18.9–50.4)	184.5 (93.5–355.9)	< 0.001
ALB (g/L)	28.9 ± 4.9	27.5 ± 5.3	< 0.001
PLT (×10^9^/L)	71.0 (55.8–107.0)	61.0 (42.2–96.0)	0.017
INR	1.5 (1.3–1.8)	2.0 (1.7–2.4)	< 0.001
Cr (μmol/L)	66.2 (53.2–83.0)	81.1(60.9–140.0)	< 0.001
Neutrophils (×10^9^/L)	3.0 (2.0–5.2)	4.0 (2.7–7.0)	< 0.001
Lymphocytes (×10^9^/L)	0.9 (0.6–1.3)	0.9 (0.6–1.3)	0.939
Monocytes (×10^9^/L)	0.4 (0.3–0.5)	0.4 (0.3–0.5)	0.087
TC (mmol/L)	2.6 (2.3–3.3)	2.1 (1.5–2.5)	< 0.001
TG (mmol/L)	0.6 (0.4–0.8)	0.5 (0.4–0.7)	0.642
HDL-C (mmol/L)	0.7 (0.5–1.0)	0.2 (0.1–0.5)	< 0.001
LDL-C (mmol/L)	1.5 (1.0–1.8)	1.0 (0.6–1.2)	< 0.001
NHR	3.9 (2.1–8.8)	23.2 (9.0–41.8)	< 0.001
MHR	0.5 (0.3–0.8)	1.7 (0.8–3.0)	< 0.001
LHR	1.2 (0.7–2.1)	3.9 (1.6–7.8)	< 0.001

Data are presented as mea*n* ± SD or *n* (%) or median (interquartile range). HE, hepatic encephalopathy; MELD, model for end-stage liver disease; ALT, alanine aminotransferase; AST, aspartate aminotransferase; TBIL, total bilirubin; ALB, albumin; PLT, platelet count; INR, international normalized ratio; Cr, creatinine; TC, total cholesterol; TG, triglyceride; HDL-C, high-density lipoprotein cholesterol; LDL-C, low-density lipoprotein cholesterol; NHR, neutrophil to HDL-C ratio; MHR, monocyte to HDL-C ratio; LHR, lymphocyte to HDL-C ratio.

Kaplan–Meier analysis showed that patients with NHR < 10 had a 12-month mortality rate of 12.1%, compared to 43.2% for those with NHR ≥ 10 (*p* < 0.0001; Figure 4D). Similarly, mortality was 10.7% in patients with MELD scores <18 and 46.0% in those with MELD ≥18 (*p* < 0.0001; Figure 4E). Based on these thresholds, patients were classified into four risk groups: very low (NHR < 10, MELD <18), low (NHR ≥ 10, MELD <18), moderate (NHR < 10, MELD ≥18), and high (NHR ≥ 10, MELD ≥18). Mortality rates for the four groups were 7.2, 23.5, 30.8, and 51.4%, respectively (*p* < 0.0001; Figure 4F).

### Risk stratification in the subgroup analysis

**Figure 5:**
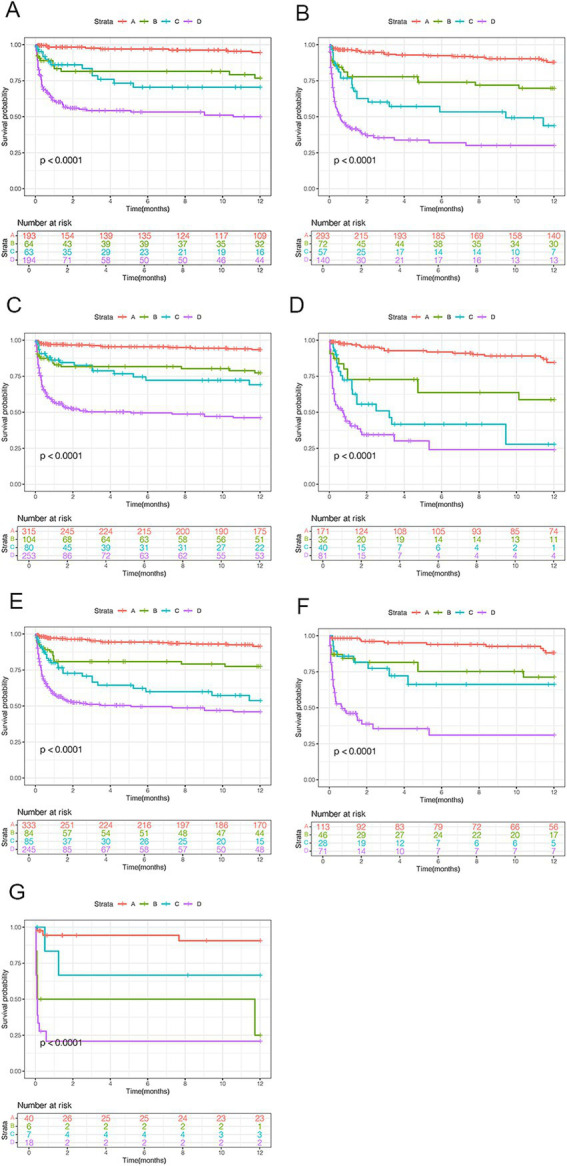
Survival analyses by clinical characteristics for patients with HBV-related cirrhosis and OHE. **(A)** Age < 55 years. **(B)** Age ≥ 55 years. **(C)** Male patients. **(D)** Female patients. **(E–G)** OHE grades II, III, and IV. HBV, hepatitis B virus; OHE, overt hepatic encephalopathy.

To assess mortality risk in subgroups, patients were stratified by age (< 55 or ≥ 55 years), sex, and OHE grades (II, III, or IV). Across all subgroups, patients with NHR ≥ 10 and MELD ≥18 (high risk) consistently had significantly higher 12-month mortality compared to those in the very low-risk group (NHR < 10, MELD <18), regardless of age (both *p* < 0.0001; Figures 5A,B). Among male patients, the 12-month mortality rates in the very low-, low-, moderate-, and high-risk groups were 5.3, 20.1, 22.5, and 47.8%, respectively (*p* < 0.0001; Figure 5C). Similar trends were observed among female patients (*p* < 0.0001; Figure 5D) and in patients with different OHE grades (*p* < 0.0001; Figures 5E,F).

### Validation the MELD scores and NHR in the test set

**Figure 6:**
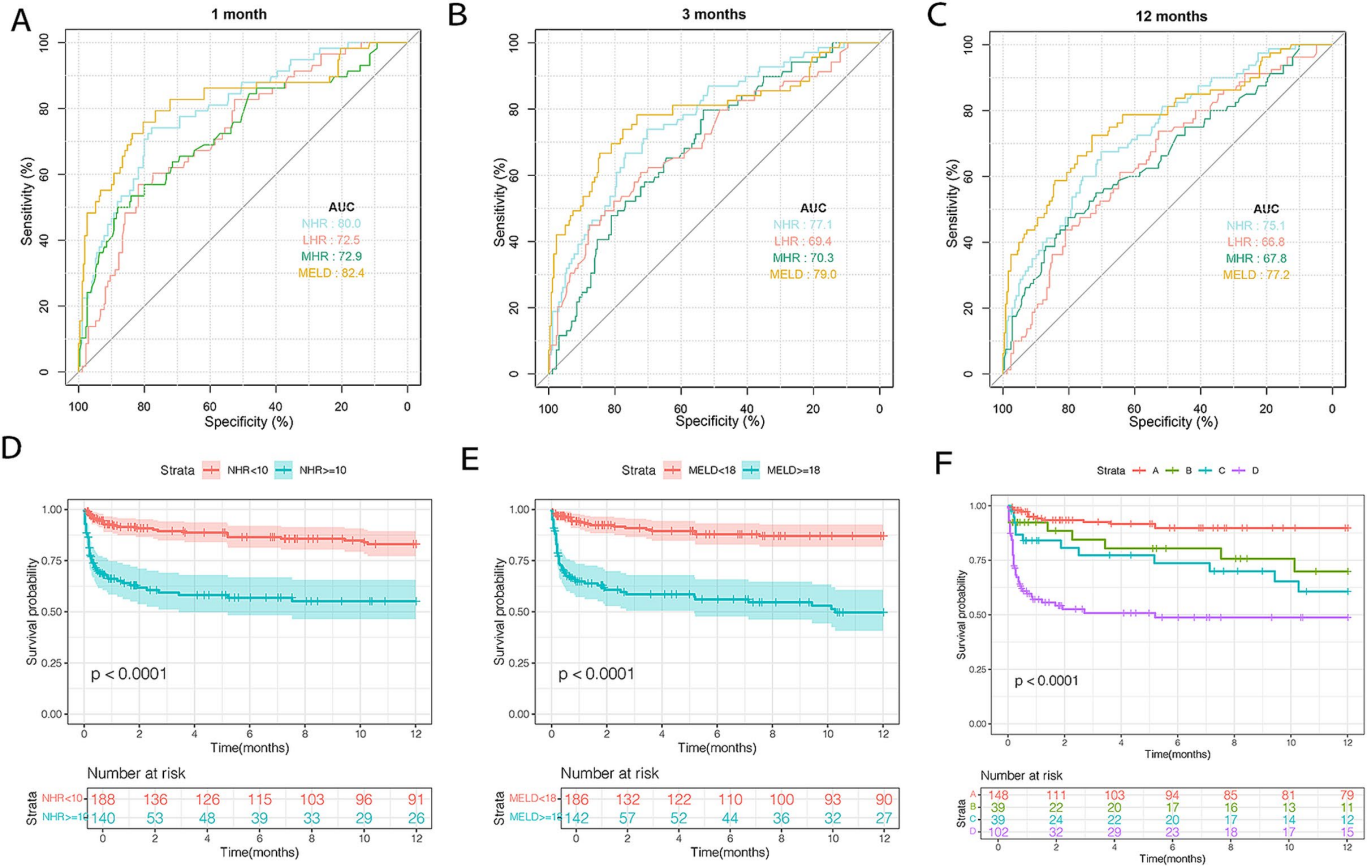
The discrimination ability of these indicators and risk stratification in the test cohort. ROC curves of NHR, MHR, LHR, and MELD score predicting 1 **(A)**, 3 **(B)**, and 12 months **(C)** TF mortality. **(D)** Survival probability in patients with NHR < 10 (*n* = 191) and ≥ 10 (*n* = 137). **(E)** Survival probability in patients with MELD < 18 (*n* = 186) and ≥ 18 (*n* = 142). (F) Survival probability in the very low- (NHR < 10 and MELD <18, *n* = 148), low- (NHR ≥ 10 and MELD <18, *n* = 39), moderate- (NHR < 10 and MELD ≥18, *n* = 39), and high-risk (NHR ≥ 10 and MELD ≥18, *n* = 102) groups. HDL-C, high-density lipoprotein cholesterol; NHR, neutrophil to HDL-C ratio; MHR, monocyte to HDL-C ratio; LHR, lymphocyte to HDL-C ratio; TF, transplant-free; MELD, model for end-stage liver disease.

The AUCs of the MELD score and NHR were calculated at 1, 3, and 12 months (Figures 6A–C). MELD scores achieved the highest predictive accuracy at all time points (AUC: 0.824, 0.790, and 0.772), followed by NHR (AUC: 0.800, 0.771, and 0.751). Both metrics significantly outperformed LHR (AUC: 0.725, 0.694, and 0.668) and MHR (AUC: 0.729, 0.703, and 0.678) (all *p* < 0.05). Patients with NHR < 10 had a 12-month TF mortality rate of 15.1%, compared to 37.2% for those with NHR ≥ 10 (*p* < 0.0001; Figure 6D). Similarly, patients with MELD scores <18 exhibited lower mortality (10.7%) than those with MELD ≥18 (42.2%; *p* < 0.0001; Figure 6E). Among the 328 patients in the test cohort, the distribution across risk groups was as follows: 45.1% in very low risk, 11.9% in low risk, 11.9% in moderate risk, and 31.1% in high risk. Corresponding 12-month mortality rates were 8.7, 20.5, 30.7, and 46.0%, respectively (*p* < 0.001; Figure 6F).

## Discussion

OHE, a severe complication of cirrhosis, is associated with significant liver dysfunction and poor prognosis (25). Identifying reliable predictive biomarkers is crucial for informing patients and guiding timely interventions. In this study, we demonstrated that NHR is a practical, inexpensive, and effective lipid-related inflammatory biomarker. Compared to LHR and MHR, NHR exhibited superior predictive value for 12-month TF mortality in patients with HBV-related OHE, highlighting its potential role in clinical risk stratification.

The liver plays a central role in lipid metabolism, and liver dysfunction often leads to impaired lipid synthesis and clearance. In advanced liver diseases such as cirrhosis or liver failure, the liver’s ability to regulate lipids, particularly HDL cholesterol, is significantly reduced. Consequently, lower circulating HDL-C levels are commonly observed and are strongly associated with disease severity (11, 26). Disruption of lipid metabolism can also activate inflammatory responses (27). Clinical and lipidomic studies have linked HDL-C-related indicators and lipid mediators to inflammatory markers (11, 28). Inflammation and dyslipidemia play crucial roles in promoting liver dysfunction and the progression of cirrhosis (8). Ratios such as NHR, MHR, and LHR reflect the interplay between inflammatory responses and lipid changes (29). In this study, we found that NHR was independently associated with 12-month transplant-free (TF) mortality. NHR, which can be easily calculated from routine blood tests, demonstrated superior predictive accuracy compared to LHR and MHR at 1-, 3-, and 12-month intervals (all *p* < 0.05). Neutrophils, as the first immune cells to infiltrate the liver, play a key role in clearing dead and infected cells while producing cytokines that modulate monocytes and lymphocytes (30). This may explain why NHR outperforms MHR and LHR as a prognostic marker. These findings were validated in an independent dataset, highlighting the potential of NHR as a valuable tool for predicting mortality in patients with HBV-related OHE.

HBV-related OHE is associated with high hospitalization and mortality rates. Identifying high-risk individuals is critical for initiating timely treatment and reducing OHE-related mortality. The MELD score is a well-established prognostic tool that reflects liver function and predicts short-term mortality in advanced liver disease (31). Similarly, NHR, derived from routine blood tests, is closely linked to inflammation and poor prognosis in cirrhotic patients. Our findings showed that NHR demonstrated predictive ability comparable to the MELD score. This combination of heightened inflammation and impaired liver function suggests a unique pathophysiological mechanism requiring attention in pretransplant mortality risk assessment. Notably, patients with elevated MELD scores (≥18) and NHR (≥10) had the worst prognoses, as shown by scatter plot distributions. In the training cohort, four risk subgroups were identified based on NHR and MELD thresholds: very low-risk (NHR < 10, MELD <18), low-risk (NHR ≥ 10, MELD <18), moderate-risk (NHR < 10, MELD ≥18), and high-risk (NHR ≥ 10, MELD ≥18). Patients in the very low-risk group, accounting for approximately 45% of the cohort, had a mortality rate of 7.2%, indicating relatively stable liver function. Clinical management for this group may focus on regular follow-up and lifestyle modifications. In the low-risk group, elevated NHR indicated active inflammation and potential liver deterioration, even with lower MELD scores, suggesting the need for early anti-inflammatory interventions. The moderate-risk group exhibited significant liver impairment but lower inflammation levels, potentially reflecting immune suppression or inflammatory exhaustion in late-stage compensated cirrhosis. Close monitoring of liver function and complications is essential for these patients. Finally, the high-risk group, characterized by severe inflammation, lipid dysregulation, and liver dysfunction, requires comprehensive management targeting inflammation, lipid metabolism, and liver support. This stratification provides a precise prognostic assessment and informs individualized treatment strategies.

This study sheds light on the complex interactions among inflammation, lipid metabolism, and TF mortality in patients with HBV-related OHE. Bacterial infections and inflammation exacerbate immune dysfunction, accelerating liver deterioration and increasing mortality risk (32, 33). Conversely, HDL-C suppresses monocyte activation and transformation, thereby attenuating inflammatory responses. Inflammation alters HDL-C composition and functionality, while dysfunctional HDL-C further exacerbates cirrhosis progression and complications (26). NHR reflects the dynamic interplay between neutrophil activity and HDL-C levels. The liver regulates lipoprotein synthesis and degradation, while HDL inhibits neutrophil activation, proliferation, and migration (34). Abnormal neutrophil activation disrupts HDL-C composition and functionality, leading to increased neutrophil production and heightened mortality risk (35).

Despite its novel findings, this study has several limitations. First, the prediction of 1-year mortality based solely on baseline data may be biased by emerging clinical events or additional decompensation during follow-up. While our analysis focused on baseline characteristics, the dynamic nature of the disease must be acknowledged. Second, although this single-center study demonstrated and validated NHR as a prognostic marker, multicenter studies are needed to confirm its predictive value. Third, due to the retrospective design, patients received various treatments and combination therapies, and subgroup analyses based on treatment modalities were not performed. Future prospective studies should explore the impact of these indicators in different treatment settings to provide more comprehensive prognostic guidance. Finally, although confounding variables were adjusted to the greatest extent possible, some unmeasured factors may have influenced the results.

## Conclusion

This cohort study supports NHR as a robust predictor of 12-month TF mortality in patients with HBV-related OHE. NHR ≥ 10 and MELD ≥18 effectively identify high-risk patients and provide valuable insights for optimizing clinical management. Further research is warranted to validate the prognostic utility of NHR in larger, multicenter studies.

## Data Availability

The original contributions presented in the study are included in the article/Supplementary material, further inquiries can be directed to the corresponding authors.
