# Supplementary material for: Inflammatory lipid biomarkers and transplant-free mortality risk in hepatitis B-related cirrhosis and hepatic encephalopathy
Source: Front Med (Lausanne). 2025 Jan 23;12:1528733. doi: 10.3389/fmed.2025.1528733 (PMC11799548; doi:10.3389/fmed.2025.1528733)
Supplement: Supplementary file 1 [file Data_Sheet_1.PDF]

序号	组	病案号	性别	年龄	入院日期	最后一次入院	**时间(d)**	**0时间(d)**
1	0	110808	1	58	2010/1/4	2010/1/5	1	1
2	0	115639	1	56	2010/10/9	2010/10/10	1	1
3	1	121140	1	54	2011/3/2	2011/3/3	1	1
4	0	108966	0	71	2011/8/2	2011/8/3	1	1
5	0	127875	0	62	2011/10/4	2011/10/5	1	1
6	0	133766	1	60	2012/4/14	2012/4/15	1	1
7	0	135224	1	50	2012/5/25	2012/5/26	1	1
8	0	143822	0	58	2013/2/1	2013/2/2	1	1
9	1	144084	1	64	2013/3/16	2013/3/17	1	1
10	0	154372	1	46	2013/11/22	2013/11/23	1	1
11	1	164091	1	54	2014/7/29	2014/7/30	1	1
12	1	179990	1	50	2015/10/7	2015/10/8	1	1
13	0	181647	1	52	2015/11/24	2015/11/25	1	1
14	0	187542	1	40	2016/4/29	2016/4/30	1	1
15	0	178636	1	51	2016/8/19	2016/8/20	1	1
16	0	198991	1	55	2017/2/10	2017/2/11	1	1
17	0	215802	1	58	2018/4/2	2018/4/3	1	1
18	1	207225	1	61	2017/8/28	2017/8/29	1	1
19	0	107744	0	77	2009/10/10	2009/10/12	2	2
20	0	109684	1	40	2009/11/6	2009/11/8	2	2
21	0	112169	0	66	2010/10/23	2010/10/25	2	2
22	0	122144	0	75	2011/4/7	2011/4/9	2	2
23	0	135402	1	57	2012/5/30	2012/6/1	2	2
24	0	127563	1	73	2012/12/3	2012/12/5	2	2
25	0	139189	1	49	2013/2/3	2013/2/5	2	2
26	0	126145	0	66	2013/4/18	2013/4/20	2	2
27	0	147754	0	66	2013/5/27	2013/5/29	2	2
28	0	148176	0	64	2013/6/6	2013/6/8	2	2
29	1	182357	1	44	2016/6/3	2016/6/5	2	2
30	0	190193	1	59	2016/8/23	2016/8/25	2	2
31	0	196825	1	57	2016/12/15	2016/12/17	2	2
32	0	212405	1	66	2018/1/9	2018/1/11	2	2
33	0	101952	1	51	2008/11/11	2008/11/16	5	5
34	0	106418	1	38	2009/6/20	2009/7/2	30	30
35	0	107148	1	57	2009/7/16	2009/7/22	30	30
36	0	**90879**	0	54	2008/11/18	2008/12/4	30	30
37	0	115036	1	26	2010/6/28	2010/7/23	30	30

38	0	**89288**	0	55	2009/1/30	2009/2/14	22	22
39	1	103163	1	70	2009/2/6	2009/2/19	30	30
40	0	119723	1	32	2010/12/29	2011/1/7	16	16
41	0	103056	1	61	2009/3/9	2009/3/16	30	30
42	0	103115	1	72	2009/3/10	2009/3/12	9	9
43	0	104239	1	59	2009/4/8	2009/4/11	30	30
44	0	124272	1	37	2011/6/11	2011/6/13	9	9
45	0	**67082**	1	47	2009/6/6	2009/6/12	6	6
46	1	128965	0	51	2011/11/9	2011/12/1	30	30
47	0	133784	1	54	2012/4/15	2012/4/18	10	10
48	0	108843	1	55	2009/9/27	2009/10/16	26	26
49	0	108580	0	60	2009/10/28	2009/11/20	30	30
50	0	**94341**	1	52	2009/11/8	2010/4/27	170	30
51	0	**83925**	1	47	2009/12/15	2009/12/16	8	8
52	0	110692	0	55	2009/12/28	2010/1/2	12	12
53	0	111331	1	45	2010/1/31	2010/2/25	30	30
54	0	111832	0	64	2010/2/27	2010/3/1	9	9
55	1	105510	1	39	2010/3/3	2010/3/15	19	19
56	0	112170	1	41	2010/3/16	2010/3/22	30	30
57	1	112310	1	61	2010/3/22	2010/3/24	9	9
58	0	112599	1	60	2010/4/4	2010/4/7	10	10
59	0	112691	1	63	2010/4/8	2010/4/28	30	30
60	1	112988	1	58	2010/4/19	2010/4/20	8	8
61	0	114262	0	73	2010/6/3	2010/6/8	30	30
62	0	114723	1	43	2010/6/18	2010/6/19	8	8
63	0	114801	1	47	2010/6/21	2010/6/25	30	30
64	1	116210	1	36	2010/8/4	2010/8/4	7	7
65	1	116259	1	57	2010/8/6	2010/8/12	13	13
66	0	116880	1	32	2010/8/31	2010/9/10	17	17
67	0	**124700**	1	52	2010/10/22	2010/10/25	3	3
68	0	118611	1	28	2010/11/16	2010/12/6	30	30
69	0	119073	1	57	2010/12/2	2010/12/7	30	30
70	0	208608	1	47	2017/10/5	2017/10/15	17	17
71	0	119620	0	70	2010/12/24	2011/1/17	30	30
72	0	120830	1	47	2011/2/17	2011/3/10	30	30
73	1	121632	1	64	2011/3/21	2011/4/8	30	30
74	1	121974	1	65	2011/3/31	2011/4/20	30	30
75	0	122742	1	63	2011/4/26	2011/5/12	23	23

76	0	123097	1	59	2011/5/7	2012/7/12	432	30
77	0	**292548**	0	76	2011/5/21	2011/5/30	30	30
78	1	123983	0	58	2011/6/2	2011/6/8	13	13
79	0	124340	1	47	2011/6/13	2011/6/23	30	30
80	0	124445	1	54	2011/6/16	2011/6/21	30	30
81	0	218538	1	47	2011/6/28	2011/7/1	10	10
82	0	125172	1	23	2011/7/9	2011/7/12	30	30
83	1	125197	1	58	2011/7/10	2019/1/25	2756	30
84	0	125534	1	34	2011/7/20	2011/7/26	13	13
85	0	125701	0	78	2011/7/25	2011/8/10	30	30
86	1	**112384**	1	64	2011/7/27	2011/8/1	30	30
87	0	125911	1	44	2011/8/1	2011/8/4	30	30
88	1	126123	1	52	2011/8/8	2011/8/10	9	9
89	0	122995	1	39	2011/8/17	2019/8/5	2910	30
90	0	126837	1	57	2011/8/30	2011/9/2	10	10
91	0	127273	0	59	2011/9/14	2011/9/22	30	30
92	1	127476	1	41	2011/9/20	2011/10/4	30	30
93	0	127614	1	53	2011/9/25	2011/9/29	11	11
94	0	129274	1	47	2011/11/19	2011/12/5	23	23
95	0	130437	1	43	2011/12/27	2011/12/30	30	30
96	0	129221	1	56	2012/1/8	2012/1/11	30	30
97	0	131062	1	34	2012/1/19	2012/1/23	11	11
98	0	131128	0	63	2012/1/24	2012/1/28	11	11
99	1	119972	1	56	2012/1/25	2012/1/27	30	30
100	0	131142	1	71	2012/1/25	2012/2/2	30	30
101	0	131462	1	39	2012/2/5	2012/2/8	30	30
102	0	123986	1	64	2012/2/11	2012/2/13	9	9
103	0	126802	1	43	2012/3/11	2012/4/5	30	30
104	0	133528	0	61	2012/4/7	2012/4/12	12	12
105	0	133859	1	55	2012/4/17	2012/4/22	30	30
106	0	134170	1	68	2012/4/26	2012/5/3	30	30
107	0	116823	1	37	2012/4/28	2012/5/7	30	30
108	0	135349	1	48	2012/5/29	2012/6/8	30	30
109	1	136327	1	71	2012/6/24	2012/6/29	12	12
110	0	136431	1	74	2012/6/26	2012/7/13	30	30
111	1	136713	1	48	2012/7/4	2012/7/9	12	12
112	0	137008	0	49	2012/7/11	2012/7/13	9	9
113	0	137195	0	65	2012/7/17	2012/8/8	29	29

114	0	137508	0	44	2012/7/25	2012/7/31	30	30
115	1	134959	1	66	2012/8/18	2012/8/27	30	30
116	1	139443	1	58	2012/9/19	2012/9/25	30	30
117	0	113421	1	42	2012/10/11	2012/10/17	30	30
118	0	140364	1	52	2012/10/20	2012/10/29	16	16
119	0	140755	0	82	2012/10/31	2012/11/15	30	30
120	0	140825	1	74	2012/11/2	2012/11/11	16	16
121	0	140912	1	41	2012/11/5	2012/11/9	11	11
122	0	141095	1	26	2012/11/9	2012/11/28	26	26
123	1	141150	0	63	2012/12/6	2013/1/1	30	30
124	1	142997	1	60	2013/1/7	2013/1/14	30	30
125	0	143666	1	59	2013/1/27	2013/1/29	9	9
126	0	144562	0	72	2013/2/28	2013/3/7	14	14
127	0	143440	1	78	2013/3/28	2013/4/2	12	12
128	0	145957	1	50	2013/4/7	2013/4/10	10	10
129	1	130600	1	57	2013/4/11	2013/4/16	12	12
130	0	146162	1	41	2013/4/12	2013/4/21	30	30
131	1	146296	1	55	2013/4/16	2013/4/22	30	30
132	0	146779	1	73	2013/4/30	2013/5/3	30	30
133	0	147547	1	42	2013/5/21	2013/5/27	30	30
134	1	147677	1	59	2013/5/25	2013/6/13	30	30
135	0	147900	1	44	2013/5/31	2016/3/22	1026	30
136	1	148079	0	50	2013/6/4	2013/6/15	18	18
137	0	148190	1	55	2013/6/7	2013/6/19	19	19
138	0	145683	1	40	2013/6/24	2013/7/8	30	30
139	0	148976	1	48	2013/6/28	2013/7/19	30	30
140	0	149241	1	40	2013/7/4	2013/7/10	30	30
141	1	149729	1	54	2013/7/17	2013/7/23	30	30
142	0	149761	1	52	2013/7/17	2013/8/8	30	30
143	0	150900	1	70	2013/8/17	2013/8/23	13	13
144	0	151181	1	48	2013/8/24	2013/9/1	8	8
145	0	151186	1	53	2013/8/25	2013/8/29	11	11
146	0	110339	1	63	2013/9/19	2013/10/4	15	15
147	1	127703	1	44	2013/9/19	2013/9/24	12	12
148	0	112316	0	45	2013/9/28	2013/9/30	9	9
149	0	139855	1	39	2013/10/25	2013/10/25	7	7
150	0	153723	1	74	2013/11/5	2013/11/11	13	13
151	0	154138	1	50	2013/11/16	2013/11/22	30	30

152	0	154356	0	83	2013/11/22	2013/12/5	30	30
153	0	154663	1	39	2013/12/1	2013/12/6	12	12
154	1	145812	1	35	2014/1/7	2014/1/29	30	30
155	1	156619	1	66	2014/1/22	2014/1/28	13	13
156	1	156822	0	52	2014/1/28	2014/2/1	11	11
157	0	101119	0	65	2014/1/30	2014/2/3	30	30
158	0	156942	0	64	2014/2/3	2014/2/8	30	30
159	0	157294	1	50	2014/2/13	2014/2/20	30	30
160	0	155007	1	71	2014/2/26	2014/3/20	30	30
161	0	157985	1	56	2014/3/3	2014/3/7	30	30
162	1	158829	1	50	2014/3/24	2014/4/11	30	30
163	0	147442	1	59	2014/3/28	2014/4/3	30	30
164	1	159897	1	64	2014/4/21	2014/5/4	30	30
165	0	160474	0	57	2014/5/5	2014/5/13	15	15
166	0	159011	1	54	2014/5/20	2014/5/24	11	11
167	0	162429	1	60	2014/6/22	2014/6/28	13	13
168	1	162462	0	87	2014/6/23	2014/7/14	30	30
169	0	162498	1	41	2014/6/23	2014/7/11	30	30
170	0	162838	0	49	2014/7/1	2014/7/10	30	30
171	0	163294	1	51	2014/7/10	2014/7/16	13	13
172	0	163392	1	70	2014/7/14	2014/7/24	30	30
173	1	164084	0	93	2014/7/29	2014/8/6	15	15
174	0	164501	1	49	2014/8/9	2014/8/13	11	11
175	0	165207	1	70	2014/8/26	2014/9/11	30	30
176	1	165528	1	77	2014/9/3	2014/9/16	20	20
177	0	165734	1	52	2014/9/9	2014/9/15	30	30
178	0	165854	1	46	2014/9/11	2014/9/17	13	13
179	0	166116	1	47	2014/9/18	2014/9/22	11	11
180	1	166332	1	24	2014/9/24	2014/10/3	30	30
181	0	166637	1	46	2014/10/4	2014/10/20	30	30
182	0	167299	1	43	2014/10/20	2014/11/15	33	30
183	0	167348	1	49	2014/10/22	2014/11/15	30	30
184	0	164887	0	66	2014/11/2	2014/11/10	8	8
185	1	168473	1	41	2014/11/19	2014/12/1	30	30
186	0	168519	1	49	2014/11/20	2014/12/1	30	30
187	1	168917	1	47	2014/12/1	2014/12/27	33	30
188	0	131200	1	54	2014/12/7	2014/12/12	30	30
189	0	169635	0	38	2014/12/18	2015/1/7	30	30

190	1	169993	1	60	2014/12/27	2015/1/23	30	30
191	1	**436008**	1	75	2014/12/29	2015/1/1	3	3
192	1	170185	1	36	2015/1/2	2015/1/21	30	30
193	1	170377	1	63	2015/1/7	2015/1/9	2	2
194	1	170491	0	44	2015/1/9	2015/1/29	30	30
195	1	170517	1	53	2015/1/10	2015/1/17	30	30
196	0	170549	1	48	2015/1/12	2015/1/27	30	30
197	1	171074	0	57	2015/1/25	2015/2/11	30	30
198	0	172155	0	76	2015/3/2	2015/3/4	30	30
199	0	172960	1	43	2015/3/23	2019/9/5	1627	30
200	1	173341	1	52	2015/4/2	2015/4/9	30	30
201	0	174072	1	53	2015/4/23	2015/5/11	25	25
202	0	170579	1	74	2015/4/25	2015/5/2	7	7
203	0	174883	1	35	2015/5/15	2015/5/18	10	10
204	0	171151	0	77	2015/5/27	2015/5/31	30	30
205	0	175994	0	58	2015/6/14	2015/6/19	12	12
206	0	151073	0	68	2015/6/21	2015/6/26	5	5
207	1	176998	1	42	2015/7/11	2015/7/12	1	1
208	0	177157	1	57	2015/7/15	2015/8/3	30	30
209	0	177666	1	41	2015/7/29	2015/8/19	30	30
210	0	178301	1	42	2015/8/15	2015/8/20	30	30
211	0	166967	0	81	2015/8/27	2015/9/10	30	30
212	0	169552	1	59	2015/9/3	2015/9/11	30	30
213	0	179529	1	45	2015/9/20	2015/9/23	10	10
214	1	174286	0	78	2015/9/25	2015/10/16	28	28
215	0	179857	1	59	2015/9/30	2015/10/11	18	18
216	1	180373	1	48	2015/10/19	2015/10/30	30	30
217	0	180753	1	46	2015/10/29	2015/11/6	30	30
218	0	181255	1	41	2015/11/13	2015/12/10	30	30
219	1	181987	1	58	2015/12/4	2015/12/10	30	30
220	0	182698	1	42	2015/12/24	2015/12/31	30	30
221	0	183017	1	41	2016/1/4	2016/1/5	8	8
222	0	183276	1	47	2016/1/11	2016/1/21	17	17
223	0	184092	0	62	2016/2/2	2016/2/9	30	30
224	0	184118	0	47	2016/2/3	2016/2/27	30	30
225	0	184232	1	74	2016/2/7	2016/2/8	8	8
226	1	185539	1	64	2016/3/14	2016/3/15	8	8
227	1	185689	1	66	2016/3/17	2016/3/22	30	30

228	0	185744	1	46	2016/3/19	2016/3/23	11	11
229	0	172344	1	47	2016/3/25	2016/3/28	10	10
230	0	184178	1	58	2016/4/21	2016/4/24	10	10
231	1	187265	0	63	2016/4/23	2016/5/9	23	23
232	0	187312	1	40	2016/4/25	2016/5/3	30	30
233	0	188289	1	47	2016/5/18	2016/6/9	29	29
234	0	188365	1	53	2016/5/20	2016/6/6	24	24
235	0	188699	1	56	2016/5/29	2016/6/3	12	12
236	0	190714	0	64	2016/7/16	2016/7/18	9	9
237	0	177756	0	67	2016/7/30	2016/7/31	8	8
238	0	191636	1	58	2016/8/8	2016/8/12	11	11
239	0	191731	1	69	2016/8/11	2016/8/17	13	13
240	0	139868	1	82	2016/8/18	2016/9/2	15	15
241	0	192245	1	40	2016/8/24	2016/9/12	30	30
242	0	192526	1	53	2016/8/31	2019/11/11	1167	30
243	0	192665	1	52	2016/9/3	2016/9/13	30	30
244	0	192686	1	68	2016/9/4	2016/9/22	25	25
245	0	192986	1	61	2016/9/12	2016/9/27	30	30
246	0	159610	1	57	2016/9/19	2016/9/22	30	30
247	0	193350	1	52	2016/9/20	2016/10/13	30	30
248	0	194241	0	65	2016/10/14	2016/11/9	30	30
249	0	194573	1	47	2016/10/23	2019/8/6	1017	30
250	0	195059	1	47	2016/11/4	2016/11/15	11	11
251	0	179004	1	80	2016/11/10	2016/11/23	30	30
252	1	**73199**	1	48	2016/11/28	2016/12/5	30	30
253	0	196437	1	59	2016/12/6	2016/12/8	30	30
254	0	196700	1	72	2016/12/13	2016/12/15	30	30
255	0	197550	0	64	2017/1/3	2017/1/10	30	30
256	0	197996	1	53	2017/1/13	2017/1/14	8	8
257	0	124812	1	42	2017/1/30	2019/10/12	985	30
258	1	198593	1	54	2017/2/2	2017/2/14	30	30
259	1	198822	1	52	2017/2/7	2017/2/21	30	30
260	0	166349	1	41	2017/2/15	2017/2/20	12	12
261	0	199300	1	52	2017/2/17	2017/2/28	30	30
262	1	193882	1	65	2017/2/19	2017/3/3	30	30
263	0	199423	1	76	2017/2/21	2017/2/25	11	11
264	1	199794	1	34	2017/3/1	2017/3/9	15	15
265	0	179789	1	64	2017/4/3	2017/4/13	30	30

266	1	201659	1	43	2017/4/14	2017/4/18	30	30
267	1	201727	1	54	2017/4/17	2017/5/12	32	30
268	0	202044	1	51	2017/4/24	2017/5/2	15	15
269	0	202159	1	31	2017/4/26	2017/5/3	30	30
270	0	202317	1	66	2017/5/1	2017/5/3	9	9
271	0	202997	0	71	2017/5/17	2017/5/18	8	8
272	0	204330	1	50	2017/6/20	2017/6/27	30	30
273	1	204744	1	65	2017/6/29	2017/7/5	13	13
274	0	206424	1	62	2017/8/8	2017/8/12	11	11
275	0	206961	1	38	2017/8/21	2017/8/23	30	30
276	0	207524	1	47	2017/9/14	2017/9/17	10	10
277	0	208041	0	54	2017/9/18	2017/9/22	4	4
278	0	141333	0	79	2017/9/25	2017/10/2	14	14
279	0	208846	1	68	2017/10/12	2017/10/17	12	12
280	0	206887	0	52	2017/10/16	2017/10/23	30	30
281	0	210311	0	66	2017/11/16	2017/11/21	12	12
282	0	152073	0	71	2017/11/17	2017/11/21	30	30
283	0	210356	1	62	2017/11/17	2017/11/21	30	30
284	0	210368	1	61	2017/11/17	2017/11/18	30	30
285	0	155119	1	50	2017/12/1	2017/12/6	30	30
286	0	211077	1	54	2017/12/6	2017/12/23	24	24
287	1	211381	0	65	2017/12/13	2017/12/27	30	30
288	1	159971	1	63	2017/12/14	2019/4/11	483	30
289	0	211579	1	53	2017/12/19	2018/1/1	30	30
290	0	211925	1	47	2017/12/27	2018/1/15	30	30
291	1	184530	1	64	2017/12/28	2018/1/2	30	30
292	0	212286	0	41	2018/1/5	2018/1/22	30	30
293	0	212747	1	52	2018/1/17	2018/1/23	13	13
294	0	212818	1	41	2018/1/18	2018/1/26	30	30
295	0	212876	1	41	2018/1/19	2018/2/12	31	30
296	0	184676	1	52	2018/1/31	2018/2/10	30	30
297	1	207837	1	54	2018/2/2	2018/2/18	30	30
298	0	213973	1	75	2018/2/17	2018/3/5	30	30
299	0	186031	1	46	2018/2/20	2018/2/27	30	30
300	1	214090	0	72	2018/2/22	2018/3/8	21	21
301	0	214213	1	63	2018/2/24	2018/3/9	30	30
302	0	215111	1	51	2018/3/16	2018/4/3	30	30
303	1	146590	1	34	2014/1/21	2014/2/18	30	30

304	0	**68858**	1	60	2014/3/7	2014/4/4	28	28
305	0	167606	1	44	2014/12/30	2015/1/27	28	28
306	0	117368	1	40	2010/9/20	2010/10/19	30	30
307	0	136569	0	78	2012/8/24	2012/9/22	29	29
308	0	174419	1	49	2015/5/4	2015/6/2	30	30
309	1	207393	1	35	2017/8/31	2017/9/29	30	30
310	0	214679	1	56	2018/3/6	2018/4/4	30	30
311	0	**95972**	1	39	2009/1/20	2009/2/19	30	30
312	0	181426	1	74	2015/11/17	2015/12/17	30	30
313	0	183775	1	61	2016/1/25	2016/2/24	30	30
314	0	153557	1	54	2017/7/17	2017/8/16	30	30
315	0	138544	1	51	2012/9/9	2012/10/10	31	30
316	1	152187	0	31	2017/8/11	2017/9/11	31	30
317	1	200184	0	62	2017/11/20	2017/12/21	31	30
318	0	209119	1	43	2017/11/20	2017/12/21	31	30
319	0	102270	1	45	2009/8/15	2009/9/16	32	30
320	1	168065	0	62	2014/11/9	2014/12/11	32	30
321	1	190736	0	66	2016/7/17	2016/8/18	32	30
322	0	169074	1	48	2017/10/13	2017/11/14	32	30
323	0	171982	1	50	2015/2/26	2015/3/31	33	30
324	0	205577	1	47	2017/7/19	2017/8/21	33	30
325	0	180517	1	45	2015/10/22	2015/11/25	34	30
326	0	114938	1	59	2010/6/24	2010/7/29	35	30
327	0	130887	0	63	2012/1/12	2012/2/16	35	30
328	1	172079	1	58	2017/1/5	2017/2/9	35	30
329	0	134346	0	57	2012/5/2	2012/6/7	36	30
330	0	136692	1	56	2012/7/3	2012/8/8	36	30
331	0	156331	1	68	2014/1/14	2014/2/19	36	30
332	0	172529	1	49	2015/3/11	2015/4/16	36	30
333	0	190422	1	45	2016/7/10	2016/8/15	36	30
334	0	193102	0	66	2016/10/24	2016/11/29	36	30
335	1	192100	1	44	2016/12/14	2017/1/19	36	30
336	0	155637	1	61	2014/3/6	2014/4/12	37	30
337	0	202120	1	63	2017/4/25	2017/6/1	37	30
338	0	209213	1	50	2017/10/21	2017/11/27	37	30
339	0	150419	1	41	2013/8/5	2013/9/12	38	30
340	0	125315	1	47	2011/7/13	2011/8/20	38	30
341	0	157077	1	38	2014/2/7	2014/3/18	39	30

342	1	156883	1	52	2014/6/14	2014/7/23	39	30
343	0	191402	1	48	2016/8/2	2016/9/10	39	30
344	0	192723	1	36	2016/9/5	2016/10/14	39	30
345	1	203767	1	51	2017/6/6	2017/7/15	39	30
346	0	182623	0	51	2015/12/22	2016/1/31	40	30
347	0	**95784**	1	71	2009/8/12	2009/9/22	41	30
348	0	114247	0	57	2010/6/3	2010/7/14	41	30
349	0	137210	1	57	2013/8/10	2013/9/20	41	30
350	0	157598	0	59	2014/2/20	2014/4/2	41	30
351	0	145281	1	41	2015/5/1	2015/6/11	41	30
352	0	174893	1	44	2016/7/30	2016/9/9	41	30
353	0	149073	1	60	2013/7/1	2013/8/12	42	30
354	0	161844	0	57	2014/6/8	2014/7/20	42	30
355	0	194947	1	34	2016/11/1	2016/12/13	42	30
356	0	103828	1	66	2009/3/17	2009/4/29	43	30
357	0	106962	1	47	2009/7/8	2009/8/20	43	30
358	1	111667	1	30	2010/2/20	2010/4/4	43	30
359	0	114435	1	58	2010/6/9	2010/7/22	43	30
360	0	127254	1	46	2011/9/14	2011/10/27	43	30
361	0	134522	0	36	2012/5/7	2012/6/19	43	30
362	0	215489	1	33	2018/3/26	2018/5/8	43	30
363	0	111467	0	77	2010/2/6	2010/3/22	44	30
364	0	128933	1	67	2011/11/8	2011/12/22	44	30
365	0	154086	1	53	2013/11/14	2013/12/28	44	30
366	0	109902	1	40	2009/11/17	2010/1/1	45	30
367	0	126422	1	57	2011/10/3	2011/11/17	45	30
368	0	126667	1	51	2011/10/5	2011/11/19	45	30
369	0	125099	0	48	2011/12/21	2012/2/4	45	30
370	0	206703	1	38	2017/8/15	2017/9/29	45	30
371	0	184176	1	60	2016/2/5	2016/3/22	46	30
372	0	101785	1	51	2008/10/24	2008/12/10	47	30
373	0	136103	1	48	2012/6/17	2012/8/3	47	30
374	0	**96525**	1	68	2008/11/16	2009/1/3	48	30
375	0	128352	1	48	2011/10/21	2011/12/8	48	30
376	0	210047	1	60	2017/11/10	2017/12/28	48	30
377	0	84937	0	69	2010/8/27	2010/10/15	49	30
378	0	111006	0	69	2010/2/8	2010/3/30	50	30
379	0	112891	1	45	2010/4/15	2010/6/4	50	30

380	0	133466	1	54	2012/4/5	2012/5/25	50	30
381	1	157962	1	47	2014/3/3	2014/4/22	50	30
382	0	171201	1	61	2017/8/27	2017/10/16	50	30
383	1	215011	1	68	2018/3/14	2018/5/3	50	30
384	1	102781	1	47	2009/1/8	2009/3/5	56	30
385	0	114719	0	48	2010/6/18	2010/8/8	51	30
386	0	150349	1	53	2015/11/27	2016/1/17	51	30
387	0	192213	1	64	2017/4/22	2017/6/13	52	30
388	1	132850	1	43	2012/3/16	2012/5/8	53	30
389	1	183861	0	65	2016/1/27	2016/3/21	54	30
390	1	188574	1	49	2016/5/25	2016/7/18	54	30
391	1	172431	1	46	2015/3/10	2015/5/4	55	30
392	0	185576	0	51	2016/3/15	2016/5/9	55	30
393	0	**99993**	1	50	2012/8/13	2012/10/8	56	30
394	1	184947	1	32	2016/2/29	2016/4/25	56	30
395	0	128621	1	61	2011/10/30	2011/12/26	57	30
396	1	131577	1	50	2012/2/9	2012/4/6	57	30
397	0	170490	1	52	2015/1/9	2015/3/7	57	30
398	1	102050	0	78	2009/1/9	2009/3/8	58	30
399	0	103873	1	56	2009/3/20	2009/5/17	58	30
400	0	134845	1	52	2012/8/22	2012/10/19	58	30
401	0	145451	0	51	2013/3/24	2013/5/22	59	30
402	0	206686	1	27	2017/8/14	2017/10/12	59	30
403	0	147671	1	64	2013/5/24	2013/7/23	60	30
404	1	146435	1	58	2013/7/3	2013/9/2	61	30
405	0	158670	1	60	2014/5/15	2014/7/15	61	30
406	0	**71738**	0	31	2009/1/6	2009/3/9	62	30
407	0	107286	1	57	2010/10/12	2010/12/13	62	30
408	0	127888	0	48	2011/10/5	2011/12/6	62	30
409	0	153467	0	65	2013/10/29	2013/12/30	62	30
410	0	208584	1	30	2018/4/4	2018/6/6	63	30
411	0	124708	0	68	2011/9/20	2011/11/23	64	30
412	0	203417	1	49	2017/5/29	2017/8/1	64	30
413	0	207974	0	53	2017/9/15	2017/11/18	64	30
414	1	211626	1	51	2018/3/21	2018/5/24	64	30
415	0	134714	1	51	2012/8/20	2012/10/24	65	30
416	0	101960	1	37	2011/11/21	2012/1/27	67	30
417	0	137471	0	54	2014/6/3	2014/8/9	67	30

418	1	107697	1	65	2009/8/5	2009/10/12	68	30
419	0	114651	0	67	2010/8/17	2010/10/24	68	30
420	1	108797	1	53	2009/9/24	2009/12/1	68	30
421	0	169226	1	66	2014/12/8	2015/2/15	69	30
422	0	110178	1	40	2009/12/1	2010/2/9	70	30
423	0	122933	0	70	2011/5/3	2011/7/12	70	30
424	0	133412	1	44	2012/4/4	2012/6/13	70	30
425	0	165552	0	59	2014/9/4	2014/11/13	70	30
426	1	119393	1	52	2010/12/16	2011/2/25	71	30
427	0	201810	1	56	2017/4/18	2017/6/28	71	30
428	0	174396	0	73	2017/9/10	2017/11/20	71	30
429	0	200439	1	53	2017/3/15	2017/5/27	73	30
430	0	209659	1	46	2017/11/1	2018/1/14	74	30
431	1	107188	0	50	2009/7/18	2009/10/1	75	30
432	0	97905	1	53	2010/3/5	2010/5/20	76	30
433	0	210595	1	59	2017/11/23	2018/2/8	77	30
434	1	186181	1	52	2016/3/29	2016/6/15	78	30
435	0	180359	1	62	2016/1/27	2016/4/15	79	30
436	0	109552	1	31	2009/10/30	2010/1/18	80	30
437	1	122880	0	52	2014/2/15	2014/5/6	80	30
438	1	103282	1	51	2009/10/1	2009/12/21	81	30
439	0	196190	1	58	2016/12/1	2017/2/20	81	30
440	0	175479	1	54	2016/3/16	2016/6/6	82	30
441	0	182110	0	71	2015/12/8	2016/2/29	83	30
442	0	214266	1	60	2018/2/25	2018/5/22	86	30
443	0	101704	0	57	2008/10/18	2009/1/13	87	30
444	0	188916	1	35	2016/10/4	2016/12/30	87	30
445	0	121427	1	74	2012/7/10	2012/10/6	88	30
446	0	176189	0	69	2015/6/18	2015/9/14	88	30
447	0	193552	1	51	2016/9/26	2016/12/24	89	30
448	0	160258	1	51	2014/4/29	2014/7/28	90	30
449	0	206150	1	49	2017/8/1	2017/10/30	90	30
450	0	167274	1	47	2015/8/6	2015/11/5	91	30
451	0	214699	1	61	2018/3/28	2018/6/27	91	30
452	1	112732	0	67	2010/4/9	2010/7/13	95	30
453	0	137173	0	59	2012/7/17	2012/10/21	96	30
454	0	134173	1	56	2012/10/18	2013/1/25	99	30
455	0	183918	0	49	2016/1/28	2016/5/7	100	30

456	1	76500	1	75	2014/7/18	2014/10/29	103	30
457	0	141254	1	46	2012/11/14	2013/2/26	104	30
458	0	112935	1	51	2013/7/31	2013/11/12	104	30
459	0	142708	1	62	2013/5/7	2013/8/22	107	30
460	0	162142	1	52	2014/6/15	2014/9/30	107	30
461	1	178506	1	55	2018/1/12	2018/4/29	107	30
462	1	162038	0	65	2014/6/12	2014/9/29	109	30
463	0	127302	1	39	2011/9/15	2012/1/4	111	30
464	0	150920	1	50	2016/11/9	2017/2/28	111	30
465	0	201344	1	54	2017/4/7	2017/7/27	111	30
466	0	111796	1	57	2010/2/26	2010/6/22	116	30
467	0	208550	1	62	2017/10/1	2018/1/26	117	30
468	0	210367	1	78	2017/11/17	2018/3/14	117	30
469	0	215249	1	60	2018/3/20	2018/7/19	121	30
470	1	118642	1	53	2010/11/17	2011/3/19	122	30
471	0	129042	1	47	2015/7/29	2015/12/2	126	30
472	0	111294	1	49	2010/1/29	2010/6/4	126	30
473	0	103726	1	45	2017/6/19	2017/10/24	127	30
474	0	202559	1	59	2017/5/7	2017/9/13	129	30
475	1	170069	1	56	2016/3/7	2016/7/15	130	30
476	0	160855	0	51	2016/3/20	2016/7/28	130	30
477	1	195783	1	55	2016/11/21	2017/3/31	130	30
478	0	150817	1	39	2013/8/14	2013/12/27	135	30
479	0	170180	0	65	2016/2/24	2016/7/8	135	30
480	1	203328	1	54	2017/6/12	2017/10/25	135	30
481	0	188902	1	47	2018/3/26	2018/8/10	137	30
482	0	145788	1	38	2013/4/2	2013/8/19	139	30
483	0	202474	1	71	2017/7/25	2017/12/11	139	30
484	0	135196	1	57	2012/6/20	2012/11/7	140	30
485	0	215682	0	72	2018/3/30	2018/8/17	140	30
486	0	145507	0	61	2014/9/7	2015/1/27	142	30
487	0	169436	1	56	2016/3/5	2016/7/26	143	30
488	1	172891	1	50	2015/3/22	2015/8/18	149	30
489	1	200908	1	77	2017/5/27	2017/10/24	150	30
490	1	211699	1	46	2018/1/15	2018/6/14	150	30
491	0	133070	0	50	2012/3/22	2012/8/21	152	30
492	1	141583	1	51	2016/3/2	2016/8/1	152	30
493	0	107862	1	28	2009/8/12	2010/1/12	153	30

494	1	170463	1	55	2015/1/8	2015/6/10	153	30
495	0	193590	1	52	2017/8/17	2018/1/17	153	30
496	1	148473	1	54	2015/9/7	2016/2/9	155	30
497	1	108135	1	49	2009/8/24	2010/1/27	156	30
498	1	190021	1	76	2017/3/24	2017/8/27	156	30
499	1	130986	1	58	2012/2/17	2012/7/23	157	30
500	0	145889	1	37	2013/4/5	2013/9/9	157	30
501	0	178673	1	48	2017/9/7	2018/2/11	157	30
502	0	91155	0	57	2010/11/1	2011/4/10	160	30
503	0	160790	1	59	2014/5/13	2014/10/22	162	30
504	1	112862	1	53	2011/5/11	2011/10/21	163	30
505	1	117385	1	51	2010/10/22	2011/4/7	167	30
506	0	204747	1	54	2017/6/29	2017/12/14	168	30
507	1	163870	1	70	2014/7/24	2015/1/11	171	30
508	0	172852	1	47	2015/11/17	2016/5/6	171	30
509	0	131186	1	52	2012/1/27	2012/7/18	173	30
510	1	170139	1	76	2015/4/26	2015/10/19	176	30
511	0	184611	1	60	2016/5/7	2016/10/31	177	30
512	1	208530	1	48	2018/3/21	2018/9/17	180	30
513	0	104438	0	46	2009/4/19	2009/10/19	183	30
514	0	180478	1	42	2017/12/13	2018/6/14	183	30
515	0	140432	1	42	2012/10/22	2013/4/26	186	30
516	0	117297	1	56	2010/9/16	2011/3/31	196	30
517	0	215586	1	66	2018/3/28	2018/10/12	198	30
518	0	213387	0	61	2018/4/7	2018/10/25	201	30
519	1	103528	1	50	2009/3/2	2009/9/22	204	30
520	0	174975	1	78	2018/3/9	2018/9/30	205	30
521	1	215846	1	47	2018/4/3	2018/10/26	206	30
522	1	210307	1	44	2017/11/16	2018/6/14	210	30
523	0	185565	1	52	2018/3/31	2018/10/27	210	30
524	0	209224	1	64	2017/10/22	2018/5/22	212	30
525	1	172179	1	52	2015/6/3	2016/1/2	213	30
526	0	170205	1	47	2015/5/27	2015/12/27	214	30
527	0	178016	1	59	2018/2/5	2018/9/7	214	30
528	0	207456	0	60	2018/3/5	2018/10/8	217	30
529	1	214613	1	54	2018/3/5	2018/10/8	217	30
530	1	106914	1	60	2009/12/21	2010/7/27	218	30
531	0	213917	0	54	2018/2/14	2018/9/20	218	30

532	0	105445	0	54	2009/5/26	2009/12/31	219	30
533	0	177558	1	63	2015/7/27	2016/3/2	219	30
534	0	181397	1	54	2017/2/20	2017/9/28	220	30
535	0	164389	1	62	2018/2/19	2018/9/27	220	30
536	1	185149	1	52	2016/3/4	2016/10/12	222	30
537	0	143142	1	74	2013/1/10	2013/8/23	225	30
538	0	143748	0	63	2013/4/3	2013/11/14	225	30
539	1	211282	0	52	2017/12/11	2018/7/24	225	30
540	1	141606	0	76	2012/11/26	2013/7/10	226	30
541	0	**357562**	0	37	2017/10/30	2018/6/13	226	30
542	0	**114877**	1	46	2009/8/6	2010/3/21	227	30
543	0	113118	1	53	2010/4/24	2010/12/7	227	30
544	0	**109609**	0	58	2009/9/30	2009/10/22	30	30
545	1	104920	1	54	2009/11/24	2010/7/10	228	30
546	1	141660	1	41	2013/4/12	2013/11/27	229	30
547	1	138692	1	72	2013/8/10	2014/3/27	229	30
548	1	214409	1	25	2018/2/28	2018/10/17	231	30
549	0	170910	0	60	2017/9/2	2018/4/27	237	30
550	0	203404	1	45	2017/5/28	2018/1/24	241	30
551	0	213038	0	70	2018/1/23	2018/9/21	241	30
552	1	164795	1	58	2014/8/17	2015/4/17	243	30
553	0	190232	1	57	2016/10/14	2017/6/14	243	30
554	0	195184	0	54	2017/1/29	2017/9/30	244	30
555	1	127852	1	64	2012/7/1	2013/3/4	246	30
556	0	103813	1	41	2009/4/26	2009/12/29	247	30
557	1	161382	1	66	2014/5/27	2015/1/30	248	30
558	0	108798	1	56	2016/1/25	2016/10/2	251	30
559	1	206757	1	51	2017/8/16	2018/4/26	253	30
560	1	112031	1	71	2018/1/10	2018/9/20	253	30
561	0	190839	1	61	2016/10/24	2017/7/7	256	30
562	0	212015	0	80	2017/12/29	2018/9/13	258	30
563	0	130184	0	70	2011/12/18	2012/9/3	260	30
564	0	213519	1	40	2018/2/3	2018/10/25	264	30
565	0	109355	1	52	2016/8/21	2017/5/15	267	30
566	0	202840	1	55	2017/5/13	2018/2/5	268	30
567	0	139977	1	79	2012/10/8	2013/7/4	269	30
568	1	211277	0	55	2017/12/11	2018/9/6	269	30
569	0	**96497**	1	29	2014/9/1	2015/5/30	271	30

570	0	139308	1	48	2016/5/5	2017/2/6	277	30
571	0	211198	1	57	2017/12/9	2018/9/12	277	30
572	1	207229	0	56	2017/8/28	2018/6/3	279	30
573	1	195034	1	50	2016/11/30	2017/9/6	280	30
574	0	210390	1	48	2017/11/19	2018/8/28	282	30
575	1	182578	1	57	2016/9/22	2017/7/2	283	30
576	0	188491	1	54	2017/12/18	2018/9/30	286	30
577	0	179235	1	61	2015/10/16	2016/8/1	290	30
578	1	194541	1	58	2016/10/21	2017/8/8	291	30
579	0	143429	1	57	2014/6/29	2015/4/17	292	30
580	0	210192	1	46	2017/11/14	2018/9/3	293	30
581	0	165357	1	54	2016/9/26	2017/7/18	295	30
582	0	137385	1	60	2017/5/9	2018/3/1	296	30
583	0	201884	1	45	2017/8/29	2018/6/21	296	30
584	0	194811	1	70	2016/10/28	2017/8/28	304	30
585	0	126209	1	46	2017/11/7	2018/9/8	305	30
586	0	210385	1	35	2017/11/18	2018/9/20	306	30
587	1	183518	1	30	2016/1/18	2016/11/21	308	30
588	0	203624	1	60	2017/6/2	2018/4/6	308	30
589	0	202695	0	50	2017/6/7	2018/4/11	308	30
590	0	187955	1	63	2017/11/23	2018/9/28	309	30
591	1	154902	1	47	2013/12/6	2014/10/12	310	30
592	0	171308	1	52	2016/9/24	2017/8/1	311	30
593	1	139557	1	64	2014/6/9	2015/4/17	312	30
594	1	186123	1	56	2016/4/28	2017/3/10	316	30
595	0	120006	1	50	2012/2/29	2013/1/11	317	30
596	0	101086	1	56	2011/5/3	2012/3/20	322	30
597	0	211080	1	45	2017/12/6	2018/10/24	322	30
598	0	121890	1	57	2011/3/29	2012/2/19	327	30
599	0	202658	0	52	2017/5/9	2018/4/3	329	30
600	0	191362	1	46	2017/11/21	2018/10/16	329	30
601	0	210572	0	73	2017/11/23	2018/10/19	330	30
602	0	210080	1	49	2017/11/11	2018/10/11	334	30
603	0	**91958**	1	54	2008/11/3	2009/10/4	335	30
604	0	200204	1	71	2017/3/10	2018/2/8	335	30
605	0	206561	1	62	2017/8/11	2018/7/13	336	30
606	0	180103	1	57	2017/10/20	2018/9/25	340	30
607	0	156967	0	59	2014/2/15	2015/1/24	343	30

608	0	183639	1	64	2017/1/5	2017/12/14	343	30
609	0	109854	1	46	2009/11/13	2010/10/26	347	30
610	0	200348	0	77	2017/5/27	2018/5/9	347	30
611	0	118965	0	61	2011/10/4	2012/9/17	349	30
612	0	166681	0	63	2017/6/2	2018/5/19	351	30
613	0	101739	1	50	2008/10/21	2009/10/8	352	30
614	0	111300	1	65	2010/1/29	2011/1/21	357	30
615	0	146679	1	55	2015/4/27	2016/4/18	357	30
616	1	203284	1	55	2017/5/25	2018/5/17	357	30
617	0	209648	1	62	2017/11/1	2018/10/24	357	30
618	0	117506	1	59	2010/9/27	2011/9/21	359	30
619	0	**87789**	1	44	2011/1/13	2012/1/10	362	30
620	0	139242	1	78	2017/4/26	2018/4/23	362	30
621	0	182953	1	53	2017/3/2	2018/3/2	365	30
622	0	187514	0	79	2017/10/27	2018/10/27	365	30
623	0	200433	1	56	2017/3/15	2018/3/16	366	30
624	1	120189	1	35	2011/10/18	2012/10/19	367	30
625	0	206625	1	47	2017/8/13	2018/8/15	367	30
626	0	85855	1	66	2017/5/12	2018/5/16	369	30
627	0	161653	1	56	2017/8/28	2018/9/5	373	30
628	0	167880	1	66	2014/12/15	2015/12/24	374	30
629	1	159358	1	61	2015/8/11	2016/8/23	378	30
630	1	100053	1	49	2009/4/18	2010/5/4	381	30
631	1	110885	1	56	2010/1/7	2011/1/23	381	30
632	0	133685	1	56	2013/6/8	2014/6/24	381	30
633	0	205968	1	52	2017/9/28	2018/10/17	384	30
634	0	120511	1	34	2011/2/3	2012/2/23	385	30
635	0	194344	0	71	2017/3/26	2018/4/20	390	30
636	0	119796	1	75	2011/1/1	2012/2/3	398	30
637	1	201325	1	38	2017/4/6	2018/5/13	402	30
638	0	147174	1	49	2013/5/11	2014/6/18	403	30
639	0	169700	1	46	2014/12/19	2016/1/26	403	30
640	0	188915	1	76	2017/7/11	2018/8/19	404	30
641	0	180126	1	32	2016/6/3	2017/7/14	406	30
642	1	112717	1	62	2010/4/9	2011/5/21	407	30
643	0	153992	1	51	2014/4/27	2015/6/9	408	30
644	0	204886	1	59	2017/7/3	2018/8/19	412	30
645	1	206988	1	68	2017/8/22	2019/1/4	500	30

646	0	186792	0	50	2017/7/19	2018/9/5	413	30
647	1	152335	1	46	2013/9/25	2014/11/14	415	30
648	1	156670	1	43	2014/10/13	2015/12/7	420	30
649	0	166145	1	54	2016/12/8	2018/2/1	420	30
650	0	206163	1	43	2017/8/1	2018/9/25	420	30
651	0	151288	1	44	2017/8/23	2018/10/17	420	30
652	0	**77254**	1	68	2016/8/3	2017/9/28	421	30
653	0	169427	1	45	2015/11/25	2017/1/20	422	30
654	0	171424	1	53	2015/11/13	2017/1/9	423	30
655	0	206967	1	57	2017/8/21	2018/10/18	423	30
656	0	206626	1	55	2017/8/13	2018/10/11	424	30
657	0	181968	1	42	2017/8/23	2018/10/23	426	30
658	0	131286	1	60	2017/8/28	2018/10/28	426	30
659	1	197405	1	44	2016/12/29	2018/3/1	427	30
660	0	146478	1	50	2016/4/4	2017/6/6	428	30
661	0	179085	1	38	2016/3/30	2017/6/8	435	30
662	0	205141	1	38	2017/7/9	2018/9/19	437	30
663	1	144670	1	53	2015/8/18	2016/11/4	444	30
664	0	205706	1	42	2017/7/22	2018/10/10	445	30
665	0	202273	1	52	2017/8/4	2019/2/27	572	30
666	0	132755	1	51	2012/3/14	2013/6/3	446	30
667	1	131679	0	71	2012/11/23	2014/2/12	446	30
668	0	102340	1	51	2008/12/10	2010/3/2	447	30
669	0	103594	0	77	2009/10/12	2010/5/30	230	30
670	0	**142785**	1	58	2017/7/25	2018/10/24	456	30
671	1	111538	0	51	2015/1/3	2016/4/8	461	30
672	1	148590	1	50	2013/6/19	2014/9/25	463	30
673	0	**199577**	1	44	2009/11/28	2011/3/9	466	30
674	0	132485	1	54	2016/3/24	2017/7/5	468	30
675	1	109099	1	49	2009/10/12	2011/1/24	469	30
676	0	184881	1	57	2017/7/9	2018/10/24	472	30
677	0	161529	1	51	2014/5/31	2015/9/19	476	30
678	0	156725	1	45	2016/12/15	2018/4/12	483	30
679	0	**71085**	1	54	2010/1/23	2011/5/26	488	30
680	0	200719	1	48	2017/3/22	2018/7/26	491	30
681	1	195665	1	37	2016/11/18	2018/3/26	493	30
682	0	156486	1	59	2014/1/19	2015/5/29	495	30
683	0	182811	0	71	2015/12/28	2017/5/7	496	30

684	1	169191	1	49	2015/2/28	2016/7/9	497	30
685	0	139846	1	52	2012/10/4	2014/2/14	498	30
686	0	163625	1	29	2014/11/2	2016/3/14	498	30
687	0	197815	0	70	2017/1/9	2018/5/22	498	30
688	1	154511	1	62	2013/11/26	2015/4/10	500	30
689	0	132469	1	43	2013/2/7	2014/6/26	504	30
690	0	165151	1	47	2014/8/25	2016/1/12	505	30
691	0	156743	1	46	2014/1/26	2015/6/16	506	30
692	0	194943	0	74	2016/11/1	2018/3/23	507	30
693	0	192591	1	54	2017/4/29	2018/9/18	507	30
694	0	**213075**	1	68	2010/3/8	2011/8/9	519	30
695	0	118911	1	49	2017/4/4	2018/9/12	526	30
696	0	139149	0	68	2017/4/29	2018/10/9	528	30
697	1	143188	1	51	2016/12/9	2018/5/22	529	30
698	0	118533	1	45	2013/6/25	2014/12/9	532	30
699	0	201144	0	75	2017/4/2	2018/9/18	534	30
700	0	202368	1	55	2017/5/2	2018/10/18	534	30
701	0	165180	1	80	2014/9/18	2016/3/11	540	30
702	0	114814	1	60	2011/5/21	2012/11/15	544	30
703	0	107748	1	54	2009/9/23	2011/3/21	544	30
704	0	194282	1	46	2016/10/16	2018/4/14	545	30
705	1	**100910**	1	55	2014/6/16	2015/12/21	553	30
706	0	115758	1	63	2014/3/6	2015/9/14	557	30
707	0	111556	1	45	2010/2/11	2011/8/23	558	30
708	0	127166	1	59	2011/10/19	2013/5/7	566	30
709	1	191633	1	38	2017/3/17	2018/10/5	567	30
710	0	148788	1	64	2016/1/25	2017/8/15	568	30
711	0	184255	0	65	2016/3/22	2017/10/11	568	30
712	0	191826	1	45	2016/8/13	2018/3/5	569	30
713	1	156365	1	80	2017/1/16	2018/8/11	572	30
714	1	198493	1	51	2017/1/28	2018/9/2	582	30
715	0	103633	1	44	2009/10/17	2011/6/1	592	30
716	0	170938	1	55	2015/1/21	2016/9/9	597	30
717	0	162001	0	58	2014/12/10	2016/7/30	598	30
718	1	139935	1	41	2014/11/20	2016/7/22	610	30
719	1	192156	1	38	2017/1/20	2018/9/22	610	30
720	0	188231	1	53	2016/9/9	2018/5/16	614	30
721	0	148347	1	55	2015/8/20	2017/5/10	629	30

722	1	140373	1	64	2012/10/21	2014/7/17	634	30
723	0	175761	1	37	2015/6/8	2017/3/3	634	30
724	0	198052	1	49	2017/1/15	2018/10/11	634	30
725	1	**65986**	1	66	2017/1/16	2018/10/12	634	30
726	1	188677	1	35	2016/5/28	2018/2/23	636	30
727	0	106768	1	51	2009/7/2	2011/4/11	648	30
728	1	115813	1	41	2016/12/19	2018/10/4	654	30
729	0	**54895**	1	59	2016/10/10	2018/7/27	655	30
730	0	112627	1	61	2016/12/11	2018/9/27	655	30
731	0	180206	0	56	2016/12/23	2018/10/13	659	30
732	1	111154	1	49	2013/4/19	2015/2/8	660	30
733	0	196131	1	45	2016/11/30	2018/9/21	660	30
734	0	197736	1	35	2017/1/7	2018/10/29	660	30
735	0	103400	0	80	2009/2/21	2010/12/17	664	30
736	0	173324	1	47	2015/4/16	2017/2/20	676	30
737	1	104151	1	54	2012/6/5	2014/4/22	686	30
738	0	189094	1	31	2016/6/7	2018/4/25	687	30
739	0	178360	1	72	2016/11/28	2018/10/16	687	30
740	0	169744	1	48	2014/12/20	2016/11/8	689	30
741	0	113915	1	62	2014/8/31	2016/7/21	690	30
742	0	195860	1	48	2016/11/23	2018/10/17	693	30
743	0	**89609**	1	45	2016/6/23	2018/5/22	698	30
744	1	121795	1	53	2011/5/5	2013/4/12	708	30
745	0	192643	1	49	2016/9/2	2018/8/22	719	30
746	1	102692	1	49	2013/1/28	2015/1/22	724	30
747	0	187659	1	48	2016/5/3	2018/4/27	724	30
748	0	118141	0	74	2012/5/30	2014/5/29	729	30
749	1	193530	0	56	2016/9/25	2018/9/26	731	30
750	0	188754	1	63	2016/10/17	2018/10/29	742	30
751	1	**33378**	1	49	2009/5/18	2011/6/5	748	30
752	0	166908	1	53	2014/10/11	2016/11/2	753	30
753	1	116888	1	64	2010/9/1	2012/9/24	754	30
754	1	192951	1	46	2016/9/10	2018/10/17	767	30
755	0	192898	1	62	2016/9/8	2018/10/17	769	30
756	0	192945	1	35	2016/9/9	2018/10/24	775	30
757	0	151963	1	48	2015/5/19	2017/7/3	776	30
758	1	132898	1	64	2016/8/30	2018/10/19	780	30
759	0	154246	1	59	2014/10/13	2016/12/2	781	30

760	0	127301	1	46	2016/3/4	2018/4/30	787	30
761	0	190659	1	39	2016/7/15	2018/9/11	788	30
762	0	192305	1	50	2016/8/25	2018/10/24	790	30
763	0	159604	1	47	2014/4/12	2016/6/17	797	30
764	0	189872	0	71	2016/6/27	2018/9/2	797	30
765	1	175950	1	41	2016/8/14	2018/10/22	799	30
766	0	120489	1	51	2012/1/25	2014/4/18	814	30
767	0	179952	0	77	2015/10/27	2018/1/21	817	30
768	0	189178	1	53	2016/6/10	2018/9/6	818	30
769	0	122365	1	60	2012/4/10	2014/7/11	822	30
770	0	156529	1	53	2015/12/4	2018/3/6	823	30
771	1	175469	1	52	2016/6/23	2018/9/27	826	30
772	1	145256	1	53	2013/3/19	2015/6/26	829	30
773	0	181060	1	45	2015/11/6	2018/2/24	841	30
774	0	129153	1	41	2012/4/12	2014/8/4	844	30
775	0	188261	1	52	2016/6/23	2018/10/16	845	30
776	0	134439	1	54	2014/12/18	2017/4/16	850	30
777	0	148876	1	43	2014/11/18	2017/3/19	852	30
778	0	**87138**	0	74	2014/5/22	2016/9/28	860	30
779	0	**99826**	1	56	2009/10/3	2012/2/13	863	30
780	1	115449	1	71	2010/7/9	2012/11/22	867	30
781	0	142960	1	43	2016/3/9	2018/7/24	867	30
782	0	144282	1	37	2013/3/22	2015/8/10	871	30
783	0	175689	1	70	2015/6/5	2017/10/27	875	30
784	0	177180	1	56	2016/4/15	2018/9/14	882	30
785	0	130692	1	70	2012/1/5	2014/6/6	883	30
786	0	132762	1	62	2013/3/9	2015/8/9	883	30
787	1	**85357**	1	72	2013/5/14	2015/10/26	895	30
788	0	174306	1	65	2016/2/29	2018/8/21	904	30
789	0	103418	1	48	2010/11/10	2013/5/7	909	30
790	0	174950	1	50	2015/6/8	2017/12/7	913	30
791	0	170019	1	56	2016/1/29	2018/8/10	924	30
792	0	144227	1	63	2014/8/9	2017/2/19	925	30
793	0	160295	0	54	2016/4/3	2018/10/15	925	30
794	0	152772	0	63	2013/11/16	2016/5/31	927	30
795	0	125266	1	65	2016/3/15	2018/10/16	945	30
796	0	144221	1	71	2013/8/13	2016/3/17	947	30
797	1	153571	1	46	2014/5/3	2016/12/8	950	30

798	0	104722	1	53	2009/5/4	2011/12/15	955	30
799	0	99141	1	78	2009/7/27	2012/3/9	956	30
800	0	140640	1	48	2016/2/18	2018/10/9	964	30
801	0	169254	1	40	2015/12/8	2018/8/1	967	30
802	0	**98095**	1	47	2009/2/26	2011/10/24	970	30
803	0	185015	1	46	2016/3/1	2018/12/16	1020	30
804	0	179227	1	47	2015/9/11	2018/5/24	986	30
805	1	157654	1	66	2014/2/22	2016/11/7	989	30
806	0	183336	1	55	2016/2/8	2018/10/27	992	30
807	1	171743	1	30	2015/2/14	2017/11/13	1003	30
808	0	129492	0	52	2015/11/16	2018/8/16	1004	30
809	0	**93376**	1	43	2009/3/23	2011/12/26	1008	30
810	0	105059	1	66	2009/6/3	2012/3/17	1018	30
811	1	172542	1	62	2015/3/12	2017/12/28	1022	30
812	0	146666	1	42	2015/12/8	2018/9/25	1022	30
813	0	114410	1	61	2010/6/8	2013/4/3	1030	30
814	0	125609	1	33	2012/1/8	2014/11/3	1030	30
815	1	174935	0	60	2015/7/25	2018/5/23	1033	30
816	0	165406	0	61	2015/12/28	2018/10/26	1033	30
817	0	123551	1	51	2014/2/28	2016/12/28	1034	30
818	0	101411	1	47	2008/9/26	2011/7/28	1035	30
819	1	168209	1	59	2015/12/15	2018/10/15	1035	30
820	0	155375	0	65	2013/12/19	2016/10/26	1042	30
821	0	140752	1	39	2014/6/20	2017/4/28	1043	30
822	0	181918	1	56	2015/12/2	2018/10/10	1043	30
823	0	170393	1	33	2015/1/7	2017/11/22	1050	30
824	0	164976	1	32	2014/12/23	2017/11/10	1053	30
825	0	175945	1	29	2015/6/12	2018/5/8	1061	30
826	0	104425	1	60	2009/12/21	2012/11/23	1068	30
827	0	158125	1	31	2015/3/6	2018/2/16	1078	30
828	0	171206	1	40	2015/1/28	2018/1/11	1079	30
829	1	131127	1	60	2012/9/1	2015/8/20	1083	30
830	1	134254	1	40	2012/4/29	2015/4/19	1085	30
831	0	176225	0	74	2015/8/29	2019/4/3	1313	30
832	0	126568	1	48	2012/12/12	2015/12/10	1093	30
833	1	128203	1	61	2015/5/23	2018/5/23	1096	30
834	0	128161	1	63	2013/4/25	2016/5/10	1111	30
835	0	152178	0	58	2013/9/22	2016/10/15	1119	30

836	1	**95005**	1	51	2015/8/31	2018/9/25	1121	30
837	0	122823	1	44	2015/9/10	2018/10/8	1124	30
838	0	175026	1	53	2015/9/20	2018/10/22	1128	30
839	0	172332	1	47	2015/3/6	2018/4/12	1133	30
840	1	144791	1	58	2015/3/27	2018/5/12	1142	30
841	1	173072	0	68	2015/3/26	2018/5/30	1161	30
842	0	146735	1	47	2015/3/20	2018/5/27	1164	30
843	1	178302	1	46	2015/8/15	2018/10/22	1164	30
844	0	170485	1	40	2015/1/24	2018/4/9	1171	30
845	0	144164	0	64	2013/9/3	2016/12/3	1187	30
846	1	123952	1	39	2011/12/17	2015/3/24	1193	30
847	0	144148	1	55	2015/2/16	2018/5/24	1193	30
848	0	121148	1	35	2015/2/22	2018/5/31	1194	30
849	0	156361	0	71	2014/2/21	2017/5/31	1195	30
850	0	170239	0	65	2015/2/3	2018/5/15	1197	30
851	0	167975	0	64	2014/11/5	2018/2/18	1201	30
852	0	156840	1	58	2014/1/29	2017/5/17	1204	30
853	0	106248	1	68	2012/2/22	2012/10/13	234	30
854	0	132949	1	57	2012/3/20	2015/7/21	1218	30
855	1	162863	1	38	2014/12/16	2018/4/18	1219	30
856	0	99881	1	43	2009/4/6	2011/6/27	812	30
857	0	169627	1	37	2014/12/17	2018/4/25	1225	30
858	1	108110	1	57	2015/1/16	2018/5/29	1229	30
859	0	111867	1	57	2015/1/8	2018/5/27	1235	30
860	1	145527	0	61	2013/3/26	2016/8/15	1238	30
861	0	160558	1	45	2014/6/6	2017/10/27	1239	30
862	0	135084	1	45	2013/11/4	2017/3/30	1242	30
863	0	168531	1	60	2014/11/21	2018/4/16	1242	30
864	0	170071	1	64	2014/12/29	2018/5/24	1242	30
865	0	**113203**	1	46	2008/12/31	2012/5/28	1244	30
866	0	146475	1	58	2014/9/23	2018/2/28	1254	30
867	0	111937	1	50	2010/3/4	2013/8/10	1255	30
868	0	155355	0	68	2014/9/7	2018/2/13	1255	30
869	0	154922	1	40	2013/12/7	2017/5/26	1266	30
870	0	164307	1	56	2014/11/4	2018/5/5	1278	30
871	0	111396	1	61	2014/10/29	2018/5/8	1287	30
872	1	131662	1	62	2012/4/17	2015/10/29	1290	30
873	0	166814	1	44	2014/10/9	2018/5/3	1302	30

874	0	167036	1	57	2014/10/14	2018/5/9	1303	30
875	0	139771	1	66	2013/5/22	2016/12/26	1314	30
876	0	164741	1	55	2014/8/15	2018/3/21	1314	30
877	0	142036	1	55	2012/12/25	2016/8/9	1323	30
878	0	126026	1	58	2011/8/5	2015/3/24	1327	30
879	0	151745	1	37	2014/8/15	2018/4/3	1327	30
880	0	**98807**	1	50	2013/9/25	2017/5/18	1331	30
881	0	157187	1	51	2014/8/20	2018/5/5	1354	30
882	0	146483	1	63	2014/8/23	2018/5/13	1359	30
883	0	112892	1	65	2011/5/16	2015/2/5	1361	30
884	0	157029	1	56	2014/2/7	2017/11/3	1365	30
885	0	108715	1	63	2009/9/21	2013/6/25	1373	30
886	0	163059	1	39	2014/7/6	2018/4/11	1375	30
887	0	102222	0	67	2012/12/22	2016/9/30	1378	30
888	1	88349	1	60	2010/4/10	2014/1/18	1379	30
889	0	121234	1	45	2011/3/6	2014/12/20	1385	30
890	0	**101163**	1	69	2009/2/9	2012/12/26	1416	30
891	0	**89520**	1	70	2010/5/1	2014/5/13	1473	30
892	0	163249	1	44	2014/7/9	2018/8/9	1492	30
893	0	155071	1	60	2014/7/19	2018/8/22	1495	30
894	0	144408	1	51	2013/2/24	2017/4/30	1526	30
895	1	129011	1	41	2014/6/30	2018/9/10	1533	30
896	0	115853	1	62	2011/11/4	2016/1/16	1534	30
897	0	158646	1	53	2014/3/19	2018/6/1	1535	30
898	1	155457	1	48	2013/12/21	2018/3/7	1537	30
899	1	138096	1	52	2012/8/10	2016/10/28	1540	30
900	0	**91106**	1	46	2014/7/11	2018/9/30	1542	30
901	0	132343	1	29	2014/7/23	2018/10/22	1552	30
902	0	154831	1	59	2014/6/20	2018/10/2	1565	30
903	0	161966	1	44	2014/6/11	2018/9/28	1570	30
904	1	121379	0	62	2014/7/1	2018/10/22	1574	30
905	0	161111	1	45	2014/6/18	2018/10/16	1581	30
906	0	143378	1	32	2013/1/17	2017/5/22	1586	30
907	0	156703	1	54	2014/1/24	2018/5/30	1587	30
908	0	123202	0	52	2011/5/11	2015/9/21	1594	30
909	0	123923	1	48	2011/5/31	2015/10/14	1597	30
910	1	101400	1	55	2010/8/26	2015/1/11	1599	30
911	0	159502	1	52	2014/4/9	2018/9/5	1610	30

912	1	153739	1	49	2014/1/29	2018/7/12	1625	30
913	0	110882	1	58	2010/10/3	2015/3/19	1628	30
914	0	156371	1	43	2014/1/15	2018/7/2	1629	30
915	0	146561	1	50	2013/5/10	2017/11/24	1659	30
916	1	145820	1	42	2013/4/2	2017/10/24	1666	30
917	0	156384	1	58	2014/2/14	2018/9/9	1668	30
918	1	154717	1	64	2014/3/8	2018/10/4	1671	30
919	0	**49212**	1	53	2013/11/7	2018/6/6	1672	30
920	1	**111816**	0	55	2014/3/11	2018/10/20	1684	30
921	0	156328	1	57	2014/1/14	2018/8/30	1689	30
922	1	157817	1	57	2014/2/26	2018/10/16	1693	30
923	0	134294	1	56	2012/5/1	2016/12/24	1698	30
924	1	103243	1	52	2014/2/6	2018/10/4	1701	30
925	0	104327	1	52	2009/4/13	2013/12/30	1722	30
926	0	107021	1	48	2011/2/14	2015/11/9	1729	30
927	1	128794	1	53	2012/11/6	2017/8/3	1731	30
928	1	135789	1	79	2012/6/26	2017/4/1	1740	30
929	0	144997	1	63	2014/1/7	2018/10/15	1742	30
930	0	154702	1	53	2013/12/2	2018/9/29	1762	30
931	0	115725	1	64	2012/5/16	2017/3/22	1771	30
932	0	154704	1	62	2013/12/2	2018/10/10	1773	30
933	0	108662	1	36	2009/9/17	2014/7/29	1776	30
934	0	107162	1	43	2012/3/31	2017/2/24	1791	30
935	0	112944	1	39	2010/4/18	2015/4/8	1816	30
936	0	**97943**	1	39	2011/9/2	2016/8/22	1816	30
937	1	139519	0	48	2012/9/21	2017/9/30	1835	30
938	0	102215	1	53	2009/1/19	2014/2/7	1845	30
939	0	152287	0	52	2013/9/24	2018/10/22	1854	30
940	0	**133000**	1	53	2013/6/17	2018/7/19	1858	30
941	1	118900	1	71	2010/11/26	2016/1/23	1884	30
942	0	**116997**	1	62	2012/4/22	2017/6/23	1888	30
943	0	**99491**	1	59	2011/9/6	2016/11/8	1890	30
944	0	**100322**	1	56	2009/10/4	2014/12/13	1896	30
945	1	110540	1	47	2009/12/19	2015/3/30	1927	30
946	0	130045	1	60	2011/12/13	2017/4/3	1938	30
947	0	102277	1	41	2008/12/5	2014/4/6	1948	30
948	0	109402	0	67	2013/5/3	2018/9/2	1948	30
949	1	147142	1	57	2013/6/11	2018/10/14	1951	30

950	0	117986	1	49	2013/4/21	2018/9/3	1961	30
951	0	102176	1	37	2008/11/28	2014/4/15	1964	30
952	0	112128	1	58	2010/3/15	2015/8/3	1967	30
953	0	147133	1	45	2013/5/10	2018/10/3	1972	30
954	0	146336	1	35	2013/4/17	2018/9/28	1990	30
955	1	109957	1	46	2011/6/24	2017/1/24	2041	30
956	0	145191	1	65	2013/3/18	2018/10/22	2044	30
957	0	138711	1	53	2012/8/28	2018/4/18	2059	30
958	0	107052	1	47	2013/1/17	2018/10/8	2090	30
959	0	124565	1	51	2012/1/14	2017/10/5	2091	30
960	0	129566	0	47	2012/2/26	2017/12/7	2111	30
961	0	140040	1	54	2012/10/10	2018/7/23	2112	30
962	0	115041	1	44	2012/10/14	2018/8/20	2136	30
963	1	142224	0	59	2012/12/13	2018/10/22	2139	30
964	0	103622	1	42	2011/3/16	2017/1/25	2142	30
965	0	122838	1	54	2011/4/29	2017/4/19	2182	30
966	0	124205	1	39	2011/6/9	2017/6/1	2184	30
967	0	132326	0	59	2012/10/14	2018/10/10	2187	30
968	1	132933	1	34	2012/6/27	2018/6/29	2193	30
969	0	112690	0	72	2010/10/4	2016/10/22	2210	30
970	1	105045	1	66	2009/2/23	2015/3/15	2211	30
971	0	134851	1	52	2012/5/16	2018/7/15	2251	30
972	0	132555	1	62	2012/3/8	2018/5/10	2254	30
973	0	136840	1	49	2012/7/7	2018/10/10	2286	30
974	0	130568	1	40	2012/1/1	2018/4/19	2300	30
975	1	132165	1	55	2012/3/23	2018/9/9	2361	30
976	0	111056	1	51	2011/7/9	2017/12/31	2367	30
977	0	131460	1	51	2012/2/5	2018/9/2	2401	30
978	0	**59337**	1	61	2012/1/25	2018/10/19	2459	30
979	0	128736	1	59	2011/11/24	2018/9/17	2489	30
980	1	123253	1	51	2011/10/10	2018/8/6	2492	30
981	1	129523	1	52	2011/11/26	2018/9/22	2492	30
982	0	118606	1	37	2011/6/1	2018/4/2	2497	30
983	1	112927	1	45	2010/4/16	2017/3/14	2524	30
984	1	112235	0	36	2010/3/18	2017/2/17	2528	30
985	0	111201	1	49	2010/4/13	2017/3/16	2529	30
986	0	128849	1	46	2011/11/6	2018/10/17	2537	30
987	0	111992	1	49	2010/3/8	2017/3/6	2555	30

988	1	121518	1	44	2011/3/16	2018/4/3	2575	30
989	0	124667	0	47	2011/6/23	2018/10/13	2669	30
990	0	124446	1	48	2011/6/16	2018/10/13	2676	30
991	0	123823	1	49	2011/5/28	2018/10/19	2701	30
992	0	111834	1	46	2010/2/28	2017/8/23	2733	30
993	1	**92322**	1	47	2010/6/25	2018/1/26	2772	30
994	0	115851	1	58	2010/12/6	2018/9/26	2851	30
995	0	118739	1	54	2010/11/20	2018/9/25	2866	30
996	0	116829	1	55	2010/10/12	2018/8/24	2873	30
997	0	107643	1	42	2009/8/3	2017/7/7	2895	30
998	0	**99144**	1	46	2010/10/18	2018/9/25	2899	30
999	0	110048	1	49	2010/5/30	2018/5/11	2903	30
1000	0	115915	1	42	2010/8/27	2018/10/6	2962	30
1001	0	103113	1	58	2010/8/27	2018/10/10	2966	30
1002	0	110153	0	63	2010/1/15	2018/3/2	2968	30
1003	0	114552	1	47	2010/6/13	2018/9/12	3013	30
1004	0	111142	1	62	2010/1/21	2018/4/22	3013	30
1005	0	110530	1	49	2009/12/18	2018/4/13	3038	30
1006	0	109986	1	46	2009/11/20	2018/4/30	3083	30
1007	0	108970	1	55	2009/10/5	2018/4/25	3124	30
1008	0	108386	0	54	2009/9/4	2018/4/18	3148	30
1009	0	**141007**	1	34	2009/7/15	2018/8/23	3326	30
1010	1	**137313**	1	44	2008/12/19	2018/3/28	3386	30
1011	1	**139092**	1	57	2009/3/1	2018/7/10	3418	30
1012	0	104311	0	50	2009/4/12	2018/9/14	3442	30
1013	0	**142389**	0	52	2009/1/16	2018/9/4	3518	30
1014	0	135892	1	80	2012/9/14	2012/9/17	3	3
1015	0	136454	1	38	2012/10/4	2012/10/7	3	3
1016	0	164771	1	40	2014/8/16	2014/8/19	3	3
1017	0	129327	1	56	2014/11/14	2014/11/17	3	3
1018	0	179261	1	75	2015/9/12	2015/9/15	3	3
1019	1	202287	1	79	2017/4/29	2017/5/2	3	3
1020	0	109977	1	28	2009/11/20	2009/11/23	3	3
1021	1	123257	1	58	2011/5/12	2011/5/16	4	4
1022	0	154911	1	46	2014/5/6	2014/5/10	4	4
1023	0	167418	1	59	2014/12/26	2014/12/30	4	4
1024	1	102974	1	51	2011/3/22	2011/3/27	5	5
1025	1	142292	1	40	2012/12/16	2012/12/21	5	5

1026	0	178464	0	67	2015/8/19	2015/8/24	5	5
1027	0	146079	1	53	2018/2/5	2018/2/10	5	5
1028	0	117655	1	44	2010/10/6	2010/10/12	6	6
1029	1	186542	1	48	2016/4/7	2016/4/13	6	6
1030	0	198571	1	71	2017/2/23	2017/3/1	6	6
1031	0	117894	1	55	2010/10/17	2010/10/23	6	6
1032	0	111113	1	70	2011/4/18	2013/1/8	631	30
1033	0	129634	1	54	2011/11/30	2011/12/7	7	7
1034	1	139497	1	53	2012/9/20	2012/9/27	7	7
1035	0	167539	1	60	2014/12/20	2014/12/27	7	7
1036	0	198351	0	79	2017/1/23	2017/1/30	7	7
1037	1	101611	1	44	2008/10/11	2008/10/19	8	8
1038	1	100430	1	73	2011/7/4	2011/7/12	8	8
1039	0	140849	1	54	2012/11/2	2012/11/11	9	9
1040	0	154549	1	44	2013/11/27	2013/12/6	9	9
1041	0	191001	1	54	2016/7/23	2016/8/1	9	9
1042	1	196202	0	77	2016/12/1	2016/12/10	9	9
1043	0	113583	1	57	2010/5/12	2010/5/22	10	10
1044	0	119851	1	35	2011/1/4	2011/1/14	10	10
1045	0	206145	1	54	2017/8/1	2017/8/11	10	10
1046	0	209427	1	61	2017/10/26	2017/11/5	10	10
1047	0	142593	1	46	2012/12/24	2013/1/4	11	11
1048	1	183906	0	57	2016/1/28	2016/2/8	11	11
1049	0	141031	1	48	2012/11/8	2012/11/20	12	12
1050	0	112113	1	71	2013/1/20	2013/2/1	12	12
1051	0	146559	1	45	2013/4/23	2013/5/5	12	12
1052	0	101941	0	30	2008/11/10	2008/11/22	12	12
1053	0	103893	0	55	2009/3/21	2009/4/3	13	13
1054	0	138392	0	59	2012/8/18	2012/9/1	14	14
1055	1	142756	1	60	2012/12/30	2013/1/13	14	14
1056	0	106312	0	60	2011/6/12	2011/6/27	15	15
1057	0	140705	1	44	2012/10/30	2012/11/15	16	16
1058	0	98310	0	59	2010/2/6	2010/2/24	18	18
1059	0	120374	1	68	2011/1/26	2011/2/13	18	18
1060	0	145409	1	75	2013/3/22	2013/4/9	18	18
1061	1	103020	0	68	2009/1/29	2009/2/18	20	20
1062	0	133205	1	59	2014/2/16	2014/3/8	20	20
1063	0	143259	1	59	2013/1/14	2013/2/4	21	21

1064	0	154879	1	54	2013/12/6	2013/12/30	24	24
1065	0	144584	0	33	2014/2/12	2014/3/8	24	24
1066	0	94099	1	42	2010/1/16	2010/2/10	25	25
1067	0	209967	1	43	2017/11/9	2017/12/4	25	25
1068	1	116046	0	48	2010/7/29	2010/8/23	25	25
1069	0	143001	0	69	2013/2/24	2013/3/22	26	26
1070	1	133338	1	70	2014/4/30	2014/5/26	26	26
1071	0	111188	1	36	2010/1/23	2010/2/19	27	27
1072	0	139765	1	30	2012/9/28	2012/10/25	27	27
1073	0	166830	1	44	2014/10/9	2014/11/5	27	27
1074	0	167606	1	44	2014/12/30	2015/1/27	28	28
1075	1	174955	0	57	2015/5/18	2018/4/2	1050	30
1076	0	129639	1	48	2011/11/30	2015/1/19	1146	30
1077	0		0	48				1
1078	0		0	71				1
1079	0		0	66				1
1080	0		0	77				2
1081	1		0	65				2
1082	0		0	66				2
1083	0		0	64				2
1084	0		1	51				2
1085	0		1	65				30
1086	0		0	78				30
1087	0		0	56				30
1088	0		0	70				30
1089	1		0	51				30
1090	1		1	63				30
1091	0		0	59				30
1092	0		0	73				30
1093	0		0	68				30
1094	0		0	76				30
1095	1		0	60				12
1096	0		0	76				30
1097	0		0	91				30
1098	0		0	58				30
1099	1		0	77				30
1100	0		0	74				30
1101	0		0	78				30

1102	0	0	53	30
1103	0	0	58	30
1104	0	0	54	30
1105	0	1	48	30
1106	0	1	71	12
1107	0	0	49	9
1108	0	0	82	30
1109	0	1	74	16
1110	0	0	61	30
1111	0	1	76	30
1112	0	0	73	30
1113	0	1	47	30
1114	0	0	71	30
1115	0	1	78	12
1116	1	1	61	9
1117	0	0	73	30
1118	0	1	55	30
1119	0	1	58	30
1120	0	1	44	30
1121	0	1	74	13
1122	0	0	83	30
1123	1	1	39	12
1124	0	1	61	30
1125	1	0	73	30
1126	0	1	54	30
1127	0	1	59	30
1128	1	0	87	30
1129	1	0	93	15
1130	0	1	77	20
1131	1	1	24	30
1132	0	1	54	30
1133	0	0	76	30
1134	0	1	52	12
1135	1	1	52	30
1136	0	0	77	30
1137	0	1	63	30
1138	0	0	58	12
1139	1	0	68	5

1140	1	0	51	21
1141	1	0	81	30
1142	1	0	78	28
1143	0	1	52	30
1144	1	1	43	22
1145	0	0	70	1
1146	0	0	65	13
1147	0	0	71	30
1148	0	0	62	30
1149	0	0	58	30
1150	0	0	64	10
1151	1	0	67	8
1152	1	1	69	13
1153	0	0	70	17
1154	1	1	52	30
1155	0	1	52	30
1156	0	1	80	30
1157	0	0	70	30
1158	0	0	53	25
1159	0	0	52	30
1160	0	1	66	30
1161	0	0	60	30
1162	0	0	66	30
1163	0	0	71	13
1164	1	0	71	8
1165	0	0	78	14
1166	1	1	50	30
1167	0	1	54	30
1168	1	1	63	30
1169	0	0	71	30
1170	0	1	61	30
1171	1	0	65	30
1172	1	1	75	30
1173	0	0	71	30
1174	0	0	72	21
1175	1	1	85	30
1176	0	0	64	30
1177	0	0	70	30

1178	0	0	65	30
1179	0	0	78	29
1180	0	1	56	30
1181	1	0	47	30
1182	0	1	61	30
1183	0	0	69	32
1184	0	0	66	32
1185	0	1	53	33
1186	0	0	75	33
1187	0	0	71	34
1188	1	1	58	35
1189	0	0	57	36
1190	1	0	66	36
1191	0	0	60	37
1192	0	1	47	38
1193	0	1	78	39
1194	1	0	76	42
1195	0	1	60	42
1196	0	0	77	44
1197	1	1	70	47
1198	1	0	69	50
1199	0	1	61	50
1200	0	0	76	51
1201	0	0	75	52
1202	1	0	78	55
1203	0	1	50	57
1204	0	0	64	57
1205	0	0	18	60
1206	0	0	65	62
1207	0	1	65	65
1208	0	0	67	68
1209	1	0	69	68
1210	0	0	70	70
1211	0	0	59	70
1212	0	0	73	71
1213	0	0	51	75
1214	0	1	53	76
1215	0	0	53	78

1216	0	0	52	80
1217	0	0	71	83
1218	0	0	69	88
1219	0	1	47	91
1220	0	1	60	92
1221	0	0	57	93
1222	0	1	52	93
1223	0	0	65	94
1224	0	0	67	95
1225	0	0	53	102
1226	0	0	52	104
1227	0	0	78	104
1228	1	0	55	109
1229	0	1	39	111
1230	0	0	58	114
1231	0	1	49	116
1232	0	0	59	127
1233	1	0	51	130
1234	0	1	71	139
1235	0	0	72	140
1236	0	1	45	141
1237	0	0	61	142
1238	0	1	77	150
1239	1	1	46	150
1240	0	0	57	150
1241	0	0	62	156
1242	1	1	76	156
1243	0	1	70	158
1244	0	1	59	162
1245	0	1	47	171
1246	0	0	72	173
1247	1	0	60	197
1248	0	0	72	198
1249	1	0	62	202
1250	0	1	52	204
1251	0	1	78	205
1252	0	1	52	210
1253	1	1	72	214

1254	0	1	63	216
1255	1	0	60	217
1256	0	0	54	218
1257	0	1	62	220
1258	0	0	63	225
1259	0	0	50	228
1260	0	0	59	230
1261	1	0	70	241
1262	0	1	66	248
1263	0	0	70	249
1264	0	0	70	260
1265	0	0	59	268
1266	0	1	29	271
1267	0	0	67	274
1268	0	0	66	281
1269	0	0	74	283
1270	0	0	37	292
1271	0	1	46	293
1272	0	0	62	304
1273	1	1	70	304
1274	1	0	64	304
1275	0	1	30	308
1276	0	1	54	318
1277	0	1	51	320
1278	1	1	46	326
1279	0	1	46	329
1280	0	0	73	330
1281	0	1	53	905
1282	0	0	63	339
1283	0	0	73	343
1284	0	0	77	347
1285	0	0	61	349
1286	0	0	75	355
1287	0	0	57	361
1288	0	1	78	362
1289	0	1	66	374
1290	0	0	61	377
1291	0	1	56	381

1292	1	0	71	390
1293	0	1	52	393
1294	0	1	70	395
1295	0	1	75	398
1296	0	0	58	417
1297	0	0	70	421
1298	1	1	71	430
1299	0	0	66	431
1300	0	0	53	438
1301	0	1	43	453
1302	0	0	74	456
1303	0	0	62	471
1304	0	0	71	496
1305	0	1	46	506
1306	0	0	74	507
1307	0	1	54	507
1308	0	1	60	513
1309	0	0	75	534
1310	0	1	80	540
1311	1	1	52	556
1312	0	0	65	568
1313	1	1	46	574
1314	0	0	60	593
1315	0	0	70	607
1316	0	0	50	624
1317	0	0	69	646
1318	0	1	67	658
1319	0	0	66	659
1320	0	1	49	660
1321	0	1	44	677
1322	0	1	56	685
1323	0	0	79	689
1324	0	0	69	697
1325	1	0	70	706
1326	0	0	71	749
1327	0	1	53	757
1328	0	0	62	793
1329	0	0	77	817

1330	0	0	72	826
1331	0	0	77	827
1332	0	0	62	843
1333	0	0	74	860
1334	0	0	73	865
1335	1	1	71	867
1336	1	1	70	875
1337	0	1	62	883
1338	0	0	64	908
1339	0	0	63	927
1340	0	0	74	940
1341	0	1	78	956
1342	0	0	64	957
1343	1	0	68	659
1344	1	1	61	1030
1345	0	0	60	1033
1346	1	0	61	1033
1347	1	1	57	1038
1348	0	0	66	1044
1349	0	1	32	1053
1350	0	1	61	1096
1351	0	0	69	1124
1352	0	0	68	1161
1353	0	0	58	1178
1354	0	0	64	1187
1355	0	0	74	1229
1356	0	0	61	1238
1357	0	1	68	1254
1358	1	0	68	1255
1359	1	0	70	1283
1360	0	1	53	1302
1361	0	1	56	1327
1362	1	0	62	1330
1363	0	0	56	1336
1364	0	0	74	1337
1365	0	1	55	1392
1366	1	1	70	1473
1367	0	1	68	1499

1368	1	0	61	1578
1369	0	0	54	1584
1370	0	1	37	1652
1371	1	1	42	1666
1372	1	1	41	1767
1373	1	0	73	1977
1374	0	0	52	1986
1375	0	0	59	2139
1376	0	0	69	2148
1377	1	0	72	2210
1378	0	0	59	2245
1379	1	1	52	2296
1380	0	0	65	2298
1381	0	1	49	2469
1382	0	1	59	2489
1383	0	0	68	2560
1384	0	0	72	2651
1385	0	1	75	2869
1386	1	1	49	2903
1387	0	1	43	3076
1388	0	1	75	3
1389	1	1	55	7
1390	0	0	56	7
1391	0	1	60	7
1392	0	0	62	8
1393	0	0	61	8
1394	0	0	64	10
1395	0	0	48	10
1396	0	1	54	10
1397	0	1	45	12
1398	0	0	30	12
1399	0	1	68	18
1400	0	0	53	20
1401	0	0	54	21
1402	0	0	69	23
1403	0	0	69	26
1404	0	0	57	1050

30结局（	90时间	90结局	360时间		360结局	结局（1=	死亡原因	出血(1=y	SBP
1	1	1	1	0.03	1	1	上消化道	1	0
1	1	1	1	0.03	1	1	肾功能衰	0	0
1	1	1	1	0.03	1	1	循环衰竭	1	0
1	1	1	1	0.03	1	1	肝衰竭	0	0
1	1	1	1	0.03	1	1	失血性休	1	0
1	1	1	1	0.03	1	1	上消化道	1	0
1	1	1	1	0.03	1	1	多脏器功	1	0
1	1	1	1	0.03	1	1	失血性休	1	0
1	1	1	1	0.03	1	1	上消化道	1	0
1	1	1	1	0.03	1	1	慢加急肝	1	0
1	1	1	1	0.03	1	1	感染中毒	0	1
1	1	1	1	0.03	1	1	曲张破裂	1	0
1	1	1	1	0.03	1	1	感染中毒	1	1
1	1	1	1	0.03	1	1	曲张破裂	1	0
1	1	1	1	0.03	1	1	消化道出	1	0
1	1	1	1	0.03	1	1	肝性脑病	0	0
1	1	1	1	0.03	1	1	失血性休	1	0
1	1	1	1	0.03	1	1	感染中毒	0	0
1	2	1	2	0.07	1	1	心率失常	0	0
1	2	1	2	0.07	1	1	消化道出	1	0
1	2	1	2	0.07	1	1	多脏器功	1	0
1	2	1	2	0.07	1	1	多脏器功	1	0
1	2	1	2	0.07	1	1	感染中毒	1	0
1	2	1	2	0.07	1	1	感染中毒	0	0
1	2	1	2	0.07	1	1	多脏器功	0	0
1	2	1	2	0.07	1	1	呼吸衰竭	0	1
1	2	1	2	0.07	1	1	多脏器功	0	0
1	2	1	2	0.07	1	1	多脏器功	1	0
1	2	1	2	0.07	1	1	感染中毒	1	0
1	2	1	2	0.07	1	1	失血性休	1	1
1	2	1	2	0.07	1	1	消化道出	0	0
1	2	1	2	0.07	1	1	感染中毒	0	0
1	5	1	5	0.17	1	1	肝衰竭，	0	0
0	12	0	12	0.4	0	0		1	0
0	6	0	6	0.2	0	0		0	0
0	16	0	16	0.53	0	0		0	0
0	25	0	25	0.83	0	0		0	0

1	22	1	15	0.5	1	1		1	0
0	13	0	13	0.43	0	0		1	0
1	16	1	9	0.3	1	1		0	0
0	7	0	7	0.23	0	0		0	0
1	9	1	2	0.07	1	1		0	0
0	3	0	3	0.1	0	0		1	0
1	9	1	2	0.07	1	1		0	1
1	6	1	6	0.2	1	1	多脏器功	1	0
0	22	0	22	0.73	0	0		0	0
1	10	1	3	0.1	1	1		0	0
1	26	1	19	0.63	1	1		0	1
0	23	0	23	0.77	0	0		0	0
0	90	0	170	5.67	0	0		0	1
1	8	1	1	0.03	1	1		0	0
1	12	1	5	0.17	1	1		0	0
0	25	0	25	0.83	0	0		0	0
1	9	1	2	0.07	1	1		0	0
1	19	1	12	0.4	1	1		0	0
0	6	0	6	0.2	0	0		0	0
1	9	1	2	0.07	1	1		0	1
1	10	1	3	0.1	1	1		0	0
0	20	0	20	0.67	0	0		0	0
1	8	1	1	0.03	1	1		1	0
0	5	0	5	0.17	0	0		0	0
1	8	1	1	0.03	1	1		0	0
0	4	0	4	0.13	0	0		0	0
1	7	1	0	0	1	1		1	0
1	13	1	6	0.2	1	1		0	0
1	17	1	10	0.33	1	1		1	0
1	3	1	3	0.1	1	1	多脏器功	1	0
0	20	0	20	0.67	0	0		0	0
0	5	0	5	0.17	0	0		0	0
1	17	1	10	0.33	1	1		1	0
0	24	0	24	0.8	0	0		0	0
0	21	0	21	0.7	0	0		0	1
0	18	0	18	0.6	0	0		0	0
0	20	0	20	0.67	0	0		0	0
1	23	1	16	0.53	1	1		0	1

0	90	0	360	12	0	0		0	0
0	9	0	9	0.3	0	0		0	0
1	13	1	6	0.2	1	1		0	0
0	10	0	10	0.33	0	0		0	0
0	5	0	5	0.17	0	0		0	0
1	10	1	3	0.1	1	1		1	0
0	3	0	3	0.1	0	0		1	0
0	90	0	360	12	0	0		0	0
1	13	1	6	0.2	1	1		0	0
0	16	0	16	0.53	0	0		0	0
0	5	0	5	0.17	0	0		1	0
0	3	0	3	0.1	0	0		0	0
1	9	1	2	0.07	1	1		0	0
0	90	0	360	12	0	0		1	0
1	10	1	3	0.1	1	1		0	0
0	8	0	8	0.27	0	0		0	0
0	14	0	14	0.47	0	0		0	0
1	11	1	4	0.13	1	1		0	0
1	23	1	16	0.53	1	1		0	0
0	3	0	3	0.1	0	0		0	0
0	3	0	3	0.1	0	0		0	0
1	11	1	4	0.13	1	1		1	0
1	11	1	4	0.13	1	1		0	0
0	2	0	2	0.07	0	0		0	0
0	8	0	8	0.27	0	0		1	0
0	3	0	3	0.1	0	0		1	0
1	9	1	2	0.07	1	1		1	1
0	25	0	25	0.83	0	0		1	1
1	12	1	5	0.17	1	1		1	0
0	5	0	5	0.17	0	0		1	0
0	7	0	7	0.23	0	0		0	0
0	9	0	9	0.3	0	0		0	0
0	10	0	10	0.33	0	0		1	0
1	12	1	5	0.17	1	1		0	0
0	17	0	17	0.57	0	0		0	1
1	12	1	5	0.17	1	1		0	0
1	9	1	2	0.07	1	1		0	0
1	29	1	22	0.73	1	1		1	1

0	6	0	6	0.2	0	0		0	0
0	9	0	9	0.3	0	0		0	0
0	6	0	6	0.2	0	0		0	0
0	6	0	6	0.2	0	0		0	0
1	16	1	9	0.3	1	1		1	0
0	15	0	15	0.5	0	0		0	0
1	16	1	9	0.3	1	1		0	0
1	11	1	4	0.13	1	1		1	0
1	26	1	19	0.63	1	1		0	0
0	26	0	26	0.87	0	0		0	0
0	7	0	7	0.23	0	0		0	0
1	9	1	2	0.07	1	1		1	0
1	14	1	7	0.23	1	1		0	0
1	12	1	5	0.17	1	1		1	0
1	10	1	3	0.1	1	1		0	0
1	12	1	5	0.17	1	1		1	0
0	9	0	9	0.3	0	0		0	0
0	6	0	6	0.2	0	0		0	0
0	3	0	3	0.1	0	0		0	0
0	6	0	6	0.2	0	0		0	0
0	19	0	19	0.63	0	0		0	0
0	90	0	360	12	0	0		0	0
1	18	1	11	0.37	1	1		1	0
1	19	1	12	0.4	1	1		0	0
0	14	0	14	0.47	0	0		0	0
0	21	0	21	0.7	0	0		0	0
0	6	0	6	0.2	0	0		0	0
0	6	0	6	0.2	0	0		0	0
0	22	0	22	0.73	0	0		0	0
1	13	1	6	0.2	1	1		0	0
1	8	1	8	0.27	1	1	失血性休	1	0
1	11	1	4	0.13	1	1		0	0
1	15	1	15	0.5	1	1	感染中毒	0	1
1	12	1	5	0.17	1	1		1	1
1	9	1	2	0.07	1	1		0	0
1	7	1	0	0	1	1		0	0
1	13	1	6	0.2	1	1		1	0
0	6	0	6	0.2	0	0		0	0

0	13	0	13	0.43	0	0		0	0
1	12	1	5	0.17	1	1		0	0
0	22	0	22	0.73	0	0		1	0
1	13	1	6	0.2	1	1		0	0
1	11	1	4	0.13	1	1		0	0
0	4	0	4	0.13	0	0		0	0
0	5	0	5	0.17	0	0		1	0
0	7	0	7	0.23	0	0		0	0
0	22	0	22	0.73	0	0		0	0
0	4	0	4	0.13	0	0		1	0
0	18	0	18	0.6	0	0		0	0
0	6	0	6	0.2	0	0		1	0
0	13	0	13	0.43	0	0		0	0
1	15	1	8	0.27	1	1		1	1
1	11	1	4	0.13	1	1		0	0
1	13	1	6	0.2	1	1		0	0
0	21	0	21	0.7	0	0		0	0
0	18	0	18	0.6	0	0		0	0
0	9	0	9	0.3	0	0		0	0
1	13	1	6	0.2	1	1		0	0
0	10	0	10	0.33	0	0		0	0
1	15	1	8	0.27	1	1		0	0
1	11	1	4	0.13	1	1		1	0
0	16	0	16	0.53	0	0		0	0
1	20	1	13	0.43	1	1		0	0
0	6	0	6	0.2	0	0		1	0
1	13	1	6	0.2	1	1		1	0
1	11	1	4	0.13	1	1		0	0
0	9	0	9	0.3	0	0		1	0
0	16	0	16	0.53	0	0		0	1
0	33	0	26	0.87	0	0		1	0
0	24	0	24	0.8	0	0		0	0
1	8	1	8	0.27	1	1	多脏器功	0	0
0	12	0	12	0.4	0	0		1	0
0	11	0	11	0.37	0	0		0	0
0	33	0	26	0.87	0	0		0	0
0	5	0	5	0.17	0	0		1	0
0	20	0	20	0.67	0	0		0	0

0	27	0	27	0.9	0	0		0	0
1	3	1	3	0.1	1	1	肝功能衰	0	0
0	19	0	19	0.63	0	0		1	0
1	2	1	2	0.07	1	1	肝性脑病	0	0
0	20	0	20	0.67	0	0		0	0
0	7	0	7	0.23	0	0		1	0
0	15	0	15	0.5	0	0		0	0
0	17	0	17	0.57	0	0		0	0
0	2	0	2	0.07	0	0		1	0
0	90	0	360	12	0	0		0	0
0	7	0	7	0.23	0	0		0	0
1	25	1	18	0.6	1	1		0	0
1	7	1	7	0.23	1	1	曲张破裂	1	0
1	10	1	3	0.1	1	1		0	0
0	4	0	4	0.13	0	0		0	0
1	12	1	5	0.17	1	1		0	0
1	5	1	5	0.17	1	1	感染性休	0	0
1	1	1	1	0.03	1	1	感染中毒	0	1
0	19	0	19	0.63	0	0		0	0
0	21	0	21	0.7	0	0		0	0
0	5	0	5	0.17	0	0		0	0
0	14	0	14	0.47	0	0		0	0
0	8	0	8	0.27	0	0		1	0
1	10	1	3	0.1	1	1		1	0
1	28	1	21	0.7	1	1		0	0
1	18	1	11	0.37	1	1		1	0
0	11	0	11	0.37	0	0		0	0
0	8	0	8	0.27	0	0		0	0
0	27	0	27	0.9	0	0		0	0
0	6	0	6	0.2	0	0		0	1
0	7	0	7	0.23	0	0		1	0
1	8	1	1	0.03	1	1		0	0
1	17	1	10	0.33	1	1		1	0
0	7	0	7	0.23	0	0		0	0
0	24	0	24	0.8	0	0		0	0
1	8	1	1	0.03	1	1		1	0
1	8	1	1	0.03	1	1		1	0
0	5	0	5	0.17	0	0		1	0

1	11	1	4	0.13	1	1		0	0
1	10	1	3	0.1	1	1		1	0
1	10	1	3	0.1	1	1		0	0
1	23	1	16	0.53	1	1		1	0
0	8	0	8	0.27	0	0		0	0
1	29	1	22	0.73	1	1		0	0
1	24	1	17	0.57	1	1		0	0
1	12	1	5	0.17	1	1		0	0
1	9	1	2	0.07	1	1		1	0
1	8	1	1	0.03	1	1		0	0
1	11	1	4	0.13	1	1		0	0
1	13	1	6	0.2	1	1		0	0
1	15	1	15	0.5	1	1	肝性脑病	0	0
0	19	0	19	0.63	0	0		0	0
0	90	0	360	12	0	0		0	0
0	10	0	10	0.33	0	0		0	0
1	25	1	18	0.6	1	1		0	0
0	15	0	15	0.5	0	0		0	1
0	3	0	3	0.1	0	0		0	0
0	23	0	23	0.77	0	0		0	0
0	26	0	26	0.87	0	0		0	0
0	90	0	360	12	0	0		0	0
1	11	1	11	0.37	1	1	感染中毒	1	0
0	13	0	13	0.43	0	0		1	0
0	7	0	7	0.23	0	0		1	0
0	2	0	2	0.07	0	0		0	0
0	2	0	2	0.07	0	0		0	0
0	7	0	7	0.23	0	0		0	0
1	8	1	1	0.03	1	1		0	0
0	90	0	360	12	0	0		0	0
0	12	0	12	0.4	0	0		0	0
0	14	0	14	0.47	0	0		1	0
1	12	1	5	0.17	1	1		1	0
0	11	0	11	0.37	0	0		0	0
0	12	0	12	0.4	0	0		1	0
1	11	1	4	0.13	1	1		1	0
1	15	1	8	0.27	1	1		0	0
0	10	0	10	0.33	0	0		1	0

0	4	0	4	0.13	0	0		1	0
0	32	0	25	0.83	0	0		0	0
1	15	1	8	0.27	1	1		0	0
0	7	0	7	0.23	0	0		0	0
1	9	1	2	0.07	1	1		0	0
1	8	1	1	0.03	1	1		1	0
0	7	0	7	0.23	0	0		0	0
1	13	1	6	0.2	1	1		1	0
1	11	1	4	0.13	1	1		0	0
0	2	0	2	0.07	0	0		0	0
1	10	1	3	0.1	1	1		1	0
1	4	1	4	0.13	1	1	感染中毒	0	0
1	14	1	7	0.23	1	1		1	1
1	12	1	5	0.17	1	1		0	0
0	7	0	7	0.23	0	0		0	0
1	12	1	5	0.17	1	1		0	0
0	4	0	4	0.13	0	0		0	0
0	4	0	4	0.13	0	0		0	0
0	1	0	1	0.03	0	0		0	0
0	5	0	5	0.17	0	0		1	0
1	24	1	17	0.57	1	1		1	0
0	14	0	14	0.47	0	0		1	0
0	90	0	360	12	0	0		1	0
0	13	0	13	0.43	0	0		1	0
0	19	0	19	0.63	0	0		1	0
0	5	0	5	0.17	0	0		0	0
0	17	0	17	0.57	0	0		0	0
1	13	1	6	0.2	1	1		0	0
0	8	0	8	0.27	0	0		0	0
0	31	0	24	0.8	0	0		0	0
0	10	0	10	0.33	0	0		0	0
0	16	0	16	0.53	0	0		0	0
0	16	0	16	0.53	0	0		0	0
0	7	0	7	0.23	0	0		0	0
1	21	1	14	0.47	1	1		0	0
0	13	0	13	0.43	0	0		0	0
0	18	0	18	0.6	0	0		0	0
0	28	0	28	0.93	0	0		0	1

1	28	1	28	0.93	1	1	多脏器功	1	1
1	28	1	28	0.93	1	1	慢性肝衰	1	0
0	29	0	29	0.97	0	0		0	0
1	29	1	29	0.97	1	1	多脏器功	0	1
0	29	0	29	0.97	0	0		0	1
0	29	0	29	0.97	0	0		1	0
0	29	0	29	0.97	0	0		1	0
0	30	0	30	1	0	0		0	0
0	30	0	30	1	0	0		0	0
0	30	0	30	1	0	0		0	0
0	30	1	30	1	1	1	多脏器功	1	0
0	31	0	31	1.03	0	0		1	0
0	31	0	31	1.03	0	0		0	0
0	31	1	31	1.03	1	1	肾衰竭	0	1
0	31	1	31	1.03	1	1	感染中毒	0	0
0	32	1	32	1.07	1	1	多脏器功	1	0
0	32	0	32	1.07	0	0		0	0
0	32	0	32	1.07	0	0		0	0
0	32	0	32	1.07	0	0		1	0
0	33	0	33	1.1	0	0		0	0
0	33	0	33	1.1	0	0		0	0
0	34	0	34	1.13	0	0		0	0
0	35	0	35	1.17	0	0		0	0
0	35	0	35	1.17	0	0		0	0
0	35	0	35	1.17	0	0		0	0
0	36	1	36	1.2	1	1	多脏器功	0	0
0	36	0	36	1.2	0	0		0	0
0	36	0	36	1.2	0	0		0	0
0	36	0	36	1.2	0	0		0	0
0	36	0	36	1.2	0	0		0	0
0	36	1	36	1.2	1	1	心功能不	0	0
0	36	1	36	1.2	1	1	感染中毒	1	0
0	37	0	37	1.23	0	0		0	0
0	37	0	37	1.23	0	0		0	0
0	37	0	37	1.23	0	0		0	0
0	38	0	38	1.27	0	0		0	0
0	38	0	38	1.27	0	0		0	1
0	39	0	39	1.3	0	0		0	0

0	39	0	39	1.3	0	0		1	0
0	39	0	39	1.3	0	0		0	0
0	39	0	39	1.3	0	0		0	0
0	39	0	39	1.3	0	0		0	0
0	40	0	40	1.33	0	0		0	0
0	41	0	41	1.37	0	0		0	1
0	41	0	41	1.37	0	0		0	0
0	41	0	41	1.37	0	0		0	1
0	41	0	41	1.37	0	0		1	1
0	41	1	41	1.37	1	1	失血性休	1	0
0	41	0	41	1.37	0	0		1	0
0	42	0	42	1.4	0	0		0	1
0	42	0	42	1.4	0	0		0	0
0	42	0	42	1.4	0	0		1	0
0	43	0	43	1.43	0	0		0	0
0	43	1	43	1.43	1	1	上消化道	1	0
0	43	0	43	1.43	0	0		1	0
0	43	0	43	1.43	0	0		0	1
0	43	1	43	1.43	1	1		1	0
0	43	0	43	1.43	0	0		0	0
0	43	0	43	1.43	0	0		1	0
0	44	1	44	1.47	1	1		0	0
0	44	0	44	1.47	0	0		0	0
0	44	1	44	1.47	1	1	慢加急肝	1	1
0	45	0	45	1.5	0	0		0	0
0	45	0	45	1.5	0	0		1	1
0	45	1	45	1.5	1	1		0	0
0	45	1	45	1.5	1	1		1	0
0	45	0	45	1.5	0	0		0	0
0	46	1	46	1.53	1	1	低血容量	1	1
0	47	0	47	1.57	0	0		0	0
0	47	0	47	1.57	0	0		1	1
0	48	1	48	1.6	1	1	多脏器功	1	1
0	48	0	48	1.6	0	0		0	0
0	48	0	48	1.6	0	0		0	0
0	49	1	49	1.63	1	1		1	0
0	50	1	50	1.67	1	1	多脏器功	1	0
0	50	0	50	1.67	0	0		0	0

0	50	0	50	1.67	0	0		0	0
0	50	0	50	1.67	0	0		1	0
0	50	0	50	1.67	0	0		0	0
0	50	0	50	1.67	0	0		0	0
0	56	1	56	1.87	1	1	脑出血	0	0
0	51	0	51	1.7	0	0		0	0
0	51	1	51	1.7	1	1	慢加急肝	1	0
0	52	0	52	1.73	0	0		1	0
0	53	0	53	1.77	0	0		0	0
0	54	0	54	1.8	0	0		0	0
0	54	0	54	1.8	0	0		0	0
0	55	0	55	1.83	0	0		1	1
0	55	0	55	1.83	0	0		0	0
0	56	0	56	1.87	0	0		1	1
0	56	0	56	1.87	0	0		0	0
0	57	0	57	1.9	0	0		0	0
0	57	0	57	1.9	0	0		0	0
0	57	0	57	1.9	0	0		0	0
0	58	1	58	1.93	1	1	循环衰竭	0	0
0	58	1	58	1.93	1	1	上消化道	1	1
0	58	0	58	1.93	0	0		0	1
0	59	0	59	1.97	0	0		0	0
0	59	0	59	1.97	0	0		0	0
0	60	0	60	2	0	0		0	1
0	61	0	61	2.03	0	0		0	0
0	61	0	61	2.03	0	0		0	0
0	62	0	62	2.07	0	0		0	0
0	62	1	62	2.07	1	1		1	0
0	62	0	62	2.07	0	0		0	0
0	62	0	62	2.07	0	0		1	0
0	63	0	63	2.1	0	0		0	0
0	64	0	64	2.13	0	0		0	0
0	64	0	64	2.13	0	0		1	0
0	64	0	64	2.13	0	0		1	0
0	64	0	64	2.13	0	0		1	0
0	65	0	65	2.17	0	0		0	1
0	67	0	67	2.23	0	0		0	0
0	67	0	67	2.23	0	0		0	0

0	68	0	68	2.27	0	0		0	0
0	68	0	68	2.27	0	0		0	0
0	68	0	68	2.27	0	0		0	0
0	69	0	69	2.3	0	0		0	0
0	70	1	70	2.33	1	1		0	0
0	70	0	70	2.33	0	0		0	0
0	70	0	70	2.33	0	0		0	0
0	70	0	70	2.33	0	0		0	0
0	71	0	71	2.37	0	0		0	0
0	71	1	71	2.37	1	1	感染中毒	0	0
0	71	0	71	2.37	0	0		0	0
0	73	0	73	2.43	0	0		0	0
0	74	0	74	2.47	0	0		0	0
0	75	1	75	2.5	1	1	肝昏迷	0	0
0	76	0	76	2.53	0	0		1	0
0	77	0	77	2.57	0	0		0	0
0	78	0	78	2.6	0	0		0	0
0	79	0	79	2.63	0	0		0	0
0	80	0	80	2.67	0	0		0	1
0	80	1	80	2.67	1	1	多脏器功	0	0
0	81	1	81	2.7	1	1	多脏器功	0	0
0	81	0	81	2.7	0	0		0	0
0	82	1	82	2.73	1	1	多脏器功	0	0
0	83	0	83	2.77	0	0		0	1
0	86	0	86	2.87	0	0		0	0
0	87	0	87	2.9	0	0		0	0
0	87	0	87	2.9	0	0		1	0
0	88	1	88	2.93	1	1	多脏器功	0	0
0	88	1	88	2.93	1	1	多脏器功	0	0
0	89	0	89	2.97	0	0		0	0
0	90	0	90	3	0	0		0	1
0	90	0	90	3	0	0		0	0
0	90	0	91	3.03	1	1	多脏器功	0	0
0	90	0	91	3.03	0	0		0	0
0	90	0	95	3.17	0	0		0	0
0	90	0	96	3.2	1	1	肝性脑病	0	0
0	90	0	99	3.3	0	0		1	0
0	90	0	100	3.33	1	1	肝性脑病	1	0

0	90	0	103	3.43	1	1	消化道出	1	0
0	90	0	104	3.47	0	0		0	0
0	90	0	104	3.47	1	1	肝性脑病	0	1
0	90	0	107	3.57	1	1	多脏器功	0	0
0	90	0	107	3.57	0	0		0	0
0	90	0	107	3.57	0	0		0	0
0	90	0	109	3.63	0	0		0	0
0	90	0	111	3.7	0	0		0	0
0	90	0	111	3.7	0	0		0	0
0	90	0	111	3.7	0	0		0	0
0	90	0	116	3.87	0	0		0	0
0	90	0	117	3.9	0	0		1	0
0	90	0	117	3.9	0	0		1	0
0	90	0	121	4.03	0	0		1	0
0	90	0	122	4.07	0	0		0	0
0	90	0	126	4.2	1	1	感染中毒	0	0
0	90	0	126	4.2	0	0		1	0
0	90	0	127	4.23	0	0		0	0
0	90	0	129	4.3	0	0		0	0
0	90	0	130	4.33	0	0		1	0
0	90	0	130	4.33	0	0		0	0
0	90	0	130	4.33	0	0		0	0
0	90	0	135	4.5	0	0		0	0
0	90	0	135	4.5	0	0		0	0
0	90	0	135	4.5	0	0		0	0
0	90	0	137	4.57	0	0		0	0
0	90	0	139	4.63	0	0		0	0
0	90	0	139	4.63	0	0		0	0
0	90	0	140	4.67	0	0		0	0
0	90	0	140	4.67	0	0		1	0
0	90	0	142	4.73	1	1	肝性脑病	0	0
0	90	0	143	4.77	0	0		1	0
0	90	0	149	4.97	0	0		0	0
0	90	0	150	5	0	0		0	0
0	90	0	150	5	0	0		0	0
0	90	0	152	5.07	0	0		0	0
0	90	0	152	5.07	0	0		0	0
0	90	0	153	5.1	1	1	多脏器功	0	0

0	90	0	153	5.1	0	0		1	0
0	90	0	153	5.1	0	0		1	0
0	90	0	155	5.17	1	1	心源性猝	0	0
0	90	0	156	5.2	1	1	肝性脑病	0	0
0	90	0	156	5.2	1	1	肝肾综合	0	0
0	90	0	157	5.23	0	0		1	0
0	90	0	157	5.23	0	0		1	0
0	90	0	157	5.23	1	1	失血性休	0	0
0	90	0	160	5.33	1	1		1	1
0	90	0	162	5.4	0	0		0	1
0	90	0	163	5.43	0	0		0	1
0	90	0	167	5.57	0	0		1	0
0	90	0	168	5.6	0	0		0	0
0	90	0	171	5.7	0	0		0	0
0	90	0	171	5.7	0	0		0	0
0	90	0	173	5.77	0	0		0	0
0	90	0	176	5.87	0	0		1	0
0	90	0	177	5.9	1	1	慢加急肝	0	0
0	90	0	180	6	0	0		0	0
0	90	0	183	6.1	0	0		0	0
0	90	0	183	6.1	0	0		0	0
0	90	0	186	6.2	0	0		0	0
0	90	0	196	6.53	0	0		0	0
0	90	0	198	6.6	0	0		0	0
0	90	0	201	6.7	0	0		0	0
0	90	0	204	6.8	0	0		0	0
0	90	0	205	6.83	0	0		0	0
0	90	0	206	6.87	0	0		1	0
0	90	0	210	7	0	0		0	0
0	90	0	210	7	0	0		1	0
0	90	0	212	7.07	0	0		0	0
0	90	0	213	7.1	0	0		0	0
0	90	0	214	7.13	1	1	感染中毒	0	0
0	90	0	214	7.13	0	0		1	0
0	90	0	217	7.23	0	0		1	0
0	90	0	217	7.23	0	0		1	0
0	90	0	218	7.27	0	0		0	0
0	90	0	218	7.27	0	0		0	0

0	90	0	219	7.3	0	0		0	0
0	90	0	219	7.3	1	1	消化道出	1	0
0	90	0	220	7.33	0	0		0	0
0	90	0	220	7.33	0	0		1	0
0	90	0	222	7.4	0	0		1	0
0	90	0	225	7.5	0	0		0	0
0	90	0	225	7.5	0	0		0	0
0	90	0	225	7.5	0	0		0	0
0	90	0	226	7.53	1	1	多脏器功	0	0
0	90	0	226	7.53	0	0		1	0
0	90	0	227	7.57	0	0		0	0
0	90	0	227	7.57	0	0		0	0
0	22	0	22	0.73	0	0		1	0
0	90	0	228	7.6	0	0		0	0
0	90	0	229	7.63	0	0		0	0
0	90	0	229	7.63	0	0		1	0
0	90	0	231	7.7	0	0		0	0
0	90	0	237	7.9	0	0		1	0
0	90	0	241	8.03	0	0		1	0
0	90	0	241	8.03	0	0		1	0
0	90	0	243	8.1	0	0		0	0
0	90	0	243	8.1	0	0		0	0
0	90	0	244	8.13	0	0		0	0
0	90	0	246	8.2	0	0		0	0
0	90	0	247	8.23	0	0		0	0
0	90	0	248	8.27	0	0		0	0
0	90	0	251	8.37	1	1	上消化道	0	0
0	90	0	253	8.43	0	0		0	0
0	90	0	253	8.43	0	0		1	0
0	90	0	256	8.53	0	0		0	0
0	90	0	258	8.6	0	0		0	0
0	90	0	260	8.67	0	0		0	0
0	90	0	264	8.8	0	0		1	0
0	90	0	267	8.9	0	0		0	0
0	90	0	268	8.93	0	0		0	0
0	90	0	269	8.97	0	0		0	0
0	90	0	269	8.97	0	0		0	0
0	90	0	271	9.03	1	1	慢性肝衰	1	0

0	90	0	277	9.23	0	0		1	0
0	90	0	277	9.23	0	0		1	0
0	90	0	279	9.3	0	0		0	0
0	90	0	280	9.33	0	0		0	0
0	90	0	282	9.4	0	0		1	0
0	90	0	283	9.43	1	1	感染中毒	0	0
0	90	0	286	9.53	0	0		0	0
0	90	0	290	9.67	0	0		0	0
0	90	0	291	9.7	0	0		1	0
0	90	0	292	9.73	0	0		0	0
0	90	0	293	9.77	0	0		0	0
0	90	0	295	9.83	0	0		0	0
0	90	0	296	9.87	0	0		0	0
0	90	0	296	9.87	0	0		0	0
0	90	0	304	10.13	0	0		1	0
0	90	0	305	10.17	0	0		1	0
0	90	0	306	10.2	0	0		0	0
0	90	0	308	10.27	1	1	呼吸心跳	0	0
0	90	0	308	10.27	0	0		1	0
0	90	0	308	10.27	0	0		1	0
0	90	0	309	10.3	0	0		1	0
0	90	0	310	10.33	0	0		0	0
0	90	0	311	10.37	1	1	失血性休	1	0
0	90	0	312	10.4	0	0		1	1
0	90	0	316	10.53	0	0		0	0
0	90	0	317	10.57	1	1		0	0
0	90	0	322	10.73	0	0		0	0
0	90	0	322	10.73	0	0		1	0
0	90	0	327	10.9	0	0		0	0
0	90	0	329	10.97	0	0		1	0
0	90	0	329	10.97	0	0		0	0
0	90	0	330	11	0	0		0	0
0	90	0	334	11.13	0	0		0	0
0	90	0	335	11.17	0	0		0	0
0	90	0	335	11.17	0	0		0	0
0	90	0	336	11.2	0	0		0	0
0	90	0	340	11.33	0	0		1	0
0	90	0	343	11.43	1	1	消化道出	0	0

0	90	0	343	11.43	1	1	肝性脑病	0	0
0	90	0	347	11.57	1	1		0	0
0	90	0	347	11.57	0	0		1	0
0	90	0	349	11.63	1	1		0	0
0	90	0	351	11.7	0	0		1	0
0	90	0	352	11.73	1	1	失血性休	1	0
0	90	0	357	11.9	0	0		1	0
0	90	0	357	11.9	0	0		0	0
0	90	0	357	11.9	0	0		0	0
0	90	0	357	11.9	0	0		0	0
0	90	0	359	11.97	0	0		0	0
0	90	0	360	12	0	0		0	0
0	90	0	360	12	0	0		1	0
0	90	0	360	12	0	1	猝死	1	0
0	90	0	360	12	0	0		0	0
0	90	0	360	12	0	0		1	0
0	90	0	360	12	0	1		0	1
0	90	0	360	12	0	0		0	0
0	90	0	360	12	0	0		0	0
0	90	0	360	12	0	0		1	0
0	90	0	360	12	0	0		0	0
0	90	0	360	12	0	0		0	0
0	90	0	360	12	0	1	循环衰竭	0	0
0	90	0	360	12	0	1		0	0
0	90	0	360	12	0	0		1	0
0	90	0	360	12	0	0		0	0
0	90	0	360	12	0	0		0	0
0	90	0	360	12	0	0		0	0
0	90	0	360	12	0	1		0	0
0	90	0	360	12	0	0		0	0
0	90	0	360	12	0	0		0	0
0	90	0	360	12	0	0		0	0
0	90	0	360	12	0	0		0	0
0	90	0	360	12	0	0		0	0
0	90	0	360	12	0	1		0	0
0	90	0	360	12	0	1	上消化道	1	0
0	90	0	360	12	0	0		0	0
0	90	0	360	12	0	1	感染中毒	0	0

0	90	0	360	12	0	0		1	0
0	90	0	360	12	0	0		0	0
0	90	0	360	12	0	0		1	0
0	90	0	360	12	0	0		0	0
0	90	0	360	12	0	0		0	0
0	90	0	360	12	0	0		1	0
0	90	0	360	12	0	0		0	0
0	90	0	360	12	0	0		0	0
0	90	0	360	12	0	0		0	0
0	90	0	360	12	0	0		1	0
0	90	0	360	12	0	0		0	0
0	90	0	360	12	0	0		1	0
0	90	0	360	12	0	0		1	0
0	90	0	360	12	0	1	慢性肝功	0	0
0	90	0	360	12	0	1	感染中毒	1	0
0	90	0	360	12	0	0		1	0
0	90	0	360	12	0	0		0	0
0	90	0	360	12	0	1	曲张破裂	1	0
0	90	0	360	12	0	0		1	0
0	90	0	360	12	0	1	肝衰竭	0	0
0	90	0	360	12	0	0		0	0
0	90	0	360	12	0	0		0	0
0	90	0	360	12	0	0		0	0
0	90	0	230	7.67	1	1	上消化道	0	0
0	90	0	360	12	0	0		0	0
0	90	0	360	12	0	1	肺动脉高	1	0
0	90	0	360	12	0	0		1	0
0	90	0	360	12	0	0		1	0
0	90	0	360	12	0	0		0	0
0	90	0	360	12	0	0		0	0
0	90	0	360	12	0	0		0	0
0	90	0	360	12	0	0		0	0
0	90	0	360	12	0	0		0	0
0	90	0	360	12	0	1	急诊，死	1	0
0	90	0	360	12	0	0		1	0
0	90	0	360	12	0	0		1	0
0	90	0	360	12	0	0		1	0
0	90	0	360	12	0	1	失血性休	0	0

0	90	0	360	12	0	1	失血性休	0	0
0	90	0	360	12	0	0		1	0
0	90	0	360	12	0	1	呼吸衰竭	0	0
0	90	0	360	12	0	0		1	0
0	90	0	360	12	0	1	肝性脑病	0	0
0	90	0	360	12	0	0		1	0
0	90	0	360	12	0	0		0	1
0	90	0	360	12	0	0		0	0
0	90	0	360	12	0	1	慢性心功	1	0
0	90	0	360	12	0	0		1	0
0	90	0	360	12	0	0		0	0
0	90	0	360	12	0	0		1	0
0	90	0	360	12	0	0		0	0
0	90	0	360	12	0	0		0	0
0	90	0	360	12	0	1	感染中毒	1	0
0	90	0	360	12	0	0		1	0
0	90	0	360	12	0	0		1	1
0	90	0	360	12	0	0		0	0
0	90	0	360	12	0	0		0	0
0	90	0	360	12	0	0		1	0
0	90	0	360	12	0	1	肝性脑病	0	0
0	90	0	360	12	0	0		1	0
0	90	0	360	12	0	0		1	0
0	90	0	360	12	0	1		1	0
0	90	0	360	12	0	0		0	0
0	90	0	360	12	0	0		1	0
0	90	0	360	12	0	0		1	0
0	90	0	360	12	0	0		1	0
0	90	0	360	12	0	0		0	0
0	90	0	360	12	0	0		0	0
0	90	0	360	12	0	0		1	0
0	90	0	360	12	0	0		0	0
0	90	0	360	12	0	0		0	0
0	90	0	360	12	0	0		1	0
0	90	0	360	12	0	0		0	0
0	90	0	360	12	0	0		1	0
0	90	0	360	12	0	0		1	1
0	90	0	360	12	0	0		1	0

0	90	0	360	12	0	0		0	0
0	90	0	360	12	0	0		0	0
0	90	0	360	12	0	0		1	0
0	90	0	360	12	0	0		0	0
0	90	0	360	12	0	0		1	0
0	90	0	360	12	0	0		1	0
0	90	0	360	12	0	0		1	0
0	90	0	360	12	0	0		0	0
0	90	0	360	12	0	0		0	0
0	90	0	360	12	0	0		1	0
0	90	0	360	12	0	1	慢性肝衰	0	0
0	90	0	360	12	0	0		0	0
0	90	0	360	12	0	0		0	0
0	90	0	360	12	0	1	多脏器功	0	0
0	90	0	360	12	0	0		0	0
0	90	0	360	12	0	0		0	0
0	90	0	360	12	0	0		0	0
0	90	0	360	12	0	0		0	0
0	90	0	360	12	0	0		1	0
0	90	0	360	12	0	1	慢性肝衰	1	0
0	90	0	360	12	0	0		1	0
0	90	0	360	12	0	0		0	0
0	90	0	360	12	0	0		0	0
0	90	0	360	12	0	0		0	0
0	90	0	360	12	0	1	多脏器功	0	0
0	90	0	360	12	0	0		1	0
0	90	0	360	12	0	0		1	0
0	90	0	360	12	0	0		0	0
0	90	0	360	12	0	0		0	0
0	90	0	360	12	0	0		0	0
0	90	0	360	12	0	0		0	1
0	90	0	360	12	0	0		0	0
0	90	0	360	12	0	0		1	0
0	90	0	360	12	0	0		1	0
0	90	0	360	12	0	0		0	0
0	90	0	360	12	0	0		0	0
0	90	0	360	12	0	0		0	0
0	90	0	360	12	0	0		1	0

0	90	0	360	12	0	0		1	0
0	90	0	360	12	0	0		0	0
0	90	0	360	12	0	0		1	0
0	90	0	360	12	0	0		0	0
0	90	0	360	12	0	0		1	0
0	90	0	360	12	0	0		0	0
0	90	0	360	12	0	1		0	0
0	90	0	360	12	0	1	HE	0	0
0	90	0	360	12	0	0		0	0
0	90	0	360	12	0	0		0	0
0	90	0	360	12	0	0		0	0
0	90	0	360	12	0	0		1	0
0	90	0	360	12	0	0		0	0
0	90	0	360	12	0	0		0	0
0	90	0	360	12	0	0		0	0
0	90	0	360	12	0	0		0	0
0	90	0	360	12	0	1	肝性脑病	0	0
0	90	0	360	12	0	0		1	0
0	90	0	360	12	0	0		0	0
0	90	0	360	12	0	1	HE	1	0
0	90	0	360	12	0	0		1	0
0	90	0	360	12	0	1	慢加急肝	1	0
0	90	0	360	12	0	0		0	0
0	90	0	360	12	0	0		0	0
0	90	0	360	12	0	0		1	0
0	90	0	360	12	0	1		1	0
0	90	0	360	12	0	0		0	0
0	90	0	360	12	0	0		0	0
0	90	0	360	12	0	0		1	0
0	90	0	360	12	0	0		1	0
0	90	0	360	12	0	0		1	0
0	90	0	360	12	0	0		0	0
0	90	0	360	12	0	0		1	0
0	90	0	360	12	0	0		0	0
0	90	0	360	12	0	0		0	0
0	90	0	360	12	0	0		0	0
0	90	0	360	12	0	0		0	0
0	90	0	360	12	0	0		0	0

0	90	0	360	12	0	0		0	0
0	90	0	360	12	0	1	肝性脑病	1	0
0	90	0	360	12	0	0		1	0
0	90	0	360	12	0	0		1	0
0	90	0	360	12	0	1	上消化道	0	0
0	90	0	360	12	0	1	肝衰竭	1	0
0	90	0	360	12	0	0		0	0
0	90	0	360	12	0	0		0	0
0	90	0	360	12	0	0		0	0
0	90	0	360	12	0	0		1	1
0	90	0	360	12	0	0		0	0
0	90	0	360	12	0	0		0	0
0	90	0	360	12	0	0		0	0
0	90	0	360	12	0	1	肝肾综合	0	0
0	90	0	360	12	0	0		1	0
0	90	0	360	12	0	0		0	0
0	90	0	360	12	0	0		0	0
0	90	0	360	12	0	0		0	0
0	90	0	360	12	0	0		0	0
0	90	0	360	12	0	0		0	0
0	90	0	360	12	0	0		0	0
0	90	0	360	12	0	0		0	0
0	90	0	360	12	0	1	慢加急肝	0	0
0	90	0	360	12	0	1	感染中毒	0	1
0	90	0	360	12	0	0		0	0
0	90	0	360	12	0	0		0	0
0	90	0	360	12	0	0		1	0
0	90	0	360	12	0	0		1	0
0	90	0	360	12	0	0		0	0
0	90	0	360	12	0	0		0	0
0	90	0	360	12	0	0		1	0
0	90	0	360	12	0	0		0	0
0	90	0	360	12	0	0		0	0
0	90	0	360	12	0	1		0	0
0	90	0	360	12	0	0		0	0
0	90	0	360	12	0	0		1	0
0	90	0	360	12	0	0		0	0
0	90	0	360	12	0	0		0	0

0	90	0	360	12	0	0		1	0
0	90	0	360	12	0	0		0	0
0	90	0	360	12	0	0		1	0
0	90	0	360	12	0	0		1	0
0	90	0	360	12	0	0		1	0
0	90	0	360	12	0	0		0	0
0	90	0	360	12	0	0		1	0
0	90	0	360	12	0	0		1	0
0	90	0	360	12	0	0		1	0
0	90	0	360	12	0	1	多脏器功	0	0
0	90	0	360	12	0	0		0	0
0	90	0	360	12	0	0		1	0
0	90	0	360	12	0	0		0	0
0	90	0	360	12	0	0		0	0
0	90	0	360	12	0	0		0	0
0	90	0	360	12	0	0		0	0
0	90	0	360	12	0	0		1	0
0	90	0	234	7.8	1	1	上消化道	1	0
0	90	0	360	12	0	0		0	0
0	90	0	360	12	0	0		1	0
0	90	0	360	12	0	1	肝肾综合	0	0
0	90	0	360	12	0	0		1	0
0	90	0	360	12	0	0		0	0
0	90	0	360	12	0	0		0	0
0	90	0	360	12	0	0		0	0
0	90	0	360	12	0	0		0	0
0	90	0	360	12	0	0		0	0
0	90	0	360	12	0	0		1	0
0	90	0	360	12	0	0		1	0
0	90	0	360	12	0	0		0	0
0	90	0	360	12	0	0		0	0
0	90	0	360	12	0	0		0	0
0	90	0	360	12	0	0		1	0
0	90	0	360	12	0	0		1	0
0	90	0	360	12	0	0		0	0
0	90	0	360	12	0	0		1	0
0	90	0	360	12	0	1	曲张破裂	1	0
0	90	0	360	12	0	0		0	0

0	90	0	360	12	0	0		1	0
0	90	0	360	12	0	0		1	0
0	90	0	360	12	0	0		0	0
0	90	0	360	12	0	0		0	0
0	90	0	360	12	0	1		1	0
0	90	0	360	12	0	0		0	0
0	90	0	360	12	0	0		0	0
0	90	0	360	12	0	0		0	0
0	90	0	360	12	0	0		0	0
0	90	0	360	12	0	0		0	0
0	90	0	360	12	0	0		0	0
0	90	0	360	12	0	0		0	0
0	90	0	360	12	0	0		0	0
0	90	0	360	12	0	0		1	0
0	90	0	360	12	0	1		1	0
0	90	0	360	12	0	0		0	0
0	90	0	360	12	0	0		0	0
0	90	0	360	12	0	0		1	0
0	90	0	360	12	0	0		1	0
0	90	0	360	12	0	0		1	0
0	90	0	360	12	0	0		0	0
0	90	0	360	12	0	0		0	0
0	90	0	360	12	0	0		0	0
0	90	0	360	12	0	0		0	0
0	90	0	360	12	0	0		0	0
0	90	0	360	12	0	0		0	0
0	90	0	360	12	0	0		0	0
0	90	0	360	12	0	0		1	0
0	90	0	360	12	0	0		1	0
0	90	0	360	12	0	0		0	0
0	90	0	360	12	0	0		1	0
0	90	0	360	12	0	0		1	0
0	90	0	360	12	0	0		0	0
0	90	0	360	12	0	0		0	0
0	90	0	360	12	0	0		0	0
0	90	0	360	12	0	0		0	0
0	90	0	360	12	0	1		0	0
0	90	0	360	12	0	0		0	0

0	90	0	360	12	0	0		1	0
0	90	0	360	12	0	1		0	0
0	90	0	360	12	0	0		0	0
0	90	0	360	12	0	0		0	0
0	90	0	360	12	0	0		1	0
0	90	0	360	12	0	0		1	0
0	90	0	360	12	0	0		1	0
0	90	0	360	12	0	0		1	0
0	90	0	360	12	0	0		0	0
0	90	0	360	12	0	0		0	0
0	90	0	360	12	0	0		1	0
0	90	0	360	12	0	0		0	0
0	90	0	360	12	0	0		1	0
0	90	0	360	12	0	0		0	0
0	90	0	360	12	0	0		1	0
0	90	0	360	12	0	0		1	0
0	90	0	360	12	0	0		0	0
0	90	0	360	12	0	0		1	0
0	90	0	360	12	0	0		0	0
0	90	0	360	12	0	0		1	0
0	90	0	360	12	0	0		0	0
0	90	0	360	12	0	1	急性重症	0	0
0	90	0	360	12	0	0		1	0
0	90	0	360	12	0	0		1	0
0	90	0	360	12	0	0		0	0
0	90	0	360	12	0	0		0	0
0	90	0	360	12	0	0		0	0
0	90	0	360	12	0	0		0	0
0	90	0	360	12	0	0		1	0
0	90	0	360	12	0	1		0	0
0	90	0	360	12	0	0		0	0
0	90	0	360	12	0	0		0	0
0	90	0	360	12	0	0		0	0
0	90	0	360	12	0	0		1	0
0	90	0	360	12	0	0		0	0
0	90	0	360	12	0	1	失血性休	0	0
0	90	0	360	12	0	0		0	0
0	90	0	360	12	0	0		0	0

0	90	0	360	12	0	0		1	0
0	90	0	360	12	0	0		0	0
0	90	0	360	12	0	0		0	0
0	90	0	360	12	0	0		1	0
0	90	0	360	12	0	0		1	0
0	90	0	360	12	0	0		1	0
0	90	0	360	12	0	0		1	0
0	90	0	360	12	0	0		0	0
0	90	0	360	12	0	0		0	0
0	90	0	360	12	0	0		1	0
0	90	0	360	12	0	0		1	0
0	90	0	360	12	0	0		0	0
0	90	0	360	12	0	0		1	0
0	90	0	360	12	0	0		1	0
0	90	0	360	12	0	0		1	0
0	90	0	360	12	0	0		0	0
0	90	0	360	12	0	0		0	0
0	90	0	360	12	0	0		1	0
0	90	0	360	12	0	0		0	0
0	90	0	360	12	0	1		1	0
0	90	0	360	12	0	1	肝性脑病	0	0
0	90	0	360	12	0	0		0	0
0	90	0	360	12	0	0		0	0
0	90	0	360	12	0	0		0	0
0	90	0	360	12	0	0		0	0
0	90	0	360	12	0	0		0	0
0	90	0	360	12	0	0		0	0
0	90	0	360	12	0	0		1	0
0	90	0	360	12	0	0		1	0
0	90	0	360	12	0	0		0	0
0	90	0	360	12	0	0		0	0
0	90	0	360	12	0	0		0	0
0	90	0	360	12	0	1		0	0
0	90	0	360	12	0	1		0	0
0	90	0	360	12	0	0		0	0
0	90	0	360	12	0	0		0	0
0	90	0	360	12	0	0		0	0
0	90	0	360	12	0	0		0	0

0	90	0	360	12	0	0		0	0
0	90	0	360	12	0	0		1	0
0	90	0	360	12	0	0		1	0
0	90	0	360	12	0	0		1	1
0	90	0	360	12	0	0		1	0
0	90	0	360	12	0	0		1	0
0	90	0	360	12	0	0		1	0
0	90	0	360	12	0	0		1	0
0	90	0	360	12	0	0		1	0
0	90	0	360	12	0	0		1	0
0	90	0	360	12	0	0		1	0
0	90	0	360	12	0	0		1	0
0	90	0	360	12	0	0		0	0
0	90	0	360	12	0	0		1	0
0	90	0	360	12	0	0		1	0
0	90	0	360	12	0	0		0	0
0	90	0	360	12	0	0		1	0
0	90	0	360	12	0	0		1	0
0	90	0	360	12	0	0		0	0
0	90	0	360	12	0	0		0	1
0	90	0	360	12	0	0		1	0
0	90	0	360	12	0	0		1	0
0	90	0	360	12	0	0		0	0
0	90	0	360	12	0	0		0	0
0	90	0	360	12	0	0		1	0
0	90	0	360	12	0	0		0	0
1	3	1	3	0.1	1	1	多脏器功	0	0
1	3	1	3	0.1	1	1	多脏器功	1	0
1	3	1	3	0.1	1	1	失血性休	1	0
1	3	1	3	0.1	1	1	失血性休	1	0
1	3	1	3	0.1	1	1	多脏器功	1	0
1	3	1	3	0.1	1	1	感染中毒	0	0
1	3	1	3	0.1	1	1	急性肝衰	0	0
1	4	1	4	0.13	1	1		1	0
1	4	1	4	0.13	1	1	多脏器功	0	0
1	4	1	4	0.13	1	1	多脏器功	1	0
1	5	1	5	0.17	1	1		1	0
1	5	1	5	0.17	1	1	多脏器功	0	0

1	5	1	5	0.17	1	1	慢加急肝	1	0
1	5	1	5	0.17	1	1	感染中毒	0	0
1	6	1	6	0.2	1	1		1	1
1	6	1	6	0.2	1	1	慢加急肝	1	0
1	6	1	6	0.2	1	1	多脏器功	0	0
1	6	1	6	0.2	1	1	多脏器功	0	0
0	90	0	360	12	0	1		0	1
1	7	1	7	0.23	1	1		1	0
1	7	1	7	0.23	1	1	感染中毒	0	0
1	7	1	7	0.23	1	1	失血性休	1	0
1	7	1	7	0.23	1	1	慢加急肝	0	0
1	8	1	8	0.27	1	1	上消化道	1	0
1	8	1	8	0.27	1	1		1	0
1	9	1	9	0.3	1	1	多脏器功	1	0
1	9	1	9	0.3	1	1	消化道出	1	0
1	9	1	9	0.3	1	1	呼吸衰竭	0	0
1	9	1	9	0.3	1	1	慢加急肝	0	0
1	10	1	10	0.33	1	1		0	0
1	10	1	10	0.33	1	1		0	0
1	10	1	10	0.33	1	1	慢加急肝	1	1
1	10	1	10	0.33	1	1	曲张破裂	1	1
1	11	1	11	0.37	1	1	多脏器功	1	0
1	11	1	11	0.37	1	1	肝性脑病	0	0
1	12	1	12	0.4	1	1	多脏器功	0	0
1	12	1	12	0.4	1	1	曲张破裂	1	0
1	12	1	12	0.4	1	1	肝性脑病	0	0
1	12	1	12	0.4	1	1	急性肝衰	0	0
1	13	1	13	0.43	1	1	多脏器功	0	0
1	14	1	14	0.47	1	1	肝性脑病	1	0
1	14	1	14	0.47	1	1	上消化道	0	0
1	15	1	15	0.5	1	1		1	0
1	16	1	16	0.53	1	1	上消化道	1	0
1	18	1	18	0.6	1	1		0	1
1	18	1	18	0.6	1	1		1	1
1	18	1	18	0.6	1	1	多脏器功	0	0
1	20	1	20	0.67	1	1	肝衰竭、	0	0
1	20	1	20	0.67	1	1	消化道出	1	0
1	21	1	21	0.7	1	1	多脏器功	1	1

1	24	1	24	0.8	1	1	多脏器功	0	0
1	24	1	24	0.8	1	1	多脏器功	1	0
1	25	1	25	0.83	1	1		0	0
1	25	1	25	0.83	1	1	慢加急肝	0	1
1	25	1	25	0.83	1	1	呼吸衰竭	0	0
1	26	1	26	0.87	1	1	多脏器功	1	0
1	26	1	26	0.87	1	1	肝性脑病	0	1
1	27	1	27	0.9	1	1		1	0
1	27	1	27	0.9	1	1	多脏器功	0	0
1	27	1	27	0.9	1	1	多脏器功	0	0
1	28	1	28	0.93	1	1	慢性肝衰	1	0
0	90	0	360	12	0	0		1	0
0	90	0	360	12	0	0		1	0
1	1	1	0	0.03	1			1	
1	1	1	0	0.03	1			0	
1	1	1	0	0.03	1			1	
1	2	1	0	0.07	1			0	
1	2	1	0	0.07	1			1	
1	2	1	0	0.07	1			0	
1	2	1	0	0.07	1			1	
1	2	1	0	0.07	1			1	
0	24	0	0	0.8	0			0	
0	3	0	0	0.1	0			0	
0	18	0	0	0.6	0			0	
0	21	0	0	0.7	0			1	
0	22	0	0	0.73	0			0	
0	20	0	0	0.67	0			0	
0	15	0	0	0.5	0			0	
0	5	0	0	0.17	0			0	
0	17	0	0	0.57	0			0	
0	21	0	0	0.7	0			0	
1	12	1	0	0.17	1			0	
0	9	0	0	0.3	0			0	
0	3	0	0	0.1	0			0	
0	24	0	0	0.8	0			0	
0	10	0	0	0.33	0			0	
0	17	0	0	0.57	0			0	
0	16	0	0	0.53	0			0	

1	30	1	0	0.77	1	0
0	16	0	0	0.53	0	0
0	18	0	0	0.6	0	1
0	13	0	0	0.43	0	0
1	12	1	0	0.17	1	0
1	9	1	0	0.07	1	0
0	15	0	0	0.5	0	0
1	16	1	0	0.3	1	0
0	7	0	0	0.23	0	0
0	24	0	0	0.8	0	0
0	23	0	0	0.77	0	0
0	5	0	0	0.17	0	1
0	6	0	0	0.2	0	0
1	12	1	0	0.17	1	1
1	9	1	0	0.07	1	1
0	8	0	0	0.27	0	1
0	9	0	0	0.3	0	0
0	16	0	0	0.53	0	1
0	16	0	0	0.53	0	0
1	13	1	0	0.2	1	1
0	13	0	0	0.43	0	0
1	12	1	0	0.17	1	0
0	23	0	0	0.77	0	0
0	16	0	0	0.53	0	0
0	4	0	0	0.13	0	1
0	6	0	0	0.2	0	1
0	21	0	0	0.7	0	0
1	15	1	0	0.27	1	0
1	20	1	0	0.43	1	0
0	9	0	0	0.3	0	1
0	5	0	0	0.17	0	1
0	2	0	0	0.07	0	1
1	12	1	0	0.17	1	1
0	6	0	0	0.2	0	0
0	4	0	0	0.13	0	0
0	17	0	0	0.57	0	0
1	12	1	0	0.17	1	0
1	5	1	0	0.17	1	0

1	21	1	0	0.7	1	1
0	14	0	0	0.47	0	0
1	28	1	0	0.7	1	0
0	13	0	0	0.43	0	0
1	22	1	0	0.5	1	1
1	1	1	0	0.03	1	0
1	13	1	0	0.2	1	1
0	11	0	0	0.37	0	0
0	10	0	0	0.33	0	0
0	3	0	0	0.1	0	0
1	10	1	0	0.1	1	0
1	8	1	0	0.03	1	0
1	13	1	0	0.2	1	0
1	17	1	0	0.33	1	0
0	10	0	0	0.33	0	0
0	23	0	0	0.77	0	0
0	13	0	0	0.43	0	1
0	15	0	0	0.5	0	0
1	25	1	0	0.6	1	1
0	23	0	0	0.77	0	0
0	12	0	0	0.4	0	1
0	6	0	0	0.2	0	0
0	10	0	0	0.33	0	1
1	13	1	0	0.43	1	0
1	8	1	0	0.03	1	1
1	14	1	0	0.23	1	0
0	7	0	0	0.23	0	0
0	5	0	0	0.17	0	0
0	4	0	0	0.13	0	1
0	4	0	0	0.13	0	0
0	1	0	0	0.03	0	0
0	14	0	0	0.47	0	1
0	3	0	0	0.1	0	1
0	9	0	0	0.3	0	0
1	21	1	0	0.47	1	0
0	16	0	0	0.53	0	0
0	28	0	0	0.93	0	0
0	28	0	0	0.93	0	0

0	28	0	0	0.93	0	0
1	29	1	0	0.97	1	0
0	29	0	0	0.97	0	1
0	30	0	0	1	0	1
0	30	0	0	1	0	0
0	32	1	0	1.07	1	0
0	32	0	0	1.07	0	0
0	33	0	0	1.1	0	0
0	33	0	0	1.1	0	1
0	34	1	0	1.13	1	0
0	35	0	0	1.17	0	0
0	36	1	0	1.2	1	0
0	36	1	0	1.2	1	0
0	37	1	0	1.23	1	0
0	38	0	0	1.27	0	0
0	39	1	0	1.3	1	1
0	42	1	0	1.4	1	0
0	42	0	0	1.4	0	0
0	44	1	0	1.47	1	0
0	47	0	0	1.57	0	0
0	50	1	0	1.67	1	1
0	50	0	0	1.67	0	0
0	51	1	0	1.7	1	0
0	52	1	0	1.73	1	1
0	55	0	0	1.83	0	1
0	57	0	0	1.9	0	0
0	57	0	0	1.9	0	0
0	60	0	0	2	0	0
0	62	0	0	2.07	0	1
0	65	0	0	2.17	0	1
0	68	0	0	2.27	0	0
0	68	1	0	2.27	1	0
0	70	0	0	2.33	0	0
0	70	0	0	2.33	0	0
0	71	0	0	2.37	0	0
0	75	1	0	2.5	1	0
0	76	0	0	2.53	0	1
0	78	0	0	2.6	0	0

0	80	1	0	2.67	1	0
0	83	0	0	2.77	0	0
0	88	1	0	2.93	1	0
0	91	0	0	3.03	1	0
0	92	0	0	3.07	0	1
0	93	0	0	3.1	0	1
0	93	0	0	3.1	0	1
0	94	0	0	3.13	0	1
0	95	0	0	3.17	0	0
0	102	0	0	3.4	0	0
0	104	0	0	3.47	0	0
0	104	0	0	3.47	1	0
0	109	0	0	3.63	1	0
0	111	0	0	3.7	0	0
0	114	0	0	3.8	0	0
0	116	0	0	3.87	0	0
0	127	0	0	4.23	0	0
0	130	0	0	4.33	0	0
0	139	0	0	4.63	0	0
0	140	0	0	4.67	0	1
0	141	0	0	4.7	0	0
0	142	0	0	4.73	1	0
0	150	0	0	5	0	0
0	150	0	0	5	0	0
0	150	0	0	5	0	0
0	156	0	0	5.2	1	0
0	156	0	0	5.2	1	0
0	158	0	0	5.27	0	0
0	162	0	0	5.4	0	0
0	171	0	0	5.7	0	0
0	173	0	0	5.77	0	0
0	197	0	0	6.57	0	1
0	198	0	0	6.6	1	0
0	202	0	0	6.73	0	0
0	204	0	0	6.8	0	0
0	205	0	0	6.83	0	0
0	210	0	0	7	0	1
0	214	0	0	7.13	1	0

0	216	0	0	7.2	0	1
0	217	0	0	7.23	0	1
0	218	0	0	7.27	0	0
0	220	0	0	7.33	0	1
0	225	0	0	7.5	0	0
0	228	0	0	7.6	0	0
0	230	0	0	7.67	0	0
0	241	0	0	8.03	0	1
0	248	0	0	8.27	0	0
0	249	0	0	8.3	1	1
0	260	0	0	8.67	0	0
0	268	0	0	8.93	0	1
0	271	0	0	9.03	1	1
0	274	0	0	9.13	0	0
0	281	0	0	9.37	0	0
0	283	0	0	9.43	1	0
0	292	0	0	9.73	0	0
0	293	0	0	9.77	0	0
0	304	0	0	10.13	1	1
0	304	0	0	10.13	0	1
0	304	0	0	10.13	1	0
0	308	0	0	10.27	1	0
0	318	0	0	10.6	0	1
0	320	0	0	10.67	0	1
0	326	0	0	10.87	0	1
0	329	0	0	10.97	0	0
0	330	0	0	11	0	0
0	905	0	360	12	0	0
0	339	0	0	11.3	1	0
0	343	0	0	11.43	0	0
0	347	0	0	11.57	0	1
0	349	0	0	11.63	1	0
0	355	0	0	11.83	0	1
0	361	0	360	12	0	0
0	362	0	360	12	0	1
0	374	0	360	12	0	0
0	377	0	360	12	0	0
0	381	0	360	12	0	1

0	390	0	360	12	0	0
0	393	0	360	12	0	0
0	395	0	360	12	0	0
0	398	0	360	12	0	0
0	417	0	360	12	0	0
0	421	0	360	12	0	0
0	430	0	360	12	0	1
0	431	0	360	12	0	1
0	438	0	360	12	0	0
0	453	0	360	12	0	0
0	456	0	360	12	0	1
0	471	0	360	12	0	1
0	496	0	360	12	0	0
0	506	0	360	12	0	0
0	507	0	360	12	0	1
0	507	0	360	12	0	1
0	513	0	360	12	0	0
0	534	0	360	12	0	1
0	540	0	360	12	0	0
0	556	0	360	12	0	0
0	568	0	360	12	0	1
0	574	0	360	12	0	0
0	593	0	360	12	0	0
0	607	0	360	12	0	0
0	624	0	360	12	0	1
0	646	0	360	12	0	0
0	658	0	360	12	0	1
0	659	0	360	12	0	0
0	660	0	360	12	0	0
0	677	0	360	12	0	0
0	685	0	360	12	0	1
0	689	0	360	12	0	0
0	697	0	360	12	0	0
0	706	0	360	12	0	1
0	749	0	360	12	0	1
0	757	0	360	12	0	0
0	793	0	360	12	0	0
0	817	0	360	12	0	0

0	826	0	360	12	0	0
0	827	0	360	12	0	1
0	843	0	360	12	0	0
0	860	0	360	12	0	0
0	865	0	360	12	0	0
0	867	0	360	12	0	1
0	875	0	360	12	0	0
0	883	0	360	12	0	0
0	908	0	360	12	0	0
0	927	0	360	12	0	0
0	940	0	360	12	0	0
0	956	0	360	12	0	1
0	957	0	360	12	0	0
0	659	0	360	12	0	0
0	1030	0	360	12	0	0
0	1033	0	360	12	0	0
0	1033	0	360	12	0	0
0	1038	0	360	12	0	0
0	1044	0	360	12	0	0
0	1053	0	360	12	0	1
0	1096	0	360	12	0	1
0	1124	0	360	12	0	0
0	1161	0	360	12	0	0
0	1178	0	360	12	0	1
0	1187	0	360	12	0	0
0	1229	0	360	12	0	0
0	1238	0	360	12	0	0
0	1254	0	360	12	0	1
0	1255	0	360	12	0	1
0	1283	0	360	12	0	0
0	1302	0	360	12	0	0
0	1327	0	360	12	0	1
0	1330	0	360	12	0	0
0	1336	0	360	12	0	0
0	1337	0	360	12	0	0
0	1392	0	360	12	0	0
0	1473	0	360	12	0	1
0	1499	0	360	12	0	0

0	1578	0	360	12	0	1
0	1584	0	360	12	0	0
0	1652	0	360	12	0	1
0	1666	0	360	12	0	1
0	1767	0	360	12	0	0
0	1977	0	360	12	0	0
0	1986	0	360	12	0	0
0	2139	0	360	12	0	1
0	2148	0	360	12	0	0
0	2210	0	360	12	0	1
0	2245	0	360	12	0	0
0	2296	0	360	12	0	1
0	2298	0	360	12	0	0
0	2469	0	360	12	0	0
0	2489	0	360	12	0	0
0	2560	0	360	12	0	1
0	2651	0	360	12	0	0
0	2869	0	360	12	0	0
0	2903	0	360	12	0	1
0	3076	0	360	12	0	0
1	3	1	0	0.1	1	1
1	7	1	0	0.23	1	1
1	7	1	0	0.23	1	0
1	7	1	0	0.23	1	1
1	8	1	0	0.27	1	1
1	8	1	0	0.27	1	1
1	10	1	0	0.33	1	1
1	10	1	0	0.33	1	0
1	10	1	0	0.33	1	1
1	12	1	0	0.4	1	0
1	12	1	0	0.4	1	0
1	18	1	0	0.6	1	1
1	20	1	0	0.67	1	1
1	21	1	0	0.7	1	1
1	23	1	0	0.77	1	0
1	26	1	0	0.87	1	1
0	1050	0	360	12	0	1

腹水	**3腹水**	高血压(1	糖尿病(1	HE	ALT	AST	TBIL(um	**1TBIL(m**	DBIL(un
1	2	1	0	3	53.7	151.5	75.5	4.42	41.3
1	2	0	0	2	29.1	56.1	43	2.51	19.5
0	0	0	0	3	32.5	35.6	20.6	1.20	12.9
0	0	0	0	4	652.8	1548.5	53.3	3.12	37.7
1	2	0	0	4	10.8	23.3	8.1	0.47	4.7
1	2	0	0	3	12.7	24.1	85.4	4.99	44.3
1	2	0	0	4	35	80.9	230.4	13.47	162.1
1	2	0	1	3	110.1	151.3	18.1	1.06	12.1
1	0	1	0	3	14.5	27	8.3	0.49	3.7
1	1	0	1	3	62.9	244.2	74.2	4.34	65
1	2	0	1	3	67.9	208.1	265.6	15.53	201.4
1	1	0	0	3	425.1	280.3	328.5	19.21	233.6
1	1	0	0	2	21.6	99.5	177.4	10.37	156.6
0	0	0	1	3	332.3	704.4	29.3	1.71	15
1	3	0	0	4	29.2	63.3	336.5	19.68	184.4
1	2	0	0	4	41.4	116.1	137.4	8.04	78.5
0	0	1	1	2	31.4	21	10.5	0.61	4.5
1	1	0	0	2	52.7	55.3	373.2	21.82	276.6
1	2	0	0	3	60.4	137.9	466.4	27.27	321.9
1	2	0	0	3	271.4	401	184	10.76	105.3
1	3	0	0	4	22.6	37.6	50.5	2.95	34.5
0	0	1	1	2	1491.9	1554.6	84.1	4.92	69.3
1	2	0	1	4	67.8	172.1	318.4	18.62	241.8
0	0	1	1	3	51.6	66.7	17	0.99	11.4
1	2	1	0	4	64.5	115.9	154.8	9.05	130
1	2	1	1	2	19.8	39	10.9	0.64	8.2
1	1	1	1	2	121	309.8	515.9	30.17	389.4
1	2	0	0	2	66.6	41.7	272.5	15.94	190.4
0	0	0	1	4	21.2	36.4	158.1	9.25	122.1
1	2	0	1	2	18.1	42.6	235.1	13.75	150.4
1	2	1	1	2	68.5	273.1	129.1	7.55	84
0	0	0	1	3	159.1	620.4	303.7	17.76	242.6
1	1	0	0	3	686.5	752.7	498.9	29.18	277.8
1	1	0	0	2	126.4	176.9	202.7	11.85	124.8
0	0	0	0	4	17.6	24.5	22.5	1.32	7.8
1	2	0	0	2	48.6	118.6	35.2	2.06	23
0	0	0	0	2	148.4	105.7	344.6	20.15	218.2

1	2	0	0	4	321.6	628.2	469.4	27.45	274
0	0	0	0	3	161.2	106.5	83.9	4.91	51.1
1	1	0	0	2	206.6	253.2	447.5	26.17	234.6
1	2	0	0	3	20.9	22.3	26.4	1.54	10.4
1	2	0	0	2	1157.3	2429.7	56	3.27	38.1
1	1	0	0	2	31.3	117.1	95.2	5.57	63.3
1	2	0	0	3	85.2	128.7	530.6	31.03	342
1	1	0	1	2	44.3	82.6	505.1	29.54	299.7
1	1	1	0	2	52.2	103.1	433.5	25.35	347.4
0	0	0	0	4	54	64.2	47.1	2.75	22.6
1	2	0	0	3	14.4	40.1	554	32.40	288.9
1	1	1	0	3	39.5	45.8	52.9	3.09	27.8
1	3	0	0	2	27.2	51.2	141.2	8.26	58.8
1	1	0	0	4	350.6	693.9	541.8	31.68	300.8
1	1	0	0	2	99.9	143.1	460.8	26.95	292.2
1	2	0	0	3	30.3	58.1	133.4	7.80	107.3
1	2	1	0	2	129.3	384.8	368.7	21.56	227.3
1	2	0	0	4	43.9	90	515.8	30.16	282.7
1	2	0	1	4	77.1	101.5	53.6	3.13	30.5
1	2	0	0	4	64.3	161.9	409.7	23.96	287.3
1	2	0	0	3	73.2	60.7	170.4	9.96	90.3
1	2	0	1	2	75.1	109.8	39.3	2.30	19.4
1	2	0	0	2	31.2	176.7	433.4	25.35	282.1
0	0	0	0	3	21.6	66.8	75.3	4.40	33.2
1	2	0	0	2	98.2	161.2	189.7	11.09	79.5
1	1	0	0	2	19.6	31.1	30.9	1.81	15.3
1	2	0	0	4	76.9	231.6	557.2	32.58	349.2
1	2	0	1	3	272.3	255.8	955.4	55.87	439.6
1	2	0	0	2	30.1	45.1	389.2	22.76	198.4
0	0	0	0	4	41.8	36.9	38.2	2.23	26.2
1	2	0	0	2	26.2	62.4	30.6	1.79	8.3
1	2	0	0	2	206.3	381.8	557.7	32.61	334.9
1	3	0	0	2	27.7	124.5	262.7	15.36	216.4
0	0	1	0	2	472	339.2	549.6	32.14	280.4
1	3	0	0	3	28.2	22.1	44	2.57	27.1
1	3	0	0	2	9.9	13.1	8	0.47	4.9
0	0	0	0	3	23.5	25.4	61.1	3.57	23.7
1	2	0	0	2	443.8	658	405.8	23.73	307

0	0	1	0	2	16.6	22.9	35.6	2.08	15.7
0	0	1	1	3	19.2	19.1	35.5	2.08	14
1	1	0	1	4	152.3	234.7	286.7	16.77	193.1
1	1	0	0	2	17.7	23.4	29.1	1.70	15.4
1	2	0	0	2	24.8	44.7	26.2	1.53	19.5
1	3	0	1	2	215.6	192.1	43.1	2.52	28.5
1	2	0	0	2	17.9	61.2	40.7	2.38	22.7
0	0	0	1	2	47.4	52.1	17.7	1.04	6.8
1	1	0	0	2	209.7	369.8	602.3	35.22	390.8
1	2	0	0	2	11.3	25.2	74	4.33	51
1	0	0	0	3	15.8	21.9	26	1.52	13.4
0	0	0	0	2	44.5	63.1	87.3	5.11	25.1
1	2	0	0	3	134.7	117	663	38.77	499.2
1	0	0	0	4	16.6	16.7	31.7	1.85	17.4
1	2	1	0	2	76.8	125.3	172.2	10.07	122.2
0	0	0	0	3	41.9	61.3	36.1	2.11	25.1
1	1	0	0	2	911.2	738	257.1	15.04	161.7
1	1	1	0	2	282.9	176.1	576.6	33.72	347.1
1	3	0	0	2	135.2	147.6	735.8	43.03	511.6
1	2	0	0	2	26.3	37.3	70	4.09	47.1
1	2	0	0	4	48	44.7	21	1.23	9
1	2	0	0	2	1793.9	1121.7	475.5	27.81	294.7
1	2	1	0	2	58.1	185.6	622.5	36.40	386.5
1	2	0	0	4	26.4	87.8	124.1	7.26	77.8
1	2	0	0	4	20.7	53.4	56.4	3.30	35.3
1	0	0	0	2	18.7	91.3	46.1	2.70	32.5
1	2	0	0	3	101.1	121.7	102.5	5.99	61.3
1	3	0	0	2	26.1	30.8	56.8	3.32	21.5
0	0	0	0	3	138.1	299.4	253.8	14.84	181
1	2	0	1	4	27	31.2	24.7	1.44	11.8
0	0	0	0	2	31.5	41.5	33.5	1.96	10.7
1	0	0	0	2	26.3	53.7	130.2	7.61	61.5
1	0	0	1	2	16.5	23.5	51	2.98	37.4
1	2	0	0	2	2310.3	1482.4	338.5	19.80	252.7
1	3	1	1	2	10.5	34.1	103.8	6.07	83.2
0	0	0	1	2	52.8	98.8	145.9	8.53	80.3
1	2	0	0	3	25.5	80.5	827.3	48.38	578.9
1	2	1	0	2	629.2	630	258.5	15.12	187.6

1	1	0	0	4	39.5	77.3	53.2	3.11	34.1
1	3	0	0	2	41.7	84.7	58.9	3.44	43.4
1	2	0	0	4	35.3	75.9	80.2	4.69	37.6
1	2	0	0	2	47.1	130.6	16.2	0.95	7.8
1	2	0	1	2	379.1	179.4	400.6	23.43	269.4
1	2	1	0	2	11.6	24.7	43.7	2.56	21.6
1	2	0	1	2	151.8	259.5	490	28.65	340.4
1	3	0	0	2	32.4	57.2	650.8	38.06	412.9
0	0	0	0	2	817.5	373.1	113.7	6.65	55.3
1	2	0	1	2	71.6	54.7	95.4	5.58	51.9
0	0	0	1	2	24.3	21.8	42.7	2.50	15.5
1	1	1	0	4	37.2	58.1	8.7	0.51	3.9
1	1	0	1	2	24.4	141.3	19.3	1.13	11.9
1	0	1	0	2	11.9	26.7	14.4	0.84	5.6
1	2	0	1	2	56.2	102.2	611.3	35.75	419.6
1	3	0	1	4	15.4	40.3	10.8	0.63	6.6
0	0	0	1	3	14.9	19	20.4	1.19	7.1
0	0	0	0	3	64.8	66.9	47	2.75	23.6
1	0	0	1	4	39.4	47.9	88.9	5.20	37.6
0	0	0	1	2	54.2	77.9	51.4	3.01	19.9
1	3	0	0	3	64.1	143.3	227.6	13.31	185.4
1	2	0	0	2	15.5	26.9	11.7	0.68	2.9
1	2	0	0	2	35.7	82.6	442.1	25.85	319.9
1	2	0	0	2	390	377.3	471.5	27.57	335.3
1	0	1	1	2	103.1	85.2	109.1	6.38	68.6
1	2	0	0	2	42.7	53.2	57.2	3.35	28.1
1	2	0	0	2	370.8	618.1	374.6	21.91	254.3
0	0	0	0	4	28.5	56	21.6	1.26	10.4
0	0	1	0	2	18	32.2	34.5	2.02	20.2
1	1	0	0	3	77.9	101.9	687.4	40.20	419.5
1	1	0	0	2	761.1	1607	30	1.75	19.1
1	1	0	0	3	522.8	217.4	329.2	19.25	242.9
1	1	0	0	2	50.9	180.9	113.6	6.64	78.2
1	1	0	0	4	55.2	323.8	115.3	6.74	90.7
1	2	0	1	3	31.3	82.1	138.1	8.08	111.3
1	2	0	0	2	67.2	94.4	155.3	9.08	83.8
1	2	0	0	2	47.9	104.4	46	2.69	24.5
1	1	0	0	2	99.3	203.3	449.6	26.29	341.4

1	2	0	0	2	7.4	18.8	54.5	3.19	26.6
1	2	0	0	3	128.9	143.4	552.2	32.29	384.9
1	2	0	0	2	26.1	31	13.9	0.81	6.1
1	1	0	0	2	1193	1861.4	385.1	22.52	275.3
1	2	0	0	2	47.4	112.5	361.3	21.13	247.8
0	0	1	1	2	17.8	31.9	9.2	0.54	4.8
1	3	0	0	2	25.9	53.3	44	2.57	5.2
1	2	1	0	3	98.5	85.3	84	4.91	40.3
0	0	1	1	3	5.8	15.4	2.8	0.16	1
1	2	0	0	3	39.8	44.5	33.3	1.95	16.9
1	1	0	0	2	34.4	53.6	37.1	2.17	20
1	2	0	1	3	19.1	20.3	13.6	0.80	5.6
0	0	1	1	3	16.6	27.2	24.2	1.42	10.5
1	2	0	0	3	149.7	141.6	104.2	6.09	80.9
1	1	0	0	2	91.8	94.9	207.4	12.13	173.2
1	2	1	1	2	84.7	134.3	444.3	25.98	311.9
1	2	1	1	3	5.7	13.5	25.8	1.51	14.3
1	2	0	0	2	31.1	52.7	28	1.64	13.2
1	0	0	0	2	34.2	36.6	8.3	0.49	4.6
0	0	0	0	3	429.8	565.9	340.4	19.91	265.9
0	0	1	1	2	31	39.3	33.5	1.96	21.7
1	2	1	1	4	8.4	16.5	12.9	0.75	10.7
1	0	0	0	2	142.7	152.8	278.1	16.26	202.9
1	2	0	1	2	9.7	18	16.5	0.96	8.3
1	2	0	0	2	50.2	91.1	133	7.78	322.5
1	2	0	0	3	91.4	46.4	18.9	1.11	12.4
1	1	0	0	2	1638.3	1520.7	362.8	21.22	239.1
1	1	0	0	2	125.7	796.7	180.2	10.54	90.5
1	1	0	0	4	28.3	65.6	34.8	2.04	21.1
1	2	0	0	2	320.7	171.2	513.2	30.01	356.1
1	2	0	1	2	45.4	221.7	243.5	14.24	172.7
1	2	0	0	3	27.6	41.8	157.9	9.23	98.6
1	2	0	0	2	13.9	37.6	206.8	12.09	174.7
1	0	0	0	2	1223.5	1203.1	44.4	2.60	10.9
1	2	0	0	2	16.2	61.9	95.9	5.61	60.8
1	0	0	0	2	166.6	180.4	833.6	48.75	595.8
1	2	0	1	2	20.4	34.5	17.8	1.04	8.7
1	2	0	1	3	83	103.6	93.5	5.47	62.6

1	2	1	0	3	30.4	43.6	49	2.87	27.8
1	1	0	0	4	20.1	44.7	170.7	9.98	117.6
1	3	1	1	4	55.1	53	42.9	2.51	29.6
0	0	1	1	2	2127.8	2335.7	397.2	23.23	231.1
1	2	0	0	2	49.6	73.6	183.4	10.73	116.7
1	0	0	1	3	828.6	768.2	179.6	10.50	80.9
1	2	0	0	2	25.8	53.4	73.6	4.30	50.4
1	1	1	1	2	58.4	91.3	85.5	5.00	52.1
0	0	0	0	2	4.5	19.2	12.8	0.75	7
0	0	0	0	2	48.9	70.1	33.5	1.96	14.9
0	0	0	1	4	25.6	33.5	130.4	7.63	38
0	0	0	0	2	381.2	301.3	397.7	23.26	250.6
1	2	1	0	3	19.5	23.6	73.2	4.28	38.8
1	2	0	0	2	41.9	227.8	118	6.90	71.9
1	1	0	0	2	34.1	59.4	45.1	2.64	21.6
1	1	1	0	2	231.7	288.5	401.1	23.46	216.3
1	3	1	1	3	8.6	22.4	34.7	2.03	27
1	2	1	0	2	74.1	96.8	219.4	12.83	173.6
0	0	0	1	2	274.2	338.4	97.4	5.70	68.3
1	3	0	0	2	8.4	21.5	14.9	0.87	6.8
1	2	0	0	3	100.7	112.1	404.9	23.68	331
1	2	1	0	2	17.2	27.6	15.8	0.92	7.9
1	1	1	0	4	596.7	592.9	51.4	3.01	17.1
1	2	0	0	4	128.8	222.9	138.7	8.11	84.3
1	3	0	0	3	4.8	8.4	2.9	0.17	1.2
1	3	1	0	2	1053.6	335.2	231.9	13.56	147.3
0	0	0	1	4	26.2	35.3	31.4	1.84	16.2
0	0	0	0	4	34.9	62.5	43.4	2.54	17.5
1	2	0	0	2	41.2	76.8	509.8	29.81	356.6
1	2	0	0	2	17.8	61.1	244	14.27	175.9
0	0	0	0	3	23.1	45.7	33.4	1.95	24.4
1	2	1	0	2	852.9	617.6	221.5	12.95	156
1	1	0	0	2	140	261.7	490.1	28.66	363.7
1	2	0	1	2	40.1	39	28	1.64	11.5
1	1	0	0	2	30.7	151.2	358.2	20.95	273.8
1	1	0	1	3	19.5	29.7	19.6	1.15	9.1
1	2	0	0	3	63.2	104.1	17.8	1.04	5.2
1	1	0	1	2	13.5	31.5	6.8	0.40	4.3

1	2	0	0	2	53.2	65	105.1	6.15	82.2
1	0	0	0	2	40.7	204.1	342.9	20.05	268.9
1	2	0	0	2	17.2	47.8	146.9	8.59	87.9
1	2	0	0	4	5.4	16.3	136.1	7.96	58.7
1	2	0	0	3	114.1	136	365.4	21.37	219.3
0	0	1	0	2	1758.1	1126.8	424.3	24.81	288.6
1	2	0	1	2	12.7	56.1	412.4	24.12	314.4
1	3	1	1	2	1232.5	873.6	334.4	19.56	200.4
1	2	1	0	2	21.5	36	10.1	0.59	4.4
1	2	0	0	3	19.2	36.7	230.3	13.47	161.7
1	2	1	0	2	57.8	146.2	456.6	26.70	352
1	2	1	0	2	119.5	179.3	89.3	5.22	59.2
0	0	0	0	2	431.7	616.3	202.2	11.82	140.2
1	2	0	0	2	20.9	81.5	120.9	7.07	97.8
0	0	0	0	2	28.2	100.8	181.4	10.61	104.4
1	1	0	0	2	43	38.5	32.1	1.88	15.3
1	1	1	1	2	653.7	252	101.6	5.94	57.8
1	3	0	0	4	27.7	37.9	45.2	2.64	24.5
1	2	1	0	2	29.2	131.2	100.7	5.89	54.5
1	0	0	0	2	17.7	30.2	113.2	6.62	49
0	0	0	0	2	25.5	51	249.3	14.58	137.5
1	2	0	0	2	101.7	115.1	65.7	3.84	46.6
1	1	0	0	2	108.2	233.5	555.9	32.51	416.8
0	0	0	0	2	29.8	45.8	36.4	2.13	25.5
1	1	0	0	2	27.9	38.8	36.8	2.15	24.1
1	1	0	0	3	130.2	99.3	180.2	10.54	89.5
1	2	0	0	2	23.6	39.3	76.2	4.46	28.5
1	1	1	1	2	8.6	21.3	27.6	1.61	7.9
1	2	0	1	3	686.3	588.6	250.9	14.67	120.4
1	1	0	1	3	37.2	67.5	213.7	12.50	122
1	3	0	0	2	31.4	90.5	56.5	3.30	45.9
1	3	0	1	2	12.3	12	21.2	1.24	14.3
1	2	0	0	3	57	175.8	479.5	28.04	370.3
1	0	0	0	3	49	28.1	126.5	7.40	35.9
1	2	0	0	2	32.1	35.2	36.3	2.12	22.2
1	1	1	1	4	362.9	396.9	100.3	5.87	214.1
1	0	1	0	2	67.1	76.5	644.9	37.71	474.8
0	0	0	0	2	27.4	39	11.5	0.67	6.7

1	2	0	0	3	18	88.6	130.8	7.65	101.6
1	2	0	1	2	43.4	118.2	44	2.57	32.1
1	3	0	0	2	25.4	74.4	93	5.44	48.6
1	1	0	0	2	424	772.3	193.4	11.31	139.7
0	0	0	0	3	23	37	44.3	2.59	23.5
0	0	1	1	2	14.2	16.5	13.2	0.77	6.5
1	0	1	1	2	27.2	38.5	28.6	1.67	16.2
1	1	1	1	2	1694.7	1195.3	281.4	16.46	217.7
1	2	0	0	3	26	70.4	560.7	32.79	305.8
0	0	0	0	4	32.6	60.4	162.6	9.51	71.5
1	2	0	0	2	11.7	17	18.9	1.11	10.7
1	2	0	0	3	87.8	62	164.6	9.63	106.3
1	2	1	1	3	32.1	46	140.6	8.22	104.2
0	0	1	0	2	22.7	64.8	58.1	3.40	37.7
1	2	0	0	2	18.8	96.5	127.5	7.46	104.8
0	0	0	0	2	1409.7	3324.2	65.2	3.81	55.7
1	3	1	1	3	14.8	42.7	44	2.57	27.3
1	2	1	0	2	58.1	91	33.8	1.98	22.1
1	2	0	0	3	228.4	426.8	196.9	11.51	143.2
0	0	0	0	2	23.7	38.3	87.8	5.13	20.6
1	2	0	1	4	29	78.7	24	1.40	11.8
1	2	1	1	3	9.3	45.4	51.1	2.99	37.6
1	0	0	1	2	21	20.1	17.4	1.02	6.3
0	0	0	0	3	191.4	200.4	18.9	1.11	9.4
1	2	0	0	2	349.4	156.9	18.3	1.07	10.8
0	0	0	1	3	16.8	24.7	27.1	1.58	18.8
1	3	0	0	3	18.4	40.7	29.1	1.70	17.9
1	1	1	0	4	31.9	74.4	609.9	35.67	342.8
1	2	0	0	3	97	249.2	17.5	1.02	12.7
1	2	1	0	2	62.1	91.2	119.4	6.98	91.8
0	0	1	0	2	15.9	32.3	15.4	0.90	8.1
1	2	0	0	2	11.3	51.5	153	8.95	108
1	1	0	0	2	109.9	131.3	119.1	6.96	71.3
0	0	0	0	4	39.6	40.6	94.4	5.52	19.2
1	0	0	0	3	45.9	68.2	174.5	10.20	120.5
1	3	0	0	3	15.7	23.8	9.3	0.54	5.1
0	0	0	0	2	36.4	94.4	329	19.24	242.6
1	2	0	0	3	19.5	63.1	117.7	6.88	69.6

1	1	0	1	2	20.9	38.8	54.3	3.18	40.8
1	2	0	0	2	243	277.6	19	1.11	11.5
1	3	0	0	2	59	369.9	747	43.68	391.2
1	2	1	0	2	58.6	65.9	93.1	5.44	33.6
1	3	0	0	2	25.4	45.3	44.3	2.59	32.6
0	0	0	0	3	247.8	113.2	39.3	2.30	23
1	0	0	0	2	24.3	24.9	16.8	0.98	9.5
1	3	0	0	3	76.1	95.9	90.4	5.29	47.6
0	0	0	0	2	1705.6	1205.7	204.4	11.95	162.5
1	2	0	0	3	46.1	52.4	343	20.06	227
1	2	0	0	2	37.5	56.1	10.2	0.60	6.6
1	3	0	0	2	16.1	29.9	39.2	2.29	9.8
1	1	0	0	2	12.7	13.4	32	1.87	15
1	3	0	1	2	31.1	37	11	0.64	3.9
1	0	0	1	2	18.6	39.5	174.2	10.19	117.6
1	2	0	0	2	50.8	90.7	427	24.97	250.8
0	0	0	1	2	15.5	23.4	86.2	5.04	17.2
1	1	0	0	3	34.7	56.7	65.1	3.81	48.1
1	2	0	0	3	18.8	44.6	9.8	0.57	4
1	3	0	1	2	38.1	53.1	259.6	15.18	139
1	2	1	0	3	22.8	69.6	483.3	28.26	393.7
1	2	0	0	2	28.2	65.6	61.6	3.60	46
1	1	0	0	3	25.5	58	96.2	5.63	53.9
0	0	1	1	2	421.9	615.8	182.3	10.66	97.1
1	2	0	0	3	12.5	23.7	29.5	1.73	13.6
1	3	1	1	2	59.2	72.1	90.6	5.30	55.4
1	1	0	0	2	161.4	169.8	133.7	7.82	103.6
1	2	0	0	2	12.5	64.3	54.1	3.16	39.5
1	0	0	0	3	284.1	111.4	226.1	13.22	177.8
0	0	0	0	2	1751.8	1390.4	187.3	10.95	98.9
1	1	1	1	2	43.5	63.9	42.8	2.50	22.3
1	2	0	0	3	40.5	111.6	105.7	6.18	91.5
1	2	0	0	3	14.2	37.8	69.3	4.05	57.5
1	2	1	0	2	17.5	57.7	66.9	3.91	53.2
0	0	1	0	2	475.7	402.7	153.2	8.96	124.3
1	0	0	0	3	26	59.5	143.3	8.38	67.9
1	1	1	0	2	44.6	135.8	355.9	20.81	215.8
1	1	0	0	2	142.6	129.5	290.1	16.96	226.7

1	1	0	0	2	19	27.3	13.5	0.79	7.1
1	3	0	0	2	42.8	56.7	19.8	1.16	7.9
0	0	0	1	2	31.5	36.2	209.9	12.27	167.7
1	2	1	1	2	187.2	330.7	325.4	19.03	232.5
1	1	0	0	2	377	154.7	388.4	22.71	243
1	2	0	1	2	17.9	30.6	50.9	2.98	24
0	0	0	0	2	29.7	47.2	230.8	13.50	133.5
1	1	0	0	2	32.3	47.7	130.4	7.63	94
1	2	1	0	3	497.6	362.3	320.1	18.72	234.4
0	0	1	1	3	21.9	53.6	23.2	1.36	14.1
1	0	0	0	3	9.7	36.7	77.5	4.53	50
1	3	0	1	3	36.5	46.9	161.3	9.43	95.1
1	3	0	0	4	36.9	70.4	29.1	1.70	12.8
0	0	0	0	2	69.2	68.8	7	0.41	4.8
1	1	0	0	2	132.2	148.6	497.9	29.12	314.7
1	1	1	1	2	20.5	82.4	200.4	11.72	127
1	3	0	0	3	529.7	1265.7	81.6	4.77	86.1
1	3	0	0	2	19.1	33.2	26.8	1.57	13.7
1	0	0	0	2	88.1	117.8	615.6	36.00	483.1
0	0	0	1	4	45.7	47.5	39.7	2.32	17.1
1	0	0	0	2	78	98.8	176.4	10.32	134.4
1	2	0	0	2	15.9	42.9	136.6	7.99	72.5
1	1	0	0	3	16.9	32.9	44.7	2.61	23.9
1	1	0	0	3	45	114	541	31.64	413.6
1	3	0	0	2	670.6	1056.6	470.8	27.53	262.9
1	3	0	0	2	54.2	74.7	129.2	7.56	62.2
1	2	1	1	3	27.8	40.8	212.5	12.43	148.8
1	2	0	0	2	28.9	185.8	269.2	15.74	183.3
0	0	0	0	2	49.6	118.3	548.2	32.06	394.6
1	3	0	0	2	32.4	96	65.5	3.83	38.7
1	0	0	0	2	32.6	85.3	40.3	2.36	19.8
1	2	0	0	3	194.6	247.6	267.6	15.65	190.1
1	2	1	1	2	26.9	43.6	37.6	2.20	19.8
1	3	0	0	2	39.2	92.5	154.1	9.01	87.5
1	1	0	0	2	25.4	54.9	273.4	15.99	185.5
1	2	1	0	3	50.8	85.1	81.9	4.79	41
0	0	1	1	2	9	14.4	33.3	1.95	20.3
1	0	0	0	2	42.2	100.9	80	4.68	48.7

1	2	0	0	2	236.5	231	463.7	27.12	327.4
1	2	0	0	2	46.2	44.7	20.2	1.18	8.6
1	3	0	0	2	8.7	31.6	16.5	0.96	8.8
1	2	0	0	2	24.2	38.9	58.7	3.43	28.5
0	0	0	0	3	40.1	55.6	406.9	23.80	226.1
1	1	0	1	2	499.4	647	363.9	21.28	203.1
1	1	1	0	2	17.2	62.2	241.3	14.11	129.4
1	2	1	1	2	18.2	30	44.6	2.61	26.6
1	3	0	0	2	150.1	151.9	402.3	23.53	245.1
1	1	0	1	2	21.8	30.2	18.3	1.07	8.3
1	0	0	0	2	28.8	70.7	179.4	10.49	134.7
1	3	0	0	2	10.1	23.1	90.6	5.30	45.9
1	1	0	0	2	107.6	157.4	306.8	17.94	198.7
1	3	0	1	2	11.6	33.5	18.2	1.06	7.4
1	2	0	0	2	46	63	464.8	27.18	336.5
1	2	0	0	2	104.1	128.5	525.2	30.71	365.3
0	0	0	1	3	82	109.2	40.3	2.36	18.2
0	0	0	0	2	20.7	44.1	59.8	3.50	28.5
1	3	1	1	3	42.7	110.5	107.6	6.29	66.6
1	2	0	0	2	101.7	163	551.7	32.26	341.6
1	2	0	0	3	43.2	55.3	395.4	23.12	262.1
0	0	0	1	4	58.3	61.8	70.8	4.14	51.7
0	0	0	0	2	120.9	196	594	34.74	417.2
1	3	0	0	3	39.7	72.7	333.7	19.51	225.8
1	3	1	0	3	41.7	41.7	37	2.16	19.9
1	0	1	0	2	25.8	59.8	371.5	21.73	298.1
1	1	0	0	2	784.9	608.7	357.3	20.89	184.3
1	2	1	0	3	19.3	46.9	164.9	9.64	83
0	0	0	0	2	30.9	67.7	240.9	14.09	183
1	1	1	1	2	39.7	37.7	4.8	0.28	2.3
1	2	0	0	3	20.3	46.7	103.9	6.08	51.9
1	3	0	0	3	17.9	21.8	28.8	1.68	15.1
1	1	1	1	3	87.8	187.6	18.8	1.10	13.6
1	2	0	1	3	38.4	145.8	16.7	0.98	8.2
1	0	0	0	2	16.8	23.9	9	0.53	4.7
1	3	0	0	2	27.7	51.3	195.5	11.43	121.3
1	2	0	0	3	22.5	37.4	33.6	1.96	18.3
0	0	0	0	2	30.9	27.9	56.1	3.28	28.7

0	0	0	0	2	9.9	17.2	61.1	3.57	22.9
0	0	0	1	3	28.7	44.3	222.4	13.01	103.7
0	0	0	0	2	40.9	67.5	88.1	5.15	33.7
1	2	0	0	3	30.7	34.4	51.9	3.04	27.1
1	1	0	0	3	37	51.6	899	52.57	434.4
1	2	1	0	2	207.8	281.1	324.5	18.98	252.9
0	0	0	1	2	25.2	32.8	45.9	2.68	17
0	0	0	0	2	34.4	63.2	61.7	3.61	26.4
1	2	0	0	2	57.2	112.4	556.7	32.56	295.1
1	1	1	1	2	83.7	159.4	429.4	25.11	335.2
1	2	0	0	3	10.9	18.1	44.8	2.62	19
1	2	1	0	2	49.3	73.2	35	2.05	19.4
1	1	0	0	2	27.8	42.4	59.9	3.50	33.8
1	1	0	0	2	523.3	569.5	325.9	19.06	170.1
1	3	0	0	2	32.8	51.4	150.9	8.82	94.4
1	2	0	0	2	39.1	128.4	95.5	5.58	76.1
1	0	0	1	2	968.8	743	218.7	12.79	139.4
1	3	0	0	2	33	65	92	5.38	48.1
1	2	0	0	2	234.8	104.4	599.3	35.05	315.1
0	0	0	0	2	21.1	49.7	32.6	1.91	16.6
1	1	0	0	2	132.4	194.3	134.2	7.85	79.5
0	0	0	0	2	22.4	38	85.3	4.99	57.6
1	1	0	0	2	43.7	67.9	221.1	12.93	186.1
1	2	1	1	2	7	30.7	18.5	1.08	11.2
1	0	1	0	2	38.2	48	16.5	0.96	7.2
1	2	0	0	2	167.9	116.6	418.8	24.49	249
1	1	0	0	2	21.7	22.3	14.4	0.84	3.1
0	0	0	0	2	34.4	53	42.2	2.47	15.5
1	2	0	1	3	13.5	20.2	19.6	1.15	9.9
0	0	0	0	2	43.5	103	72.4	4.23	42.7
1	3	0	1	2	41.2	41.8	20.2	1.18	11.3
1	3	0	0	2	20.6	54.6	386.2	22.58	288.1
0	0	1	1	2	43	64.7	432	25.26	266.3
1	0	0	0	2	22.6	34.9	61.8	3.61	27.3
1	3	1	1	2	8.1	24.4	22.5	1.32	13.4
0	0	0	1	3	36.6	42.9	84.9	4.96	30
1	2	0	0	3	33	45.1	22.9	1.34	12.7
1	2	0	0	2	12.7	16.2	140.5	8.22	26.9

1	1	0	0	3	63.4	193.4	16.4	0.96	9.1
1	3	0	0	2	63.4	193.4	16.4	0.96	9.1
1	3	0	0	2	47.1	75.3	49.2	2.88	27.7
1	1	0	0	2	27.2	56.4	78.4	4.58	17.6
1	2	0	0	2	8.7	30.7	16.1	0.94	10.9
0	0	0	0	2	34.3	41.9	80.2	4.69	33.1
1	1	0	0	2	28.7	77.9	54.8	3.20	31.4
0	0	0	0	2	24	33.1	86.4	5.05	38.5
0	0	0	0	3	81.7	82.3	10.6	0.62	5.2
1	3	0	0	2	36.6	81.2	150.4	8.80	84
1	1	0	1	2	51.3	69.2	106.2	6.21	66.7
1	2	0	0	2	53.7	122.4	37.6	2.20	28.4
0	0	1	0	2	9.6	19.7	35.7	2.09	25.9
1	2	0	1	2	13.5	33.8	25.4	1.49	11
1	3	0	0	2	64.1	104.5	153	8.95	83.2
1	0	0	0	3	77.7	78.6	57.1	3.34	27.1
1	0	0	0	3	41.7	54.5	186.9	10.93	107.3
0	0	1	0	2	57.9	110.9	16.5	0.96	7.1
1	1	1	0	2	16.5	57.8	208	12.16	134.9
1	2	0	0	3	21.7	42	17.3	1.01	10.7
1	2	0	0	2	26.4	88.2	165.5	9.68	142.3
1	0	1	0	2	16.6	18.7	37.4	2.19	16.6
0	0	0	0	2	114.7	57.9	664.9	38.88	439.6
1	3	0	0	3	11	16.2	22.3	1.30	12.1
1	0	0	0	3	46.3	80.8	76.5	4.47	38.4
1	1	1	0	2	32.3	73.5	85.5	5.00	55.9
1	1	1	0	2	33.2	39.2	409.5	23.95	245.4
1	1	0	0	3	31	23	32.8	1.92	17.4
1	2	0	0	3	59.2	76.3	175.6	10.27	84.4
0	0	0	0	2	11.7	19.8	10.3	0.60	6.2
0	0	1	1	3	30.3	42.7	37.8	2.21	22.7
1	2	0	0	2	61.1	32.7	8.7	0.51	3.9
1	0	0	0	2	259.6	72.9	489.9	28.65	341.1
1	3	1	1	2	13.6	28.3	23.4	1.37	13.8
1	0	0	0	2	22.9	31	50.8	2.97	32.9
0	0	0	0	2	63.3	96.9	358.5	20.96	247.5
1	2	0	0	4	15.4	18.1	20	1.17	10.3
0	0	0	0	2	54.2	166.1	434.1	25.39	239.4

1	2	0	0	3	20.5	31.7	28.1	1.64	18.7
1	2	0	0	2	12.1	21.4	26.9	1.57	14.9
1	2	0	0	2	16.9	99.1	159	9.30	107.7
1	0	1	0	2	118.6	238.6	634.6	37.11	393.4
1	3	1	0	2	25.7	49.4	21.9	1.28	12.4
1	3	0	0	2	16.5	35.7	38.7	2.26	25.5
1	2	0	0	3	103.1	75.4	143.3	8.38	105
1	1	0	0	2	52.9	65.6	70	4.09	47.7
1	3	0	0	3	16.6	24.2	213.1	12.46	78
1	2	1	0	3	32.7	37.3	26.9	1.57	15
1	1	0	0	2	184.8	227.7	298.4	17.45	186.6
0	0	0	1	2	120.8	107.9	68	3.98	35.6
0	0	1	0	2	397.8	664.5	121.7	7.12	92.9
0	0	0	1	2	29.1	74.3	31.4	1.84	20.8
1	3	0	0	3	14.3	16.1	118.3	6.92	60.8
0	0	1	0	2	79.4	76.3	30.4	1.78	8.5
1	3	0	0	2	111.7	164.6	21.4	1.25	10.9
0	0	1	1	2	10.8	44.2	92.8	5.43	72.3
1	0	0	1	2	35.5	64.1	206.5	12.08	145.3
1	0	0	0	2	31.3	51.2	70.8	4.14	19.5
0	0	0	0	2	26.2	32.4	44.7	2.61	16
0	0	0	0	2	86.4	320.8	24.6	1.44	12.8
1	2	0	0	2	22.2	64.3	53.6	3.13	23.6
0	0	1	1	2	18.9	44.7	46.9	2.74	21.8
1	1	0	0	3	31.6	36.1	21.8	1.27	11.9
1	2	0	1	4	73.4	85.2	17.2	1.01	8.5
0	0	1	0	3	20.1	51.4	25	1.46	16.2
0	0	1	0	2	13.7	26.9	33.1	1.94	14.4
1	0	0	0	2	147.3	92.3	367.9	21.51	299.2
0	0	0	0	2	18.4	28	10.5	0.61	5.4
1	0	0	0	2	13.3	18.9	11.1	0.65	5.4
1	2	0	1	2	295.5	206	32.1	1.88	17.3
1	0	0	0	2	22.5	110.2	61.2	3.58	27.2
1	1	0	0	2	22.4	38.9	30.3	1.77	15.4
0	0	0	0	2	10.9	16.8	10.3	0.60	5.5
0	0	0	0	2	9	8.5	9.8	0.57	5.4
1	1	0	0	3	26.9	35.6	281.2	16.44	146.4
0	0	1	0	2	16	44.5	35.5	2.08	16.9

0	0	0	0	3	22.8	53.2	93.6	5.47	66.1
0	0	0	0	2	32.5	72.7	87.8	5.13	62.7
1	0	0	0	2	12.2	32.8	72.8	4.26	41.8
1	2	0	0	3	15.7	15.8	21.9	1.28	11.9
1	2	0	0	2	20.6	21.4	11.1	0.65	4.8
0	0	0	0	3	22.9	77.5	38.9	2.27	22.5
1	2	0	1	3	3	11.3	9.3	0.54	4
1	1	0	1	2	913.1	1457.4	135.7	7.94	100.6
1	0	0	0	3	9	17.4	42.1	2.46	14.1
1	2	0	0	2	22.9	22.3	11.7	0.68	3.1
1	2	0	1	3	27.5	97	76.9	4.50	39.3
0	0	0	0	2	69.2	72.4	25.6	1.50	14
1	1	0	1	2	38.2	31.5	7	0.41	2.2
1	3	0	0	4	55.9	119.7	105.6	6.18	27.9
1	3	0	0	2	41.8	62.1	55.3	3.23	33.7
0	0	0	0	2	11.3	1724	11.1	0.65	30.2
0	0	0	0	2	91.5	150.7	172.5	10.09	134.4
0	0	0	0	3	14.4	17.6	9.3	0.54	5.3
1	2	0	0	2	17.5	30.3	24.8	1.45	14.2
1	2	1	1	3	21.1	23.6	6.4	0.37	3.2
1	1	0	0	3	19.8	40.6	64.4	3.77	40.6
1	1	1	0	2	36.1	66.3	106.1	6.20	85.5
1	0	0	0	4	28.2	47.2	34.2	2.00	17.9
1	1	0	1	2	15.3	23.4	7.9	0.46	3.7
1	1	0	1	3	31.1	41.4	73.5	4.30	33.2
1	0	0	1	2	35.5	52.8	47.6	2.78	25.6
1	0	1	1	3	30.2	71.3	74.5	4.36	58.1
1	0	1	1	2	13.5	21.7	33.2	1.94	14.7
0	0	0	1	2	46.4	42.5	17.7	1.04	9.1
0	0	1	0	3	51.4	94.6	90.9	5.32	53.2
1	2	1	0	2	196.5	280.6	7.2	0.42	4.2
1	1	0	0	3	23	26.8	79.8	4.67	29.5
1	2	1	1	2	48.4	99.9	209.4	12.25	166.1
1	2	1	0	2	10.1	18.2	25.6	1.50	7.6
0	0	0	1	2	29.7	69.3	405.9	23.74	304.1
1	0	0	0	3	8.8	20.2	23.8	1.39	7.9
0	0	0	0	3	25.4	40.3	110.9	6.49	36.2
1	3	0	0	2	23.8	33.2	41.3	2.42	26.9

1	1	0	0	2	12	34.8	20.7	1.21	9.2
1	0	0	0	2	12.6	12.8	19.4	1.13	14.6
1	1	1	0	2	138.7	192.1	186	10.88	135
1	0	0	0	3	77.5	121.9	49.9	2.92	31.8
1	0	1	0	2	49.8	64.2	15.8	0.92	8
1	2	0	0	3	7.8	35.2	61.9	3.62	46.4
1	1	0	0	3	16	38.5	26.9	1.57	16.1
0	0	0	0	2	36.2	64.1	105.5	6.17	80.5
1	0	0	0	2	15	10.2	8.4	0.49	3.9
0	0	0	1	2	34.8	59.8	87.6	5.12	53.3
1	0	0	0	2	34.3	58.1	54.9	3.21	33
1	2	0	0	2	83.8	341.2	211.5	12.37	158.8
0	0	0	1	2	31.4	40.6	89.6	5.24	27.1
1	0	0	0	3	16.5	41.5	74.8	4.37	58.5
1	2	0	1	2	7.8	9.7	13.9	0.81	5.9
0	0	0	0	2	22.2	19.9	25.6	1.50	9.8
1	2	0	0	2	55.4	72.6	44.4	2.60	27.1
1	0	0	0	3	174.8	365.5	17.4	1.02	8
1	2	0	0	2	13.9	27.4	24.3	1.42	10.3
0	0	0	0	3	30.4	31.3	59.6	3.49	19
1	1	0	0	3	85.8	90.4	24.9	1.46	12.1
1	1	0	0	2	553	252.1	432.9	25.32	270.8
1	3	0	1	3	13.9	20.4	22.1	1.29	14.2
1	3	0	0	2	26.4	36.7	87.7	5.13	55.8
1	1	0	0	2	18.7	48.2	115.4	6.75	94
1	2	0	0	2	46.4	155.2	310.9	18.18	217.7
0	0	0	0	3	46.5	63.6	92.8	5.43	45.8
1	0	0	1	2	53.9	75.5	17.4	1.02	7.8
1	0	0	1	2	56.3	83.8	72.3	4.23	49.1
1	1	0	0	3	10.2	15.3	5.6	0.33	2.5
0	0	0	0	2	11.2	29.5	91.4	5.35	55.2
0	0	1	0	2	19.9	39	41.3	2.42	23.8
0	0	0	0	2	2013.5	2787	97.3	5.69	80.1
1	0	0	0	3	48.5	74.4	55.3	3.23	30.7
1	2	0	1	2	18.8	30.4	37.6	2.20	19.2
0	0	0	0	2	19.7	35.3	34	1.99	24
0	0	0	0	2	33.7	25.2	18.9	1.11	8.9
1	0	0	0	3	16.3	31.5	60.8	3.56	18.2

1	0	0	1	2	15.6	30.7	180.9	10.58	57.3
1	0	1	1	3	25.4	38.7	61.4	3.59	39.7
1	3	0	0	3	17.3	32.8	17.6	1.03	7.8
0	0	1	0	2	21.2	39.9	47.5	2.78	24.6
1	0	0	1	3	25.1	32.5	63.8	3.73	34.5
1	0	0	0	4	25.8	40.7	26.4	1.54	11.5
0	0	0	0	2	110	144	18.4	1.08	9.8
1	2	0	1	3	34	40.8	29	1.70	14.6
1	1	0	0	2	76	80.5	31.4	1.84	17.3
1	1	0	0	2	21.4	20.9	6.5	0.38	4.4
0	0	0	0	3	35.1	46.1	70.6	4.13	20.3
1	3	0	0	2	22.4	45.1	44.7	2.61	28.7
0	0	0	0	3	46.3	63.7	29	1.70	11.6
1	0	0	1	2	22.2	34	22	1.29	11.5
1	1	1	1	2	10.4	32.8	38.2	2.23	26.4
0	0	1	1	4	17.9	16.9	9.6	0.56	6.2
1	1	0	0	3	54.8	236.9	187	10.94	123.7
1	2	0	1	2	50.7	44.3	185.1	10.82	137.7
0	0	0	0	2	18.7	38.2	42.5	2.49	18.3
0	0	0	1	2	7.5	12.2	7.9	0.46	4.2
1	2	0	0	2	60.2	104.2	60.4	3.53	42.3
0	0	0	0	3	32.3	81.4	245.2	14.34	107.6
0	0	0	0	2	38	45.9	39.2	2.29	14.8
1	2	1	0	2	91.7	120.9	65.8	3.85	36.8
1	1	0	0	2	20.3	29.3	68.5	4.01	34.3
1	1	0	0	2	34.3	42.8	110	6.43	78.3
1	2	0	0	2	112.1	165.6	373.2	21.82	285.4
0	0	1	1	3	34	24.2	45.5	2.66	33.5
1	2	0	1	3	39.4	53.4	27.9	1.63	6.9
1	1	0	0	2	469.3	177.8	367.7	21.50	259.5
1	2	0	0	3	44.9	51.4	18.9	1.11	6.8
1	3	0	0	3	25.4	33.4	87.4	5.11	55.9
1	3	1	1	2	9.3	22.9	35	2.05	17
1	2	0	0	3	41.7	31.8	52.2	3.05	44.3
1	2	0	0	3	48.4	57.2	164.6	9.63	108.2
1	0	0	0	2	11.5	47	67.1	3.92	27.8
0	0	0	0	2	48.6	94.3	40.8	2.39	24.9
1	0	1	0	2	6.6	40.5	24.2	1.42	15.7

1	2	0	0	2	9.7	9.6	23.3	1.36	10.7
1	1	1	0	2	539.1	445.8	280.1	16.38	197.4
0	0	0	0	2	28.7	77.8	69.1	4.04	33.1
1	3	0	1	2	50.8	65.3	44.2	2.58	23.6
0	0	0	0	4	24.4	86.9	142.5	8.33	104.7
1	0	0	1	2	37	109.2	50.7	2.96	28.6
1	1	1	0	3	12.4	39.1	26.1	1.53	18.2
1	0	0	0	2	27	50.3	22.6	1.32	8.9
1	1	1	0	4	21.1	43.6	46.9	2.74	31.4
0	0	0	1	3	33.1	39.2	26.2	1.53	14
1	2	1	1	2	27.5	38.5	25.3	1.48	13.4
0	0	1	0	2	15.9	26.4	33.2	1.94	12.1
1	1	0	0	2	10.2	20.5	7.9	0.46	4.2
0	0	1	0	3	29.3	59.3	101.6	5.94	60.6
0	0	0	1	2	18.4	23.9	40.4	2.36	17.9
1	0	0	0	2	12.9	34.6	28.2	1.65	18.2
1	1	0	0	2	131.7	150.6	130	7.60	71.9
1	3	0	0	2	46.9	100.9	178.1	10.42	120.5
1	2	0	0	2	32.4	23.2	10.4	0.61	6.1
1	1	0	1	2	22.5	49	134.6	7.87	82.1
1	2	0	0	2	35.4	68.5	43.6	2.55	19.9
0	0	0	0	3	48.3	41.6	16.9	0.99	8
0	0	0	0	2	20.6	27.5	28.6	1.67	12.3
0	0	0	0	4	34.3	45.8	51.6	3.02	21.2
1	2	0	0	2	24.1	42.4	37.8	2.21	14.1
1	1	0	0	2	13.4	26.6	14.2	0.83	5.8
1	1	0	0	2	42.2	83.8	13.6	0.80	7.8
0	0	0	0	3	14.3	20.8	48.1	2.81	23.4
1	0	0	1	3	20.2	28.1	38.2	2.23	15.1
0	0	0	1	2	32.1	169.8	107.6	6.29	83.7
0	0	0	0	3	18.1	30.7	35.6	2.08	16.1
1	0	0	0	2	14.4	21	35	2.05	16.6
1	2	0	0	2	7.6	21.1	19.4	1.13	13.1
0	0	0	1	4	40.3	58.2	38.5	2.25	15.6
1	0	1	1	2	419	1361.1	58	3.39	44.5
1	0	0	0	3	14.4	17.6	17.3	1.01	9.1
1	1	0	0	2	18	21	27.7	1.62	12.7
1	0	1	1	3	23.2	39.4	27.1	1.58	12

1	1	0	0	2	14.5	52.9	107.1	6.26	69.5
1	2	1	1	2	24.2	45.1	58.8	3.44	36.2
0	0	1	0	2	39.4	81	57.1	3.34	40.2
1	1	0	0	2	16.5	24.8	3.4	0.20	1.9
1	1	1	0	2	19.2	34.3	89.4	5.23	44.9
0	0	0	0	2	26.3	155.7	69.2	4.05	40.8
1	0	0	0	2	237.4	205.9	346.9	20.29	220.4
1	2	0	0	3	23	41.5	67.6	3.95	41.2
1	1	1	0	2	45.9	86.7	24.9	1.46	18.7
1	0	0	0	2	18.1	41.8	11.1	0.65	5.2
1	2	0	0	3	26.5	37.7	53.9	3.15	24.6
1	1	0	1	2	33.2	31.6	15.9	0.93	8.1
0	0	0	1	2	18.5	31.2	133.9	7.83	110.8
1	1	0	1	2	18.8	30.6	43.9	2.57	27.4
1	3	0	0	2	30.3	50.3	45.7	2.67	20.4
0	0	0	0	2	119.4	103.4	9.9	0.58	4.5
1	3	0	0	2	11	21.9	72.2	4.22	47.3
0	0	1	0	4	4.9	30.8	39.7	2.32	19.8
1	2	0	0	3	60.3	87.6	42.7	2.50	28.1
1	0	0	0	3	35.9	64.2	45	2.63	27.7
1	0	0	0	4	31.5	32.3	155.3	9.08	79.9
1	0	0	0	2	16.5	24.1	27.8	1.63	12.5
1	2	1	1	2	139.5	354	70.2	4.11	47.5
1	2	1	1	2	42.2	45.7	240.8	14.08	156.9
1	0	1	1	3	35.3	40.9	83.3	4.87	26.8
1	0	0	0	2	10.6	17.8	11	0.64	5
0	0	0	0	2	25.6	46.8	24.8	1.45	12.9
1	3	0	0	2	8.3	9.4	12.2	0.71	6.5
0	0	1	1	2	30.4	36	93	5.44	13
0	0	0	0	2	13.4	18.1	9.2	0.54	4.8
0	0	1	0	2	22.6	33.5	26.2	1.53	11.3
1	2	0	0	4	38.2	114	70.1	4.10	58.2
0	0	0	0	3	40.6	47.3	58.4	3.42	28.3
1	1	0	0	2	24.9	43.8	12.1	0.71	5.6
0	0	0	0	3	38.9	52.3	197.1	11.53	125.2
1	1	0	0	2	24.5	26.7	22.9	1.34	10.7
1	3	0	0	2	37.6	61.4	85.4	4.99	57.2
1	0	1	0	2	22.5	37	24.7	1.44	10.6

1	2	0	1	4	27.4	42.8	31	1.81	17.5
1	0	0	0	2	52.2	79.5	74.4	4.35	28.4
1	2	0	0	2	25.9	77.9	83.2	4.87	51.3
0	0	0	0	2	11.4	16.3	46.1	2.70	16.6
1	3	0	0	2	32.9	85.9	62.4	3.65	43.6
0	0	0	0	3	27.4	43.3	52.9	3.09	18.2
0	0	0	1	2	26	29.3	28.9	1.69	13.7
1	2	1	1	2	18.1	36.5	25.5	1.49	11.2
1	1	0	0	3	31.1	44	42.7	2.50	19.8
1	3	1	1	2	5.6	23.3	7	0.41	4.7
1	0	0	0	3	21	42.1	18.4	1.08	11.7
0	0	0	0	2	1617.7	1259.3	186.4	10.90	142.9
1	2	0	0	2	1398.2	591.4	162.1	9.48	135.1
0	0	0	0	4	34.7	42.1	53.3	3.12	19.4
1	1	0	0	2	27.7	44	38.3	2.24	12.5
1	0	1	1	2	10.4	18.1	58.8	3.44	17.6
1	2	0	0	2	751.4	246.4	337.6	19.74	247
0	0	0	0	2	36.4	53	13.9	0.81	6.9
1	2	0	0	2	36.5	59	56.1	3.28	40.5
1	1	0	0	2	22.6	67.8	175.8	10.28	68
1	2	0	1	2	65	318.8	103.6	6.06	67.1
0	0	0	0	2	22	29.9	48.1	2.81	19.7
0	0	0	0	2	21.4	44.5	91.4	5.35	43.3
0	0	0	0	2	52.4	140.9	254.9	14.91	153.6
0	0	0	0	2	23.4	31.6	31.8	1.86	12.3
1	1	1	0	3	23.8	31.6	15.1	0.88	7.3
1	2	0	1	2	13.6	23.2	7.6	0.44	3.5
1	2	0	1	2	39.5	68.9	158.1	9.25	118.6
1	2	0	0	2	24.6	58.6	31.5	1.84	29.2
0	0	0	1	3	42.2	41.8	22.4	1.31	10.2
1	0	0	0	3	29.9	31.8	30.8	1.80	21.8
1	1	0	0	3	17.3	28.2	51.5	3.01	20.1
1	2	0	0	2	35.7	55	16.2	0.95	9.7
1	2	0	1	2	13.5	16.8	36.9	2.16	17
1	1	0	0	2	85.7	66.5	376.4	22.01	218.9
1	3	0	0	3	17.9	16.6	18.1	1.06	8.5
1	1	1	0	3	30.7	34.2	18.8	1.10	5.8
1	0	0	0	2	21.4	26.4	14.8	0.87	5.6

1	2	1	1	2	13.1	37.4	36.9	2.16	23.7
1	2	0	0	2	7.7	30.8	110.8	6.48	85.7
1	2	0	0	2	19.6	35.8	79.4	4.64	51.5
1	2	0	0	2	22.9	101.3	115.1	6.73	95.5
0	0	0	0	2	20.7	60.6	24.5	1.43	12.3
0	0	0	0	3	57	145.4	73.3	4.29	56.4
1	0	0	0	3	14.3	22.9	43.2	2.53	24.3
1	1	0	1	3	22.9	30.1	35.8	2.09	17.7
1	0	1	0	3	19.1	35.6	48.7	2.85	20.7
0	0	0	0	3	24.2	39.1	24.7	1.44	12.1
1	3	0	1	2	16.6	28.1	23	1.35	12.9
1	1	0	1	2	556.3	487	8	0.47	3.9
0	0	0	0	2	274.1	187.8	406.9	23.80	309.5
0	0	0	0	2	60	61.8	25.1	1.47	12.7
0	0	0	0	2	29.7	48.8	69.7	4.08	20.3
0	0	1	0	2	40.8	69.3	61.5	3.60	47.3
1	3	1	0	2	16.9	31.6	47.5	2.78	28.5
1	0	1	0	2	32.4	128.4	118.4	6.92	74
0	0	1	0	2	1560.1	1670.2	91.1	5.33	71.4
1	0	1	1	2	13.9	16.1	18.1	1.06	5.1
1	1	0	0	3	16.2	15.7	8.3	0.49	5.2
1	3	0	1	2	17.2	21.5	13.7	0.80	7.2
1	0	0	0	2	47.2	62.9	75.3	4.40	43.9
1	0	0	0	2	33.4	41.6	47.2	2.76	25.6
1	1	0	0	2	34	36	16.3	0.95	7.7
0	0	0	1	3	53.7	53.6	45.9	2.68	23.7
1	2	0	1	3	18.6	26.3	70.8	4.14	54.7
0	0	0	1	3	14.6	23.8	33.3	1.95	14.6
1	0	0	0	3	258.6	325.2	22.9	1.34	13.3
1	2	0	0	2	11.7	17.7	18.9	1.11	7.3
1	0	0	0	2	16.4	24.9	33	1.93	17.7
1	2	0	0	3	40.8	69.5	120.7	7.06	58.8
1	0	0	0	2	18.5	37.9	27.4	1.60	25.3
0	0	0	0	2	32.6	20.3	66	3.86	14
0	0	0	0	3	10.7	37.2	50.4	2.95	11
0	0	0	0	3	13.8	28.5	41.7	2.44	18.9
1	1	1	0	2	7.2	12	10.1	0.59	4.9
1	0	0	0	3	34.9	38.6	35.1	2.05	19.6

1	2	0	0	2	20.8	30.8	60.5	3.54	26.7
0	0	0	1	2	25.1	26.1	17.7	1.04	6.1
0	0	0	0	2	30.2	43.9	12.9	0.75	7.5
1	0	0	0	2	26.1	104.4	112.1	6.56	64.7
1	2	0	0	2	9.1	25	89.2	5.22	45.6
1	1	0	0	2	45.6	51.7	246.7	14.43	202.8
0	0	0	0	2	20.1	22.1	60.7	3.55	12.2
0	0	0	0	2	1002	627	444.3	25.98	297.6
0	0	1	0	2	12.2	26.1	10.3	0.60	4.6
1	2	0	0	2	175.2	243.5	88.5	5.18	74.2
1	1	0	0	4	45.7	41.9	29.1	1.70	13.6
1	2	0	0	4	46.2	104.1	52.4	3.06	24.3
0	0	0	0	2	34.8	47.3	58.2	3.40	19.1
1	2	1	0	2	22.7	28.8	72.1	4.22	32.2
1	0	0	0	3	36	65.7	31.2	1.82	14.7
0	0	1	0	2	24.4	39.4	27	1.58	9
1	0	0	0	4	41.3	40.4	125	7.31	69
1	2	0	1	2	7.8	12.3	46.8	2.74	32.4
1	1	0	0	2	29.2	36.7	70	4.09	11.4
1	2	0	0	3	22.3	39.9	45.1	2.64	15.4
0	0	0	0	4	189.1	430.2	81.7	4.78	54
1	2	1	0	2	22.7	39.5	25	1.46	10.9
0	0	0	1	2	37	70.1	85.8	5.02	68.2
1	3	0	0	2	25.5	26.9	51.9	3.04	24.5
1	0	0	1	2	25.8	35.1	75.1	4.39	48.9
1	1	0	0	2	1032.9	343.1	307.3	17.97	236.5
1	2	0	0	2	17.9	19.3	14	0.82	6.8
1	2	0	0	3	211.3	128.8	50.8	2.97	29.1
1	2	0	0	2	42.4	107.8	65.3	3.82	41.1
1	2	0	1	2	12.7	14.4	31.3	1.83	16.4
1	1	1	1	2	24	39.7	75.8	4.43	39.5
1	3	0	0	3	15.2	22.7	31.9	1.87	13.6
1	0	0	1	4	21	54	130.7	7.64	80.4
0	0	0	1	2	28.4	27.8	14.1	0.82	5.7
0	0	0	1	2	20.2	30.1	47.6	2.78	25.4
1	0	1	0	3	10.8	17	14.6	0.85	4.8
1	2	0	0	2	30.9	64	47.3	2.77	26.6
0	0	0	0	4	31.5	56.2	22.6	1.32	10.2

1	2	0	1	2	30.2	37.9	30.5	1.78	14.9
1	0	1	0	4	36.6	50.8	44.7	2.61	23.6
1	0	1	1	2	10.6	21	9.6	0.56	4.8
1	2	0	1	3	41.3	93.6	238	13.92	188.7
1	1	1	1	3	30.8	33	10.6	0.62	5.2
1	3	0	0	2	67	93.5	173.7	10.16	137.4
0	0	0	0	2	59.5	108.6	27.1	1.58	15.5
0	0	1	0	2	12.9	22.7	31.5	1.84	13.5
1	0	0	0	3	25.1	49	27.9	1.63	17.8
1	0	1	1	2	33	36	39.3	2.30	14.4
1	1	0	0	2	47.5	50.9	37.5	2.19	14.2
1	1	0	0	2	16.2	19.8	72.8	4.26	20.6
1	2	0	1	2	94.5	669.1	85.2	4.98	40.4
1	2	0	0	2	21.2	53.8	145.8	8.53	103.8
0	0	0	1	3	43.2	60.9	35.8	2.09	19.5
1	1	0	0	3	41.8	56.2	36.5	2.13	16.5
1	2	0	0	2	316.1	185.8	143.1	8.37	119.4
1	2	0	1	2	55.4	343.4	29.5	1.73	22.6
0	0	0	0	3	71.3	76.9	52.5	3.07	26.8
0	0	0	0	3	40.5	50.5	20.3	1.19	9.2
1	1	0	0	3	38.6	52.2	83.6	4.89	29.6
1	2	1	1	2	769.1	875	53.4	3.12	41.5
1	0	0	0	3	23.1	23.4	50.4	2.95	16.7
0	0	0	1	2	27.1	34.1	34.5	2.02	16.8
0	0	0	0	3	20	32.8	33.7	1.97	15.5
1	1	0	0	2	64.5	144.3	475.2	27.79	369.6
0	0	0	1	2	18.7	11.4	35.2	2.06	13.9
1	0	0	0	2	18.2	25.7	24.7	1.44	11.1
1	0	0	0	3	26.5	43.3	32.2	1.88	15.1
1	2	0	0	3	31.5	62.9	111.4	6.51	63.7
1	2	0	0	2	19.7	61.8	95.7	5.60	51.7
0	0	1	0	3	44.5	151	107.5	6.29	64.5
1	0	1	1	2	16	20.3	13	0.76	3.6
0	0	0	0	2	24.2	28.6	7.5	0.44	2.8
0	0	0	0	4	25.2	25.9	11.6	0.68	3.9
1	0	1	0	3	174.9	376.5	190.4	11.13	85.1
1	0	1	1	4	18.2	29.8	35.2	2.06	12.7
0	0	0	0	2	128.8	160.4	12.7	0.74	6.6

1	1	0	0	2	125.1	62.4	121.7	7.12	97.1
1	1	0	0	3	28.5	36.4	22.7	1.33	11.7
1	2	0	0	2	231.4	109.5	269.9	15.78	216.9
0	0	0	0	3	23.7	56.9	48.8	2.85	24.5
0	0	0	1	3	10.7	15.6	18	1.05	10
0	0	0	0	2	92.3	96.2	76	4.44	33
0	0	0	0	2	57	85.9	16.8	0.98	10.4
1	0	0	0	2	12.5	25.2	106.6	6.23	74.2
0	0	0	1	2	20.9	25	24	1.40	7.4
0	0	1	1	3	14.5	29.4	27	1.58	9.5
0	0	0	1	2	30.7	71.6	75.1	4.39	56.7
0	0	0	0	2	17	22.6	52.7	3.08	14.1
1	1	0	0	2	279.5	182.2	379.8	22.21	268.9
1	2	0	0	2	9.5	13.9	4.9	0.29	1.7
1	2	0	0	3	32	81.5	632	36.96	429.9
1	2	0	0	2	693.4	545.1	213.3	12.47	139.3
1	1	0	0	2	24	31	27.7	1.62	9.8
1	1	0	1	2	19.9	39.1	19.3	1.13	9.3
1	0	0	0	3	22.3	54.1	94.9	5.55	52.4
0	0	0	1	2	24.5	29.7	17	0.99	7.6
0	0	1	1	2	703.4	145	368.8	21.57	234.6
1	0	0	0	2	45.3	56.4	34.4	2.01	10.5
0	0	0	0	3	23.6	36.9	34.3	2.01	14.5
1	0	0	1	2	71.6	77.5	88.7	5.19	37.9
0	0	0	0	4	35.7	54.5	104.2	6.09	52.6
0	0	0	0	2	25.5	43.3	21.7	1.27	10.4
1	3	0	1	2	33.4	54.2	23.8	1.39	9.9
0	0	0	0	2	20.5	25.6	30	1.75	13.6
1	0	0	0	2	20.7	32.7	24.5	1.43	11.4
1	2	0	0	2	113.6	187	244.8	14.32	165.5
0	0	0	0	2	14.3	19.5	29.7	1.74	16.6
1	2	0	1	2	75.6	116.7	217.4	12.71	172.2
1	1	0	0	2	85.2	201.8	29.1	1.70	18.9
1	3	0	0	2	100.7	71.3	261.7	15.30	147
0	0	0	0	3	19.3	27.2	44.9	2.63	17.6
0	0	1	1	3	27.3	42.5	67.1	3.92	41
0	0	1	1	2	50.5	112.4	150.4	8.80	78
0	0	0	0	3	10.2	14.3	27.1	1.58	13.8

1	2	0	0	2	19.9	33.7	12.4	0.73	5.5
0	0	0	1	2	22.4	47.5	28.1	1.64	7.3
1	2	0	1	2	21.8	19.5	10.2	0.60	3.9
1	2	0	0	2	31.7	91.1	34.5	2.02	20.1
1	2	0	0	4	11.7	12.5	16.1	0.94	7.5
0	0	1	1	3	12.6	19.7	8.6	0.50	4.8
1	2	0	0	2	10.8	28.2	30.5	1.78	17.3
1	1	0	1	2	20.4	22.8	32.4	1.89	12.3
0	0	0	0	3	20.9	26.6	50.5	2.95	15.9
0	0	0	0	2	466	373.7	314.4	18.39	228.4
1	0	0	0	3	31	97.3	49.8	2.91	42.9
0	0	0	1	3	55.9	45.2	20.2	1.18	6
0	0	1	1	2	22.2	28.7	30.6	1.79	10.4
1	1	0	0	2	43.1	138.1	312.1	18.25	193.9
1	2	0	0	2	29.6	39.8	89.7	5.25	37.2
1	1	0	1	2	33.3	34.7	21	1.23	8.9
1	0	1	1	2	43.5	57.3	38.4	2.25	14.1
1	2	1	0	2	11.2	16.7	7.2	0.42	3.5
0	0	0	0	2	16.5	24.5	22.9	1.34	8.1
1	1	0	0	2	19.2	21.3	12.3	0.72	5.6
0	0	0	1	3	218.8	248.4	30.8	1.80	13.4
0	0	0	0	2	23.6	46	11.9	0.70	1.8
1	3	0	0	2	14.5	35.2	66.1	3.87	43.3
1	0	0	0	2	63.9	52.8	18.6	1.09	9.3
0	0	0	0	2	25.2	31.3	27.9	1.63	9.4
1	2	0	0	3	350.1	268.6	367	21.46	260.2
1	1	0	0	4	34.2	44	73.7	4.31	30.1
0	0	0	0	4	39.3	54.1	37	2.16	19.3
1	0	0	0	2	23.4	70.7	27.8	1.63	11.9
1	0	1	0	3	48.6	64.9	35.5	2.08	10.9
0	0	0	0	3	48.9	54.6	93.3	5.46	12.6
0	0	0	1	2	1607.5	1696.9	226.6	13.25	166.5
1	2	0	0	3	19	33.7	37.6	2.20	19.7
0	0	0	0	4	18.1	46.5	27.7	1.62	12.6
0	0	1	0	2	41.1	74.5	63.4	3.71	22.6
1	1	0	0	4	47.8	122.6	64.2	3.75	30.3
0	0	0	0	2	28.1	43.1	31.9	1.87	13.3
0	0	0	1	3	25.4	61.3	52.5	3.07	18

1	1	0	0	2	31.7	79.9	57.7	3.37	48.5
1	2	0	0	2	35.1	67.7	452	26.43	288.2
1	2	0	0	4	40.8	37	60.2	3.52	21.8
0	0	1	1	2	27.7	35.5	27.4	1.60	12.2
1	0	0	1	3	67.7	49.9	176	10.29	135.4
1	1	0	1	2	10.4	11.8	12	0.70	5.3
1	1	0	0	2	27.2	29.3	21.7	1.27	8.5
1	2	0	0	2	232.1	197	294.3	17.21	201.9
1	2	0	0	3	19.1	33.4	18.6	1.09	8
0	0	0	1	2	46.4	51.9	78.9	4.61	48.4
1	1	0	0	2	34.1	37.3	11.6	0.68	4.4
1	2	0	0	4	23.8	30.9	35.5	2.08	15.5
1	1	0	1	2	14.9	28.7	33.6	1.96	18.8
0	0	0	0	3	19.1	16.7	14	0.82	4
0	0	0	1	2	22.7	23.8	24.2	1.42	13.8
0	0	0	0	2	539.5	334.8	429.5	25.12	315.7
1	1	0	1	2	26.1	29.7	58	3.39	21.6
0	0	0	0	2	38.7	60.4	23.6	1.38	9.4
1	0	0	0	3	52.4	99.9	30.9	1.81	12.6
1	1	0	1	2	17.3	25.4	54.1	3.16	21.6
1	1	0	1	4	6	21.4	35.8	2.09	10.2
1	1	0	0	3	26.9	50.9	73.7	4.31	37.4
0	0	0	0	3	28.2	48.9	44.6	2.61	23.8
0	0	0	0	3	34.5	44.6	33.8	1.98	11
0	0	0	0	2	813.9	250.2	220.9	12.92	125.5
0	0	0	0	2	17.6	22.7	8.6	0.50	3.3
0	0	0	0	3	17.9	86.3	15	0.88	4.4
1	0	0	0	2	18.1	38.6	54.3	3.18	37.1
1	1	0	0	2	27.9	33.3	29.4	1.72	11.9
1	0	0	1	2	18.4	23	18.3	1.07	6.9
0	0	0	0	2	50.3	131.4	57.7	3.37	32.4
0	0	1	0	3	17.1	29.3	90.1	5.27	35.8
1	0	0	1	2	32.1	64.5	133.6	7.81	72.2
0	0	0	0	3	41	74.6	231.8	13.56	96.6
1	1	0	0	2	7.5	29.3	170.4	9.96	123.5
0	0	0	0	2	30	43.4	16.8	0.98	5
1	0	0	0	2	2141.9	2070.8	306.9	17.95	230.8
0	0	0	0	2	31.1	43.3	66	3.86	28.7

0	0	0	1	3	129.3	114	50.3	2.94	27.6
1	2	0	0	2	29.2	30.2	46.4	2.71	21
0	0	0	0	2	22.6	27.1	20.7	1.21	8.3
1	2	0	0	2	130.5	585.7	82.6	4.83	68.1
1	3	0	0	2	40	85.6	19.3	1.13	13.9
1	1	0	0	2	111.5	380.1	414.4	24.23	247.2
1	2	0	1	4	24.9	34.1	29.5	1.73	12.5
0	0	1	0	2	17.9	39.7	38	2.22	20.2
1	0	0	0	2	34.4	37.6	41.7	2.44	14.8
0	0	0	0	2	20.9	29.5	15	0.88	7
0	0	0	1	2	22	19.8	6.7	0.39	1.5
1	1	0	0	2	19	28.5	15.8	0.92	8.1
1	0	1	1	4	22.1	34.2	132.9	7.77	54.3
0	0	0	1	2	18.4	25	12.3	0.72	2.1
1	1	0	0	2	34.3	67.2	15.5	0.91	10.7
0	0	0	1	3	2991.7	1205	253.4	14.82	169.4
1	2	0	0	4	21.3	41.5	17	0.99	8.8
1	1	0	0	3	21.1	30.8	41.1	2.40	21.3
1	3	0	0	4	38.1	49.2	39.8	2.33	13.5
1	2	0	0	2	19.2	61.5	48.8	2.85	33.1
0	0	0	0	2	86.9	96.6	9.7	0.57	3.5
0	0	0	0	2	19.6	31.9	28.5	1.67	9.1
0	0	0	0	2	61	86.6	83.6	4.89	55.6
0	0	0	0	4	29.4	29.9	16.1	0.94	5.5
0	0	0	0	2	43	44.8	14.7	0.86	5
1	2	0	0	4	28.9	46.9	22.5	1.32	8.8
1	3	0	1	3	14.6	27.9	15.7	0.92	8.1
1	1	0	0	2	351.7	2229.8	81.8	4.78	70.4
0	0	0	0	2	71.1	90.3	378.2	22.12	301.5
0	0	0	0	3	13.2	18.3	7.3	0.43	2.3
1	1	1	0	2	2204.6	756.7	269.5	15.76	181.9
1	2	1	0	3	66.9	86.9	64.7	3.78	55.7
0	0	0	0	4	291.7	374.9	616.9	36.08	341.3
1	3	0	0	2	188.6	520.4	442.8	25.89	258.5
0	0	1	0	2	32.1	72.7	263.1	15.39	107.6
1	2	1	0	2	45.7	33.4	244.1	14.27	119
1	3	0	1	2	15.1	21.8	22.2	1.30	11.6
1	0	0	0	2	253.1	474.7	294.4	17.22	167.7

1	2	0	0	2	1236.5	1202.2	214.1	12.52	155.3
1	3	0	0	2	17.5	27.4	35.5	2.08	21.2
1	3	0	0	3	162.9	529.4	502.9	29.41	283.6
1	1	1	1	3	2440.7	2238	435.3	25.46	311.1
1	3	0	0	3	4	23	149.5	8.74	69.8
1	2	0	0	2	151.2	149.8	854.5	49.97	454
1	2	1	0	2	12.4	26.3	30.5	1.78	17.4
1	2	0	0	2	14.7	32	60.5	3.54	44.5
0	0	0	1	3	48.7	77.2	300.6	17.58	234.7
1	0	1	0	2	58.8	70.6	234.6	13.72	169.6
1	2	1	1	2	746.5	670.1	192.3	11.25	144.6
1	2	0	1	4	2194	2537	116.2	6.80	58
1	2	0	0	3	38	229.1	366.1	21.41	268.9
1	1	0	0	3	149.3	151.7	488.9	28.59	263.7
1	0	0	0	2	62.7	170.9	425.9	24.91	313.4
1	2	0	0	3	109.4	284.1	221.8	12.97	164.4
1	1	1	1	3	118	224.9	251.6	14.71	195.8
1	0	0	0	2	279.6	197.8	674	39.42	426.3
1	1	1	1	2	1381.2	864.7	433.3	25.34	196.3
1	2	0	0	3	25	47.5	21.9	1.28	15.3
1	1	1	0	2	110.6	120.5	185.2	10.83	122.3
1	2	1	0	3	8.4	14.6	64.5	3.77	43.8
1	0	0	0	2	65.7	123	239.2	13.99	197.9
1	2	0	0	2	72.4	103.3	359.9	21.05	266.4
0	0	1	0	4	29.5	51.4	52.4	3.06	27.3
0	0	0	0	2	375.6	243.1	285.2	16.68	196.1
1	0	0	0	3	90	228.7	296.7	17.35	79.6
1	3	0	1	2	72.3	64.8	226.9	13.27	146.6
0	0	1	0	2	32.6	49.3	47.6	2.78	24.4
1	2	0	0	4	25	43.6	42.3	2.47	22.2
1	2	0	0	2	35.1	59.6	151.8	8.88	93.7
0	0	0	0	2	19.5	35.4	139.8	8.18	82.3
1	3	0	0	2	25.4	39.3	174.1	10.18	108.1
1	0	0	0	2	448.5	433.1	419.1	24.51	321.5
1	2	1	1	2	12.9	27.3	147	8.60	40.9
1	3	0	0	2	56.5	106.8	237.4	13.88	162
1	2	0	1	3	111.2	157.6	17.2	1.01	11.3
1	2	0	0	3	29.9	40.4	23.5	1.37	13

0	0	1	0	2	273.6	165.2	554	32.40	365.7
1	1	0	0	2	390.2	547.7	72.2	4.22	52.1
1	2	0	1	2	77	96.5	703.8	41.16	396.4
1	2	1	0	3	222.9	383	523.9	30.64	389.5
1	2	0	0	2	146.9	275.8	775.6	45.36	469.4
1	1	0	1	2	17	14.3	58.9	3.44	31.8
1	3	1	0	2	776.9	867.9	78.3	4.58	57.3
1	2	0	0	3	43	188.8	606.5	35.47	368.9
1	2	0	1	2	25.2	41.9	111.4	6.51	42.3
0	0	0	0	2	2026.7	892	324	18.95	242.7
1	2	0	0	2	243	277.6	19	1.11	11.5
1	1	0	0	2	11.5	20.9	14.5	0.85	5.1
0	0	0	1	2	45.1	37	17.1	1.00	6.6
1	1	0	0	2	22.4	30.7	115.4	6.75	86.1
0	0	0	0	4	652.8	1548.5	53.3	3.12	37.7
1	1	0	0	2	15.4	46.6	149.4	8.74	120.7
1	1	0	0	2	60.4	137.9	466.4	27.27	321.9
1	1	0	1	4	22.3	42.6	124.1	7.26	102.8
1	1	1	1	2	19.8	39	10.9	0.64	8.2
1	1	0	0	2	66.6	41.7	272.5	15.94	190.4
1	1	0	0	2	99	95.5	252.2	14.75	203.2
1	1	0	1	2	23.3	43.9	55.6	3.25	15.9
1	1	1	0	3	19.1	33.7	45.4	2.65	24.2
1	1	0	0	4	25.8	33.8	52	3.04	17.6
1	1	0	1	2	17.4	29.6	30.2	1.77	13.8
1	1	1	0	2	52.2	103.1	433.5	25.35	347.4
1	1	0	1	2	75.1	109.8	39.3	2.30	19.4
1	1	0	0	2	38.1	74.6	156.7	9.16	83.4
0	0	0	0	2	21.6	66.8	75.3	4.40	33.2
1	1	0	0	2	204.5	341.2	560.3	32.77	378.7
0	0	0	0	3	36.4	57.6	92.1	5.39	42.1
0	0	0	0	2	198.4	124.3	203.1	11.88	133.2
0	0	1	1	2	19.2	19.1	35.5	2.08	14
1	1	1	0	2	43.8	71.9	19.9	1.16	11.5
1	1	0	0	2	7.5	16.8	17.5	1.02	11.4
0	0	0	0	2	30.7	63.8	23.2	1.36	16.7
0	0	0	0	3	19.1	33.9	52.1	3.05	27.2
1	1	0	0	2	11.3	25.2	74	4.33	51

1	1	1	0	2	486	633.2	294.9	17.25	202.3
1	1	0	0	2	25.8	36.3	49.5	2.89	23
1	1	0	0	2	37.9	116.6	205	11.99	164.5
0	0	0	1	2	30.1	126.5	55.2	3.23	36.5
1	1	0	0	2	2310.3	1482.4	338.5	19.80	252.7
1	1	0	0	3	25.5	80.5	827.3	48.38	578.9
1	1	1	0	2	11.6	24.7	43.7	2.56	21.6
1	1	0	1	2	151.8	259.5	490	28.65	340.4
1	1	1	0	2	22.8	75.6	40.7	2.38	24.5
1	1	0	1	2	10.6	21.4	11.8	0.69	5.4
1	1	0	0	2	11	18.8	22.1	1.29	11.8
1	1	1	1	2	46.9	94.1	32.1	1.88	20
1	1	0	0	2	17.8	56.2	116.7	6.82	78.7
1	1	1	0	2	11.9	26.7	14.4	0.84	5.6
1	1	0	0	4	15.8	25.2	29.3	1.71	13.6
1	1	0	0	2	31.5	29.3	37.1	2.17	24.8
0	0	0	0	2	22.8	26.1	34.1	1.99	14.3
1	1	0	0	2	15.6	22.1	22.8	1.33	12.3
0	0	0	0	2	34.2	55.7	16.4	0.96	8.4
1	1	0	0	2	47.9	104.4	46	2.69	24.5
1	1	0	0	2	7.4	18.8	54.5	3.19	26.6
1	1	0	0	2	128.9	143.4	552.2	32.29	384.9
1	1	0	0	2	22.5	36.4	105.1	6.15	53.4
1	1	1	0	2	131.6	134.1	146.1	8.54	118
0	0	0	1	2	53.8	72.6	23.9	1.40	11.5
1	1	0	1	2	19.1	20.3	13.6	0.80	5.6
1	1	1	1	3	5.7	13.5	25.8	1.51	14.3
1	1	1	1	4	8.4	16.5	12.9	0.75	10.7
1	1	0	0	2	50.2	91.1	133	7.78	322.5
1	1	0	0	4	28.3	65.6	34.8	2.04	21.1
1	1	0	1	2	20.4	34.5	17.8	1.04	8.7
0	0	0	0	2	4.5	19.2	12.8	0.75	7
1	1	0	0	2	23.4	39.4	99.5	5.82	66.2
1	1	0	0	2	67.4	111.6	46.7	2.73	32.7
1	1	0	0	2	34.1	59.4	45.1	2.64	21.6
1	1	0	1	2	5.3	30.5	47.5	2.78	17.5
1	1	1	0	2	231.7	288.5	401.1	23.46	216.3
1	1	1	1	3	8.6	22.4	34.7	2.03	27

1	1	0	0	2	27.3	41.6	32.1	1.88	16
1	1	1	0	2	17.2	27.6	15.8	0.92	7.9
1	1	0	0	2	4.8	8.4	2.9	0.17	1.2
1	1	1	1	2	28.2	27.9	106.7	6.24	81.5
1	1	0	0	3	29.7	57.4	199.7	11.68	167.5
0	0	1	0	3	20	56.5	83.3	4.87	58.5
1	1	0	0	2	27.2	47.5	46	2.69	27.7
0	0	0	0	2	19.7	38.6	68.2	3.99	35.7
1	1	1	0	2	21.2	36.8	87.5	5.12	41.2
1	1	1	0	2	28.8	68.4	48.2	2.82	31.8
1	1	0	0	2	1290.6	1937.3	327.4	19.15	208.1
1	1	0	0	3	19.2	36.7	230.3	13.47	161.7
1	1	1	0	2	119.5	179.3	89.3	5.22	59.2
0	0	1	1	2	17.9	33.8	62.3	3.64	43.2
1	1	0	0	2	43	38.5	32.1	1.88	15.3
1	1	0	0	2	17.7	30.2	113.2	6.62	49
0	0	0	0	2	29.8	45.8	36.4	2.13	25.5
1	1	0	0	2	15	27.6	49.2	2.88	22.4
1	1	0	0	2	11.7	16.2	15.8	0.92	7.6
0	0	0	1	2	16.7	20.7	101.8	5.95	18.9
0	0	0	0	2	7.8	14.3	17.2	1.01	7.5
0	0	1	0	2	10.8	24.6	30.5	1.78	15.1
1	1	1	0	2	36.1	112	124.2	7.26	102.1
1	1	0	0	2	23.4	38.7	144.4	8.44	130.6
0	0	1	1	2	14.2	16.5	13.2	0.77	6.5
1	1	0	0	2	14.5	41.9	321.2	18.78	255.3
1	1	1	1	2	27.2	38.5	28.6	1.67	16.2
0	0	0	1	2	56.8	51.7	99.9	5.84	87.3
1	1	0	0	3	27.4	26.2	12.5	0.73	6.8
1	1	1	1	2	14.8	42.7	44	2.57	27.3
1	1	0	0	3	228.4	426.8	196.9	11.51	143.2
1	1	1	1	2	9.3	45.4	51.1	2.99	37.6
1	1	0	1	2	8.6	17	72.8	4.26	62.4
1	1	0	0	2	10.5	31.5	46.4	2.71	37.5
1	1	0	0	2	45.9	68.2	174.5	10.20	120.5
0	0	1	0	2	10.9	21.5	17.7	1.04	11.6
1	1	0	0	2	96.2	169.8	522.8	30.57	325.4
0	0	0	0	3	7.2	16	59.8	3.50	11.9

1	1	1	0	2	10.9	27.6	23.9	1.40	15.3
1	1	1	0	2	58.6	65.9	93.1	5.44	33.6
1	1	0	0	2	24.3	24.9	16.8	0.98	9.5
0	0	0	0	2	20.1	47.2	100.4	5.87	54.7
1	1	0	0	2	46.1	52.4	343	20.06	227
1	1	0	0	2	38	64.3	146.1	8.54	100.6
1	1	0	0	3	34.7	56.7	65.1	3.81	48.1
1	1	0	1	2	23.9	45.6	26.4	1.54	11.7
1	1	0	0	2	21.5	29.4	115.3	6.74	100.4
1	1	0	0	2	32.3	51.4	521.3	30.49	400.1
1	1	0	0	2	12.5	23.7	29.5	1.73	13.6
1	1	1	1	2	59.2	72.1	90.6	5.30	55.4
1	1	1	1	2	43.5	63.9	42.8	2.50	22.3
1	1	0	0	4	58.1	61.1	136.7	7.99	99.6
1	1	1	0	2	44.6	135.8	355.9	20.81	215.8
1	1	1	0	3	57	108.6	64.4	3.77	34.7
1	1	1	0	3	41.1	30.7	24.2	1.42	13.5
1	1	0	1	3	36.5	46.9	161.3	9.43	95.1
1	1	0	0	2	15.9	42.9	136.6	7.99	72.5
0	0	1	1	2	20.6	28	25.4	1.49	10.6
0	0	1	1	2	9	14.4	33.3	1.95	20.3
1	1	0	0	2	8.7	31.6	16.5	0.96	8.8
1	1	0	1	3	15.7	14.4	22.4	1.31	12.6
1	1	0	0	3	139.3	284.2	437	25.56	299
1	1	0	0	2	19.8	34.3	20.6	1.20	14.2
0	0	0	1	2	82	109.2	40.3	2.36	18.2
1	1	1	1	3	52	38.9	81.4	4.76	51.4
1	1	0	0	2	137.4	560.8	339.3	19.84	265.9
1	1	1	1	2	39.7	37.7	4.8	0.28	2.3
1	1	0	1	4	18.5	34.5	28	1.64	16.7
0	0	0	1	3	28.7	44.3	222.4	13.01	103.7
0	0	0	0	2	28.6	44.6	44.9	2.63	24.3
1	1	1	0	2	207.8	281.1	324.5	18.98	252.9
0	0	0	0	2	34.4	63.2	61.7	3.61	26.4
1	1	0	0	2	10.9	18.1	44.8	2.62	19
0	0	0	0	2	155.8	360.1	507.1	29.65	354.3
1	1	0	0	2	32.8	51.4	150.9	8.82	94.4
1	1	0	1	3	329.6	685.9	433.9	25.37	325.5

0	0	0	0	2	21.1	49.7	32.6	1.91	16.6
1	1	1	1	2	7	30.7	18.5	1.08	11.2
1	1	0	1	2	13.5	20.2	19.6	1.15	9.9
0	0	1	1	2	43	64.7	432	25.26	266.3
0	0	0	0	3	52	58	119.6	6.99	49.6
1	1	0	0	3	22.8	51.3	48.4	2.83	19.8
1	1	0	0	2	25.4	43.3	33.6	1.96	21.3
1	1	0	0	3	17.6	25.5	57.9	3.39	42.9
1	1	1	1	2	8.1	24.4	22.5	1.32	13.4
1	1	0	1	2	172	225.8	96.6	5.65	69.2
0	0	1	0	3	26.7	58.4	57.4	3.36	47.2
1	1	0	0	2	54.6	58.6	78.7	4.60	59.1
1	1	0	0	2	18.9	79.8	103	6.02	90.4
0	0	0	0	2	24	33.1	86.4	5.05	38.5
1	1	1	0	2	42.9	85.2	163.2	9.54	145.1
1	1	1	1	3	50.9	55.1	24.2	1.42	17.2
1	1	0	0	2	37.8	73.4	202.7	11.85	167.2
1	1	0	0	2	26.4	88.2	165.5	9.68	142.3
1	1	0	0	3	31	23	32.8	1.92	17.4
0	0	0	0	2	11.7	19.8	10.3	0.60	6.2
1	1	0	0	2	53.4	91.6	306.6	17.93	223.1
0	0	1	1	3	30.3	42.7	37.8	2.21	22.7
1	1	1	1	2	13.6	28.3	23.4	1.37	13.8
1	1	0	0	2	22.9	31	50.8	2.97	32.9
1	1	0	0	2	39.2	52.8	19.5	1.14	11.7
1	1	0	0	3	19.9	40.7	31.5	1.84	15.2
1	1	1	0	2	25.7	49.4	21.9	1.28	12.4
0	0	0	0	2	18.1	21	26.4	1.54	11.8
1	1	1	0	3	32.7	37.3	26.9	1.57	15
1	1	0	0	2	14.3	16.1	118.3	6.92	60.8
1	1	0	0	3	34.5	39.1	64.2	3.75	24.8
1	1	0	0	3	29.2	39.2	128.5	7.51	32.9
1	1	0	0	2	36.5	38.1	67.6	3.95	39.3
1	1	0	0	3	31	43	43.8	2.56	16.9
1	1	0	0	2	36.3	62.7	38.3	2.24	22.2
0	0	1	0	3	20.1	51.4	25	1.46	16.2
0	0	0	0	2	18.4	28	10.5	0.61	5.4
1	1	0	1	2	16.3	22.6	52.4	3.06	32.4

0	0	0	0	2	82.1	41	53	3.10	33.5
0	0	0	0	2	10.9	16.8	10.3	0.60	5.5
0	0	1	0	2	16	44.5	35.5	2.08	16.9
1	1	0	0	3	15.7	15.8	21.9	1.28	11.9
1	1	0	1	2	3	11.3	9.3	0.54	4
1	1	0	0	2	17.8	34.5	55.5	3.25	14.8
1	1	0	1	2	31.2	49.3	83.9	4.91	58.4
1	1	1	1	3	21.1	23.6	6.4	0.37	3.2
1	1	0	1	2	35.5	52.8	47.6	2.78	25.6
1	1	0	0	2	16.2	14.7	8.9	0.52	4.5
1	1	0	0	2	23	26.8	79.8	4.67	29.5
1	1	0	0	2	10.6	16.9	12.8	0.75	6.1
1	1	0	0	2	23.8	33.2	41.3	2.42	26.9
1	1	1	1	4	13.7	49.2	23	1.35	11.4
0	0	0	1	2	32.1	48.9	61.6	3.60	27.9
1	1	0	0	2	29.6	37.1	30	1.75	5.8
1	1	0	1	2	17.8	21.1	21.4	1.25	8.2
1	1	0	0	2	34.3	58.1	54.9	3.21	33
1	1	1	0	2	48.2	62.4	104.6	6.12	77.3
1	1	0	1	2	7.8	9.7	13.9	0.81	5.9
1	1	0	0	4	42.9	59.3	80.5	4.71	44.7
1	1	0	0	2	174.8	365.5	17.4	1.02	8
0	0	0	0	3	11.7	14.5	39.8	2.33	15.6
1	1	1	0	2	54.4	153.1	41	2.40	27.8
1	1	0	1	2	140.5	139	7.2	0.42	4.4
0	0	0	0	2	11.2	29.5	91.4	5.35	55.2
0	0	1	0	2	19.9	39	41.3	2.42	23.8
1	1	0	0	2	39.1	39.3	42.3	2.47	23
1	1	0	0	3	49.3	103.5	38.2	2.23	33.7
1	1	0	0	2	22.8	37.7	8.1	0.47	4.5
1	1	0	0	2	17.3	32.8	17.6	1.03	7.8
0	0	1	0	2	21.2	39.9	47.5	2.78	24.6
1	1	0	0	2	6.2	21.8	6.6	0.39	2.9
1	1	0	1	4	16.6	20.7	33.8	1.98	13.9
0	0	0	0	3	46.3	63.7	29	1.70	11.6
1	1	0	0	2	60.2	104.2	60.4	3.53	42.3
1	1	1	1	2	14	20.2	21	1.23	10
1	1	0	0	2	20.3	29.3	68.5	4.01	34.3

0	0	1	1	2	34	24.2	45.5	2.66	33.5
0	0	0	0	3	33.7	26	21.2	1.24	12.2
1	1	0	0	2	105.4	162.6	46	2.69	29
1	1	0	1	3	39.4	53.4	27.9	1.63	6.9
1	1	0	0	2	29.8	38.5	35.8	2.09	20.2
1	1	0	0	2	23.7	37.8	30.4	1.78	17.8
0	0	1	1	2	6.9	13.4	18.7	1.09	10.1
1	1	0	1	2	14.2	35	27.1	1.58	13.6
0	0	0	0	4	38	39	94.9	5.55	44.3
0	0	0	0	2	47.2	56.6	47.8	2.80	22.2
1	1	1	0	2	16.8	44.1	148.3	8.67	121
1	1	0	0	2	25	30.8	10.2	0.60	5.3
1	1	1	1	3	23.2	39.4	27.1	1.58	12
1	1	0	0	2	23	41.5	67.6	3.95	41.2
1	1	1	0	2	45.9	86.7	24.9	1.46	18.7
1	1	0	0	2	18.1	41.8	11.1	0.65	5.2
0	0	0	0	3	66.9	63.2	67	3.92	23.2
0	0	0	0	2	119.4	103.4	9.9	0.58	4.5
0	0	1	0	4	4.9	30.8	39.7	2.32	19.8
0	0	1	0	4	34	58.8	15.4	0.90	6.1
1	1	0	0	2	8.3	9.4	12.2	0.71	6.5
0	0	1	0	2	39.1	54.1	63.4	3.71	25.4
1	1	1	0	2	389.8	399	300.6	17.58	229.2
1	1	0	0	3	8.7	12.9	22.4	1.31	11.9
1	1	0	0	2	39	53.4	6	0.35	2.7
1	1	1	0	2	60.9	80.2	14.2	0.83	6.1
1	1	0	0	2	34.3	42.3	50.9	2.98	24.7
0	0	1	0	2	17.5	28.9	16.9	0.99	8.1
1	1	0	0	2	21	42.1	18.4	1.08	11.7
0	0	0	1	2	121	115.7	26.1	1.53	12.8
0	0	1	0	2	7.6	20.7	15.2	0.89	9.3
1	1	1	1	2	17.6	32.1	82	4.80	41.8
1	1	0	0	2	28.6	36.3	49.1	2.87	23.1
1	1	0	0	3	50.6	72.2	36.9	2.16	24.7
1	1	1	1	2	12.7	28.6	33.4	1.95	24.9
1	1	0	0	2	79.6	89.4	250.6	14.65	206.8
0	0	0	1	2	18.6	25.3	31.2	1.82	16.8
1	1	0	1	3	22.9	30.1	35.8	2.09	17.7

1	1	1	0	2	29.3	114.4	94.3	5.51	73.8
0	0	0	1	2	13.2	18.2	6.5	0.38	3.7
0	0	1	0	2	30.8	47.6	31	1.81	14.7
0	0	1	0	2	1560.1	1670.2	91.1	5.33	71.4
0	0	1	0	3	17.4	27.5	26.3	1.54	13.3
1	1	0	0	3	16.2	15.7	8.3	0.49	5.2
1	1	0	0	2	33.4	41.6	47.2	2.76	25.6
1	1	0	1	3	18.6	26.3	70.8	4.14	54.7
1	1	0	0	2	26.9	33.3	44.2	2.58	25.7
0	0	0	0	3	10.7	37.2	50.4	2.95	11
0	0	0	0	2	48.2	70.7	33.5	1.96	15.4
0	0	0	1	2	25.1	26.1	17.7	1.04	6.1
0	0	1	0	3	69.7	80.6	20.1	1.18	11.9
0	0	0	1	2	30	36.3	47.6	2.78	25.8
0	0	1	0	2	24.4	39.4	27	1.58	9
1	1	0	1	2	7.8	12.3	46.8	2.74	32.4
1	1	0	0	2	29.2	36.7	70	4.09	11.4
1	1	0	0	2	51.7	57.8	118.2	6.91	42.4
0	0	1	0	2	39.4	72.5	69	4.04	29.5
1	1	0	0	2	17.9	19.3	14	0.82	6.8
1	1	1	0	3	10.8	17	14.6	0.85	4.8
1	1	0	0	2	22.1	45.6	54.5	3.19	40.7
1	1	0	0	2	67	93.5	173.7	10.16	137.4
0	0	0	0	2	115.2	34.2	17.1	1.00	8.4
1	1	1	1	2	33	36	39.3	2.30	14.4
0	0	0	0	2	23.1	34.8	28.6	1.67	12.9
0	0	0	0	2	20	32.8	33.7	1.97	15.5
1	1	0	0	2	24.8	56.8	104.8	6.13	58.3
1	1	1	1	2	16	20.3	13	0.76	3.6
1	1	0	0	4	11.5	19.1	25.1	1.47	12.1
1	1	0	0	2	24.9	44.6	48.6	2.84	25.5
1	1	0	0	2	33.5	48	28.3	1.65	11.3
1	1	0	0	3	27.5	44.8	50.5	2.95	29.6
0	0	0	0	2	21.1	49.6	12.3	0.72	4.5
1	1	0	0	2	13.9	39.9	16.3	0.95	9.4
1	1	0	0	2	17.9	56.2	79.9	4.67	33.4
1	1	0	1	2	19.9	39.1	19.3	1.13	9.3
0	0	0	0	2	19.5	31.3	32.9	1.92	14.4

0	0	1	0	2	7.3	34.3	19.7	1.15	12.5
0	0	0	0	2	12.2	19.5	3.3	0.19	22.9
1	1	0	0	3	61.9	90.9	31.7	1.85	15.4
1	1	0	0	4	11.7	12.5	16.1	0.94	7.5
0	0	0	0	3	44	85.9	76.3	4.46	33.1
1	1	1	1	2	17.6	21	17	0.99	9.1
0	0	1	0	2	92.6	119	18.8	1.10	7.4
0	0	0	0	2	19.1	16.7	14	0.82	4
1	1	0	0	2	9	14.2	11.5	0.67	5
1	1	0	1	2	17.3	25.4	54.1	3.16	21.6
0	0	0	0	3	13.1	61.2	58.5	3.42	35.9
0	0	0	1	2	30.4	34	23.9	1.40	15.4
0	0	0	1	3	18.4	33.8	24.2	1.42	9.2
1	1	0	0	2	27	61.2	20.1	1.18	9.3
1	1	0	1	3	18.4	23	18.3	1.07	6.9
1	1	0	0	2	11	9.8	31.1	1.82	17.4
0	0	0	0	2	24.5	37.7	54.5	3.19	36.6
1	1	0	0	2	151.1	329.8	165.4	9.67	92.7
1	1	0	0	2	19	28.5	15.8	0.92	8.1
1	1	0	0	2	36.4	47.4	25.6	1.50	13.5
1	1	1	0	2	2204.6	756.7	269.5	15.76	181.9
1	1	0	0	2	55	208.1	343.5	20.09	219.7
1	1	0	0	2	12.5	49.1	106.4	6.22	90.3
1	1	1	0	2	58.8	70.6	234.6	13.72	169.6
0	0	0	0	2	60.5	190.4	158.3	9.26	104.6
1	1	0	0	3	34.3	45.9	286.3	16.74	208.6
1	1	0	0	2	93.2	139	418.3	24.46	315.1
1	1	0	0	2	78.7	123.5	576.3	33.70	413
1	1	0	0	3	25	47.5	21.9	1.28	15.3
0	0	0	0	2	375.6	243.1	285.2	16.68	196.1
1	1	0	0	2	90	228.7	296.7	17.35	79.6
1	1	0	0	2	448.5	433.1	419.1	24.51	321.5
1	1	0	0	2	56.6	98.4	728.7	42.61	550
1	1	0	0	2	24.1	39.9	464.9	27.19	323.4
1	1	0	0	2	14.2	26.4	13.1	0.77	8.1
1	1	0	1	2	17	14.3	58.9	3.44	31.8
1	1	0	0	2	11.5	20.9	14.5	0.85	5.1

**1DBIL(n**	ALB	GLO	GGT	ALP	TC	TG	HDL	NHR	LHR
2.42	26.2	28.7	629.6	92.3	3.72	1.98	0.54	24.07	2.41
1.14	22.5	31.1	13.5	89.2	3.98	0.65	0.74	6.62	1.08
0.75	12.7	9.7	13.1	24.2	0.96	0.34	0.3	150	2.33
2.20	9.6	17.4	3.8	25.8	0.91	4.37	0.15	14.67	21.47
0.27	12.6	7.4	6.9	19.2	0.52	0.08	0.24	25.5	13.17
2.59	23	29.4	15.4	43.5	1.14	0.15	0.29	32.21	4.69
9.48	25.7	27.2	47.85	99	2.4	0.56	0.49	22.22	3.88
0.71	14.7	12	47.85	99	2.4	0.56	0.49	35.14	2.94
0.22	25.1	31	47.85	99	2.4	0.56	0.49	11.16	3.2
3.80	29.7	29.7	348.8	49.5	3.18	7.05	0.09	45.67	7.44
11.78	19.1	23.2	47.85	99	2.4	0.56	0.09	14.67	7.22
13.66	24.3	23.9	47.85	99	2.4	0.56	0.49	15.86	4.04
9.16	21.1	25.4	47.85	99	2.4	0.56	0.49	40.88	1.43
0.88	27.4	14.4	47.85	99	2.4	0.56	0.49	17.98	3.94
10.78	23.2	13.9	47.85	99	2.4	0.56	0.49	15.12	1.1
4.59	23.6	26.9	47.85	99	2.4	0.56	0.49	17.84	6.55
0.26	28.5	20.3	35.6	61.6	2.95	0.7	0.63	12.73	0.83
16.18	40.7	17.8	47.85	99	2.4	0.56	0.49	21.27	1.2
18.82	29.6	41.3	16.2	142.2	2.4	0.56	0.09	90	1.11
6.16	18.1	28.7	47.85	99	2.4	0.56	0.49	26.12	2.45
2.02	11.3	31.1	6.9	51.2	0.72	0.1	0.08	45	11.25
4.05	21.8	29.7	198.2	356.4	3.11	1.15	0.11	56.36	6.36
14.14	32	32.8	357.8	92.3	2.02	0.69	0.15	87.4	2.67
0.67	28	25.6	207.2	212.3	2.67	1.22	0.25	16.28	0.88
7.60	17.1	27.6	90.3	281	0.3	0.23	0.13	77.15	12.85
0.48	29	18	71.2	109.1	0.51	0.22	0.17	30.59	3.06
22.77	32.7	15.4	135.3	203.4	1.35	0.54	0.14	97.8	34.43
11.13	28.1	19.7	47.85	99	2.4	0.56	0.49	12.59	1.37
7.14	26.7	23.6	88.6	111.9	1.6	0.65	0.12	87.6	8.33
8.80	23.1	28.1	8.6	77.9	1.75	0.3	0.15	46.6	4.8
4.91	22.6	26.5	251.3	60.6	2.58	0.75	0.27	24.78	5.37
14.19	29	33.8	1407.9	939.8	9.03	2.44	0.25	14.76	11.16
16.25	41	26.8	74.2	207.5	2.61	0.67	0.28	9.68	2.61
7.30	23.3	26.3	17.7	107.7	0.92	0.32	0.32	14	0.78
0.46	33.1	30.1	13.4	61	3.14	0.43	1.51	0.62	0.34
1.35	19.2	28.3	43.9	175.1	1.38	0.32	0.45	2	2.67
12.76	29.9	23.9	202.5	186.7	2.89	1.15	0.33	9.39	4.85

16.02	30.6	46.1	70.8	209.7	2.4	0.56	0.49	7.14	1.63
2.99	27.2	34.8	179.4	124.6	2.23	1.23	0.28	20.36	6.79
13.72	27.5	32.3	105.8	154.5	2.36	0.45	0.12	85.83	12.5
0.61	35.5	29.7	47.85	99	2.4	0.56	0.49	6.53	1.22
2.23	23.6	16.6	64.3	151.9	0.41	0.15	0.12	79.17	5.83
3.70	31.7	40.2	305.5	116.4	2.18	0.9	0.56	8.21	2.5
20.00	28.1	23.4	26.9	74.2	1.06	0.44	0.17	88.47	4.18
17.53	22.4	27	18.2	100.9	1.11	0.5	0.19	41.58	1.58
20.32	25.6	29	88	127.7	2.61	1.18	0.12	60.42	11.75
1.32	35.2	36.8	47.85	99	2.4	0.56	0.49	26.33	1.51
16.89	22.7	44.5	21.2	73.1	1.34	0.41	0.21	24.29	2.14
1.63	29.4	36.2	44.8	73.6	2.17	0.25	1.11	3.78	1.26
3.44	25.3	29.7	47.85	99	2.4	0.56	0.49	28.37	1.84
17.59	21.8	41.8	11	96.5	1.17	0.44	0.36	34.44	4.44
17.09	24.6	13	25.5	73.5	0.86	0.43	0.14	46.43	5
6.27	20.2	36.4	43.3	107.8	1.35	0.79	0.2	16	4.5
13.29	25.5	47.3	144.1	195.6	0.96	0.36	0.39	40.08	1.62
16.53	15.4	36.3	47.85	99	2.4	0.56	0.49	13.88	0.82
1.78	18.1	39.9	62.7	114.7	1.96	0.49	0.71	3.8	2.82
16.80	19.7	27	19.2	25.4	0.84	0.3	0.27	26.81	1.33
5.28	22.4	29.5	74.3	142.4	1.78	0.42	0.42	11.43	0.48
1.13	20.3	40.9	40.8	103.6	3.01	0.63	1.28	3.2	0.94
16.50	14.8	16.3	17.1	37.7	0.77	0.49	0.19	18.95	0.89
1.94	25.2	40.9	25.8	158.4	2.79	1.18	0.62	4.84	0.48
4.65	30.8	29.7	47.85	99	2.4	0.56	0.19	18.95	2.11
0.89	27.1	29.4	20.8	174.5	2.62	0.61	1.37	1.68	0.44
20.42	20	29.8	23.3	88.4	0.93	0.42	0.21	61.43	5.24
25.71	34	28	57.9	29.7	1.58	0.59	0.29	15.86	1.72
11.60	18.2	24.5	14	58.5	1.72	0.36	0.51	89	0.98
1.53	25.4	23.7	80.4	98.6	0.83	0.08	0.52	4.42	0.96
0.49	23.8	29.3	70.4	195.4	1.9	0.8	0.59	1.86	0.51
19.58	21	51.4	103.3	191.5	2.47	1.68	0.11	93.6	10
12.65	21.8	30.4	473.9	144.7	4.42	3.2	0.12	78.17	12.58
16.40	30.2	34	22.5	202.4	1.34	0.48	0.1	43	13
1.58	27.3	18.7	7.6	48.9	1.24	0.4	0.43	36.98	1.16
0.29	31.7	36.8	97.9	118	2.59	0.81	0.74	4.2	0.28
1.39	33.9	26.9	31.8	121.3	3.57	0.66	1.58	2.26	0.93
17.95	31.4	32.1	98.3	250.9	3.52	3.33	0.15	31.33	4

0.92	29	26.9	26	88.4	3.31	0.59	1.26	1.33	0.34
0.82	32.2	34.5	35.4	71	4.69	1.21	0.69	6.77	1.55
11.29	29.1	42.5	118.8	197	1.52	0.47	0.11	81.82	11.82
0.90	32.8	34.3	38.3	94.3	2.87	0.6	1.09	2.18	0.58
1.14	18.7	48	51	135.1	1.99	0.63	0.78	1.74	0.38
1.67	20.5	22.3	80.2	137.6	1.34	0.19	0.49	11.33	0.49
1.33	24.1	27.9	61.3	61.6	1.86	0.2	0.6	2.38	0.67
0.40	39	36.4	38.8	102.9	3.62	0.95	1.45	2.15	0.31
22.85	34.5	32.1	29.8	124.1	1.21	0.54	0.06	65.1	19.67
2.98	34.2	30.4	21.1	68.6	2.4	0.56	0.49	5.16	1.86
0.78	22.6	11.8	9.3	29.7	2.4	0.56	0.49	17.22	1.8
1.47	32	31.6	22	81.9	3.71	0.77	1.08	1.19	0.63
29.19	31.8	12.8	129.7	99.1	2	0.6	0.14	82.14	3.86
1.02	28.7	16.3	33.5	40.5	1.69	0.62	0.53	7.21	3.6
7.15	24.3	40.2	40.4	26	0.91	0.38	0.09	49.11	5.33
1.47	19.8	37.8	25	100.8	3.16	0.48	0.42	21.02	1.52
9.46	33	30.8	95.4	124.1	1.3	0.39	0.11	47.45	17.55
20.30	34	39.4	46.6	137	1.59	0.53	0.17	28	8.06
29.92	32.9	35.2	75.9	147	1.65	0.67	0.14	31.07	6.86
2.75	28.3	23.7	16.7	220.3	2.1	0.53	0.38	4.95	1.13
0.53	35.1	29.8	46.9	141.7	2.92	0.84	1.01	2.22	0.57
17.23	32.5	24	69.7	141.8	1.47	0.43	0.11	63.73	13.36
22.60	28.5	26.2	23.6	88.7	1.28	1.11	0.04	52.2	19.25
4.55	21.2	45.7	36	35	2.53	6.88	0.15	20.53	3.67
2.06	20.2	24.7	41.8	51.9	2.53	0.12	0.22	45.27	3.23
1.90	26.4	26.8	648.3	91.7	2.79	0.76	0.42	17.31	4
3.58	25.9	41.5	47.85	99	2.4	0.56	0.49	12.16	1.49
1.26	29.5	40	47.85	99	2.4	0.56	0.49	10.29	1.31
10.58	25.4	31.2	79.5	76.7	1.37	0.6	0.12	30.5	9.25
0.69	28.3	34.1	34.5	51.2	2.35	0.77	0.54	9.06	1.41
0.63	28	32.5	38	82.2	5.01	0.46	1.95	0.68	0.65
3.60	25.1	39.4	47.85	99	2.4	0.56	0.49	8.47	1.57
2.19	25.4	47.7	47.85	99	2.4	0.56	0.49	10.92	4.29
14.78	35.3	20.8	162	132	3.41	0.7	0.21	28.57	2.1
4.87	23.5	42.6	180.7	81.7	1.74	1.39	0.16	30.06	7.81
4.70	20.2	33.7	16.5	120.7	2.17	0.47	0.26	17.12	9.65
33.85	33.5	26.3	8.1	12.3	2.13	1.08	0.02	63.5	145.5
10.97	24.1	38.5	102	109.8	1.61	0.46	0.11	22.73	12.91

1.99	22.5	39.4	34.5	96	2.12	0.75	0.27	7.85	5.44
2.54	22	45.1	28.2	99	2.37	0.66	0.18	13.39	10.5
2.20	17.8	40.7	29.5	85.7	2.8	0.47	0.22	13.05	4.64
0.46	23.1	27.5	50.8	103	3.01	0.34	1.14	3.29	0.91
15.75	26.8	48.6	44.1	114.5	1.11	0.47	0.09	58.78	28.89
1.26	25.9	24.9	19.6	79.1	2.19	0.51	0.85	1.71	0.56
19.91	30.5	35.4	171.4	213.1	1.97	0.45	0.13	86.92	7.85
24.15	26.2	17.9	46.6	83.9	1.76	0.57	0.14	34.43	6.5
3.23	40.3	28.4	40.2	145.1	2.94	1.01	0.81	4.43	1.9
3.04	34.7	44.5	156.1	210	5.75	0.86	1.08	3.67	0.15
0.91	32.6	32.5	29.4	71	2.45	0.52	1.07	1.42	0.93
0.23	30.5	22.2	28.9	57.8	2.37	0.87	0.64	130	2.81
0.70	27.6	34.9	96.6	109.1	2.07	1.86	0.23	10.3	6.22
0.33	32.2	20.4	47.85	99	2.4	0.56	0.49	67	2.1
24.54	31.3	23	143.6	99.9	2.06	0.51	0.14	27	5.21
0.39	18.6	19.8	12.4	33.6	1.49	0.65	0.32	5.22	1.53
0.42	34.2	31.5	25.6	112.1	4.19	0.7	1.18	4.37	2.07
1.38	25.2	35.7	43.7	102.4	2.04	0.27	0.72	3.51	2.01
2.20	29.9	34.2	72.2	96.4	5.46	1.96	0.76	3.61	1.57
1.16	27.2	37.4	29.5	141.1	2.36	0.72	0.85	1.93	1.41
10.84	22.2	48.9	322.1	67.5	1.49	0.5	0.19	54.58	8.26
0.17	22	31.8	33.2	75.7	5.79	0.73	1.47	2.05	1.85
18.71	29.1	38.7	26	133.8	1.25	0.48	0.09	38.33	15.11
19.61	35.7	34.3	170	146.5	2.81	0.95	0.16	36.56	7.19
4.01	29.1	34.9	55.9	199.4	3.41	1.25	0.68	3.07	2.84
1.64	27.3	26.6	16.2	68.4	2.09	0.59	0.59	3	1.32
14.87	31	30.9	112.2	157.5	1.61	0.46	0.24	22.63	6.96
0.61	26.5	36.4	77.1	196.5	2.76	1.86	0.64	2.19	1.83
1.18	27.6	32.5	22.9	77	1.7	0.57	0.48	6.06	1.67
24.53	36.7	46.1	27.9	91.3	1.47	0.61	0.12	32	5.08
1.12	29.8	19.3	62.9	241.8	1.42	0.23	0.56	12.61	1.32
14.20	24	16.2	155.6	94.1	5.43	0.53	0.21	81.67	4.24
4.57	31.8	29.5	150.5	15.6	1.09	0.9	0.14	63.29	5.71
5.30	29.2	30.5	161.8	54	2.19	2.48	0.1	81.2	8.7
6.51	25.6	26	19.7	83.8	1.18	0.65	0.08	80	13.88
4.90	26.4	19.6	47.85	99	2.4	0.56	0.49	5.14	1
1.43	28	41.8	27.4	105.1	2.81	0.67	0.67	1.9	1.01
19.96	29.2	30.3	149.1	164.6	1.7	0.85	0.12	34.83	5.08

1.56	28.4	40.4	3.5	68.2	3.1	0.6	0.48	7.35	0.54
22.51	26.1	31	23.5	53.8	1.44	0.43	0.11	27.27	5.36
0.36	27.7	33.7	56.4	69.6	2.69	0.74	0.97	3.84	0.9
16.10	26.1	18.9	46.8	103.5	1.09	0.32	0.09	57	3.11
14.49	31.6	38.3	27.8	67	0.99	0.39	0.08	81	27.63
0.28	28.9	42.1	24.4	56.4	3.63	0.74	0.81	5.91	0.75
0.30	29.7	45.7	22.5	92	3.98	0.53	1.08	3.44	1.56
2.36	30.7	26.5	41.4	92.9	4.56	0.74	1.2	3.59	2.44
0.06	24.7	45.4	41.8	55.8	5.9	2.1	0.62	6	1.42
0.99	23.8	19.9	9.4	47.2	1.68	0.38	0.52	4.44	1.21
1.17	21.7	33.3	47.6	178.6	2.85	0.48	1.03	2.74	1.13
0.33	30.6	32.6	37.1	85.2	2.4	0.56	0.49	15.16	4.63
0.61	28.8	41	16.3	107	4.43	0.5	1.5	1.08	0.73
4.73	25.4	22.8	50.7	40.4	0.7	0.22	0.12	14.08	7
10.13	34.7	25.2	148.2	153.1	2.29	0.32	0.29	36.14	2.69
18.24	28	18.9	57.9	87.1	1.4	0.38	0.1	71.7	13.4
0.84	28.5	20.2	20.4	70.4	4.26	2.6	0.32	22.19	1.91
0.77	35	22.3	92.2	94.3	3.49	0.7	0.91	9.76	0.82
0.27	34.6	23.8	56.9	84	2.75	0.95	1.13	0.35	0.39
15.55	25.4	29.4	250.6	204.8	2.18	0.82	0.14	44.21	8.79
1.27	27.7	32.3	28.4	155.4	2.2	1.32	0.68	2.13	1.21
0.63	29	43.5	145.7	105.4	1.78	1.64	0.15	6.4	6
11.87	23.9	44.4	52.9	103.2	1.5	0.34	0.11	84.91	10.18
0.49	31.1	25.8	22.1	56.2	2.4	0.56	0.49	3.43	0.98
18.86	29.5	17.6	166.4	175.4	1.39	0.39	0.1	91	3.8
0.73	20.5	35.1	67.4	122.3	3.21	1.98	0.38	12.76	1.76
13.98	30.9	22.8	82.8	143.5	1.94	0.5	0.15	37.87	9.53
5.29	20.8	38.3	26.3	101.9	2.56	0.5	0.23	34.74	6.17
1.23	21.4	22.7	132.1	146.4	2.72	0.16	0.64	7.7	0.97
20.82	31.4	22.6	38.8	98.4	1.95	0.43	0.13	31.15	3.38
10.10	27	28.5	985.5	76.2	2.69	1.6	0.16	29.38	6.38
5.77	32.5	36.4	10.4	124.2	1.82	0.36	0.72	7.57	1.28
10.22	25.7	35.8	11.5	88.9	0.9	0.32	0.06	73.83	16.67
0.64	28.4	13.9	29.8	64.8	1.24	0.04	0.5	18.06	2.46
3.56	26.1	33.7	17.4	50.7	1.32	0.17	0.18	14.22	3.83
34.84	35.5	21.2	59.1	161	1.88	0.53	0.15	12.8	11.07
0.51	25.7	17.3	12.6	40.2	1.87	0.23	0.52	13.23	1.35
3.66	20.2	26.4	79.9	68.7	2.36	0.38	0.22	26.05	4.23

1.63	34.2	37.4	47.85	99	2.4	0.56	0.49	9.8	2.73
6.88	22.8	41.6	42.4	40.1	1.22	0.24	0.12	7.83	4.92
1.73	19.5	30.9	54.4	67.5	2.22	1.33	0.31	3.94	1.84
13.51	28.1	37.6	120.8	86.5	1.86	0.31	0.17	20.29	4.76
6.82	26.8	27.7	205.8	130	1.46	0.26	0.11	21.91	5.27
4.73	31.5	12.9	186.6	87.4	1.65	0.37	0.61	51.7	3.28
2.95	24.6	36.9	84.5	46.9	3.88	0.76	0.97	8.73	0.35
3.05	40.4	38.8	367.3	183.1	5.54	1.2	0.73	12.18	4.42
0.41	25.5	25.7	47.85	99	2.4	0.56	0.49	3.55	2.78
0.87	28.3	28	32.9	82	3.12	0.63	1.29	1.77	1.22
2.22	28.6	31.4	46.7	58.4	4.02	1.23	0.88	3.72	1.94
14.65	33.9	27.3	61.3	176.8	1.63	0.45	0.15	29.07	9.53
2.27	36.9	27.6	10.8	35.7	0.98	0.1	0.31	21.19	4.23
4.20	16.8	47.5	47.85	99	2.4	0.56	0.49	8.14	1.51
1.26	31.4	27.7	91.9	93	3.44	0.42	1	1.57	0.66
12.65	33.4	22.6	253.3	105.7	1.73	0.36	0.26	11.69	8
1.58	19.2	43.2	20.2	71	1.27	0.53	0.1	88	5.4
10.15	21.9	38.5	47.85	99	2.4	0.56	0.49	19.04	0.61
3.99	30.6	22.2	597.7	73.2	2.83	1.66	0.25	26.48	3.64
0.40	25.5	19.2	5.9	35.2	2.23	0.56	0.69	1.61	0.32
19.36	27.5	22.2	110.7	60.4	1.58	0.45	0.1	16.5	6.6
0.46	29.2	25	34.9	86.3	3.05	0.96	0.74	5.85	0.47
1.00	26.4	20.3	32.7	40.2	1.73	0.25	0.61	17.08	2.85
4.93	28.4	38.4	64.8	94.1	2.73	0.54	0.16	20.31	7.63
0.07	19.7	41.6	47.85	99	2.4	0.56	0.49	6.98	1.04
8.61	26.8	27.9	94.1	87.4	1.46	0.36	0.1	49	16.6
0.95	22.1	29.8	35.5	96.3	2.47	0.91	0.74	1.08	1.66
1.02	30.5	30.4	59.3	130	2.4	0.56	0.49	1.49	1.59
20.85	31.4	27.1	27.6	117	1.49	0.45	0.11	20.36	3.18
10.29	28.2	28.9	82.5	230.7	0.85	0.27	0.07	93.71	9.43
1.43	28.4	24.7	211.2	51.9	2.28	0.59	0.54	28.48	2.56
9.12	28	33.4	87.8	87.2	1.47	0.46	0.08	99.38	23.38
21.27	33.4	27.4	73.5	111	1.3	0.49	0.08	25.63	3.13
0.67	29.2	22.3	8.5	99	3.25	0.36	1.47	0.79	0.39
16.01	24.2	37.9	431.6	85.9	2.56	1.45	0.13	90.08	3.46
0.53	21.7	28.6	47.85	99	2.4	0.56	0.49	9.24	0.84
0.30	27.3	18.3	34.6	50.6	2.06	1.48	0.64	26.59	1.83
0.25	25.6	39.1	81.1	132.3	2.7	0.5	0.52	7.31	2.69

4.81	26.9	35.2	28.5	74.8	2.18	0.79	0.26	40.19	4
15.73	23.6	26.6	118.6	144.2	0.99	0.46	0.08	58.13	10.25
5.14	19.6	41.1	11.2	40.1	0.91	0.22	0.11	81.45	11
3.43	17	23.4	47.85	99	2.4	0.56	0.49	2.37	1.57
12.82	33.4	36.8	37.2	88.1	2.4	0.56	0.49	17.37	7.24
16.88	31.3	20.8	136.8	122.8	3.15	0.59	0.18	26.72	7.28
18.39	31.8	19.5	134.7	58.4	1.86	0.72	0.11	23.64	5.64
11.72	32.2	33.5	83.9	212.1	2.4	0.33	0.22	23.77	5.55
0.26	24.4	22.4	8.1	47	2.19	1.28	0.72	3.25	2.28
9.46	20.5	23.6	47.85	99	2.4	0.56	0.49	11.49	1.61
20.58	24	26	205.1	56.2	1.43	0.97	0.11	79.64	12.18
3.46	36.6	23.8	59.4	80.7	2.81	0.54	0.51	9.88	2.2
8.20	32.8	33.5	81.2	84.5	2.99	0.59	0.19	21.05	5.68
5.72	28.9	42.3	262.7	159.9	3.09	1.74	0.15	36.33	6.6
6.11	26.8	33.4	290.1	103.6	2.28	0.68	0.48	4.38	1.44
0.89	33.6	16.1	14.7	50	1.67	0.15	0.6	23.13	1.12
3.38	30.6	23.3	97.7	91.2	1.4	0.57	0.14	51.57	11.86
1.43	22.9	31.2	40.4	53.8	2.87	0.47	0.81	7.74	0.89
3.19	26.2	19.6	391.2	146.7	1.73	0.17	0.64	7.23	1.78
2.87	30.2	22.8	23.8	101	2.69	0.34	0.59	1.58	0.75
8.04	38.1	24	21.3	119.8	2.19	0.35	0.16	12.25	3.69
2.73	23.8	32.9	123.5	105.3	2.67	0.69	0.59	3.41	3.31
24.37	22	29.1	215.4	54.6	1.59	1.06	0.11	78	7.18
1.49	21.2	24.4	47.85	99	2.4	0.56	0.49	14.2	1.47
1.41	26.8	20.6	169.5	52.1	1.59	0.47	0.32	29.38	2.59
5.23	30.4	36.7	111.4	129.8	3.01	0.46	0.42	27.76	8.05
1.67	21.2	39	47.85	99	2.4	0.56	0.49	7.08	1.8
0.46	19.6	22.7	24.1	77.2	6.18	1.46	1.07	3.83	0.71
7.04	36.1	34.3	47.85	99	2.4	0.56	0.49	11.27	2.59
7.13	21.7	30.9	38.3	434.9	2.51	0.48	0.17	63.24	3.29
2.68	27	38.6	47.85	99	2.4	0.56	0.49	51.29	2.96
0.84	24.6	15.2	18.9	48.2	1.59	0.17	0.46	23.26	1.02
21.65	24	20.3	195.6	69.2	2.12	1.91	0.13	50.92	4.69
2.10	27.3	29.4	23.5	75.4	1.74	0.24	0.29	17.07	1.93
1.30	21	37.4	38.9	65.1	2.4	0.56	0.49	38.27	6.55
12.52	30.1	27.5	66.7	83.4	2.46	0.39	0.2	21.55	5.65
27.77	34.8	23	41.1	175.9	2.19	0.66	0.15	52	9.67
0.39	28.8	21.4	47.85	99	2.4	0.56	0.49	1.31	0.47

5.94	32.8	33.4	624.4	144.5	3.27	1.98	0.21	14.76	3.57
1.88	24.9	37	47.4	234.4	1.48	0.77	0.45	5.89	1.76
2.84	27.6	42.9	42.7	65.1	1.36	0.48	0.18	20.06	2.5
8.17	40	39.9	158.8	125.7	3.09	1.2	0.15	30.07	7.8
1.37	38.4	31.7	55	100.2	3.69	0.82	1.01	3.27	0.75
0.38	26.8	17.2	36.8	50.2	2.45	1.25	0.59	4.41	1.81
0.95	30.7	30.8	53.8	136.9	3.32	0.6	1.02	2.36	1.25
12.73	30.2	31.3	190.7	121.5	2.89	2.21	0.13	50.85	15.08
17.88	25.7	24.9	14.4	62.4	1.49	0.6	0.11	73	15.64
4.18	32.3	28	57.1	123.9	3.82	0.98	0.89	1.46	1.33
0.63	31.2	22	47.85	99	2.4	0.56	0.49	25.29	0.76
6.22	37	17.2	102.1	118.3	1.98	0.32	0.17	67.59	5.94
6.09	25.6	25.9	25.9	33.6	2.4	0.56	0.49	4.35	0.24
2.20	23.4	51.5	35.5	68.4	1.21	0.28	0.14	9.36	5.79
6.13	19.8	33.7	47.85	99	2.4	0.56	0.49	15.57	1.76
3.26	34.1	22.7	483.5	151.5	5.76	1.37	0.14	42.86	24.5
1.60	26.2	27.4	9.4	63.9	1.25	0.6	0.3	10.13	3.03
1.29	38.2	30.7	80.9	98.5	2.57	0.85	0.75	3.49	0.99
8.37	23.2	33.9	47.85	99	2.4	0.56	0.49	34.67	1.04
1.20	26.7	26.6	28.9	73.9	2.81	0.56	0.84	2.54	1.25
0.69	16.6	41.9	47.85	99	2.4	0.56	0.49	22.1	2.92
2.20	28.1	23.2	13.3	51.8	1.19	0.3	0.27	12.44	2.44
0.37	35	44.4	36	98.5	5.12	0.38	1.05	1.98	2.26
0.55	26.6	15.9	4.3	44.7	1.69	0.83	0.47	31.21	3.96
0.63	21.6	29.3	44.2	180.2	2.26	0.39	0.51	8.31	2.73
1.10	32.9	46.9	157.5	160	5.09	2.54	0.33	44.15	3.58
1.05	24.9	30.9	7.4	53.4	1.35	0.28	0.39	6.9	2.28
20.05	38.9	21.4	41.6	91.7	1.42	0.58	0.14	38.93	11.43
0.74	23.8	16.2	165.2	52.3	1.9	0.47	0.49	11.1	4.61
5.37	29.7	39.2	28.5	128.1	3.02	0.61	0.3	7.83	6.3
0.47	33.1	32.5	14.3	53.8	2.81	1.25	0.73	1.53	4.3
6.32	35.6	34.5	48.7	127.1	4.98	0.25	0.77	9.36	1.79
4.17	31.5	22.4	58.4	117.2	1.42	0.33	0.31	24.94	3.84
1.12	33.3	20.7	8.5	92.5	2.32	0.2	1.1	0.59	0.77
7.05	30.1	16.2	11.9	187	1.33	0.31	0.09	35.22	6.33
0.30	32.5	24.7	111.6	131.2	2.54	0.42	0.91	3.8	0.48
14.19	22.8	29	30.3	130.9	1.47	0.43	0.07	49.71	12.29
4.07	20.5	60.1	97.9	106.3	4.79	0.6	0.49	19.24	4.69

2.39	24.4	27.8	85.4	122.4	1.59	0.95	0.23	48.3	1.57
0.67	22.6	12.4	17.6	38.5	1.5	0.51	0.33	38.91	3.82
22.88	27	33.3	62.8	97.7	1.02	0.51	0.11	56.36	9.09
1.96	32.3	31.8	47.85	99	2.4	0.56	0.49	21.69	2.78
1.91	39.3	23	11.7	37.9	0.95	0.29	0.23	18.57	2.35
1.35	32.3	17.9	130.9	84.1	2.89	0.9	0.67	14.01	2.67
0.56	25.2	19.2	20.8	93.2	2.41	0.33	0.7	4.2	2.07
2.78	24.3	35.7	39.3	165.3	1.65	0.6	0.68	7.12	1.21
9.50	27.2	31	111	163.7	2.09	0.87	0.11	84.55	15.91
13.27	32.1	26	56.2	151.3	1.65	0.42	0.14	39.29	15
0.39	26.6	12.8	47.85	99	2.4	0.56	0.49	18.73	1.78
0.57	26.3	21.8	9.2	45.7	0.98	0.71	0.32	3.28	0.78
0.88	31	21.3	15.5	50	2.4	0.56	0.49	1.35	0.45
0.23	29.5	35.2	47.85	99	2.4	0.56	0.49	7.51	2.84
6.88	34.3	45.4	27	109.4	2	0.29	0.2	23.05	2.75
14.67	20.6	46.2	65.6	103.3	1.3	0.42	0.35	35.43	1.43
1.01	28.7	34.1	7.5	77.7	1.61	0.28	0.75	1.63	0.84
2.81	20.2	27.6	12.1	122.9	1.44	0.27	0.13	27.08	2.92
0.23	29.3	27.6	19.8	82.3	4.21	0.61	1.02	9.84	0.9
8.13	30.2	27.6	79.3	90.1	1.31	0.36	0.19	14.21	1.74
23.02	24.3	29.1	199.3	67.2	1.58	1.16	0.13	91.08	5
2.69	24.8	30.1	155.8	54.1	1.61	0.62	0.26	36.88	7.31
3.15	30.4	46.9	45.5	69.2	1.94	0.57	0.35	4	2.29
5.68	29.1	30.3	62.3	153.4	1.58	0.71	0.21	17.67	18.62
0.80	27.3	41.4	20.6	67.2	3.82	0.36	1.12	2.57	0.94
3.24	26.1	44.4	173.6	116.4	4.12	1.29	0.57	6.75	3.3
6.06	23.9	37.9	73.1	81.1	2.33	0.76	0.19	31.32	8.63
2.31	30.2	33	295.2	101	2.64	1.19	0.15	64.27	7.73
10.40	33	23.5	76.4	101.8	2.02	0.91	0.13	12.69	7.46
5.78	37.3	26.7	108.2	143.5	2.4	0.56	0.49	7.31	1.88
1.30	30.4	21.7	56.3	60.3	2.22	0.49	0.6	6.65	1.67
5.35	24.5	46.3	588.9	231.6	2.33	1.44	0.2	11.9	2.65
3.36	37	17.3	21.2	102.4	1.07	0.33	0.12	11.75	5.58
3.11	25.2	33.4	267.1	77.1	2.5	1.1	0.26	9	3.12
7.27	33.9	24.2	131.1	154.5	2.46	1.28	0.13	25.92	12.08
3.97	21.7	45	83.1	206.5	3.48	1.1	0.46	16.33	1.07
12.62	30.4	52.4	11.1	64.8	1.16	0.38	0.13	37.46	6.77
13.26	29.5	23.9	112.1	126.7	2.08	0.9	0.11	66.73	17.55

0.42	31	23.1	41.4	83.7	2.43	0.59	0.83	5.36	1.08
0.46	26.1	29.8	28.3	124	3.26	0.59	1.29	1.43	0.78
9.81	27.7	13.5	17.4	88.3	2.24	1.06	0.11	16.55	3.73
13.60	31.1	28.9	123.2	133.5	2.84	1.39	0.14	30.93	6.57
14.21	31.5	22.2	135.8	121.4	1.91	0.46	0.16	16.5	5.25
1.40	24.6	34.3	34.6	29.3	1.5	0.84	0.81	6.42	0.74
7.81	34.1	41	33.7	181.5	2.4	0.56	0.49	2.45	1.1
5.50	28.4	44.3	47.85	99	2.4	0.56	0.49	30.47	4.94
13.71	28.3	20.1	109.1	99.9	1.49	0.34	0.11	85.55	6.73
0.82	30.2	31.7	54.9	48.6	1.45	0.97	0.15	37.07	5.4
2.92	28.8	32.2	57.8	63.2	2.26	0.28	0.95	3.74	0.43
5.56	24.1	30.1	166.4	144.9	2.57	1.02	0.09	92	8.11
0.75	28.1	25.2	33.9	58.9	2.85	0.27	0.84	6.74	0.79
0.28	29.8	21	84	55.4	2.75	4.23	0.37	7.95	3.62
18.40	32.4	22.7	75.7	182	1.37	0.72	0.18	20	6.67
7.43	28.1	49.3	1030.4	156.9	4.6	2.46	0.27	24.37	2.78
5.04	21.2	20.6	77.2	40.1	0.29	0.13	0.07	18.4	37.86
0.80	32.2	41.6	34.5	56.7	2.72	0.84	0.63	6.24	0.65
28.25	30.5	33.5	43.1	96.9	1.94	1.58	0.13	21.54	4.46
1.00	32.6	38.2	45.8	116.3	4.1	0.86	1.29	1.05	0.52
7.86	29.9	27	602.8	83.5	2.4	0.56	0.49	5.49	2.88
4.24	31.9	33.8	14.3	75.4	0.98	0.51	0.34	8.82	0.59
1.40	30.9	39.7	10.9	113.9	2.15	0.22	0.75	2.15	0.43
24.19	24.8	25.7	96.5	91.8	1.58	0.49	0.12	83	6.83
15.37	24.1	27.1	100.4	148.4	1.02	0.42	0.35	17.34	3.2
3.64	35.8	39.6	20.5	140.4	2.1	0.34	0.8	8.15	0.4
8.70	27.1	43.1	18	83.3	1.28	0.4	0.13	28.62	6.62
10.72	27.8	33.8	1131.4	59.6	3.77	1.75	0.15	41.13	8.87
23.08	36.5	19.6	38.6	131.8	1.48	0.47	0.12	29.33	7.5
2.26	19.9	54.4	144.4	111	4.33	1.27	0.32	24.22	7.94
1.16	26.4	33.6	275.6	140.7	2.91	1.28	0.55	2.18	2
11.12	20.9	33.9	59.9	163.7	2.04	0.89	0.1	50.7	10.1
1.16	28.4	27.6	35.7	92.6	2.73	0.82	0.78	8.33	1.03
5.12	21.1	41.4	13.4	119.8	1.37	0.33	0.1	25.2	10.7
10.85	29.5	33	64.6	143.1	3.07	0.59	0.15	55.47	3.93
2.40	26.4	34.8	24.5	87.1	2.1	0.49	0.92	1.85	0.76
1.19	26.3	30.8	26.1	73	2.93	1.36	0.36	60.83	2.5
2.85	28.1	41.2	63.4	251.7	2.4	0.56	0.49	5.1	2.45

19.15	27.6	35.2	175	133.5	1.65	0.64	0.11	94	7.09
0.50	36.6	28.8	14.7	79.6	3.23	0.4	1.17	0.82	0.25
0.51	28.6	58.1	47.85	99	2.4	0.56	0.49	4.82	2.27
1.67	28.2	23.1	16.9	95.8	1.16	0.3	0.34	12.32	2.94
13.22	22	46.7	17.4	130.1	1.28	0.45	0.61	6.49	1
11.88	25.8	35.9	131.8	81.1	2.53	0.9	0.31	6.45	2.9
7.57	26.6	36.8	45.2	36.2	1.73	0.53	0.27	12.33	3.15
1.56	25.5	21.9	31	60.3	1.64	0.9	0.12	28.83	2.17
14.33	26.9	31.3	85	149.6	1.21	0.3	0.12	27	10.67
0.49	28.2	30.2	19.8	53.3	2.85	1.47	0.51	2.16	0.39
7.88	34.8	38.1	334.9	104.2	2.44	0.88	0.23	74.91	6.61
2.68	20.3	34.8	9.2	27.9	1.26	0.37	0.13	27.54	18.46
11.62	35.7	29	140.9	261.9	1.77	0.4	0.14	33.29	9.71
0.43	23.6	31.4	40.5	83.4	2.75	0.54	0.59	15.42	0.8
19.68	33.2	17.3	26.9	155.6	2.02	0.48	0.16	27.25	9.75
21.36	33.5	31.5	45.1	103.3	2.93	0.44	0.19	50.05	7.95
1.06	28	33.4	15.4	191.2	2.17	0.43	0.93	4.35	3.98
1.67	25.3	29.8	27.8	120.1	2.84	0.32	1.05	1.33	0.71
3.89	17.3	61.1	22.9	101.1	1.25	0.38	0.26	13.08	3.46
19.98	33	28.6	44.3	115.2	2.01	0.8	0.34	14.71	2.94
15.33	22.1	30.3	11.4	54.3	1.73	0.38	0.13	71.38	5.15
3.02	27.9	49.2	45.6	141.9	1.3	0.19	0.13	22.31	8.46
24.40	34.7	22.2	46.4	195.8	1.81	0.53	0.15	60.2	12.4
13.20	30	23	13.9	46.4	0.88	0.4	0.07	60.43	21.29
1.16	28.8	28.5	100.1	280.2	5.17	0.37	1.31	0.39	0.13
17.43	30	38.5	175.2	107.3	2.13	0.5	0.11	28.73	8.27
10.78	30.2	21.8	52.3	128.4	1.45	0.38	0.46	5.87	1.74
4.85	18.9	31.5	26.5	52.5	3.44	0.48	1.82	4.84	1.04
10.70	29.3	27.6	20.6	200.6	1.01	0.33	0.08	31	12.25
0.13	31.1	20.9	38.6	66.2	2.39	1.06	0.64	9.31	1.91
3.04	22.9	19.9	4.7	45.6	1.31	0.51	0.25	23.96	3.28
0.88	28.4	43	10.4	82.8	2.07	0.43	0.65	0.89	0.75
0.80	25.1	17.1	102	78.6	2.13	1.91	0.36	27.5	4.89
0.48	21.2	12.5	47.85	99	2.4	0.56	0.49	26.41	2.51
0.27	31.2	21.1	3	41.5	2.2	0.2	0.63	1.98	1.4
7.09	23.7	44.3	20.4	153.7	2.57	0.34	0.33	25.48	2.82
1.07	30.1	29.1	20.7	98.8	3.46	0.51	0.6	4.37	1.77
1.68	31.6	25.4	21.9	113.1	4.17	0.42	1.35	1.59	0.13

1.34	28.3	27.3	10.5	122.2	1.48	0.38	0.8	1.5	0.88
6.06	30.7	36.3	47.85	99	2.4	0.56	0.49	3.65	1.06
1.97	32.7	32	192.1	228.6	3.76	1.03	1.71	2.69	0.47
1.58	26.6	33.9	57	113.1	2.71	0.33	1.27	1.76	0.42
25.40	19.9	41.2	26.6	79.4	1.11	0.43	0.17	21.65	0.82
14.79	28.1	38	46.9	179.8	1.67	0.73	0.09	28.22	9.56
0.99	28.1	34.2	14.8	146.3	4.58	1.14	1	2.33	1.26
1.54	27.4	36.8	33.3	140.6	3.71	1.31	1.08	1.46	1.12
17.26	22.5	50.3	121.8	188.3	2.4	0.94	0.13	53.08	6.15
19.60	33.7	27.5	86.4	234.6	1.96	0.96	0.11	30.36	5.36
1.11	33	25.3	164.2	88.1	4.77	0.26	1.85	1.45	1.37
1.13	25.5	24.1	65.7	111.6	2.31	0.47	0.49	8.37	4.29
1.98	23.5	30.7	47.85	99	2.4	0.56	0.49	2.45	4.94
9.95	35.3	25	141.3	146.8	1.58	0.54	0.52	2.69	2.12
5.52	28.3	35.4	17.6	120.1	0.88	0.27	0.26	11.31	0.77
4.45	23.6	43.4	98.6	76.7	2.38	0.48	0.16	16.88	8.38
8.15	31.8	27.3	269.6	232.2	2.32	0.74	0.17	11.88	6.18
2.81	20.9	38.7	21.6	102.5	2.73	0.43	0.26	11.08	4.73
18.43	36	35.7	88.4	114.9	2.06	0.38	0.44	12.05	4.09
0.97	28.3	30.3	53.2	104	2.89	0.55	0.87	1.64	2.02
4.65	25.3	46.8	137.9	105.2	2.23	0.53	0.56	11.79	1.25
3.37	33.3	33.8	44.2	116.7	2.96	0.39	0.22	7.18	10.95
10.88	30.3	28.9	47.85	99	2.4	0.56	0.49	15.22	2.04
0.65	29.6	29.2	60.9	109.6	2.96	0.92	0.78	3.81	0.51
0.42	25.9	34.3	15.7	181.9	4.43	0.59	1.57	2.96	0.94
14.56	27.7	39.1	20.6	175.8	1.4	0.46	0.27	25.93	4.07
0.18	29.1	17.4	15.9	33.3	1.58	0.46	0.45	12.69	2.29
0.91	27.6	45.9	63.3	143.5	3.56	0.51	1.29	3.38	1.94
0.58	29.7	29.2	79	159.9	3.74	0.75	0.92	7.67	1.15
2.50	24.5	43.8	210.9	131.3	3.44	0.67	0.63	2.08	1.54
0.66	28.2	35.9	37	63.8	2.26	0.67	0.57	21.58	0.98
16.85	30.3	35.4	60.5	94	2.03	0.42	0.11	41.15	10.18
15.57	29.2	38.9	47.85	99	2.4	0.56	0.49	5.49	1.55
1.60	34	28.8	34.4	80.2	2.79	1.15	0.69	1.57	0.81
0.78	26.7	46.1	52.3	70	2.78	0.5	1.15	2.35	1.04
1.75	26	37.6	17.8	78.4	1.04	0.27	0.3	5.73	3.67
0.74	21.2	20.2	17.6	54.6	1.36	0.03	0.37	7.3	1.65
1.57	32.9	22.3	17.4	65.7	1.84	0.32	0.48	2.71	0.63

0.53	28	23.3	272.6	55.5	2.57	1.34	0.68	9.1	1.21
0.53	28	23.3	272.6	55.5	2.57	1.34	0.68	6.01	1.26
1.62	28.8	31.9	47.85	99	2.4	0.56	0.49	8.35	1.76
1.03	37.1	44.1	39.9	55.4	3.4	0.55	1.18	1.46	1
0.64	25.7	47	77.1	97	2.99	1.13	0.33	7.03	2.21
1.94	28.2	27.7	36	178.9	3.89	0.54	0.86	9.6	1.35
1.84	26.1	26.1	71.9	191	4.31	0.52	0.83	2.57	0.76
2.25	31.3	26.9	33.7	104.9	2	0.35	1.01	1.66	0.91
0.30	30.6	26.3	348.6	97.8	4.9	0.83	1.05	4.61	1.21
4.91	30.7	36	66.5	78.9	3.17	0.73	0.18	19.33	5.78
3.90	30.7	36.5	114.7	109.2	3.11	1.25	1.17	2.74	0.92
1.66	25.8	23.6	532.2	94.6	2.53	0.62	0.53	5.45	1.11
1.51	28.1	38.4	15	63.4	1.6	0.46	0.21	32.14	4.29
0.64	28	23	10	59.3	2.4	0.56	0.49	2.86	1.04
4.87	23.4	53.2	87.4	125.6	1.56	0.65	0.09	11	30
1.58	20.9	27.5	17.4	72.8	2.14	0.37	0.7	7.71	1.14
6.27	18.9	34.8	54.4	140	2.05	0.8	0.52	8.85	2.12
0.42	31.1	25.6	93.4	86.8	4.07	0.47	1.79	1.8	1.97
7.89	28.5	31.3	221.8	91.6	2.32	0.71	0.15	70.13	12.47
0.63	32.4	23.5	57.3	90.4	1.46	0.12	0.67	3.7	1.28
8.32	23.4	37.3	58.4	207.7	2.58	1.17	0.1	28.3	7.8
0.97	38	26.9	174.2	76.5	4.57	0.92	0.68	3.65	1.54
25.71	32.3	32.2	64.1	85.6	2.01	0.66	0.15	32.53	6.2
0.71	22.9	39.8	21.1	95.8	2.18	0.32	0.63	11.19	1.29
2.25	36.8	53.8	47.85	99	2.4	0.56	0.49	10.92	2.39
3.27	25	21.9	16.8	106	4.2	0.68	0.4	8.7	3.23
14.35	27.2	28.2	16.7	65.9	1.4	0.45	0.12	14.08	5.25
1.02	32	24.6	57.6	135.6	2.73	0.5	0.88	3.88	0.69
4.94	30.5	41.3	49.8	113.4	1.91	0.29	0.19	40.11	6.68
0.36	34.1	20.7	43.3	58.8	2.08	1.32	0.53	12.66	2.94
1.33	26	36.2	53.6	58.5	3.02	0.64	0.24	17.25	2.42
0.23	25.8	16.5	16.5	38	1.38	0.59	0.36	53	4.03
19.95	29.9	27.9	64.6	112.9	1.52	0.39	0.12	13.9	9.58
0.81	28.6	35.1	65.5	140.9	2.57	0.22	1.41	1.4	0.43
1.92	29.2	26.4	69.8	85.6	3.35	0.41	0.9	4.66	0.76
14.47	34.5	26.3	58.3	100.9	2.99	0.44	0.22	9.45	2.91
0.60	25.9	63.8	30.2	83.3	1.94	0.22	0.65	3.4	0.48
14.00	26.1	38.9	242.8	146.1	1.4	0.38	0.43	15.35	3.95

1.09	20.3	19.9	55.1	47.6	2.08	1.02	0.51	10.37	1.39
0.87	25.5	18	5.7	66.9	2.4	0.56	0.49	3.29	2.24
6.30	36.6	41.3	374.7	132.9	4.58	3.29	0.64	9.7	2.02
23.01	26.8	44.1	280.7	115	1.58	1.1	0.17	68.35	5.47
0.73	34.5	38.8	78.3	132.4	4.18	0.39	1.2	2.6	0.38
1.49	30.5	26.2	45.5	64.9	1.43	0.11	0.44	20.43	1.75
6.14	34.5	28.6	97.6	117.4	2.43	0.57	0.54	12.3	0.41
2.79	22.9	24.4	27.8	205.1	2.45	1.14	0.36	7.22	2.72
4.56	23.2	40.6	14.7	54.2	1.16	0.27	0.27	17.78	1.48
0.88	25.3	41.4	44.1	90.4	3.64	0.53	0.86	3.57	1.37
10.91	24.4	33.9	21.9	51.7	1.49	0.47	0.09	58.56	4.22
2.08	25.7	33.5	76.6	64.2	1.45	0.61	0.54	16.3	1.48
5.43	22	47.9	76.1	99.2	2.77	1.19	0.12	18.5	10.33
1.22	31.1	49.4	213.6	61.5	2.52	1.09	0.61	4.95	1.7
3.56	33.3	18.6	20.6	44.3	4.02	0.41	0.51	4.39	0.63
0.50	34.4	28.4	140.7	124.4	3.35	2.14	0.81	3.28	4.23
0.64	22	38.1	13.2	62.5	2.4	0.56	0.49	8.98	0.8
4.23	29.8	53.5	65	78.1	2.64	1.57	0.3	6.4	3.43
8.50	25.9	30.3	27.9	111.4	2.4	0.56	0.49	10.94	1.96
1.14	23.5	35.5	18.2	102.4	2.05	0.46	1.06	1.7	0.75
0.94	34.8	29.3	22.2	66.3	2.67	0.43	1.1	1.99	0.56
0.75	40.9	32.6	669.2	112.6	1.72	2.72	1.12	2.04	1.24
1.38	23.5	23.3	99.9	51.5	2.05	0.43	0.53	3.77	2.08
1.27	28.3	28.3	65.2	67.1	3.54	0.62	1.06	1.75	0.87
0.70	28.6	25.4	19.3	130.2	3.11	0.48	0.94	1.76	0.6
0.50	33.5	27	166.2	166	3.09	0.55	1.55	1.14	0.51
0.95	37	36.9	120.3	79.7	5.62	1.3	1.28	1.45	1.38
0.84	32.1	27.1	47.85	99	2.4	0.56	0.49	6.65	2.37
17.50	28.1	31.4	58.9	144.1	2.62	1.51	0.12	36.58	9.67
0.32	24.3	11.2	15.4	45.2	1.87	0.46	0.48	10.23	1.21
0.32	30.8	23.4	17.5	42.9	2.54	1.23	0.42	7.64	2.26
1.01	25.4	28.7	63	57.3	2.61	0.93	0.64	10.83	0.66
1.59	28.6	31.7	120.2	151.7	3.78	2.82	0.32	3.47	4.78
0.90	30.4	23.9	78.1	76.2	3.31	0.35	1.34	1.77	0.36
0.32	34.7	37.2	47.3	132.7	3.25	0.41	1.08	5.08	0.81
0.32	29.1	15.4	3.2	27.2	1.44	1.71	0.3	4.83	0.9
8.56	26.4	32.7	21.6	136.4	2.49	0.47	0.99	4.55	0.81
0.99	29.3	36.1	18.3	51	3.56	0.96	1.03	4.27	1.03

3.87	21.9	39.5	35.8	262.2	2.19	0.51	0.6	3.17	2.33
3.67	32.2	67.1	102.2	102.2	2.13	0.52	0.24	49.21	4.5
2.44	29.7	20.4	37.2	94.3	3.47	0.73	1.13	1.48	0.7
0.70	22.2	26.2	9.2	35.1	1.39	0.47	0.31	41.23	2.1
0.28	30.8	22.2	17	43.1	2.61	0.38	0.53	7.34	3.57
1.32	21.9	38.5	27.8	151.6	1.73	0.38	0.68	3.1	1.06
0.23	28.6	32.4	15.4	70.5	3.12	1.25	0.61	3.87	0.82
5.88	29.2	23.4	67	187.9	1.66	0.53	0.1	50.8	25.2
0.82	35.1	24.5	82.9	69.7	3.59	0.24	1.44	1.82	0.39
0.18	30.5	22.5	15.5	47.7	4.63	0.17	1.26	1.52	0.7
2.30	29.5	30.9	335.2	74.7	3.66	0.78	1.66	1.51	0.36
0.82	24.7	23.1	15.9	96	2.4	0.56	0.49	2.24	1.43
0.13	34.7	28.8	25	128.7	3.7	0.83	1.35	2.15	0.59
1.63	32.4	44.3	1.9	110.9	3.36	0.69	1.57	5.48	0.19
1.97	31.7	25.6	45.7	93.8	3.04	0.41	1.23	1.67	0.33
1.77	20.8	17.8	17.8	49	2.11	0.35	0.77	5.56	0.65
7.86	46	32.9	1466.5	74.4	7	2.4	0.41	5.05	1.83
0.31	33.4	17.6	11.6	52.4	2.45	0.64	0.77	1.29	0.51
0.83	24.9	24.7	47.85	99	2.4	0.56	0.49	2.02	1.24
0.19	26.2	13.5	27.5	62.9	2.12	1.45	0.41	17.41	3.22
2.37	24.9	33.4	54	59.7	3.39	0.97	0.79	2.33	0.89
5.00	27.9	31.7	51.9	80	2.27	0.36	0.28	21.79	2.79
1.05	30.1	41.6	103.8	60.9	5.71	0.99	0.82	6.7	1.23
0.22	19.1	19.1	42.4	52.4	1.29	0.81	0.6	10.13	2.92
1.94	31.5	41.7	42.2	212.8	3.39	0.54	1.63	1.23	1.17
1.50	30.3	37.1	85.8	132.4	3.18	0.3	0.41	6.98	3.02
3.40	26.1	38.4	234.2	110.3	2.32	0.5	0.94	3.04	0.63
0.86	30.6	23.6	11.5	68.8	3.3	0.52	1.17	1.01	0.39
0.53	33.1	18.6	16.8	42.4	2.43	0.77	0.51	25.49	1.49
3.11	31.7	44.5	40.4	212.8	4.48	1.02	0.36	9.44	3.61
0.25	26.7	32.1	105.4	128.9	3.11	0.96	0.65	1.97	1.98
1.73	31.1	32.7	16.1	45.2	3.96	0.37	1.32	1.14	0.48
9.71	25	31.7	266.7	98.3	3.22	1.83	0.12	45.25	4
0.44	25.5	23.8	47.85	99	2.4	0.56	0.49	4.16	0.84
17.78	32.9	30.3	58.5	126.9	2.65	1.07	0.14	33.5	10.86
0.46	22	28.5	5.8	40.2	2.6	0.25	0.95	2.59	1.59
2.12	35.1	23.4	18	95.6	2	0.26	0.8	1.35	0.49
1.57	22.5	36.8	24.6	41.6	1.85	0.28	0.57	23.7	3.05

0.54	34.6	31.6	47.85	99	2.4	0.56	0.49	2.98	0.51
0.85	26.6	12.3	108.7	49.1	1.43	0.33	0.28	8.18	1.39
7.89	30.1	18.9	113.4	123.7	1.18	0.34	0.08	25.13	7.25
1.86	21.7	29.6	29.2	115.2	3.4	0.68	0.54	3.69	1.06
0.47	32.2	20.9	51.5	41.4	2.75	1.26	0.51	25.61	7.78
2.71	26.4	24.8	44.6	175.8	2.27	0.91	0.29	4.97	4.21
0.94	29.6	31.4	17.1	86.7	2.45	0.4	0.71	7.8	1.58
4.71	25.5	22.7	21.7	73.2	2.66	0.4	0.52	14.81	2.52
0.23	35.9	17.3	15.5	42	3.06	0.8	0.88	7.61	0.91
3.12	27	34.3	29.7	105.3	3.45	0.73	0.68	2.76	1.22
1.93	31.3	19	30.9	217.6	3.11	0.76	0.59	3.1	1.2
9.29	24.5	26	571.8	152.7	1.27	0.37	0.13	48.69	8.62
1.58	30.1	28.1	10.7	112.6	2.55	0.53	0.98	0.79	0.22
3.42	25.1	36.3	37.5	133.4	3.48	0.93	0.32	18.94	2.56
0.35	29.2	16.6	10.7	41.6	2.17	0.83	0.33	7.55	3.67
0.57	40.2	20.8	22.4	68.2	3	1.02	1.27	4.91	0.42
1.58	25.2	25.4	107.4	114.6	2.99	0.58	0.76	1.84	1.07
0.47	28.2	24.8	40.3	85.8	3.25	0.53	1	5.3	1.4
0.60	26	26.1	11.4	57.4	3.05	0.34	0.87	3.4	2.63
1.11	26.6	20.7	47.85	99	2.4	0.56	0.49	9.02	2.08
0.71	23.5	15.4	9.9	39.6	1.46	0.6	0.42	16.67	3.14
15.84	28.8	37.7	83.9	120.9	2.57	0.49	0.15	55.2	9.53
0.83	20.2	17.2	20	47.4	1.22	0.97	0.34	19.32	2.79
3.26	21.2	38.1	18.2	37.7	2.4	0.56	0.49	12.39	1.04
5.50	32	32.4	27.4	88.6	2.01	0.45	0.25	6.4	3.44
12.73	28.2	39.2	832.3	123.6	3.14	3.29	0.12	30.25	5
2.68	34.8	44.6	40.9	72.9	3.84	0.61	1.01	4.37	1.79
0.46	30.6	24.3	360	55.8	0.7	1.48	1.01	3.26	1.12
2.87	21.3	42.7	37.4	119.8	2.25	0.64	0.29	7.03	2.9
0.15	35.7	21.4	14.3	47.2	3.3	1.61	0.84	2.04	0.38
3.23	35.3	29.8	32.7	114.4	3.81	0.36	1.04	1.3	0.77
1.39	29.2	28.8	23.6	89.5	3.44	0.46	1.47	0.9	0.55
4.68	33.6	20.3	424.2	117.9	2.1	0.6	0.44	19.05	0.77
1.80	18.9	40.5	43.7	134.4	3.08	0.63	0.45	14.67	3.11
1.12	27.3	26.4	44.9	104.8	4.43	0.48	0.89	4.16	1.15
1.40	27.8	27.3	26	102.8	2.53	0.4	0.93	1.77	1.31
0.52	41.1	25.7	31.2	82.7	2.37	0.91	0.66	2.85	0.5
1.06	32.3	41.4	22.8	92.5	1.73	0.34	0.56	4.86	1.91

3.35	27.3	27.8	24	174.3	2.72	0.29	0.71	4.92	1.66
2.32	30.3	30.3	211.2	175.2	3.68	0.8	1.34	1.57	1.27
0.46	28.4	28	24.5	54.7	4.54	0.37	1.23	1.7	0.39
1.44	32.4	33	38	115.8	4.66	0.43	0.98	2.34	1.18
2.02	32.5	27.1	47.85	99	2.4	0.56	0.49	8.27	2.14
0.67	23	20.2	61.9	87	2.02	0.62	0.88	20.57	1.36
0.57	22.6	25.1	22.2	41.7	2.04	0.43	1.02	0.92	0.32
0.85	32.2	34.2	47.85	99	2.4	0.56	0.49	6.51	2.16
1.01	29.9	38.3	253.2	154.1	3.53	1.11	0.63	3.35	2.4
0.26	23	25.2	114.1	103.6	4.5	1.85	0.66	12.82	2.12
1.19	27.3	34.8	24.5	67	3.27	0.5	1.33	1.62	0.38
1.68	34	35	457.8	267.3	3.32	0.65	0.98	2.86	0.61
0.68	30.6	22.8	40.8	70.3	3.56	0.6	1.07	11.29	1.46
0.67	36.2	32	120.2	59.7	4.02	0.6	1.42	1.01	0.44
1.54	29.4	30	16.5	101.9	2.44	0.44	0.86	3.27	0.85
0.36	29.6	17.2	8.8	53.2	2.3	1.63	0.62	4.6	0.85
7.23	26.6	39.4	136.5	97.3	1.83	0.6	0.13	10.15	4.46
8.05	32.5	28.3	115.4	203.3	3.32	1.08	0.37	12.68	5
1.07	23.6	38.3	29.3	95.9	2.34	0.4	1.04	2.24	1.12
0.25	32.6	23	8.5	63.3	3.38	0.46	0.79	3.86	2.23
2.47	24.4	35.8	97.1	171.1	3.41	0.3	0.6	3.72	3.77
6.29	35.2	39.7	23.2	129.7	3.56	0.37	0.63	10.65	0.32
0.87	30.9	34	70.6	133.2	3.91	0.99	1.6	0.81	0.56
2.15	24.8	37.1	59	101.5	2.39	1	0.79	2.41	1.77
2.01	25	20.5	20.8	72.1	3.96	0.11	0.99	11.99	0.65
4.58	31.8	33.8	18.3	92.2	2.85	0.41	0.37	2.03	2.05
16.69	27.8	23.5	76.6	117	3.43	1.57	0.13	28.08	9.46
1.96	34.1	25.6	121.6	71.2	2.4	0.56	0.49	6.04	2.16
0.40	29.9	46.6	34.1	65.4	2.55	0.55	1.16	1.72	0.95
15.18	30.6	28.7	98.4	272.4	2.9	1.13	0.16	36.25	14.25
0.40	26.2	56	47.85	99	2.4	0.56	0.49	4.14	4.27
3.27	20.6	38.7	12.4	60.6	2.1	0.35	0.61	3.92	1.34
0.99	29.3	17.6	16.9	70.2	2.99	0.89	1.03	1.44	0.65
2.59	25.9	29.2	47.85	99	2.4	0.56	0.49	9.51	2.14
6.33	25.1	38.1	25.6	152.5	2.26	0.46	0.71	3.24	1.13
1.63	19.8	37.6	25.4	94.6	3.1	0.56	0.74	10	3.08
1.46	23.8	42.7	79.4	204.3	3.67	0.54	1.04	1.17	2.15
0.92	36.2	37	454	217.3	3.8	1.57	1.32	1.67	0.55

0.63	32.6	17.7	11.2	51.7	2.34	0.56	0.64	1.98	0.41
11.54	33.6	25.1	126.8	160.9	1.93	0.5	0.12	88	9.92
1.94	37.1	35	753.7	74.9	4.09	0.9	1.45	4.2	0.68
1.38	25.8	42.6	71.4	73.4	3.36	0.53	0.94	3.05	2.11
6.12	21.1	38.9	150.5	125.8	2.51	1.33	0.32	10.41	3.41
1.67	31.2	35.3	456.3	120.5	2.78	0.71	0.7	3.77	2.29
1.06	26	44	66.6	201	1.91	0.5	0.34	12.18	2.44
0.52	36.8	24.8	27.6	146.2	3.74	0.61	1.43	0.97	0.64
1.84	24.7	35.2	63.6	36	2.29	0.78	0.3	31.83	2.17
0.82	37	21.5	86.8	62.7	3.34	1.29	0.76	3.17	1.17
0.78	40.5	26.6	787.2	84.5	4.04	1.7	0.85	4.05	1.76
0.71	28.5	19.3	44	50.3	3.32	0.66	0.77	8.65	3.14
0.25	35.1	26.8	47.85	99	2.4	0.56	0.49	5	1.16
3.54	25.6	28.7	93.3	120.4	5.89	1.1	0.7	3.07	2.33
1.05	24.6	33.6	29.2	122.5	2.22	0.49	0.78	2.88	0.63
1.06	27.3	30.1	238.6	103.2	1.62	0.47	0.42	12.5	3.1
4.20	24	28	17.7	112.7	2.54	0.52	0.3	3.93	2.63
7.05	25.2	39.6	58.2	87.6	2.4	0.56	0.49	6.55	1.65
0.36	29.7	15.1	26.5	39.7	1.73	0.49	0.49	10.8	2.2
4.80	31	37.4	21.5	104.7	2.51	0.56	0.5	16.06	2.04
1.16	26.9	31.4	28.5	101.3	3.05	0.36	1.16	1.38	0.63
0.47	41.1	32.4	47.85	99	2.4	0.56	0.49	6.35	1.02
0.72	30	26.7	51.5	107.6	2.79	0.48	1.29	0.84	0.26
1.24	29.6	28.6	16.4	156	2.07	0.38	0.78	2.31	0.77
0.82	24.2	37.4	44.8	102.5	3.72	0.69	1.11	2.91	1.41
0.34	28.1	32.5	8.7	66.6	1.67	0.05	0.83	7.06	1.13
0.46	22	21.6	87	67.3	1.28	0.15	0.62	5.13	1.23
1.37	32.5	25.1	33.8	114.7	3.32	0.53	1.05	9.05	2.57
0.88	30.1	41	86.4	87.8	4.05	1.63	0.82	4.11	1.27
4.89	31	44.3	987.8	71.4	7.38	1.88	0.82	5.37	1.22
0.94	29.2	20.2	56.8	87.2	2.97	0.71	1.03	1.65	0.65
0.97	33.8	28.3	7.2	144.9	2.49	0.41	1.06	0.96	0.62
0.77	30.8	27.7	262.8	115.8	2.76	0.67	0.82	3.55	0.76
0.91	26.6	50.9	31.6	87.1	3.23	0.63	1.21	2.23	0.41
2.60	23.6	24.5	198.1	52.8	2.37	0.99	0.4	21.23	2.43
0.53	29.9	22.7	11.5	54.3	2.25	0.46	0.33	6.82	2.3
0.74	20.5	13.2	6.3	39.9	1.11	0.06	0.65	20.34	1.54
0.70	31.5	35.5	85.6	109.4	4.03	0.7	1.09	1.9	0.51

4.06	31.4	29	73.3	168.5	6.47	0.76	1.35	2.33	1.29
2.12	28.1	28.9	129.2	73.5	3.44	1.19	0.39	33.21	5.92
2.35	20.9	30.3	14.6	11.6	1.94	1.07	0.35	18.14	1.43
0.11	25	17.2	16.2	36.5	1.69	0.81	0.69	6.45	1.14
2.63	29.9	33.1	18.2	109	1.86	0.3	0.39	6.46	1.44
2.39	23.8	34.1	500.2	114.5	3.15	1.4	0.47	13.21	1.72
12.89	26.1	33.7	115.6	122.5	2.4	0.56	0.49	15.9	4.43
2.41	28	47.7	72.9	187.8	2.93	0.81	0.45	10.53	6.22
1.09	25.8	37.5	149.6	328.7	2.8	0.17	0.62	5.32	0.97
0.30	32.8	22	29.6	43.6	2.79	1.12	0.85	3.31	0.73
1.44	24.5	18.7	11.2	76.3	2.11	0.39	1.1	3	0.55
0.47	22.6	17.4	19.8	49.8	1.5	0.44	0.33	14.06	2.03
6.48	30.3	27.6	129.4	118.8	2.38	0.98	0.39	22.92	0.72
1.60	24.1	33.6	35.5	74.7	3.47	0.62	0.41	10.71	1.44
1.19	24.8	27.9	33.4	113.8	3.13	0.41	1.09	2.66	0.63
0.26	29.4	23	8.5	54.1	2.42	1.32	0.68	10.43	1.24
2.77	19.1	45.2	24.3	54.6	1.72	0.53	0.18	30.72	4.61
1.16	27	23.6	18.6	83.3	2.72	0.48	0.66	3.27	1.86
1.64	29.7	32.5	40.3	102.2	2.79	0.71	0.68	25.5	0.54
1.62	25.9	25.9	78.7	98.2	2.09	0.56	0.66	11.21	2.73
4.67	35.4	35.2	30.8	79	3.33	0.55	0.86	6.56	0.98
0.73	32.7	26.6	8.9	41.8	1.93	0.51	0.91	1.8	0.58
2.78	22.8	15.9	392.3	68.3	2.2	1.07	0.32	13.72	4.16
9.18	23.6	32.3	180.63	192.5	2.49	0.89	0.42	20.48	2.14
1.57	33.2	27.9	59.9	52.4	4.21	0.52	1.54	2.52	0.66
0.29	30.2	37.9	47.85	99	2.4	0.56	0.49	12.35	2.14
0.75	24.2	27.1	21.3	63.3	2.19	0.39	0.79	6.62	1.16
0.38	30.4	28.5	35.9	80.3	1.7	0.33	0.48	7.04	1.58
0.76	36.1	26.7	967.7	68.2	3.24	1.14	1.25	3.69	2.12
0.28	39.6	29.7	56.1	58.9	3.61	0.94	0.76	6.41	1.82
0.66	26	22.7	47.85	99	2.4	0.56	0.49	9.18	2.63
3.40	24.7	39	494.7	114.2	2.58	0.61	0.55	5.35	1.25
1.65	22.8	24.7	20.6	89.1	3.01	0.38	1.18	1.56	0.54
0.33	28.2	29.2	5.8	39.3	1.52	0.11	0.63	23.95	3.9
7.32	25.4	27.7	18.1	93.1	2.2	0.29	0.17	23.06	5.82
0.63	29.7	17.7	30.1	55.6	2.29	0.51	0.66	6.12	1.62
3.35	28.8	26.9	98.4	82.2	2.27	0.53	0.17	59.59	8.65
0.62	30.9	21.7	21.4	49.5	2.46	0.43	0.77	4.12	0.75

1.02	22.4	33.9	55.7	62	2.04	0.88	0.57	10.35	1.54
1.66	25.8	31.2	59.7	130.4	2.5	0.39	0.37	7.57	6.22
3.00	21.2	37.8	16	100.5	3.02	0.43	0.19	11.74	3.74
0.97	34.3	27.6	15.9	66.8	3.52	0.35	1.54	2.05	0.77
2.55	25	25.2	150.4	56.6	1.91	0.47	0.17	19.94	8.29
1.06	34.1	18.6	11.2	40.4	2.41	1	0.92	7.61	1.63
0.80	28.1	18.5	45.8	160.7	2.25	0.73	0.8	4.3	1.1
0.65	29.5	33.1	18.4	62.1	2.68	0.34	1.16	2.75	0.29
1.16	31.1	30	61.9	83	2.45	0.3	1.02	7.21	0.23
0.27	27.1	29.5	13.2	58.6	2.7	0.95	0.65	7.17	1.23
0.68	27.8	71.8	165	330.6	3.57	0.49	0.94	6.7	1.37
8.36	34.1	33	72.9	208.4	2.2	1.36	0.12	24.17	10.83
7.90	32.9	23.3	68.7	81.5	2.06	1.16	0.1	31.3	16.2
1.13	33.2	40	38.4	124.5	3.21	0.55	1.45	2.34	0.48
0.73	28.5	31.8	52.8	79.1	4.48	0.42	1.22	4.09	1.28
1.03	32.3	44	32.7	62.4	2.46	0.43	0.77	5.26	1.26
14.44	33.2	28.7	84.3	108.6	2.06	1.12	0.12	36.33	10.08
0.40	37.4	26.6	47.85	99	2.4	0.56	0.49	4.31	1.14
2.37	25.4	24	18.1	49.2	1.65	0.8	0.43	8.6	1.74
3.98	29	35.8	163.7	66.8	2.02	0.79	0.88	5.86	0.9
3.92	23.8	30.2	188.7	95.3	2.25	0.99	0.39	13.49	1.44
1.15	36.9	20.9	84.3	113.7	2.49	0.39	0.92	1.63	1.01
2.53	30.5	42.5	26	205.2	3.17	0.67	1.17	2.68	0.74
8.98	36.7	32	541.5	77.6	3.49	1.29	0.18	21.06	1.11
0.72	32.2	20.9	51.3	78.5	3.45	0.42	1.64	0.98	0.43
0.43	22.2	16	7.4	30.1	1.52	0.38	0.6	12	1.32
0.20	25.6	26.1	14.8	56.2	2.4	0.56	0.49	2.65	0.47
6.94	24	23.6	23.4	99.7	1.55	0.35	0.07	68	11.29
1.71	38.7	43.1	47.85	99	2.4	0.56	0.49	6.08	1.49
0.60	29	24.4	24.6	72.9	3.09	0.41	2.29	0.57	0.26
1.27	27.1	34.3	166.2	49	3.69	1.29	0.36	32.67	2.89
1.18	25	38.6	66.2	102	3.09	1.06	0.9	5.6	1.88
0.57	30.1	13.9	50.4	64.9	1.1	0.17	0.36	5.28	0.92
0.99	33.3	21.2	19.9	50.6	2.85	0.93	0.62	10.34	0.76
12.80	29	16.6	36.7	22.8	1.34	0.36	0.14	19.8	3.64
0.50	33.2	27.2	30.8	55.9	2.44	0.42	1.04	2.97	0.42
0.34	33.7	32.2	39.8	105.9	4.85	0.63	1.71	1.43	1.18
0.33	38.3	21.4	16	40.6	2.68	0.44	0.74	3.2	0.49

1.39	29.1	24.1	90.7	72.4	2.65	0.53	0.4	18.75	2.48
5.01	22.3	25.9	21.9	55.5	1.91	0.59	0.11	35.64	7.64
3.01	24	16.8	26	56	2.38	0.85	0.4	6.33	1.03
5.58	23.7	29.8	317.9	45.7	2.61	1.01	0.18	59.17	3.72
0.72	26.3	29.9	47.85	99	2.4	0.56	0.49	11.18	3.12
3.30	39.6	28.2	1393.7	101.7	5.55	2.09	0.5	5.06	2.86
1.42	30.2	29.1	42.8	79.6	2.62	0.89	0.72	14.75	2.19
1.04	35.4	39.7	47.85	99	2.4	0.56	0.49	8.37	1.78
1.21	23.1	28.2	68.6	165.8	5.01	0.59	1.78	1.33	0.29
0.71	33.7	33.6	18	125.3	2.46	0.25	1.04	2.07	0.99
0.75	31.4	44.4	32.6	116	2.19	0.35	1.09	1.2	0.45
0.23	31.4	19.4	214.6	203.3	2.25	1.2	0.67	15.82	1.37
18.10	30.7	25.1	120.2	134.4	2.11	0.82	0.14	66.93	4.29
0.74	32.3	36.5	57.3	177.4	3.44	0.94	1.15	1.9	1.17
1.19	25.2	29.2	21.9	66.3	3.04	0.36	0.88	0.91	0.43
2.77	28.5	31.1	114.5	126.4	2.3	0.51	0.27	44.59	4.04
1.67	24.3	48.5	18.6	52.1	2.74	0.49	0.64	19.2	2.2
4.33	20.3	25.6	40.9	47.8	0.95	0.38	0.14	33	3.21
4.18	45.6	31.5	144.9	125.6	3.8	0.86	0.89	2.4	1.83
0.30	35.7	20.4	54.3	54.8	2.4	2.01	0.74	7.3	0.81
0.30	24.5	25.8	11.8	72.6	2	0.41	0.95	4.95	0.84
0.42	28.9	23.8	13.8	53	1.46	0.74	0.47	11.98	4.17
2.57	25.8	31.1	29.9	113.8	3.91	0.69	0.51	2.35	1.63
1.50	28.3	31.2	21.5	69	2.2	0.35	1.04	2.58	0.26
0.45	30.6	27.5	16	67.4	2.06	0.82	0.58	4.5	0.98
1.39	30.7	43.6	143.3	200.2	3.26	0.39	0.93	2.26	0.29
3.20	31	29.8	44.9	148.7	3.55	0.61	0.61	3.15	0.61
0.85	37.1	21.8	18.8	148.9	2.5	0.34	0.88	1.24	0.52
0.78	30.2	21	60.3	54.2	1.99	0.61	0.56	19.46	3.36
0.43	21.8	21.2	47.85	99	2.4	0.56	0.49	12.24	1.43
1.04	31	26.6	26.2	36	2.32	0.11	0.8	5.23	1.39
3.44	26.1	26.2	18.7	125.8	3.47	0.36	0.78	4.83	1.54
1.48	12.2	28.6	47.85	99	2.4	0.56	0.49	5.82	1.8
0.82	30.9	16.7	8.6	55.8	2.3	0.33	0.89	4.87	0.34
0.64	30.3	29.1	12.1	82.8	2.4	0.56	0.49	2.24	0.8
1.11	30.4	33.9	18.9	68.6	3.29	0.68	1.01	3.04	0.76
0.29	28.1	23.5	38.7	61.3	3.86	1.17	0.77	7.45	1.12
1.15	32.2	31.6	72.6	54.4	4.52	0.58	1.37	5.76	0.76

1.56	27.9	34.1	46.1	122.6	2.5	0.52	1.09	4.77	0.92
0.36	34.9	30.9	46.9	107.1	2	0.61	0.8	2.75	0.63
0.44	29.4	29.5	25.5	109.9	2.82	0.36	1	11.2	0.97
3.78	37.6	36.6	356.1	82.1	8.66	0.87	1.23	2.16	0.8
2.67	31.8	29.5	30.6	83.8	3.94	1.14	1.65	3.23	0.73
11.86	26.6	16.7	47.85	99	2.4	0.56	0.49	30.63	4.43
0.71	30.3	23.6	13.5	95.9	1.95	0.27	0.91	1.36	0.29
17.40	28.8	31.6	39.2	61.8	1.88	0.71	0.13	34.77	3
0.27	27.9	18.3	317.6	91.8	2.65	1.57	0.37	33.24	11.32
4.34	17	18.9	17.2	18.5	1.09	0.28	0.1	12.8	28.7
0.80	24.9	29.7	11.1	67.9	2.38	0.4	0.8	1.38	0.5
1.42	32.1	56.1	35	83.1	2.72	0.49	1.2	1.75	0.25
1.12	29.5	29.7	47.85	99	2.4	0.56	0.49	2.65	2.45
1.88	25.8	35.8	27.1	62.7	2.03	0.34	0.7	4.09	1.4
0.86	34.8	25.4	332.2	94.5	2.32	1.04	1.05	2.51	0.66
0.53	32.2	36.5	72.7	118.2	5.38	2.63	2.17	0.97	0.65
4.04	31.3	40.1	25.7	168	5.78	0.55	0.37	22.24	3.27
1.89	27	31.1	17.2	47.8	2.17	0.46	0.75	2.83	0.28
0.67	28	28.9	15.4	69.7	3.11	0.78	1.24	1.34	0.65
0.90	26.5	35	12.2	114.7	4.29	0.4	1.98	2.13	0.67
3.16	28.7	39.6	81.4	286	1.69	0.58	0.46	2.83	2.17
0.64	25.5	29.4	13.3	104.3	3.16	0.58	1.1	2.55	1.38
3.99	22.3	49.4	40.3	92.3	1.53	0.42	0.07	51.29	7.86
1.43	25.9	31.3	47.85	99	2.4	0.56	0.49	23.98	1.08
2.86	29.8	36.1	21.6	163.3	3.82	0.66	0.99	3.1	1.31
13.83	32	24.8	77.7	123.5	2.73	1.05	0.13	35	18.31
0.40	32.6	16.3	20.3	91.7	2.62	1.02	0.57	9.23	2.12
1.70	25.8	18.4	95.7	61.2	1.18	0.72	0.2	29.2	7.4
2.40	21.8	36.4	46.3	96.3	4.22	0.83	0.65	4.46	1.54
0.96	26	28.1	17.6	46.4	2.6	0.7	0.63	12.98	0.95
2.31	25.5	39.9	73.4	91.8	3.41	0.91	0.68	7.25	0.81
0.80	35.4	38	47.85	99	2.4	0.56	0.49	3.71	0.65
4.70	29.6	46.6	59.1	119.2	4.61	1.36	0.55	5.89	2.09
0.33	36	31.7	27	165.6	3.58	0.85	1.09	1.74	0.46
1.49	30.9	36.4	35.5	65.4	2.55	0.75	0.49	1.8	0.59
0.28	25.6	23.7	7.1	35.2	1.59	0.14	0.7	6.39	0.77
1.56	28.9	44.3	90.6	138.7	2.92	1.09	0.37	8.35	4.92
0.60	27.9	32	62.6	127.3	3.34	0.72	0.85	2.24	2.26

0.87	29.3	28.7	47.85	99	2.4	0.56	0.49	7.39	3.02
1.38	35.4	38	54.2	111.9	2.56	0.36	0.98	2.92	0.37
0.28	33.5	24.7	34.5	70.3	1.7	0.49	0.6	7.88	0.35
11.04	29	30.7	538.5	65.5	2.7	1.14	0.12	88.25	7.67
0.30	28.2	19.2	73.5	64.8	2.19	0.8	0.56	19.09	2.71
8.04	28.1	19	282.7	65.6	2.69	0.59	0.13	67.62	10.62
0.91	34.2	21.2	88	72	2.51	0.42	1.01	7.51	1.35
0.79	27.3	16.7	7.1	32.7	2.02	0.45	0.69	5.22	1.91
1.04	27	28.4	47.85	99	2.4	0.56	0.49	26.02	3.12
0.84	38.6	27.6	40.6	108.4	4.27	0.83	1.45	2.23	0.77
0.83	29.5	32.9	27.8	119.5	2.79	0.54	1.08	1.66	1.44
1.20	25.1	24.8	21.5	52.9	1.97	0.4	0.61	3.52	0.77
2.36	40.3	35.3	47.85	99	2.4	0.56	0.49	38.51	3.69
6.07	32.7	37.9	21.8	49.5	0.87	0.25	0.09	36.67	5.67
1.14	29.6	52.1	38.9	59.4	2.88	0.27	0.93	3.29	0.38
0.96	31.5	32.4	25.2	78.5	4.06	0.79	1.24	1.2	0.99
6.98	20.8	27.9	24.9	92.5	1.56	0.23	0.13	38.38	3.23
1.32	19	26.8	126.6	41.5	1.63	0.37	0.22	31.95	4.77
1.57	33.4	39.1	73	215.8	3.18	0.62	1.19	2.94	1.94
0.54	25.6	28.2	47.85	99	2.4	0.56	0.49	21.73	5.78
1.73	26.8	26.8	21.4	114.9	2.22	0.55	1.31	1.53	0.38
2.43	23.6	33.1	39	65	2.06	0.98	0.13	19.31	6.77
0.98	31.5	29.3	8.6	68	3.98	0.33	1.16	1.29	0.51
0.98	32.1	33	51	215.8	2.91	0.85	1.12	2.24	1.24
0.91	33.7	29.8	11.6	52	2.72	0.38	0.97	3.35	1.3
21.61	31.9	42.2	47.85	99	2.4	0.56	0.49	5.31	1.92
0.81	29.6	30	106.4	99.8	3.29	2.51	1.19	4.54	1.37
0.65	26.6	18.4	12.3	55.4	2.08	0.46	0.7	9.27	2.19
0.88	18.9	12.4	11.8	35.3	1.2	0.13	0.52	22.33	2.27
3.73	25.7	45.6	15.9	197.2	1.55	0.36	0.41	4.39	2.44
3.02	30.1	40.3	305.7	135.1	4.35	1.01	1.23	3.74	1.39
3.77	30.8	45	47.85	99	2.4	0.56	0.49	15.92	3.67
0.21	32.4	42.1	101.1	57.3	5.4	1.49	1.18	1.53	1.01
0.16	29.7	22.8	11.6	58	2.65	0.75	0.73	6.29	1.85
0.23	37.1	21.5	36.8	62.1	4.3	0.7	1.06	5.33	1.16
4.98	28.4	31.5	320.8	99.4	2.55	0.83	0.28	34.79	3.11
0.74	33.2	46.8	38.1	53	3.22	0.6	1.4	3.09	0.67
0.39	29.5	26.7	128.2	59.5	2.5	1.4	0.36	5.22	3.28

5.68	33.5	35.1	87.2	137.4	2.65	0.79	0.15	41.2	8.67
0.68	19.8	16.6	6.9	63.4	1.45	0.11	0.61	38.8	2.21
12.68	27	37.8	116.9	129.4	1.93	0.54	0.11	50	7.64
1.43	35.5	40.1	30.6	108.8	2.45	0.37	1.08	1.43	0.58
0.58	19.8	20.4	54	50.4	1.33	0.49	0.4	13.2	7.33
1.93	31.5	30.8	52	104.8	2.06	0.62	0.43	4.67	2.23
0.61	31.4	30.8	550.8	169.8	10.39	5.87	0.48	7.21	3.85
4.34	31.2	20.9	18.4	90.6	3.03	0.37	0.64	4.69	0.56
0.43	35.9	31.3	22.1	50.7	3.46	1.39	0.89	1.67	0.8
0.56	32.9	36.6	75.4	87.4	4.41	1.63	1.17	1.86	0.89
3.32	18.2	32.1	108.4	139.6	2.9	0.73	0.61	3.21	2.92
0.82	34.3	23.3	19.1	109.2	3.21	0.42	1.85	0.77	0.69
15.73	32.3	23.5	97.9	119.5	1.77	0.42	0.14	33.93	5.43
0.10	35	35.8	9.7	65.8	2.6	0.19	0.64	5.64	0.3
25.14	25.2	26.2	383.7	214.7	2.61	0.56	1	5.5	0.6
8.15	30.3	35.4	55.5	131.1	1.68	0.7	0.13	48.77	7.23
0.57	34.6	30.9	37	74.4	3.29	0.85	1.9	1.53	0.42
0.54	28	32.3	51.3	90.5	3.71	0.75	0.74	7.97	1.35
3.06	28.2	35.8	181.6	64.3	4.15	1.49	0.63	12.71	0.86
0.44	34.2	30.9	143.1	89.2	3.33	0.58	1.23	2.28	0.41
13.72	30.3	32.5	219.6	115.7	2.37	0.4	0.18	45.83	3.44
0.61	27.7	30.3	36.7	75.7	5.62	0.47	1.37	1.01	0.8
0.85	28.5	34.4	24	58.3	3.29	0.47	0.91	4.14	1.25
2.22	33.1	40.8	193.8	143.5	2.84	0.66	0.55	3.96	1.71
3.08	26.3	37	31.7	110.2	2.35	0.37	0.69	6.33	1.88
0.61	33.1	38.1	15.8	119.9	2.29	0.33	0.87	2.02	2.62
0.58	24.6	26.3	222.4	70.5	4.67	0.45	1.78	7.62	0.53
0.80	34.1	23.6	16.3	56.7	2.79	0.51	0.84	3.88	0.43
0.67	28.2	18.9	21	62.4	2.71	0.51	0.81	10.25	1.43
9.68	31.9	32.7	119.5	84.1	1.29	0.39	0.13	36.31	17
0.97	22.1	22.8	14.2	45.2	1.28	0.41	0.65	2.92	0.52
10.07	37.1	36.7	54.6	226.1	2.4	0.56	0.49	1.08	1.22
1.11	30.8	36.9	1820.8	276.3	5.8	2.27	0.65	13.82	2.06
8.60	34.9	42.3	47.1	106.5	2.71	0.42	0.23	20.26	5.48
1.03	34.2	32.5	9.7	104.5	2.96	0.74	1.31	0.92	0.37
2.40	38.5	41.4	49.7	174.3	3.56	1.5	0.95	2.04	1.42
4.56	32.9	45.6	117	80.2	2.4	0.56	0.49	10.41	4.57
0.81	35.9	31.4	104.1	91.6	2.6	0.53	0.85	2.05	0.98

0.32	35.7	41.1	25.3	80.9	2.44	0.12	1.14	6.35	0.54
0.43	35.1	48.9	38.8	97.6	3.72	1.23	0.81	6.05	1.11
0.23	31.7	24.8	15.6	53.2	2.57	0.36	0.88	1.69	0.74
1.18	30.5	51.5	47.85	99	2.4	0.56	0.49	3.45	2.9
0.44	25.9	19.3	9.8	48.9	2.4	0.56	0.49	12.16	1.43
0.28	29.1	33.8	106	70.7	2.93	1.56	0.73	4.41	1.07
1.01	23.1	38.8	7.7	50.7	1.15	0.04	0.22	10.32	4
0.72	31.6	22.2	14.9	65.7	2.86	0.77	0.81	5.12	1.1
0.93	25.5	22.3	5.1	128	1.92	0.28	0.79	1.11	0.46
13.36	31.1	50	84.3	168.6	3.02	1.49	0.17	22.06	1.65
2.51	18.4	19.8	146.9	40.5	2.4	0.56	0.49	7.39	2.45
0.35	28.5	33.5	84.5	70.8	6.53	1.24	1.29	2.1	2.01
0.61	37.8	33.9	22	77.3	4.49	1.08	0.59	20.19	1.15
11.34	37.3	32	2439.1	322.9	4.89	3.17	0.25	34.12	3.56
2.18	26.5	35.7	14.1	111.1	1.83	0.16	0.61	8.44	2.11
0.52	17.9	22.3	27.7	72.5	2	0.48	0.66	7.21	1.17
0.82	37.2	45.6	48.5	116.7	4.71	0.6	1.7	2.33	0.6
0.20	16.5	13.3	17.7	24.6	1.22	0.22	0.39	10.54	0.26
0.47	31.6	29.7	17.4	44.5	4.46	1.13	1.46	1.96	0.54
0.33	28.3	25.5	23.4	66.4	2.03	0.45	0.68	4.07	0.72
0.78	27.8	30	59.3	189.1	3.23	0.63	1.07	2.98	1.52
0.11	17.9	25.9	24.8	74.9	5.23	0.52	1.46	2.53	3.36
2.53	23	25.8	14.1	56.9	2.05	0.71	0.36	23.17	6
0.54	21.1	31.9	47.85	99	2.4	0.56	0.49	8.78	4.29
0.55	37.4	40.4	18.1	82.4	4.7	0.65	1.2	0.83	1.48
15.22	26.5	26.1	46.7	112.9	1.48	0.53	0.11	94.27	13.27
1.76	32	25.9	24.5	99.6	3.24	0.83	1.1	1.91	0.55
1.13	23.6	35.5	22	145.1	3.46	0.35	1.21	0.87	0.7
0.70	32.2	32.3	108.2	71.7	3.08	1.16	0.89	2.94	1.1
0.64	33.3	30.6	111.8	81.9	4.32	1	0.89	2.92	1.35
0.74	38.5	37.4	39.2	95.4	3.23	0.33	1.47	1.8	0.33
9.74	31	36.1	87.6	94	2.55	0.98	0.15	22.33	8.47
1.15	30.6	26	254	169	2.94	0.66	1.22	1.69	0.79
0.74	32.7	22.5	69.2	67.1	2.83	1.3	0.87	11.49	4.94
1.32	30.2	29.9	47.85	99	2.4	0.56	0.49	10.59	1.53
1.77	24	26.6	17.7	76.5	2.67	0.61	1.17	3.85	0.77
0.78	31.3	30.6	15.2	157	3.89	0.6	1.51	1.84	0.6
1.05	37.8	48.6	48.1	82	6.76	1.2	0.83	3.71	3.2

2.84	14.8	17.9	156.5	46.6	0.28	0.09	0.09	62.78	10.44
16.85	28	45.1	60.9	171.3	1.26	0.49	0.34	30.59	5.29
1.27	19.9	40.9	19.8	106.9	3.62	0.43	1.39	1.08	2.52
0.71	25.5	19.6	156.9	46.5	3.34	0.73	0.71	6.37	2.21
7.92	24.6	19.1	60.1	58.2	1.41	0.55	0.09	36.56	21.67
0.31	34	24.8	11.7	40.1	2.17	0.55	0.58	6.5	1.45
0.50	25.7	20.6	20.4	45.7	2.25	0.49	0.69	10.84	2.58
11.81	29.7	21	110.5	106.5	1.63	0.41	0.1	98	16.6
0.47	23.5	24.4	15.5	87	2.71	0.37	1.14	1.66	0.54
2.83	29	18.9	40.3	40	1.83	0.48	0.51	18.43	3.22
0.26	25.1	19.2	24.6	55.9	2.72	0.83	0.65	4.11	1.35
0.91	29.9	27.4	135.9	119.3	3.59	0.92	0.6	6.15	2.42
1.10	22.9	16	30.1	44.3	2.4	0.56	0.49	15.14	2.06
0.23	38.4	32.1	82.5	51.2	2.4	0.56	0.49	8.63	1.78
0.81	26.7	28.8	22.5	96.7	1.18	0.44	0.36	26.11	1.94
18.46	36.3	19.8	80.2	185.4	1.91	0.94	0.14	23.57	8.93
1.26	32	33.4	40	146.9	3.69	0.49	1.52	1.28	0.48
0.55	25.9	34.5	12.2	85.6	3.04	0.31	0.96	5.98	1.48
0.74	25.2	30.2	37.1	122.9	3.4	0.44	1.17	7.83	1
1.26	25.5	24.8	6.2	61.7	1.42	0.46	0.79	2.47	0.84
0.60	35.7	31.2	18.8	77.7	2.78	0.99	1.21	4.05	0.66
2.19	25.8	43.4	38.9	199.9	1.95	0.55	0.41	12.05	7
1.39	27.5	28	19.5	307.2	3.25	0.64	1.03	1.8	2.06
0.64	28.8	34.9	70.5	47	4.13	0.5	1.18	3.19	1.9
7.34	31	25	48.8	123.9	2.23	0.41	0.21	60.48	3.48
0.19	35.6	24.6	124.6	134.1	3.86	0.73	1.11	5.76	1.44
0.26	44.7	27.7	166.2	66.9	5.07	1.46	2.33	0.85	0.83
2.17	29.8	30.7	123.1	66.6	2.5	0.73	0.46	9.43	1.33
0.70	26.9	21	49.7	49.3	2.1	0.65	0.62	8.37	1.95
0.40	34.6	39.9	27.6	53.9	3.26	0.46	0.99	3.57	0.65
1.89	33.3	51.2	512.7	116.2	4.39	2.05	1.29	5.6	0.62
2.09	47.3	34.9	108	148.8	4.74	0.88	2.26	1.94	0.69
4.22	35.3	43.9	54.2	135.7	1.81	0.31	0.33	14.39	2.33
5.65	27.3	44.4	17.8	194.6	1.95	0.34	0.55	7.45	1.64
7.22	28.1	31.2	11.9	195.8	1.33	0.89	0.59	1.41	0.85
0.29	29.5	27.4	31.1	132.7	3.66	0.68	1.92	0.52	0.73
13.50	37.4	27.8	61.6	143.8	2.69	1	0.13	31.54	6.38
1.68	36.2	30.1	28.2	78.4	3.26	0.34	1.87	0.91	0.27

1.61	27.4	49.4	165.1	157.9	2.4	0.56	0.49	7.14	4.29
1.23	24.7	25.3	39.6	47.3	2.16	0.57	0.44	4.98	1.27
0.49	30.5	29.8	48.5	95.3	4.35	2.9	0.81	4.52	1.14
3.98	18.2	22.4	24.9	74.1	1.01	0.17	0.13	20.9	31.15
0.81	17.3	27.2	39.1	47.4	2.25	0.14	0.9	10.11	1.11
14.46	25.9	18.6	1429.4	46.3	3.76	2.38	0.26	11.54	0.77
0.73	39.7	28.1	10.5	55.4	2.62	1.01	0.59	10	2.03
1.18	34.4	37.8	228.1	95.2	3.56	0.9	0.85	1.55	0.65
0.87	28.4	33	59.9	142.5	3.6	0.42	1.45	3.17	0.41
0.41	37	32.2	14.9	79.2	1.95	0.43	0.67	7.58	2.16
0.09	30.2	24.6	17.2	83.7	2.18	0.34	0.8	19.13	1.75
0.47	28.8	27.6	14.8	80.1	2.23	0.22	1.06	3.87	0.75
3.18	29.6	29.7	47.85	99	2.4	0.56	0.49	4.29	0.82
0.12	31.5	30.7	24.2	76.6	4.13	0.75	1.34	5.15	1.57
0.63	28.2	19.7	32.3	87.9	2.15	0.3	0.52	14.62	4.81
9.91	31.4	33	47.85	99	2.4	0.56	0.49	5.31	2.65
0.51	18	39.3	12.3	96.6	1.85	0.28	0.88	7.39	1.36
1.25	22.8	21	17	41.5	1.55	0.24	0.98	9.18	1.43
0.79	23.7	38	9	118.8	3.36	0.51	1.3	1.55	0.75
1.94	23.7	27.2	104.9	92.1	2.38	0.74	0.42	12.86	1.67
0.20	32.7	26.6	44.9	84.2	2.65	0.85	0.94	3.3	1.06
0.53	25.9	21.6	55.5	49.6	3.24	0.64	1.4	3.14	0.5
3.25	25.5	39.8	31.6	156.8	1.72	0.59	0.4	9	6.25
0.32	40.1	30	33	93.9	4	0.52	1.18	1.36	1.44
0.29	28.7	23.7	24.7	53	1.77	0.21	1.08	3.98	1.39
0.51	23.6	45.9	11.7	111.7	4.2	0.55	1.23	0.98	1.14
0.47	29.3	34.4	47.85	99	2.4	0.56	0.49	14.14	0.9
4.12	19.6	21.6	880.8	110.5	1.78	0.96	0.15	59.6	5.27
17.63	22.4	31.8	47.85	99	2.4	0.56	0.49	31.24	2.18
0.13	28.1	19.3	13.2	59.3	2.69	1.62	0.71	10.96	3.28
10.64	27	14	37.1	268.6	0.73	0.3	0.1	89	13.2
3.26	13.8	27.2	47.85	99	2.4	0.56	0.49	24.63	1.59
19.96	20.3	13.7	42.3	34.5	2.4	0.56	0.49	34.29	2.65
15.12	28.2	36.6	40.8	99.9	1.15	0.34	0.12	60.17	12.33
6.29	29.3	37.8	19.2	106.8	3.13	0.45	0.35	10.74	2.57
6.96	31.6	26.1	47.85	99	2.4	0.56	0.49	8.43	1.29
0.68	25.2	30.4	23.7	71.5	1.6	0.32	0.53	5.04	1.51
9.81	28.6	35.1	67.9	107.2	1.12	0.29	0.08	84.38	15.5

9.08	25.7	24.6	125.4	67.4	5.06	1.23	0.2	47.2	4.15
1.24	26.9	32.5	18.9	42.3	1.6	0.4	0.56	29.7	1.13
16.58	33.4	25.6	184.7	41.5	1.3	0.39	0.29	22.41	2.07
18.19	32.4	29.8	162.7	112.1	1.83	0.82	0.13	54.54	5.85
4.08	31.2	16.9	47.85	99	2.4	0.56	0.49	7.45	1.41
26.55	27.3	29.3	58.1	145.4	1.53	0.71	0.1	98	10
1.02	25.3	37.8	24.2	105.9	2.55	0.53	0.56	10.66	1.18
2.60	13.3	23.5	47.85	99	2.4	0.56	0.49	16.16	5.47
13.73	16.8	55.4	96.3	61.8	1.57	1.86	0.09	76	3.89
9.92	31.4	39.2	31.9	115.4	2.45	0.59	0.15	32.8	11.2
8.46	29.2	35.1	241	178.3	2.89	0.68	0.14	74	11.5
3.39	26.5	30.5	90.8	102.9	1.33	0.78	0.65	20.31	1.69
15.73	30.6	29.7	445.1	69.4	2.42	0.78	0.11	96	5.27
15.42	33.4	20.3	22.3	44.3	0.98	0.45	0.07	76	12.43
18.33	27.4	31.5	22.7	172.6	1.24	0.43	0.08	47.38	6.63
9.61	16.7	19.6	21.9	168	0.91	0.31	0.07	38.43	4
11.45	24.2	31.2	74.7	53.3	1.58	0.63	0.09	53.78	10.56
24.93	28.5	30.4	75.2	173.3	1.98	0.92	0.32	32.19	7.5
11.48	34.4	38.7	122.7	174	1.46	0.45	0.12	30.25	12.75
0.89	30.2	28.2	90.3	133.3	1.5	0.75	0.22	76	8.68
7.15	32.6	24.6	114.8	56.6	1.85	1.16	0.07	79	16
2.56	30.4	40.2	50.7	41.9	0.95	0.09	0.2	98	7.95
11.57	30.5	18.5	507.3	127.1	2.36	1.27	0.11	78.8	13.18
15.58	19.8	43.6	84	82.3	1.92	0.45	0.16	91.81	4.75
1.60	31.2	39.3	25.7	76.6	2.41	0.32	0.88	5.56	0.59
11.47	26.7	28.2	87.2	209.9	2.32	0.78	0.13	22	9.62
4.65	24.9	34.2	57.8	120.4	2.01	0.38	1.25	4.96	0.72
8.57	29.9	33.3	38.7	151.7	1.38	0.42	0.33	17.88	0.55
1.43	32.4	34.8	32.5	222	1.83	0.5	0.38	9.71	5.26
1.30	24.4	33.1	42	97	2.03	0.36	0.73	4.33	2.66
5.48	26.2	30.1	31.1	96.4	0.89	0.29	0.16	34.75	0.94
4.81	20.7	47.2	15.2	128.7	2.03	0.27	0.34	9.06	2.44
6.32	22.2	42	7.2	50.1	1.69	0.52	0.44	4.55	0.91
18.80	25.3	32.7	101.8	341	1.6	0.69	0.1	48.3	12.5
2.39	29.2	27.6	8.7	94	3.08	0.47	0.67	7.64	3.93
9.47	20	48.5	30.5	111.1	0.89	0.3	0.32	16.56	3.75
0.66	22.2	24.1	18.3	51.4	2.4	0.56	0.49	13.92	3.1
0.76	30.1	29.5	75.1	90.5	1.49	0.77	0.3	49.93	5.77

21.39	29.3	47	118.6	102.8	2.22	0.76	0.15	31.33	7.4
3.05	19.7	26.6	120.2	96.4	2.23	0.23	0.35	19.77	4.51
23.18	26.3	43.7	34.3	200.8	1.87	0.55	0.67	5.37	1.04
22.78	25.8	43	312.1	124	2.4	0.56	0.49	16.47	2.63
27.45	32	28.8	52.9	122	1.65	1.23	0.13	35.54	8.15
1.86	16.3	18	19.6	136.1	1.94	0.24	0.34	19.26	2.79
3.35	28.5	32.3	104.6	170.8	2.05	1	0.09	60	9.22
21.57	25.2	48.8	323.7	145.6	1.86	0.64	0.5	22.16	2.46
2.47	24	38.8	20.5	69.7	2.24	0.29	0.39	13.77	1.72
14.19	36.2	18.6	225.1	135	3.55	1.06	0.16	52.69	11.38
0.67	22.6	12.4	17.6	38.5	1.5	0.51	0.33	55.06	6.03
0.30	37.3	23.7	13.4	83.4	3.48	0.68	1.08	1.06	0.54
0.39	39	28.4	45.6	81	3.64	1.31	0.63	1.75	0.92
5.04	16.8	12.4	145.5	201.3	1.25	0.39	0.09	21.11	5.56
2.20	9.6	17.4	3.8	25.8	0.91	4.37	0.15	14.67	21.47
7.06	26.4	19.8	8.9	72.3	0.7	0.22	0.15	148.33	7.07
18.82	29.6	41.3	16.2	142.2	2.4	0.56	0.49	7.35	0.20
6.01	18.6	18.5	98.3	225.2	2.48	0.72	0.08	47.38	5.38
0.48	29	18	71.2	109.1	0.51	0.22	0.17	30.59	3.06
11.13	28.1	19.7	47.85	99	2.4	0.56	0.49	12.59	1.37
11.88	21.6	15.8	46.3	58.4	1.21	0.35	0.07	117.29	15.29
0.93	37.8	40.9	21.9	105.9	2.02	0.45	1.22	1.82	0.92
1.42	31.5	39.5	129.5	146.8	2.15	0.43	1.02	1.94	1.23
1.03	42.6	48.9	12.2	77.1	2.38	0.43	1.05	1.90	0.48
0.81	30.6	42.5	40.1	146.5	2.97	0.76	1.14	2.04	0.39
20.32	25.6	29	88	127.7	2.61	1.18	0.12	60.42	11.75
1.13	20.3	40.9	40.8	103.6	3.01	0.63	1.28	3.20	0.94
4.88	22.4	51.1	21.6	100	2.06	0.71	0.65	13.54	1.60
1.94	25.2	40.9	25.8	158.4	2.79	1.18	0.62	4.84	0.48
22.15	33.6	40.2	74.3	128.7	1.84	0.94	0.17	31.76	4.12
2.46	28.3	35.1	9.2	79.5	2.1	0.46	0.89	1.12	0.67
7.79	24.6	52.3	157.9	301.1	4.26	1.59	0.16	54.38	8.56
0.82	32.2	34.5	35.4	71	4.69	1.21	0.69	6.77	1.55
0.67	29.4	31.5	21.6	107.7	3.52	1.08	0.6	3.42	0.87
0.67	26.3	40.2	7	80.1	1.21	0.28	0.48	1.52	0.79
0.98	28.4	36.3	57.6	183.5	2.57	0.9	0.62	1.89	1.69
1.59	28.2	36.1	37.3	239.6	3.01	0.54	0.96	2.27	1.38
2.98	34.2	30.4	21.1	68.6	2.4	0.56	0.49	5.16	1.86

11.83	30.2	34.1	356.7	161.1	2.99	0.99	0.13	23.54	11.15
1.35	23.4	36.8	13.3	109.7	1.98	0.45	0.9	1.02	0.84
9.62	17	19.5	58.7	69.2	2.28	1.67	0.09	229.44	29.56
2.13	26.6	43.8	277.3	102.2	2.63	1.27	0.53	5.64	2.28
14.78	35.3	20.8	162	132	3.41	0.7	0.21	28.57	2.10
33.85	33.5	26.3	8.1	12.3	2.13	1.08	0.02	263.50	145.50
1.26	25.9	24.9	19.6	79.1	2.19	0.51	0.85	1.71	0.56
19.91	30.5	35.4	171.4	213.1	1.97	0.45	0.13	86.92	7.85
1.43	25.1	45.9	182.6	201.1	3.44	0.93	0.99	3.64	1.86
0.32	24.2	28.7	10.7	72.2	3	0.83	0.51	2.35	1.35
0.69	22	28	13.7	51.7	2.98	1.28	0.69	2.64	0.19
1.17	29.5	23.4	79.6	66.2	1.69	0.24	0.61	3.82	1.31
4.60	20.4	35.1	45.9	152.1	3.21	0.82	0.18	6.89	3.22
0.33	32.2	20.4	47.85	99	2.4	0.56	0.49	3.71	2.10
0.80	29.8	29.8	19.8	47.5	2.88	0.51	1.03	15.90	0.68
1.45	28.1	26	47.85	99	2.4	0.56	0.49	8.76	1.08
0.84	32.9	30.1	20.2	77	2.12	0.33	0.95	1.61	0.39
0.72	28	31.2	16.9	55.5	2.2	0.52	0.93	2.47	0.69
0.49	20.5	42.5	20.9	114	2.94	0.54	0.86	3.23	3.57
1.43	28	41.8	27.4	105.1	2.81	0.67	0.67	1.90	1.01
1.56	28.4	40.4	3.5	68.2	3.1	0.6	0.48	7.35	0.54
22.51	26.1	31	23.5	53.8	1.44	0.43	0.11	27.27	5.36
3.12	29.1	33	16.9	41.7	2.01	0.43	0.41	5.76	1.54
6.90	31.1	37.3	154	186.9	2.26	1.33	0.14	44.86	9.14
0.67	25.4	40.2	21.7	73.4	2.99	0.21	0.59	5.05	1.69
0.33	30.6	32.6	37.1	85.2	2.4	0.56	0.49	15.16	4.63
0.84	28.5	20.2	20.4	70.4	4.26	2.6	0.32	22.19	1.91
0.63	29	43.5	145.7	105.4	1.78	1.64	0.15	6.40	6.00
18.86	29.5	17.6	166.4	175.4	1.39	0.39	0.1	101.50	3.80
1.23	21.4	22.7	132.1	146.4	2.72	0.16	0.64	7.70	0.97
0.51	25.7	17.3	12.6	40.2	1.87	0.23	0.52	13.23	1.35
0.41	25.5	25.7	47.85	99	2.4	0.56	0.49	3.55	2.78
3.87	20.1	34.6	36	138.6	1	0.22	0.26	41.12	1.46
1.91	23.2	43	187	128.3	2.74	0.67	0.54	5.61	2.19
1.26	31.4	27.7	91.9	93	3.44	0.42	1	1.57	0.66
1.02	28.8	33.8	19.6	100.2	3.69	0.51	1.21	2.07	0.64
12.65	33.4	22.6	253.3	105.7	1.73	0.36	0.26	11.69	8.00
1.58	19.2	43.2	20.2	71	1.27	0.53	0.1	128.40	5.40

0.94	24.6	36	39.4	79.3	6	0.59	1.33	3.23	0.99
0.46	29.2	25	34.9	86.3	3.05	0.96	0.74	5.85	0.47
0.07	19.7	41.6	47.85	99	2.4	0.56	0.49	6.98	1.04
4.77	28.6	35.9	47.85	99	2.4	0.56	0.49	10.71	2.29
9.80	20.3	34.6	50.9	202.9	1.26	0.47	0.09	104.33	16.00
3.42	24	38.8	8.5	42.1	1.53	0.84	0.12	52.92	8.33
1.62	25.8	33.9	47.85	99	2.4	0.56	0.49	5.82	1.37
2.09	30.2	50.9	9	61.5	2.51	0.34	0.61	2.89	1.56
2.41	22.9	35.4	19.6	172.4	2.39	0.43	0.53	4.49	1.79
1.86	21.8	31.5	21	125.3	1.2	0.37	0.19	38.05	8.16
12.17	28.2	24.6	222.1	178.8	2.11	0.35	0.13	56.08	4.62
9.46	20.5	23.6	47.85	99	2.4	0.56	0.49	11.49	1.61
3.46	36.6	23.8	59.4	80.7	2.81	0.54	0.51	9.88	2.20
2.53	31.4	32.8	47.85	99	2.4	0.56	0.49	1.90	2.55
0.89	33.6	16.1	14.7	50	1.67	0.15	0.6	23.13	1.12
2.87	30.2	22.8	23.8	101	2.69	0.34	0.59	1.58	0.75
1.49	21.2	24.4	47.85	99	2.4	0.56	0.49	14.20	1.47
1.31	30	35.1	18.8	71.8	2.62	0.81	0.71	2.65	0.73
0.44	33	23.4	18.1	48.7	3.22	1.01	0.62	3.66	0.48
1.11	26.7	22.1	11.1	115.2	1.46	0.26	0.75	1.75	0.92
0.44	29.1	19.8	47.85	99	2.4	0.56	0.49	7.14	2.45
0.88	29.4	23.8	16	71.2	2.57	0.32	0.73	2.85	4.37
5.97	28.2	22.1	204.1	284.1	3.82	1.11	0.18	73.44	10.00
7.64	29.6	35	47.85	99	2.4	0.56	0.49	5.84	1.29
0.38	26.8	17.2	36.8	50.2	2.45	1.25	0.59	4.41	1.81
14.93	27.5	26.4	100.3	121.6	1.61	0.72	0.1	53.00	4.50
0.95	30.7	30.8	53.8	136.9	3.32	0.6	1.02	2.36	1.25
5.11	36.6	45.3	1474.5	859.9	11.69	1.36	0.48	8.38	4.08
0.40	30.5	15.5	16.9	67.6	2.14	0.38	0.69	3.70	1.32
1.60	26.2	27.4	9.4	63.9	1.25	0.6	0.3	10.13	3.03
8.37	23.2	33.9	47.85	99	2.4	0.56	0.49	34.67	1.04
2.20	28.1	23.2	13.3	51.8	1.19	0.3	0.27	12.44	2.44
3.65	23.9	18.3	47.85	99	2.4	0.56	0.49	7.98	1.67
2.19	28.5	37.1	18.5	152.1	2.95	0.79	0.49	4.82	5.41
7.05	30.1	16.2	11.9	187	1.33	0.31	0.09	35.22	6.33
0.68	27.4	29.3	49.2	130.2	1.64	0.45	0.33	25.91	2.00
19.03	27.4	33.7	66	249.7	2.53	0.99	0.42	35.24	2.86
0.70	37.8	29.2	13.5	60.3	2.97	0.35	1.33	6.92	0.30

0.89	36.6	27.4	9.1	43.6	2.35	0.46	0.62	2.77	0.66
1.96	32.3	31.8	47.85	99	2.4	0.56	0.49	21.69	2.78
0.56	25.2	19.2	20.8	93.2	2.41	0.33	0.7	4.20	2.07
3.20	26.4	31.2	16.8	58.9	1.49	0.28	0.28	3.04	0.93
13.27	32.1	26	56.2	151.3	1.65	0.42	0.14	39.29	15.00
5.88	29.2	29.8	23.2	92.8	0.94	0.36	0.2	22.00	1.50
2.81	20.2	27.6	12.1	122.9	1.44	0.27	0.13	27.08	2.92
0.68	25.4	49.3	26.2	103.9	3.28	0.53	1.1	1.45	1.36
5.87	22.4	32.2	16.9	118	1.37	0.85	0.06	192.67	18.67
23.40	28.6	18.6	48.7	88.2	1.27	0.66	0.05	53.40	4.20
0.80	27.3	41.4	20.6	67.2	3.82	0.36	1.12	2.57	0.94
3.24	26.1	44.4	173.6	116.4	4.12	1.29	0.57	6.75	3.30
1.30	30.4	21.7	56.3	60.3	2.22	0.49	0.6	6.65	1.67
5.82	21.1	33.3	45.9	125.1	3.99	0.45	0.32	6.53	1.50
12.62	30.4	52.4	11.1	64.8	1.16	0.38	0.13	37.46	6.77
2.03	27.2	37.7	331.8	162.1	4.42	1.14	1.07	2.43	0.84
0.79	28.7	24.9	68.5	73.1	3.84	0.43	0.81	13.51	0.64
5.56	24.1	30.1	166.4	144.9	2.57	1.02	0.09	92.00	8.11
4.24	31.9	33.8	14.3	75.4	0.98	0.51	0.34	8.82	0.59
0.62	40.4	24.2	28.1	71.7	3.08	0.57	1.2	0.93	0.64
1.19	26.3	30.8	26.1	73	2.93	1.36	0.36	60.83	2.50
0.51	28.6	58.1	47.85	99	2.4	0.56	0.49	4.82	2.27
0.74	27	29.6	21.6	110	2.47	0.26	1.24	4.66	0.25
17.49	29.8	31.2	99.3	88.4	1.78	0.37	0.12	43.58	7.67
0.83	22.8	17.2	38	156.9	1.93	0.35	0.38	7.45	0.82
1.06	28	33.4	15.4	191.2	2.17	0.43	0.93	4.35	3.98
3.01	29.9	17.3	47.85	99	2.4	0.56	0.49	9.08	1.55
15.55	28.6	32.3	64.6	181.8	1.23	0.6	0.09	18.78	7.56
0.13	31.1	20.9	38.6	66.2	2.39	1.06	0.64	9.31	1.91
0.98	24.4	34.2	44.8	71.6	3.98	0.65	0.7	7.27	1.11
6.06	30.7	36.3	47.85	99	2.4	0.56	0.49	3.65	1.06
1.42	30.2	35.8	58.5	199.3	6.5	0.73	1.29	1.98	0.60
14.79	28.1	38	46.9	179.8	1.67	0.73	0.09	28.22	9.56
1.54	27.4	36.8	33.3	140.6	3.71	1.31	1.08	1.46	1.12
1.11	33	25.3	164.2	88.1	4.77	0.26	1.85	1.45	1.37
20.72	28.2	37.6	109.9	686.3	3.63	1.29	0.39	7.18	1.79
5.52	28.3	35.4	17.6	120.1	0.88	0.27	0.26	11.31	0.77
19.04	28.5	24.8	123.8	93.9	2.78	1.3	0.16	30.56	5.25

0.97	28.3	30.3	53.2	104	2.89	0.55	0.87	1.64	2.02
0.65	29.6	29.2	60.9	109.6	2.96	0.92	0.78	3.81	0.51
0.58	29.7	29.2	79	159.9	3.74	0.75	0.92	7.67	1.15
15.57	29.2	38.9	47.85	99	2.4	0.56	0.49	5.49	1.55
2.90	29.1	22.6	17	96.9	2.79	0.65	0.7	4.80	1.39
1.16	39.9	40	92.1	153.4	4.21	0.66	1.42	1.34	0.28
1.25	19.3	28.9	12.9	56.1	1.28	0.36	0.27	6.52	4.00
2.51	37.8	19.5	201.3	146.7	2.29	0.61	0.53	3.75	1.64
0.78	26.7	46.1	52.3	70	2.78	0.5	1.15	2.35	1.04
4.05	24.8	44.1	70.9	119.2	3.33	0.73	0.26	13.19	6.08
2.76	26.6	27.5	47.85	99	2.4	0.56	0.49	3.69	1.08
3.46	25.5	31.9	45.1	30.1	1.57	0.46	0.1	42.70	4.90
5.29	31.4	38.9	131.6	162.4	4.78	1.4	0.36	5.67	2.31
2.25	31.3	26.9	33.7	104.9	2	0.35	1.01	1.66	0.91
8.49	25.7	49.6	47.85	99	2.4	0.56	0.49	6.14	1.27
1.01	31	51.4	164.5	225.3	3.77	0.35	0.8	4.69	0.70
9.78	27.6	23.5	84.1	166.7	2.97	0.55	0.12	20.58	5.75
8.32	23.4	37.3	58.4	207.7	2.58	1.17	0.1	28.30	7.80
1.02	32	24.6	57.6	135.6	2.73	0.5	0.88	3.88	0.69
0.36	34.1	20.7	43.3	58.8	2.08	1.32	0.53	12.66	2.94
13.05	17.8	40.1	23.1	173.7	1.85	0.87	0.23	21.74	7.39
1.33	26	36.2	53.6	58.5	3.02	0.64	0.24	17.25	2.42
0.81	28.6	35.1	65.5	140.9	2.57	0.22	1.41	1.40	0.43
1.92	29.2	26.4	69.8	85.6	3.35	0.41	0.9	4.66	0.76
0.68	25.7	21	39.5	105.1	2.15	0.1	0.75	5.00	0.63
0.89	26.5	42.2	7.6	93	2.4	0.56	0.49	5.16	4.04
0.73	34.5	38.8	78.3	132.4	4.18	0.39	1.2	2.60	0.38
0.69	31.2	35.8	15	69.2	2.76	0.4	1.12	1.22	0.30
0.88	25.3	41.4	44.1	90.4	3.64	0.53	0.86	3.57	1.37
3.56	33.3	18.6	20.6	44.3	4.02	0.41	0.51	4.39	0.63
1.45	30.1	33.7	70.7	128.1	6.32	0.38	1.64	4.95	0.34
1.92	27	32.1	62.9	71.1	3.01	0.19	0.7	11.73	3.34
2.30	32.8	38	66.7	153	5.41	0.89	1.1	3.76	0.49
0.99	27.8	26.2	47.85	99	2.4	0.56	0.49	7.35	1.29
1.30	22	37.8	63.3	98.8	2.55	0.8	0.62	1.69	2.73
0.95	37	36.9	120.3	79.7	5.62	1.3	1.28	1.45	1.38
0.32	24.3	11.2	15.4	45.2	1.87	0.46	0.48	10.23	1.21
1.89	24.2	44.3	47.85	99	2.4	0.56	0.49	2.98	0.94

1.96	28.5	27.7	48.2	107.3	2.37	0.38	0.73	7.34	2.04
0.32	34.7	37.2	47.3	132.7	3.25	0.41	1.08	5.08	0.81
0.99	29.3	36.1	18.3	51	3.56	0.96	1.03	4.27	1.03
0.70	22.2	26.2	9.2	35.1	1.39	0.47	0.31	41.23	2.10
0.23	28.6	32.4	15.4	70.5	3.12	1.25	0.61	3.87	0.82
0.87	27.2	63.9	28.4	66.5	1.66	0.9	0.43	3.09	1.42
3.42	23.2	29.9	67.8	138.6	3.35	0.62	1.01	2.18	1.88
0.19	26.2	13.5	27.5	62.9	2.12	1.45	0.41	17.41	3.22
1.50	30.3	37.1	85.8	132.4	3.18	0.3	0.41	6.98	3.02
0.26	30.8	18.3	13	81.6	2.52	0.97	0.57	2.28	1.18
1.73	31.1	32.7	16.1	45.2	3.96	0.37	1.32	1.14	0.48
0.36	31.6	26.1	19.4	45.6	2.38	0.44	0.69	2.58	0.86
1.57	22.5	36.8	24.6	41.6	1.85	0.28	0.57	23.70	3.05
0.67	25.3	21.2	16.5	61.6	3	0.66	0.85	3.62	0.94
1.63	23.9	27.2	22.9	92.5	3.72	0.59	0.95	3.62	0.69
0.34	28.9	32.5	43.9	139.3	3.65	0.55	1.43	1.68	0.25
0.48	31.9	23.5	8.7	54.3	3.56	0.84	0.98	0.85	0.46
1.93	31.3	19	30.9	217.6	3.11	0.76	0.59	3.10	1.20
4.52	31.4	26.6	69.7	160.2	2.82	0.42	0.74	11.16	1.34
0.35	29.2	16.6	10.7	41.6	2.17	0.83	0.33	7.55	3.67
2.61	23.2	29.3	27.2	143.6	3.09	0.47	0.71	6.44	0.85
0.47	28.2	24.8	40.3	85.8	3.25	0.53	1	5.30	1.40
0.91	34.2	21.8	27.9	50.1	2.74	0.78	0.78	7.38	1.71
1.63	21.3	16	44	44.9	2.1	0.4	0.3	9.60	3.93
0.26	29.7	18.1	21.2	57.8	2.5	1.23	0.56	14.48	3.43
3.23	35.3	29.8	32.7	114.4	3.81	0.36	1.04	1.30	0.77
1.39	29.2	28.8	23.6	89.5	3.44	0.46	1.47	0.90	0.55
1.35	29.7	47.9	30.9	93	2.73	0.45	0.93	5.14	1.34
1.97	32.3	51.4	47.85	99	2.4	0.56	0.49	7.55	1.02
0.26	24	33.9	25.4	133	3.25	0.57	1.43	0.95	1.33
0.46	28.4	28	24.5	54.7	4.54	0.37	1.23	1.70	0.39
1.44	32.4	33	38	115.8	4.66	0.43	0.98	2.34	1.18
0.17	29.8	23.4	8.4	70.2	2.4	0.56	0.49	11.20	0.51
0.81	27.9	32.8	47.85	99	2.4	0.56	0.49	2.04	0.92
0.68	30.6	22.8	40.8	70.3	3.56	0.6	1.07	11.29	1.46
2.47	24.4	35.8	97.1	171.1	3.41	0.3	0.6	3.72	3.77
0.58	36.6	34.5	12.9	86.6	3.42	0.41	1.09	1.24	0.60
2.01	25	20.5	20.8	72.1	3.96	0.11	0.99	11.99	0.65

1.96	34.1	25.6	121.6	71.2	2.4	0.56	0.49	6.04	2.16
0.71	29	30.9	24.9	79.9	1.71	0.92	0.45	11.98	4.02
1.70	25.1	38.3	199.5	130.1	3.52	0.57	0.77	3.64	2.57
0.40	29.9	46.6	34.1	65.4	2.55	0.55	1.16	1.72	0.95
1.18	26	41.1	36.1	127.5	2.85	0.41	1.2	1.93	0.46
1.04	30.4	30.3	15.8	162	3.03	0.28	1.18	1.54	0.25
0.59	28.6	39.3	9	57.6	2.15	0.7	0.4	4.75	0.98
0.80	25.3	25.7	15.8	65.3	2.2	0.4	0.73	7.88	2.34
2.59	37.3	18.2	10	47.5	1.58	0.47	0.33	4.76	3.48
1.30	26.1	20.7	50.3	81.1	2.66	0.95	0.87	6.10	1.23
7.08	25.9	36.3	209.3	252.2	5.05	2.25	0.16	14.75	8.31
0.31	25.2	18.3	27.8	119.5	3.33	0.36	1.24	4.95	1.81
0.70	31.5	35.5	85.6	109.4	4.03	0.7	1.09	1.90	0.51
2.41	28	47.7	72.9	187.8	2.93	0.81	0.45	10.53	6.22
1.09	25.8	37.5	149.6	328.7	2.8	0.17	0.62	5.32	0.97
0.30	32.8	22	29.6	43.6	2.79	1.12	0.85	3.31	0.73
1.36	25.5	30.8	36.2	74.9	2.15	0.39	1.09	3.86	0.62
0.26	29.4	23	8.5	54.1	2.42	1.32	0.68	10.43	1.24
1.16	27	23.6	18.6	83.3	2.72	0.48	0.66	3.27	1.86
0.36	30.7	45.5	64.2	99.4	3.32	0.63	1.07	2.86	0.64
0.38	30.4	28.5	35.9	80.3	1.7	0.33	0.48	7.04	1.58
1.49	31.9	45.2	21.5	65.7	3.2	1.12	1.03	2.41	0.74
13.40	30.3	36.6	131.7	136.7	2.26	0.81	0.12	66.92	7.00
0.70	27.1	25.9	11.6	36	2.52	0.46	0.66	17.08	0.80
0.16	25.7	24.4	21.2	29.7	3.16	0.33	0.84	8.98	1.12
0.36	36.2	26.3	25.1	60.6	3.08	0.92	0.83	4.82	1.08
1.44	26.5	33.3	40.3	49.3	2.46	3.21	0.78	6.04	1.23
0.47	28.9	34.2	43.4	102.2	4.64	0.35	1.55	1.21	1.33
0.68	27.8	71.8	165	330.6	3.57	0.49	0.94	6.70	1.37
0.75	28.6	33.7	32.8	89.3	2.23	0.74	0.57	3.81	2.33
0.54	24.3	24.5	11.7	71.3	1.61	0.22	0.76	5.18	1.36
2.44	28.5	34.7	59.3	101.3	3.01	0.52	1.35	4.36	0.20
1.35	32.5	38.5	14.5	73.8	4.62	0.78	1.23	2.00	0.38
1.44	26.8	29.3	182.1	138.8	3.28	0.21	0.94	9.88	0.64
1.46	23	25.9	47.85	99	2.4	0.56	0.49	14.37	2.24
12.09	25.4	29.1	189.5	527.8	2.01	1.18	0.11	247.18	12.91
0.98	28.2	35	17.4	163.6	2.51	0.63	0.86	2.97	1.20
1.04	35.4	39.7	47.85	99	2.4	0.56	0.49	8.37	1.78

4.32	27.9	31.4	256.6	298.9	5.23	0.51	1.27	4.43	0.94
0.22	32	27.6	18.2	68.8	3.14	1.03	0.67	3.10	0.46
0.86	29.4	28.7	30	94.8	3.23	0.5	1.21	1.54	1.55
4.18	45.6	31.5	144.9	125.6	3.8	0.86	0.89	2.40	1.83
0.78	31.5	48.5	18.8	91	2.59	0.29	0.92	2.07	1.77
0.30	24.5	25.8	11.8	72.6	2	0.41	0.95	4.95	0.84
1.50	28.3	31.2	21.5	69	2.2	0.35	1.04	2.58	0.26
3.20	31	29.8	44.9	148.7	3.55	0.61	0.61	3.15	0.61
1.50	29.4	36	22.4	176.1	2.97	0.4	1.16	3.89	0.59
0.64	30.3	29.1	12.1	82.8	2.4	0.56	0.49	2.24	0.80
0.90	34.6	44.5	138.8	177.3	4.34	1.22	1.39	2.86	0.71
0.36	34.9	30.9	46.9	107.1	2	0.61	0.8	2.75	0.63
0.70	27.4	34.2	16.7	85.1	2.57	0.48	0.78	1.77	2.08
1.51	32.6	34.6	51.4	145.4	2.67	0.84	1.04	1.35	0.29
0.53	32.2	36.5	72.7	118.2	5.38	2.63	2.17	0.97	0.65
1.89	27	31.1	17.2	47.8	2.17	0.46	0.75	2.83	0.28
0.67	28	28.9	15.4	69.7	3.11	0.78	1.24	1.34	0.65
2.48	27.5	41.4	31.4	131.3	2.73	0.56	1.29	3.41	0.70
1.73	26	36.6	32.1	147.3	3.07	0.9	1.14	1.35	1.09
0.40	32.6	16.3	20.3	91.7	2.62	1.02	0.57	9.23	2.12
0.28	25.6	23.7	7.1	35.2	1.59	0.14	0.7	6.39	0.77
2.38	26.3	34.3	15.2	77.5	1.21	0.85	0.13	62.31	7.54
8.04	28.1	19	282.7	65.6	2.69	0.59	0.13	67.62	10.62
0.49	24.2	18	6.7	43.9	1.31	0.3	0.52	8.83	0.33
0.84	38.6	27.6	40.6	108.4	4.27	0.83	1.45	2.23	0.77
0.75	30.2	24.3	14	74.1	2.41	0.4	0.94	0.95	0.83
0.91	33.7	29.8	11.6	52	2.72	0.38	0.97	3.35	1.30
3.41	31.2	24.5	47.85	99	2.4	0.56	0.49	3.73	0.59
0.21	32.4	42.1	101.1	57.3	5.4	1.49	1.18	1.53	1.01
0.71	29.7	35.4	23.8	113.1	3.24	0.44	0.78	3.87	0.62
1.49	28.7	30.9	75	70.6	2.69	0.81	0.73	1.36	1.11
0.66	23.8	26.4	9.9	56.7	3.73	0.83	1.48	3.78	0.74
1.73	32.9	30	96.5	171.2	2.5	0.42	0.68	3.54	0.79
0.26	31.9	40.2	13.3	47.9	2.6	0.88	1.04	1.10	0.29
0.55	26.4	41.8	87.3	127.1	2.99	0.6	0.51	6.53	4.55
1.95	23.7	41.6	10.3	69.8	2.22	0.37	0.56	4.02	1.63
0.54	28	32.3	51.3	90.5	3.71	0.75	0.74	7.97	1.35
0.84	31.5	52.5	30.8	100.8	3.13	0.42	1.48	3.24	0.37

0.73	30.3	26.2	17.9	78.3	2.27	0.74	0.84	4.94	0.45
1.34	28.1	28.9	5.9	97.7	2.4	0.56	0.49	4.06	1.33
0.90	31.6	30.9	63.6	67.5	1.84	0.73	0.67	4.48	1.94
0.44	25.9	19.3	9.8	48.9	2.4	0.56	0.49	12.16	1.43
1.94	31	25.8	54	142.7	3.97	0.59	1.02	1.74	1.10
0.53	30.6	13.3	90.6	210.9	1.55	1.08	0.33	5.18	1.82
0.43	32.9	31.2	34.9	99.3	3.99	0.97	1.05	1.97	1.19
0.23	38.4	32.1	82.5	51.2	2.4	0.56	0.49	8.63	1.78
0.29	34.8	29.7	12.3	99.4	2.62	0.67	0.9	1.24	0.51
1.26	25.5	24.8	6.2	61.7	1.42	0.46	0.79	2.47	0.84
2.10	26.7	28.5	40.5	87.1	3.37	0.61	0.98	3.78	1.41
0.90	29.4	32.3	42.9	114.4	2.1	0.68	0.37	15.73	2.19
0.54	27.2	40.9	23.4	86.8	3.72	0.57	0.82	3.66	0.61
0.54	35.1	43.7	47.85	99	2.4	0.56	0.49	1.65	1.35
0.40	34.6	39.9	27.6	53.9	3.26	0.46	0.99	3.57	0.65
1.02	21.2	15.4	2.3	23.7	1.03	0.17	0.34	18.71	4.94
2.14	26.7	36	30.4	113.9	1.82	0.47	0.77	2.14	0.19
5.42	26.9	65.3	89	156.7	3.44	0.63	0.39	10.23	1.67
0.47	28.8	27.6	14.8	80.1	2.23	0.22	1.06	3.87	0.75
0.79	31.8	29.7	47.85	99	2.4	0.56	0.49	3.47	2.24
10.64	27	14	37.1	268.6	0.73	0.3	0.1	103.50	13.20
12.85	32.8	19.4	31.1	136.1	1.54	0.33	0.84	3.45	0.83
5.28	25.1	24.6	12.5	85.4	1	0.39	0.1	64.30	9.00
9.92	31.4	39.2	31.9	115.4	2.45	0.59	0.15	32.80	11.20
6.12	21.7	18.2	34.9	51.1	1.16	0.69	0.13	143.92	16.69
12.20	29.4	16.5	35.4	46.1	1.72	0.7	0.11	21.00	1.36
18.43	22.8	34.4	129.1	380.5	2.3	0.94	0.31	68.71	5.16
24.15	27.6	23.3	83.1	120.4	1.51	0.53	0.09	83.67	12.00
0.89	30.2	28.2	90.3	133.3	1.5	0.75	0.22	116.09	8.68
11.47	26.7	28.2	87.2	209.9	2.32	0.78	0.13	22.00	9.62
4.65	24.9	34.2	57.8	120.4	2.01	0.38	1.25	4.96	0.72
18.80	25.3	32.7	101.8	341	1.6	0.69	0.1	48.30	12.50
32.16	31.6	33.1	52.9	563.4	2.18	0.66	0.17	35.29	6.47
18.91	22.9	22.3	18.3	86.2	1.17	0.45	0.29	5.52	0.69
0.47	34.4	20.7	33.4	115.6	2.4	0.56	0.49	13.02	0.69
1.86	16.3	18	19.6	136.1	1.94	0.24	0.34	19.26	2.79
0.30	37.3	23.7	13.4	83.4	3.48	0.68	1.08	1.06	0.54

lhr1	MHR	mhr1	PHR	LDL	WBC	NE #	LY #	MO
2.00	0.37	1.00	351.85	2.62	14.8	13	1.3	0.2
2.00	1.22	2.00	105.41	2.67	6	4.9	0.8	0.9
2.00	0.67	2.00	373.33	0.54	5.3	4.5	0.7	0.2
3.00	0.67	2.00	256	0.43	6.56	2.2	3.22	0.1
3.00	0.42	2.00	312.5	0.17	9.7	6.12	3.16	0.1
3.00	1.72	3.00	272.41	0.67	11.36	9.34	1.36	0.5
2.00	0.2	1.00	82.45	1.14	13.03	10.89	1.9	0.1
2.00	0.61	2.00	302.04	1.14	20.12	17.22	1.44	0.3
2.00	0.61	2.00	175.51	1.14	8.12	5.47	1.57	0.3
3.00	1.11	2.00	448.89	0.39	4.97	4.11	0.67	0.1
2.00	8.89	3.00	215.56	1.14	2.26	1.32	0.65	0.8
2.00	0.61	2.00	410.2	1.14	10.28	7.77	1.98	0.3
2.00	1.22	2.00	121.22	1.14	21.42	20.03	0.7	0.6
2.00	1.02	2.00	208.16	1.14	11.54	8.81	1.93	0.5
2.00	0.84	2.00	116.33	1.14	8.51	7.41	0.54	0.41
3.00	0.82	2.00	283.67	1.14	13.59	8.74	3.21	0.4
1.00	0.79	2.00	231.75	1.71	8.74	8.02	0.52	0.5
2.00	0.61	2.00	115.1	1.14	11.22	10.42	0.59	0.3
1.00	3.33	2.00	424.44	1.14	3.82	3.6	0.1	0.3
2.00	0.41	1.00	118.37	1.14	14.41	12.8	1.2	0.2
3.00	3.75	3.00	412.5	0.36	4.9	3.6	0.9	0.3
3.00	4.55	3.00	1463.64	1.49	7.4	6.2	0.7	0.5
2.00	2.67	3.00	360	1.03	13.99	13.11	0.4	0.4
1.00	1.6	3.00	85.6	0.76	5.72	4.07	0.22	0.4
3.00	2.15	3.00	953.85	0.03	16.7	13.93	1.67	0.28
2.00	1.18	2.00	1000	0.13	5.86	5.2	0.52	0.2
3.00	2.14	3.00	614.29	0.11	30.89	27.7	4.82	0.3
2.00	0.82	2.00	94.69	1.14	7.22	6.17	0.67	0.4
3.00	0.83	2.00	336.67	0.68	32.35	28.61	1	0.1
3.00	4.67	3.00	281.33	0.98	8.37	6.99	0.72	0.7
3.00	1.48	2.00	237.04	1.56	8.84	6.69	1.45	0.4
3.00	1.48	2.00	216	8.88	7.51	3.69	2.79	0.37
1.00	1.79	2.00	192.86	1.06	4.03	2.71	0.73	0.5
1.00	1.56	2.00	70.63	0.42	5.23	4.48	0.25	0.5
1.00	0.66	2.00	27.02	1.73	1.63	0.94	0.51	1
2.00	0.89	2.00	108.89	0.98	2.4	0.9	1.2	0.4
3.00	1.21	2.00	209.09	1.37	5.1	3.1	1.6	0.4

2.00	1.43	2.00	69.8	1.14	4.7	3.5	0.8	0.7
3.00	2.5	3.00	500	1.72	8.1	5.7	1.9	0.7
3.00	3.33	3.00	675	1.1	14.4	10.3	1.5	0.4
2.00	0.61	2.00	112.24	1.14	4	3.2	0.6	0.3
3.00	5.83	3.00	830	0.29	10.8	9.5	0.7	0.7
2.00	0.54	2.00	148.21	1.65	6.5	4.6	1.4	0.3
2.00	2.94	3.00	676.47	0.17	17.61	15.04	0.71	0.5
2.00	1.58	2.00	248.42	0.45	8.6	7.9	0.3	0.3
3.00	2.5	3.00	1650	1.05	9.75	7.25	1.41	0.3
2.00	1.22	2.00	98.78	1.14	14.6	12.9	0.74	0.6
2.00	1.9	3.00	268.1	0.63	6.13	5.1	0.45	0.4
2.00	0.27	1.00	79.28	1.07	7.9	4.2	1.4	0.3
2.00	0.61	2.00	159.18	1.14	15.41	13.9	0.9	0.3
3.00	1.08	2.00	117.22	0.34	14.81	12.4	1.6	0.39
3.00	5	3.00	322.86	0.22	7.8	6.5	0.7	0.7
3.00	6.5	3.00	130	0.61	4.7	3.2	0.9	1.3
2.00	0.51	2.00	50.26	0.21	17.32	15.63	0.63	0.2
1.00	0.82	2.00	82.04	1.14	7.6	6.8	0.4	0.4
2.00	0.42	2.00	24.23	1	5.2	2.7	2	0.3
2.00	1.11	2.00	98.89	0.21	7.82	7.24	0.36	0.3
1.00	0.95	2.00	117.14	1.1	5.3	4.8	0.2	0.4
1.00	0.16	1.00	50	1.27	5.6	4.1	1.2	0.2
1.00	1.58	2.00	130	0.22	3.92	3.6	0.17	0.3
1.00	0.32	1.00	104.84	1.65	3.42	3	0.3	0.2
1.00	2.63	2.00	74.74	1.14	4.2	3.6	0.4	0.5
1.00	0.22	1.00	36.5	1	3.42	2.3	0.6	0.3
3.00	1.9	3.00	385.71	0.22	15.41	12.9	1.1	0.4
2.00	1.03	2.00	128.28	0.73	5.5	4.6	0.5	0.3
2.00	1.18	2.00	17.65	0.79	2.8	2	0.5	0.6
2.00	0.96	2.00	113.46	0.51	3.02	2.3	0.5	0.5
1.00	0.68	2.00	76.61	1.01	1.72	1.1	0.3	0.4
3.00	6.36	3.00	2036.36	0.96	14.81	12.5	1.1	0.7
3.00	3.33	3.00	733.33	2.07	11.78	9.38	1.51	0.4
3.00	2	3.00	970	0.64	6	4.3	1.3	0.2
2.00	0.7	2.00	105.12	0.45	17.01	15.9	0.5	0.3
1.00	0.95	2.00	197.3	1.16	3.54	3.11	0.21	0.7
1.00	0.19	1.00	38.54	1.47	5.64	3.57	1.47	0.3
2.00	2.8	3.00	800	1.42	5.7	4.7	0.6	0.42

1.00	0.48	2.00	48.65	1.64	2.51	1.67	0.43	0.6
2.00	0.58	2.00	85.51	3.1	6.45	4.67	1.07	0.4
3.00	4.55	3.00	1563.64	0.59	12.74	9	1.3	0.5
1.00	0.37	1.00	87.52	1.19	3.58	2.38	0.63	0.4
1.00	0.13	1.00	167.95	0.43	2.12	1.36	0.3	0.1
1.00	0.41	1.00	86.12	0.61	5.93	5.55	0.24	0.2
1.00	0.92	2.00	70.67	0.92	2.11	1.43	0.4	0.55
1.00	0.21	1.00	30.48	1.44	4.1	3.12	0.45	0.3
3.00	6.83	3.00	1033.33	0.21	12.44	9.91	1.18	0.41
2.00	0.82	2.00	220	1.14	4.07	2.53	0.91	0.4
2.00	0.2	1.00	153.06	1.14	9.48	8.44	0.88	0.1
1.00	0.37	1.00	41.11	1.72	2.14	1.28	0.68	0.4
2.00	2.14	3.00	210	1.09	12.58	11.5	0.54	0.3
2.00	0.94	2.00	147.36	0.69	6.28	3.82	1.91	0.5
3.00	3.33	3.00	575.56	0.34	5.02	4.42	0.48	0.3
2.00	1.67	3.00	79.52	1.64	10.28	8.83	0.64	0.7
3.00	3.64	3.00	1020.91	0.52	8.01	5.22	1.93	0.4
3.00	4.12	3.00	402.35	0.59	7.12	4.76	1.37	0.7
3.00	5.71	3.00	395.71	0.77	6.02	4.35	0.96	0.8
2.00	0.53	2.00	72.11	0.79	2.52	1.88	0.43	0.2
1.00	0.34	1.00	32.08	1.2	3.13	2.24	0.58	0.34
3.00	2.73	3.00	1400	0.41	9.23	7.01	1.47	0.3
3.00	27.5	3.00	1300	0.14	7.14	6.11	0.77	1.1
2.00	1.87	3.00	149.33	0.73	3.89	3.08	0.55	0.28
2.00	1.36	2.00	745.45	1.69	11.92	9.96	0.71	0.3
2.00	0.71	2.00	185.71	2.04	10.15	7.27	1.68	0.3
2.00	1.22	2.00	108.98	1.14	6.87	5.96	0.73	0.6
2.00	1.02	2.00	171.43	1.14	6.44	5.04	0.64	0.5
3.00	3.42	3.00	386.67	0.69	5.13	3.66	1.11	0.41
2.00	0.56	2.00	100	1.13	6.02	4.89	0.76	0.3
1.00	0.21	1.00	54.36	1.57	3.76	1.32	1.26	0.4
2.00	1.02	2.00	80.41	1.14	5.66	4.15	0.77	0.5
3.00	1.02	2.00	162.04	1.14	8.51	5.35	2.1	0.5
2.00	109.52	3.00	371.43	2.02	7.17	6	0.44	23
3.00	3.13	3.00	743.75	0.8	7.69	4.81	1.25	0.5
3.00	2.31	3.00	211.54	0.69	8.78	4.45	2.51	0.6
3.00	23.5	3.00	4220	0.02	10.32	5.27	2.91	0.47
3.00	2.73	3.00	443.64	0.64	4.38	2.5	1.42	0.3

3.00	0.74	2.00	501.48	1.07	4.33	2.12	1.47	0.2
3.00	3.33	3.00	311.11	1.21	5	2.41	1.89	0.6
3.00	1.36	2.00	115.45	1.04	4.38	2.87	1.02	0.3
1.00	0.35	1.00	136.32	1.33	5.85	3.75	1.04	0.4
3.00	2.22	3.00	663.33	0.32	9.18	5.29	2.6	0.2
1.00	0.31	1.00	54.59	0.79	2.29	1.45	0.48	0.26
3.00	3.85	3.00	626.15	0.73	13.91	11.3	1.02	0.5
3.00	1.36	2.00	195.71	0.53	6.49	4.82	0.91	0.19
2.00	0.37	1.00	143.21	1.18	5.62	3.59	1.54	0.3
1.00	0.19	1.00	24.44	2.63	4.26	3.96	0.16	0.2
1.00	0.28	1.00	43.93	0.91	2.94	1.52	1	0.3
2.00	0.47	2.00	306.41	1.28	11.27	8.32	1.8	0.3
3.00	0.87	2.00	314.78	1.04	4.38	2.37	1.43	0.2
2.00	0.65	2.00	70.2	1.14	3.48	1.82	1.03	0.32
3.00	3.57	3.00	238.57	0.84	4.92	3.78	0.73	0.5
2.00	1.09	2.00	200	0.69	2.16	1.67	0.49	0.35
2.00	0.34	1.00	96.1	2.25	8.57	5.16	2.44	0.4
2.00	0.14	1.00	103.47	0.82	4.68	2.53	1.45	0.1
2.00	0.39	1.00	67.11	2.4	4.38	2.74	1.19	0.3
2.00	0.82	2.00	56.94	0.62	3.28	1.64	1.2	0.7
3.00	1.74	3.00	536.84	0.54	13.94	10.37	1.57	0.33
2.00	0.2	1.00	103.4	3.31	7.49	3.02	2.72	0.3
3.00	4.44	3.00	633.33	0.33	5.28	3.45	1.36	0.4
3.00	3.13	3.00	868.75	1.29	7.78	5.85	1.15	0.5
2.00	0.88	2.00	63.82	1.28	4.65	2.09	1.93	0.6
2.00	1.19	2.00	61.69	0.86	2.58	1.77	0.78	0.7
3.00	2.08	3.00	451.67	0.7	8.45	5.43	1.67	0.5
2.00	0.48	2.00	59.69	0.86	2.93	1.4	1.17	0.31
2.00	1.04	2.00	80	0.69	4.36	2.91	0.8	0.5
3.00	1.67	3.00	286.67	0.63	4.97	3.84	0.61	0.2
2.00	0.71	2.00	64.82	0.48	8.92	7.06	0.74	0.4
2.00	1.43	2.00	738.1	3.48	19.75	17.15	0.89	0.3
3.00	1.43	2.00	457.14	0.32	10.72	8.86	0.8	0.2
3.00	6	3.00	284	0.56	10.35	8.12	0.87	0.6
3.00	5	3.00	167.5	0.27	11.53	9.67	1.11	0.4
2.00	0.2	1.00	140.82	1.14	3.4	2.52	0.49	0.1
2.00	0.45	2.00	77.61	1.46	2.21	1.27	0.68	0.3
3.00	1.67	3.00	845	0.38	5.2	4.18	0.61	0.2

1.00	0.42	2.00	96.67	1.92	4.26	3.53	0.26	0.2
3.00	2.73	3.00	201.82	0.33	3.88	3	0.59	0.3
1.00	1.65	3.00	58.04	1.21	5.66	3.72	0.87	1.6
2.00	5.56	3.00	655.56	0.27	5.85	5.13	0.28	0.5
3.00	5	3.00	780	0.26	12.51	8.64	2.21	0.4
1.00	0.74	2.00	186.05	2.18	5.94	4.79	0.61	0.6
2.00	0.65	2.00	195.09	2.09	6.45	3.72	1.68	0.7
2.00	0.25	1.00	32.83	2.13	8.35	4.31	2.93	0.3
2.00	0.48	2.00	154.84	3.72	5.41	3.72	0.88	0.3
2.00	0.58	2.00	81.54	0.54	3.12	2.31	0.63	0.3
2.00	0.39	1.00	33.4	1.13	4.62	2.82	1.16	0.4
3.00	1.1	2.00	351.02	1.14	11.28	7.43	2.27	0.54
1.00	0.47	2.00	100	2.59	3.4	1.62	1.09	0.7
3.00	13.33	3.00	683.33	0.49	2.66	1.69	0.84	1.6
2.00	1.38	2.00	200	1.02	12.09	10.48	0.78	0.4
3.00	2	3.00	474	0.42	9.51	7.17	1.34	0.2
2.00	0.78	2.00	195	2.71	8.1	7.1	0.61	0.25
1.00	0.44	2.00	43.3	1.82	9.94	8.88	0.75	0.4
1.00	0.44	2.00	39.65	0.87	1.04	0.39	0.44	0.5
3.00	5	3.00	645.71	0.79	8.34	6.19	1.23	0.7
2.00	0.44	2.00	44.41	0.51	2.57	1.45	0.82	0.3
3.00	1.33	2.00	600	0.7	2.44	0.96	0.9	0.2
3.00	3.64	3.00	2418.18	0.5	11.57	9.34	1.12	0.4
2.00	0.61	2.00	15.1	1.14	2.71	1.68	0.48	0.3
2.00	7	3.00	514	0.61	11.49	10.15	0.38	0.7
2.00	1.32	2.00	231.58	2.3	6.05	4.85	0.67	0.5
3.00	2.6	3.00	753.33	0.84	7.85	5.68	1.43	0.39
3.00	3.04	3.00	252.17	0.83	11.36	7.99	1.42	0.7
2.00	0.61	2.00	133.44	1.67	6.09	4.93	0.62	0.39
2.00	3.92	3.00	1023.08	1.08	4.7	4.05	0.44	0.51
3.00	1.88	3.00	121.25	1.21	6.49	4.7	1.02	0.3
2.00	0.97	2.00	60.97	0.55	7.43	5.45	0.92	0.7
3.00	8.33	3.00	690	0.21	6.15	4.43	1	0.5
2.00	0.8	2.00	180	0.62	10.62	9.03	1.23	0.4
2.00	2.22	3.00	202.22	0.67	3.78	2.56	0.69	0.4
3.00	4.27	3.00	526.67	0.26	20.18	16.93	1.66	0.64
2.00	0.58	2.00	93.46	0.97	8.62	6.88	0.7	0.3
2.00	1.36	2.00	247.27	1.27	7.43	5.73	0.93	0.3

2.00	0.41	1.00	242.86	1.14	6.89	4.8	1.34	0.2
3.00	3.33	3.00	525	0.58	1.83	0.94	0.59	0.4
2.00	0.97	2.00	70.32	1.28	2.13	1.22	0.57	0.3
3.00	2.35	3.00	486.47	0.72	4.97	3.45	0.81	0.4
3.00	1.82	3.00	572.73	0.76	3.92	2.41	0.58	0.2
2.00	0.82	2.00	59.67	0.64	36.21	31.54	2	0.5
1.00	0.72	2.00	63.92	2.11	9.46	8.47	0.34	0.7
3.00	0.41	2.00	451.23	3.32	13.5	8.89	3.23	0.3
2.00	1.84	3.00	132.65	1.14	3.84	1.74	1.36	0.9
2.00	0.16	1.00	35.19	1.05	4.19	2.28	1.57	0.2
2.00	0.8	2.00	72.73	1.56	5.64	3.27	1.71	0.7
3.00	2	3.00	604	0.79	6.55	4.36	1.43	0.3
2.00	0.97	2.00	300	0.67	8.32	6.57	1.31	0.3
2.00	0.41	1.00	60	1.14	5.28	3.99	0.74	0.2
1.00	0.3	1.00	52	1.85	2.39	1.57	0.66	0.3
3.00	2.31	3.00	361.54	0.85	6.35	3.04	2.08	0.6
3.00	3	3.00	3210	0.41	14.45	12.84	0.54	0.3
1.00	0.61	2.00	98.78	1.14	10	9.33	0.3	0.3
2.00	0.8	2.00	428	1.25	8.14	6.62	0.91	0.2
1.00	0.29	1.00	31.01	1.22	1.47	1.11	0.22	0.2
3.00	3.1	3.00	374	0.54	17.45	16.05	0.66	0.31
1.00	1.35	2.00	66.76	1.75	5.08	4.33	0.35	1
2.00	0.33	1.00	106.56	0.73	13.04	10.42	1.74	0.2
3.00	1.88	3.00	208.75	1.3	4.75	3.25	1.22	0.3
2.00	0.2	1.00	214.29	1.14	4.4	3.42	0.51	0.1
3.00	3	3.00	860	0.43	7.44	4.9	1.66	0.3
2.00	0.81	2.00	102.7	0.71	2.35	0.8	1.23	0.6
2.00	0.61	2.00	163.27	1.14	1.87	0.73	0.78	0.3
2.00	2.36	3.00	221.82	0.36	2.99	2.24	0.35	0.26
3.00	4.29	3.00	785.71	0.2	7.69	6.56	0.66	0.3
2.00	0.19	1.00	396.3	1.37	17.76	15.38	1.38	0.1
3.00	3.75	3.00	1662.5	0.65	11.01	7.95	1.87	0.3
2.00	3.75	3.00	825	0.28	2.39	2.05	0.25	0.3
1.00	0.41	1.00	13.61	1.21	1.93	1.16	0.58	0.6
2.00	2.31	3.00	1192.31	0.82	12.98	11.71	0.45	0.3
1.00	0.41	1.00	132.65	1.14	5.33	4.53	0.41	0.2
2.00	0.31	1.00	131.25	0.73	20.43	17.02	1.17	0.2
2.00	1.15	2.00	394.23	1.91	7	3.8	1.4	0.6

2.00	1.92	3.00	143.85	0.98	12.69	10.45	1.04	0.5
3.00	6.25	3.00	405	0.62	6	4.65	0.82	0.5
3.00	3.64	3.00	167.27	0.36	10.72	8.96	1.21	0.4
2.00	1.57	2.00	90.61	1.14	2.23	1.16	0.77	0.77
3.00	0.61	2.00	142.86	1.14	14.29	8.51	3.55	0.3
3.00	3.33	3.00	783.33	1.99	7.15	4.81	1.31	0.6
3.00	4.55	3.00	654.55	0.67	3.7	2.6	0.62	0.5
3.00	3.18	3.00	577.27	1.37	6.89	5.23	1.22	0.7
2.00	0.38	1.00	186.11	1	4.52	2.34	1.64	0.27
2.00	0.2	1.00	88.57	1.14	8.71	5.63	0.79	0.1
3.00	5.45	3.00	1927.27	0.45	10.92	8.76	1.34	0.6
2.00	0.2	1.00	150.98	1.68	7.05	5.04	1.12	0.1
3.00	2.11	3.00	352.63	1.69	6.11	4	1.08	0.4
3.00	2	3.00	560	2.13	7.37	5.45	0.99	0.3
2.00	1.04	2.00	59.17	1.35	3.09	2.1	0.69	0.5
2.00	0.33	1.00	122.33	1	15.02	13.88	0.67	0.2
3.00	3.57	3.00	878.57	0.72	9.51	7.22	1.66	0.5
1.00	0.53	2.00	86.42	1.01	7.45	6.27	0.72	0.43
2.00	0.47	2.00	185.94	0.85	6.5	4.63	1.14	0.3
1.00	1.02	2.00	39.32	0.72	1.71	0.93	0.44	0.6
2.00	1.25	2.00	300.63	1.3	3.3	1.96	0.59	0.2
2.00	0.51	2.00	108.47	0.83	4.45	2.01	1.95	0.3
3.00	2.73	3.00	1136.36	0.52	24.06	22.06	0.79	0.3
2.00	1.02	2.00	84.49	1.14	8.17	6.96	0.72	0.5
2.00	1.25	2.00	812.5	1.21	11.18	9.4	0.83	0.4
3.00	0.71	2.00	311.9	1.45	18.36	11.66	3.38	0.3
2.00	1.02	2.00	151.02	1.14	5.05	3.47	0.88	0.5
1.00	0.28	1.00	60.19	2.83	5.53	4.1	0.76	0.3
2.00	0.61	2.00	143.67	1.14	7.69	5.52	1.27	0.3
2.00	2.35	3.00	200	1.43	12.05	10.75	0.56	0.4
2.00	0.82	2.00	538.78	1.14	29.06	25.13	1.45	0.4
2.00	0.43	2.00	234.78	1.02	11.75	10.7	0.47	0.2
3.00	3.85	3.00	315.38	0.75	8.33	6.62	0.61	0.5
2.00	1.07	2.00	94.48	1.12	6.11	4.95	0.56	0.31
3.00	0.2	1.00	266.53	1.14	24.67	18.75	3.21	0.1
3.00	2	3.00	407	0.65	5.9	4.31	1.13	0.4
3.00	4	3.00	829.33	0.86	26.79	22.92	1.45	0.6
1.00	0.2	1.00	55.92	1.14	0.98	0.64	0.23	0.1

2.00	1.43	2.00	216.19	1.56	4.67	3.1	0.75	0.3
2.00	0.22	1.00	328.89	0.39	3.9	2.65	0.79	0.1
2.00	3.89	3.00	305.56	0.79	4.62	3.61	0.45	0.7
3.00	3.33	3.00	656	1.38	6.19	4.51	1.17	0.5
1.00	0.2	1.00	85.54	1.96	4.46	3.3	0.76	0.2
2.00	0.68	2.00	147.46	1.37	3.95	2.6	1.07	0.4
2.00	0.29	1.00	66.47	1.68	4.43	2.41	1.28	0.3
3.00	0.77	2.00	815.38	1.29	9.53	6.61	1.96	0.1
3.00	2.73	3.00	463.64	0.69	19.93	15.14	1.72	0.3
2.00	0.34	1.00	38.65	1.87	2.86	1.3	1.18	0.3
1.00	0.61	2.00	197.96	1.14	13.32	12.39	0.37	0.3
3.00	1.76	3.00	255.29	1.06	13.64	11.49	1.01	0.3
1.00	0.61	2.00	61.22	1.14	2.37	2.13	0.12	0.3
3.00	1.43	2.00	292.86	0.46	2.35	1.31	0.81	0.2
2.00	0.43	2.00	144.9	1.14	9.28	7.63	0.86	0.21
3.00	2.14	3.00	1435.71	4.28	10.73	6	3.43	0.3
2.00	1.33	2.00	230	0.54	4.66	3.04	0.91	0.4
2.00	0.21	1.00	98.67	1.38	3.83	2.62	0.74	0.16
2.00	0.41	1.00	184.49	1.14	17.91	16.99	0.51	0.2
2.00	0.71	2.00	54.76	1.46	3.77	2.13	1.05	0.6
2.00	0.61	2.00	393.88	1.14	14.07	10.83	1.43	0.3
2.00	1.11	2.00	111.11	0.53	4.28	3.36	0.66	0.3
2.00	0.29	1.00	23.05	3.45	5.36	2.08	2.37	0.3
2.00	1.06	2.00	204.26	0.72	18.16	14.67	1.86	0.5
2.00	0.78	2.00	147.06	0.13	6.34	4.24	1.39	0.4
2.00	1.52	2.00	384.85	3.66	16.47	14.57	1.18	0.5
2.00	1.03	2.00	206.15	0.64	4.03	2.69	0.89	0.4
3.00	2.86	3.00	371.43	0.55	7.5	5.45	1.6	0.4
3.00	0.61	2.00	125.71	0.83	8.67	5.44	2.26	0.3
3.00	1.33	2.00	363.33	1	4.94	2.35	1.89	0.4
3.00	0.41	2.00	373.97	1.74	4.66	1.12	3.14	0.3
2.00	0.26	1.00	123.38	1.7	9.55	7.21	1.38	0.2
2.00	0.65	2.00	78.71	0.95	9.26	7.73	1.19	0.2
1.00	0.45	2.00	26.36	0.91	2.02	0.65	0.85	0.5
3.00	7.78	3.00	855.56	0.9	4.36	3.17	0.57	0.7
1.00	0.66	2.00	268.13	1.25	4.14	3.46	0.44	0.6
3.00	7.14	3.00	705.71	0.56	4.67	3.48	0.86	0.5
3.00	1.63	3.00	175.51	1.63	13.22	9.43	2.3	0.8

2.00	2.61	3.00	213.04	0.54	11.68	11.11	0.36	0.6
2.00	1.21	2.00	113.33	0.93	17.86	12.84	1.26	0.4
3.00	3.64	3.00	518.18	0.22	7.8	6.2	1	0.4
2.00	0.61	2.00	148.98	1.14	12.83	10.63	1.36	0.3
2.00	1.96	3.00	304.35	0.53	5.19	4.27	0.54	0.45
2.00	0.6	2.00	237.31	1.69	11.71	9.39	1.79	0.4
2.00	0.54	2.00	242.86	1.42	4.88	2.94	1.45	0.38
2.00	0.6	2.00	70	0.54	6.75	4.84	0.82	0.41
3.00	6.36	3.00	963.64	0.67	11.54	9.3	1.75	0.7
3.00	3.57	3.00	435.71	0.73	9.1	5.5	2.1	0.5
2.00	0.82	2.00	122.45	1.14	10.9	9.18	0.87	0.4
1.00	1.25	2.00	26.25	0.32	1.45	1.05	0.25	0.4
1.00	0.22	1.00	62.04	1.14	0.91	0.66	0.22	0.11
2.00	1.22	2.00	218.37	1.14	6.16	3.68	1.39	0.6
2.00	2.5	3.00	285	0.97	6.29	4.61	0.55	0.5
2.00	0.86	2.00	86.29	0.84	13.01	12.4	0.5	0.3
1.00	0.13	1.00	74.67	0.44	2.04	1.22	0.63	0.1
2.00	3.08	3.00	216.92	0.64	4.1	3.52	0.38	0.4
1.00	0.88	2.00	258.82	2.67	12.02	10.04	0.92	0.9
2.00	2.11	3.00	107.37	0.56	3.26	2.7	0.33	0.4
3.00	2.31	3.00	1061.54	0.48	13.82	11.84	0.65	0.3
3.00	0.77	2.00	626.92	0.75	13.3	9.59	1.9	0.2
2.00	0.86	2.00	145.71	1.05	2.42	1.4	0.8	0.3
3.00	2.86	3.00	685.24	0.43	8.64	3.71	3.91	0.6
1.00	0.09	1.00	178.57	2.08	5.26	2.88	1.05	0.1
2.00	0.67	2.00	151.58	1.72	6.56	3.85	1.88	0.38
3.00	2.63	3.00	533.68	1.12	8.87	5.95	1.64	0.5
3.00	2.67	3.00	740	1.57	11.6	9.64	1.16	0.4
3.00	3.69	3.00	641.54	0.89	3.1	1.65	0.97	0.48
2.00	0.61	2.00	147.76	1.14	5.01	3.58	0.92	0.3
2.00	1.83	3.00	156.67	1.42	5.38	3.99	1	1.1
2.00	1.5	2.00	275	1.23	3.18	2.38	0.53	0.3
3.00	6.67	3.00	406.67	0.54	3.11	1.41	0.67	0.8
2.00	1.46	2.00	219.23	1.45	3.94	2.34	0.81	0.38
3.00	8.46	3.00	930.77	1.22	5.75	3.37	1.57	1.1
2.00	1.52	2.00	123.91	1.07	8.71	7.51	0.49	0.7
3.00	3.85	3.00	128.46	0.41	6.54	4.87	0.88	0.5
3.00	3.64	3.00	793.64	0.84	10.4	7.34	1.93	0.4

2.00	0.84	2.00	69.16	1.31	6.11	4.45	0.9	0.7
1.00	0.53	2.00	34.88	1.29	3.32	1.85	1	0.69
2.00	1.82	3.00	512.73	1.25	2.75	1.82	0.41	0.2
3.00	1.43	2.00	807.14	1.51	5.92	4.33	0.92	0.2
3.00	5	3.00	265	1.17	3.81	2.64	0.84	0.8
1.00	0.86	2.00	15.31	0.37	5.9	5.2	0.6	0.7
2.00	0.51	2.00	98.37	1.14	2.02	1.2	0.54	0.25
3.00	1.63	3.00	200	1.14	17.62	14.93	2.42	0.8
3.00	4.55	3.00	1881.82	0.44	11.33	9.41	0.74	0.5
3.00	3.33	3.00	413.33	0.97	6.95	5.56	0.81	0.5
1.00	0.32	1.00	57.26	1.04	4.28	3.55	0.41	0.3
3.00	3.33	3.00	304.44	1.13	9.97	8.28	0.73	0.3
1.00	0.12	1.00	172.62	1.66	6.77	5.66	0.66	0.1
2.00	0.81	2.00	164.86	0.69	4.79	2.94	1.34	0.3
3.00	2.78	3.00	338.89	0.84	5.8	3.6	1.2	0.5
2.00	1.48	2.00	403.7	3.92	8.17	6.58	0.75	0.4
3.00	10	3.00	972.86	0.39	17.33	12.94	2.65	0.7
1.00	0.32	1.00	101.9	1.77	4.6	3.93	0.41	0.2
3.00	2.31	3.00	402.31	0.53	3.87	2.8	0.58	0.3
1.00	0.16	1.00	54.26	1.6	2.32	1.36	0.67	0.2
2.00	1.22	2.00	85.71	1.14	4.75	2.69	1.41	0.6
1.00	0.59	2.00	115.29	0.43	3.72	3	0.2	0.2
1.00	0.27	1.00	43.2	1.12	2.16	1.61	0.32	0.2
3.00	2.5	3.00	658.33	0.58	15.56	12.47	0.82	0.3
2.00	1.43	2.00	289.71	0.23	8.43	6.07	1.12	0.5
1.00	0.38	1.00	99	0.9	7.55	6.52	0.32	0.3
3.00	3.08	3.00	700	0.59	5.38	3.72	0.86	0.4
3.00	4	3.00	405.33	1.97	8.39	6.17	1.33	0.6
3.00	2.5	3.00	436.67	0.2	5.08	3.52	0.9	0.3
3.00	0.81	2.00	246.88	1.38	11.27	7.75	2.54	0.26
2.00	0.55	2.00	166.18	1.82	2.62	1.2	1.1	0.3
3.00	3	3.00	570	1.05	6.92	5.07	1.01	0.3
2.00	0.38	1.00	112.82	1.71	7.7	6.5	0.8	0.3
3.00	2	3.00	730	0.59	4.35	2.52	1.07	0.2
2.00	2	3.00	986.67	1.73	9.61	8.32	0.59	0.3
1.00	0.33	1.00	44.78	0.87	2.82	1.7	0.7	0.3
2.00	1.94	3.00	494.44	1.84	23	21.9	0.9	0.7
2.00	0.41	1.00	124.49	1.14	4.1	2.5	1.2	0.2

3.00	3.64	3.00	767.27	0.58	11.77	10.34	0.78	0.4
1.00	0.34	1.00	34.79	1.4	1.36	0.96	0.29	0.4
2.00	0.61	2.00	585.71	1.14	3.96	2.36	1.11	0.3
2.00	1.18	2.00	505.88	0.59	6.15	4.19	1	0.4
2.00	0.66	2.00	74.92	0.35	5.55	3.96	0.61	0.4
2.00	1.61	3.00	241.94	1.37	3.12	2	0.9	0.5
2.00	2.59	3.00	281.48	0.65	4.8	3.33	0.85	0.7
2.00	3.33	3.00	461.67	1.09	4.21	3.46	0.26	0.4
3.00	6.67	3.00	440	0.55	5.13	3.24	1.28	0.8
1.00	0.59	2.00	45.1	1.48	1.6	1.1	0.2	0.3
3.00	1.3	2.00	1165.22	1.08	20.15	17.23	1.52	0.3
3.00	3.08	3.00	715.38	0.58	6.57	3.58	2.4	0.4
3.00	3.57	3.00	1300	0.99	6.87	4.66	1.36	0.5
1.00	0.41	1.00	80.34	1.54	10.04	9.1	0.47	0.24
3.00	0.63	2.00	668.75	0.93	6.76	4.36	1.56	0.1
3.00	2.11	3.00	396.84	1.77	12.04	9.51	1.51	0.4
2.00	0.32	1.00	145.16	0.82	8.16	4.05	3.7	0.3
1.00	0.48	2.00	81.9	1.15	2.54	1.4	0.75	0.5
2.00	1.15	2.00	203.85	0.59	4.5	3.4	0.9	0.3
2.00	0.88	2.00	338.24	1.39	6.9	5	1	0.3
3.00	1.54	2.00	325.38	0.35	11.14	9.28	0.67	0.2
3.00	2.31	3.00	224.62	0.62	4.67	2.9	1.1	0.3
3.00	2.67	3.00	716	0.76	12.55	9.03	1.86	0.4
3.00	8.57	3.00	957.14	0.15	5.91	4.23	1.49	0.6
1.00	0.15	1.00	34.81	2.59	0.78	0.51	0.17	0.2
3.00	1.82	3.00	557.27	0.97	4.47	3.16	0.91	0.2
2.00	0.87	2.00	87.39	0.9	3.82	2.7	0.8	0.4
2.00	0.16	1.00	97.8	0.65	12.21	8.8	1.9	0.3
3.00	7.5	3.00	442.5	0.31	4.14	2.48	0.98	0.6
2.00	0.31	1.00	216.56	1.28	7.76	5.96	1.22	0.2
2.00	2	3.00	65.6	0.35	6.93	5.99	0.82	0.5
1.00	0.62	2.00	83.69	1.16	1.38	0.58	0.49	0.4
3.00	0.56	2.00	150	0.67	12.21	9.9	1.76	0.2
2.00	0.82	2.00	130.61	1.14	14.81	12.94	1.23	0.4
2.00	0.84	2.00	94.29	1.38	2.22	1.25	0.88	0.53
2.00	0.91	2.00	80	1.03	10.8	8.41	0.93	0.3
2.00	1.15	2.00	69.33	2.27	4.1	2.62	1.06	0.69
1.00	0.3	1.00	18.3	2.03	2.4	2.14	0.18	0.4

1.00	0.88	2.00	63.75	0.79	2.12	1.2	0.7	0.7
2.00	0.41	1.00	24.08	1.14	2.67	1.79	0.52	0.2
1.00	0.12	1.00	17.08	1.15	5.8	4.6	0.8	0.2
1.00	0.24	1.00	53.07	1.02	2.97	2.23	0.53	0.3
1.00	4.71	3.00	147.65	0.22	4.37	3.68	0.14	0.8
3.00	2.78	3.00	900	0.55	4.02	2.54	0.86	0.25
2.00	0.3	1.00	54	1.83	4.11	2.33	1.26	0.3
2.00	0.44	2.00	45.19	1.57	3.12	1.58	1.21	0.48
3.00	1.54	2.00	415.38	1.07	8.4	6.9	0.8	0.2
3.00	3.18	3.00	721.82	0.59	4.31	3.34	0.59	0.35
2.00	0.15	1.00	81.41	0.98	6.28	2.69	2.53	0.27
3.00	0.82	2.00	118.37	1.46	6.84	4.1	2.1	0.4
3.00	0.82	2.00	304.08	1.14	4.46	1.2	2.42	0.4
2.00	0.58	2.00	113.46	1.03	2.8	1.4	1.1	0.3
1.00	0.77	2.00	288.46	0.38	3.96	2.94	0.2	0.2
3.00	1.25	2.00	406.25	1.27	4.61	2.7	1.34	0.2
3.00	2.94	3.00	576.47	0.86	3.37	2.02	1.05	0.5
3.00	1.15	2.00	292.31	1.3	5.52	2.88	1.23	0.3
2.00	1.82	3.00	218.18	1.1	7.4	5.3	1.8	0.8
2.00	0.46	2.00	101.61	1.16	3.74	1.43	1.76	0.4
2.00	0.36	1.00	283.93	1.35	7.5	6.6	0.7	0.2
3.00	1.82	3.00	227.27	1.62	4.91	1.58	2.41	0.4
2.00	0.61	2.00	114.29	1.14	9.83	7.46	1	0.3
1.00	0.18	1.00	53.08	1.54	3.79	2.97	0.4	0.14
1.00	0.33	1.00	38.98	1.79	3.6	4.65	1.48	0.52
2.00	2.44	3.00	344.44	0.9	8.7	7	1.1	0.66
2.00	0.67	2.00	113.33	0.77	7.31	5.71	1.03	0.3
2.00	0.54	2.00	93.1	1.71	8.79	4.36	2.5	0.7
2.00	0.43	2.00	133.7	2.35	8.46	7.06	1.06	0.4
2.00	0.48	2.00	100	1.87	2.91	1.31	0.97	0.3
2.00	0.96	2.00	164.91	1.02	13.24	12.3	0.56	0.55
3.00	3.45	3.00	320	0.78	6.86	4.527	1.12	0.38
2.00	0.61	2.00	86.53	1.14	4.13	2.69	0.76	0.3
1.00	0.72	2.00	81.16	1.5	1.88	1.08	0.56	0.5
2.00	0.35	1.00	69.57	1.34	4.3	2.7	1.2	0.4
2.00	1	2.00	141.33	0.32	3.14	1.72	1.1	0.3
2.00	0.81	2.00	124.86	1.21	3.53	2.7	0.61	0.3
1.00	1.04	2.00	27.08	1.29	2	1.3	0.3	0.5

2.00	0.88	2.00	72.65	1.37	7.55	6.19	0.82	0.6
2.00	0.44	2.00	141.18	1.37	5.63	4.09	0.86	0.3
2.00	1.02	2.00	195.92	1.14	5.63	4.09	0.86	0.5
2.00	0.25	1.00	111.44	1.62	6.97	1.72	1.18	0.3
2.00	1.21	2.00	330.3	2.25	3.43	2.32	0.73	0.4
2.00	0.23	1.00	59.3	1.7	10.13	8.26	1.16	0.2
1.00	0.29	1.00	106.02	2.42	3.06	2.13	0.63	0.24
1.00	0.69	2.00	31.78	0.4	2.87	1.68	0.92	0.7
2.00	0.67	2.00	48.57	3.09	6.79	4.84	1.27	0.7
3.00	3.33	3.00	483.33	1.83	5.45	3.48	1.04	0.6
1.00	0.77	2.00	105.04	1.36	4.91	3.2	1.08	0.9
2.00	0.47	2.00	68.68	1.7	3.91	2.89	0.59	0.25
3.00	3.81	3.00	1004.76	1.2	8.91	6.75	0.9	0.8
2.00	1.08	2.00	140.82	1.14	2.19	1.4	0.51	0.53
3.00	2.22	3.00	955.56	0.81	13.61	9.9	2.7	0.2
2.00	1.14	2.00	34.29	0.88	7.3	5.4	0.8	0.8
2.00	0.38	1.00	44.62	0.68	6.3	4.6	1.1	0.2
2.00	0.34	1.00	118.99	1.78	7.79	3.23	3.52	0.6
3.00	3.07	3.00	873.33	1.15	13.66	10.52	1.87	0.46
2.00	0.75	2.00	49.25	0.81	3.61	2.48	0.86	0.5
3.00	4	3.00	700	1.57	4.14	2.83	0.78	0.4
2.00	0.62	2.00	105.88	3.41	4.01	2.48	1.05	0.42
3.00	2	3.00	525.33	0.91	6.58	4.88	0.93	0.3
2.00	0.16	1.00	174.6	1.26	8.4	7.05	0.81	0.1
2.00	0.82	2.00	202.04	1.14	7.09	5.35	1.17	0.4
2.00	1.25	2.00	185	2.35	5.66	3.48	1.29	0.5
3.00	2.5	3.00	395	0.54	2.81	1.69	0.63	0.3
1.00	0.11	1.00	56.14	1.52	4.49	3.41	0.61	0.1
3.00	1.58	2.00	289.47	1.09	9.78	7.62	1.27	0.3
2.00	0.57	2.00	271.7	1.02	8.89	6.71	1.56	0.3
2.00	3.33	3.00	225.83	2.2	5.18	4.14	0.58	0.8
2.00	0.83	2.00	253.89	1.01	22.96	19.08	1.45	0.3
3.00	7.5	3.00	1625	0.65	18.55	16.68	1.15	0.9
1.00	0.14	1.00	66.67	0.63	3.15	1.98	0.6	0.2
1.00	0.11	1.00	39.33	1.88	5.31	4.19	0.68	0.1
2.00	0.91	2.00	297.27	1.5	3.09	2.08	0.64	0.2
1.00	0.92	2.00	78.46	1.26	3.08	2.21	0.31	0.6
2.00	1.86	3.00	127.91	0.8	9.9	6.6	1.7	0.8

2.00	1.22	2.00	133.33	1.04	6.78	5.29	0.71	0.62
2.00	0.41	1.00	122.45	1.14	3.46	1.61	1.1	0.2
2.00	0.31	1.00	117.19	2.56	8.41	6.21	1.29	0.2
3.00	1.59	3.00	827.65	0.8	14.41	11.62	0.93	0.27
1.00	0.5	2.00	124.17	2.39	4.1	3.12	0.46	0.6
2.00	0.45	2.00	161.36	0.74	10.76	8.99	0.77	0.2
1.00	1.3	2.00	56.85	1.11	7.08	6.64	0.22	0.7
2.00	0.83	2.00	127.78	1.15	4.11	2.6	0.98	0.3
2.00	1.11	2.00	229.63	0.49	5.6	4.8	0.4	0.3
2.00	0.47	2.00	57.44	2	4.75	3.07	1.18	0.4
2.00	6.67	3.00	226.67	0.48	5.93	5.27	0.38	0.6
2.00	0.74	2.00	87.41	0.53	10	8.8	0.8	0.4
3.00	2.5	3.00	675	1.6	3.98	2.22	1.24	0.3
2.00	1	2.00	62.95	1.44	4.54	3.02	1.04	0.61
1.00	0.98	2.00	47.84	1.91	3.06	2.24	0.32	0.5
2.00	0.62	2.00	150.99	1.92	6.85	2.66	3.43	0.5
1.00	0.61	2.00	192.45	1.14	5.97	4.4	0.39	0.3
2.00	0.53	2.00	293.33	1.88	3.66	1.92	1.03	0.16
2.00	0.2	1.00	46.53	1.14	7.47	5.36	0.96	0.1
1.00	0.25	1.00	29.25	0.79	2.9	1.8	0.8	0.26
1.00	0.18	1.00	39.45	1.13	3.15	2.19	0.62	0.2
2.00	0.09	1.00	75.89	0.23	4.11	2.29	1.39	0.1
2.00	0.75	2.00	133.96	1.47	4.8	2	1.1	0.4
1.00	0.38	1.00	67.92	1.71	3.37	1.85	0.92	0.4
1.00	0.85	2.00	46.17	1.38	2.44	1.65	0.56	0.8
1.00	0.39	1.00	23.61	1.35	2.94	1.77	0.79	0.6
2.00	0.39	1.00	34.69	2.94	4.16	1.85	1.77	0.5
2.00	0.41	1.00	121.22	1.14	4.83	3.26	1.16	0.2
3.00	4.17	3.00	1141.67	1.29	6.36	4.39	1.16	0.5
2.00	0.83	2.00	57.08	0.89	5.57	4.91	0.58	0.4
2.00	1.07	2.00	178.81	1.45	4.63	3.21	0.95	0.45
1.00	0.78	2.00	92.19	0.95	7.49	6.93	0.42	0.5
3.00	0.94	2.00	54.38	1.76	2.97	1.11	1.53	0.3
1.00	0.22	1.00	54.03	1.21	3.09	2.37	0.48	0.3
1.00	0.56	2.00	79.63	1.64	6.83	5.49	0.87	0.6
1.00	1	2.00	108.33	0.52	1.86	1.45	0.27	0.3
1.00	0.3	1.00	47.47	1.08	5.7	4.5	0.8	0.3
2.00	0.68	2.00	65.05	1.82	5.82	4.4	1.06	0.7

2.00	1	2.00	81.67	1.11	3.6	1.9	1.4	0.6
3.00	1.67	3.00	1216.67	1.27	13.79	11.81	1.08	0.4
1.00	0.18	1.00	33.19	1.52	2.9	1.67	0.79	0.2
2.00	0.97	2.00	416.13	0.79	14.37	12.78	0.65	0.3
2.00	0.94	2.00	115.09	1.7	6.06	3.89	1.89	0.5
2.00	0.29	1.00	76.47	0.57	3.31	2.11	0.72	0.2
1.00	0.49	2.00	156.39	1.72	3.01	2.36	0.5	0.3
3.00	5.3	3.00	1120	0.56	8.42	5.08	2.52	0.53
1.00	0.35	1.00	14.17	1.83	3.43	2.62	0.56	0.5
1.00	0.33	1.00	126.98	2.76	3.39	1.92	0.88	0.41
1.00	0.6	2.00	9.76	2.17	3.52	2.5	0.6	0.99
2.00	0.61	2.00	269.39	1.14	2	1.1	0.7	0.3
1.00	0.52	2.00	56.3	1.85	3.9	2.9	0.8	0.7
1.00	0.13	1.00	93.63	1.35	9.6	8.6	0.3	0.2
1.00	0.49	2.00	47.48	1.04	3.13	2.05	0.41	0.6
1.00	0.26	1.00	77.92	1.19	4.97	4.28	0.5	0.2
2.00	0.73	2.00	219.51	5.36	3.61	2.07	0.75	0.3
1.00	0.26	1.00	47.92	1.32	1.63	0.99	0.39	0.2
2.00	0.41	1.00	96.73	1.14	1.88	0.99	0.61	0.2
2.00	0.49	2.00	213.9	0.89	9.1	7.14	1.32	0.2
1.00	0.51	2.00	87.34	1.41	2.99	1.84	0.7	0.4
2.00	1.43	2.00	207.14	1.36	7.34	6.1	0.78	0.4
2.00	0.49	2.00	263.41	3.23	6.97	5.49	1.01	0.4
2.00	0.67	2.00	140.33	0.56	8.79	6.08	1.75	0.4
2.00	0.22	1.00	44.17	1.88	4.3	2	1.9	0.36
2.00	0.98	2.00	143.9	2.45	4.74	2.86	1.24	0.4
1.00	0.32	1.00	49.36	0.89	3.94	2.86	0.59	0.3
1.00	0.43	2.00	30.26	1.64	1.96	1.18	0.46	0.5
2.00	1.2	2.00	251.76	1.38	14.41	13	0.76	0.61
2.00	1.67	3.00	127.78	2.69	5.3	3.4	1.3	0.6
2.00	0.62	2.00	163.08	1.67	3.07	1.28	1.29	0.4
1.00	0.34	1.00	61.67	2.09	2.46	1.51	0.64	0.45
2.00	5	3.00	1558.33	2.03	6.25	5.43	0.48	0.6
1.00	0.82	2.00	68.16	1.14	2.67	2.04	0.41	0.4
3.00	2.86	3.00	1050	1.55	7.25	4.69	1.52	0.4
2.00	0.32	1.00	154.74	1.08	4.87	2.46	1.51	0.3
1.00	0.63	2.00	43	0.8	1.48	1.08	0.39	0.5
2.00	1.05	2.00	101.75	0.48	16.27	13.51	1.74	0.6

1.00	0.82	2.00	146.94	1.14	1.9	1.46	0.25	0.4
2.00	2.5	3.00	285.71	0.96	3.22	2.29	0.39	0.7
3.00	6.25	3.00	942.5	0.39	3.07	2.01	0.58	0.5
2.00	0.56	2.00	89.63	1.57	3.01	1.99	0.57	0.3
3.00	0.59	2.00	217.65	1.24	18.33	13.06	3.97	0.3
2.00	1.38	2.00	96.55	1.12	2.88	1.44	1.22	0.4
2.00	0.7	2.00	145.63	1.16	7.49	5.54	1.12	0.5
2.00	1.54	2.00	190.38	1.38	9.38	7.7	1.31	0.8
1.00	0.35	1.00	76.14	1.67	7.91	6.7	0.8	0.31
2.00	1.34	2.00	90.29	1.3	3.17	1.88	0.83	0.91
2.00	0.85	2.00	83.05	1.53	3.09	1.83	0.71	0.5
3.00	1.54	2.00	264.62	0.54	8.87	6.33	1.12	0.2
1.00	0.51	2.00	44.29	0.94	1.8	0.77	0.22	0.5
2.00	0.63	2.00	145	2.12	7.41	6.06	0.82	0.2
2.00	1.82	3.00	233.33	1.5	4.13	2.49	1.21	0.6
1.00	0.32	1.00	42.83	0.51	7.33	6.23	0.53	0.41
2.00	0.26	1.00	61.05	1.29	2.65	1.4	0.81	0.2
2.00	0.2	1.00	89	1.66	7.7	5.3	1.4	0.2
2.00	0.11	1.00	166.55	2.01	6.44	2.96	2.29	0.1
2.00	0.61	2.00	102.86	1.14	5.82	4.42	1.02	0.3
2.00	1.19	2.00	164.29	0.57	9.42	7	1.32	0.5
3.00	2.67	3.00	1293.33	1.53	10.96	8.28	1.43	0.4
2.00	1.18	2.00	223.53	0.48	7.96	6.57	0.95	0.4
2.00	0.82	2.00	103.88	1.14	7.21	6.07	0.51	0.4
2.00	0.8	2.00	110.8	0.92	3.51	1.6	0.86	0.2
3.00	3.83	3.00	270	1.09	4.84	3.63	0.6	0.46
2.00	0.79	2.00	80.79	2.2	6.98	4.41	1.81	0.8
2.00	0.3	1.00	91.19	1.64	4.93	3.29	1.13	0.3
2.00	2.07	3.00	179.31	1.14	3.14	2.04	0.84	0.6
1.00	0.48	2.00	59.52	1.55	2.11	1.71	0.32	0.4
1.00	0.19	1.00	29.23	1.9	2.7	1.35	0.8	0.2
1.00	0.2	1.00	46.26	1.18	2.49	1.32	0.81	0.3
1.00	0.91	2.00	143.18	0.93	8.79	8.38	0.34	0.4
2.00	0.6	2.00	273.33	2.06	8.4	6.6	1.4	0.27
2.00	0.34	1.00	106.74	2.92	5.14	3.7	1.02	0.3
2.00	0.43	2.00	48.82	0.89	3.13	1.65	1.22	0.4
1.00	0.91	2.00	59.7	1.3	2.34	1.88	0.33	0.6
2.00	0.36	1.00	126.79	0.8	4.35	2.72	1.07	0.2

2.00	0.56	2.00	109.86	0.88	5.43	3.49	1.18	0.4
2.00	0.07	1.00	38.06	2	4.2	2.1	1.7	0.1
1.00	0.24	1.00	57.72	2.79	2.87	2.09	0.48	0.3
2.00	0.41	1.00	97.35	2.82	4.02	2.29	1.16	0.4
2.00	0.37	1.00	408.16	1.14	5.83	4.05	1.05	0.18
2.00	0.8	2.00	67.05	0.97	19.91	18.1	1.2	0.7
1.00	0.59	2.00	15	0.81	1.56	0.94	0.33	0.6
2.00	0.57	2.00	144.9	1.14	4.48	3.19	1.06	0.28
2.00	0.95	2.00	109.37	2.13	4.1	2.11	1.51	0.6
2.00	0.45	2.00	195.45	3.07	10.07	8.46	1.4	0.3
1.00	0.3	1.00	35.26	1.76	3.36	2.16	0.5	0.4
1.00	0.31	1.00	48.16	1.69	3.72	2.8	0.6	0.3
2.00	0.37	1.00	185.98	1.92	14.27	12.08	1.56	0.4
1.00	0.21	1.00	57.04	2.05	2.41	1.43	0.62	0.3
1.00	0.47	2.00	79.07	1.18	4.32	2.81	0.73	0.4
1.00	0.48	2.00	157.58	1.01	3.78	2.85	0.53	0.3
3.00	6.15	3.00	203.08	0.98	5.03	1.32	0.58	0.8
3.00	3.24	3.00	208.11	1.53	7.39	4.69	1.85	1.2
2.00	0.29	1.00	85.58	1.04	4.53	2.33	1.16	0.3
2.00	0.76	2.00	481.01	2.16	5.26	3.05	1.76	0.6
2.00	1	2.00	203.33	2.37	6.06	2.23	2.26	0.6
1.00	0.63	2.00	40.32	1.29	7.17	6.71	0.2	0.4
1.00	0.19	1.00	16.31	1.73	2.45	1.3	0.89	0.3
2.00	0.25	1.00	27.85	0.97	3.6	1.9	1.4	0.2
1.00	0.61	2.00	79.8	1.52	13.23	11.87	0.64	0.6
2.00	1.08	2.00	132.16	1.47	1.77	0.75	0.76	0.4
3.00	8.46	3.00	792.31	1.58	5.64	3.65	1.23	1.1
2.00	1.43	2.00	70.2	1.14	4.37	2.96	1.06	0.7
1.00	0.34	1.00	68.97	1.02	3.32	2	1.1	0.4
3.00	1.25	2.00	690	1.38	9.45	5.8	2.28	0.2
2.00	0.61	2.00	516.33	1.14	5.37	2.03	2.09	0.3
2.00	0.49	2.00	81.97	1.01	3.94	2.39	0.82	0.3
1.00	0.78	2.00	58.64	1.45	2.52	1.48	0.67	0.8
2.00	5.92	3.00	526.53	1.14	6.36	4.66	1.05	2.9
2.00	0.42	2.00	25.63	0.88	3.22	2.3	0.8	0.3
2.00	0.41	1.00	154.05	1.39	10.63	7.4	2.28	0.3
2.00	0.19	1.00	75.87	1.97	4.19	1.22	2.24	0.2
1.00	0.23	1.00	155.3	1.4	3.32	2.2	0.73	0.31

1.00	0.31	1.00	119.38	1.36	1.68	1.27	0.26	0.2
3.00	2.5	3.00	1216.67	0.74	14.76	12.96	1.19	0.3
1.00	0.55	2.00	76.55	2.21	7.69	6.09	0.98	0.8
2.00	0.35	1.00	51.06	1.73	5.88	2.87	1.98	0.33
2.00	0.63	2.00	130.31	0.68	4.95	3.33	1.09	0.2
2.00	0.57	2.00	180	1.8	4.83	2.64	1.6	0.4
2.00	1.76	3.00	455.88	1.39	5.6	4.14	0.83	0.6
1.00	0.21	1.00	23.08	1.57	2.64	1.39	0.92	0.3
2.00	1.67	3.00	101.33	1.53	10.97	9.55	0.65	0.5
2.00	0.66	2.00	81.58	2.04	3.69	2.41	0.89	0.5
2.00	0.35	1.00	136.47	2.73	5.5	3.44	1.5	0.3
2.00	0.78	2.00	198.7	2.09	9.75	6.66	2.42	0.6
2.00	0.82	2.00	251.02	1.14	3.55	2.45	0.57	0.4
2.00	1.29	2.00	51.43	3.08	5.09	2.15	1.63	0.9
1.00	0.64	2.00	62.82	1.01	3.06	2.25	0.49	0.5
2.00	0.48	2.00	261.9	1.08	7.18	5.25	1.3	0.2
2.00	1.67	3.00	130	1.17	2.19	1.18	0.79	0.5
2.00	0.2	1.00	120.41	1.14	5.06	3.21	0.81	0.1
2.00	0.41	1.00	126.53	1.05	7.21	5.29	1.08	0.2
2.00	0.8	2.00	84.8	0.92	9.97	8.03	1.02	0.4
1.00	0.26	1.00	33.97	1.28	2.84	1.6	0.73	0.3
2.00	0.82	2.00	214.29	1.14	4.05	3.11	0.5	0.4
1.00	0.27	1.00	33.57	1.32	1.64	1.08	0.34	0.35
1.00	0.26	1.00	139.74	1.36	2.62	1.8	0.6	0.2
2.00	0.36	1.00	119.82	1.87	5.34	3.23	1.57	0.4
2.00	0.36	1.00	222.89	0.91	6.84	5.86	0.94	0.3
2.00	0.16	1.00	78.06	0.39	4.32	3.18	0.76	0.1
2.00	0.29	1.00	126.67	2.25	13.4	9.5	2.7	0.3
2.00	0.61	2.00	136.59	2.35	4.94	3.37	1.04	0.5
2.00	0.37	1.00	108.54	5.62	5.8	4.4	1	0.3
1.00	0.29	1.00	27.57	1.33	2.84	1.7	0.67	0.3
1.00	0.47	2.00	28.68	0.99	2	1.02	0.66	0.5
1.00	0.37	1.00	167.56	1.34	3.8	2.91	0.62	0.3
1.00	0.66	2.00	31.57	1.5	3.32	2.7	0.5	0.8
2.00	1.25	2.00	145	1.09	9.93	8.49	0.97	0.5
2.00	1.21	2.00	248.48	1.47	3.33	2.25	0.76	0.4
2.00	0.46	2.00	90.77	0.51	15.22	13.22	1	0.3
1.00	0.18	1.00	49.54	2.46	2.9	2.07	0.56	0.2

2.00	0.37	1.00	45.7	2.19	5.68	3.15	1.74	0.5
3.00	1.03	2.00	408.21	2.07	15.75	12.95	2.31	0.4
2.00	0.86	2.00	115.43	0.43	7.33	6.35	0.5	0.3
2.00	0.23	1.00	98.55	0.56	5.46	4.45	0.79	0.16
2.00	1.03	2.00	136.92	1.72	3.55	2.52	0.56	0.4
2.00	0.85	2.00	125.53	1.65	7.66	6.21	0.81	0.4
3.00	1.63	3.00	124.49	1.14	11.92	7.79	2.17	0.8
3.00	1.33	2.00	177.78	1.22	8.48	4.74	2.8	0.6
2.00	0.48	2.00	132.26	2	4.5	3.3	0.6	0.3
1.00	0.35	1.00	46.71	1.33	3.74	2.81	0.62	0.3
1.00	0.36	1.00	45.45	0.83	4.5	3.3	0.6	0.4
2.00	1.82	3.00	196.97	0.93	5.4	4.64	0.67	0.6
1.00	1.03	2.00	107.69	0.97	9.69	8.94	0.28	0.4
2.00	0.73	2.00	69.27	1.15	5.57	4.39	0.59	0.3
1.00	0.18	1.00	47.71	1.36	3.99	2.9	0.69	0.2
2.00	0.44	2.00	158.82	1.07	8.16	7.09	0.84	0.3
3.00	2.22	3.00	333.33	0.92	7	5.53	0.83	0.4
2.00	0.15	1.00	125.76	1.82	4.01	2.16	1.23	0.1
1.00	0.74	2.00	75	1.49	18.57	17.34	0.37	0.5
2.00	0.76	2.00	119.7	1.08	9.7	7.4	1.8	0.5
2.00	0.35	1.00	49.3	1.46	7.21	5.64	0.84	0.3
1.00	0.55	2.00	50.99	0.69	2.4	1.64	0.53	0.5
2.00	1.56	2.00	296.88	0.87	6.2	4.39	1.33	0.5
2.00	0.71	2.00	147.62	1.24	10.2	8.6	0.9	0.3
1.00	0.45	2.00	28.18	1.94	5.42	3.88	1.02	0.7
2.00	0.41	1.00	424.49	1.14	8.91	6.05	1.05	0.2
2.00	0.63	2.00	117.72	0.93	6.68	5.23	0.92	0.5
2.00	0.71	2.00	187.5	1.14	4.62	3.38	0.76	0.34
2.00	0.24	1.00	145.6	1.59	8.31	4.61	2.65	0.3
2.00	0.26	1.00	157.89	2.32	6.91	4.87	1.38	0.2
2.00	1	2.00	151.02	1.14	5.99	4.5	1.29	0.49
2.00	0.55	2.00	78.55	1.56	4.49	2.94	0.69	0.3
1.00	0.25	1.00	40.17	0.46	2.84	1.84	0.64	0.3
2.00	0.48	2.00	198.41	0.78	18.96	15.09	2.46	0.3
3.00	1.18	2.00	364.71	1.02	6.03	3.92	0.99	0.2
2.00	3.47	3.00	119.7	1.23	5.39	4.04	1.07	2.29
3.00	1.76	3.00	447.06	1.53	13.39	10.13	1.47	0.3
1.00	0.26	1.00	70.26	1.54	4.1	3.17	0.58	0.2

2.00	1.05	2.00	108.77	0.97	7.3	5.9	0.88	0.6
3.00	1.89	3.00	289.19	1.51	6.3	2.8	2.3	0.7
2.00	1.58	2.00	302.11	1.59	3.53	2.23	0.71	0.3
1.00	0.06	1.00	64.94	1.49	4.78	3.16	1.19	0.1
3.00	2.35	3.00	623.53	1.38	5.51	3.39	1.41	0.4
2.00	0.54	2.00	93.48	1.25	8.8	7	1.5	0.5
2.00	0.59	2.00	49.75	0.83	4.57	3.44	0.88	0.47
1.00	0.86	2.00	83.62	1.36	3.91	3.19	0.34	1
1.00	0.78	2.00	23.53	1	8	7.35	0.23	0.8
2.00	0.46	2.00	215.38	1.55	5.91	4.66	0.8	0.3
2.00	0.11	1.00	270.21	1.91	8.54	6.3	1.29	0.1
3.00	4.17	3.00	816.67	0.97	4.73	2.9	1.3	0.5
3.00	63	3.00	1135	0.84	5.62	3.13	1.62	6.3
1.00	0.34	1.00	99.31	1.88	4.7	3.4	0.7	0.5
2.00	0.33	1.00	197.54	2.63	7.03	4.99	1.56	0.4
2.00	0.26	1.00	100	1.31	5.91	4.05	0.97	0.2
3.00	2.5	3.00	1741.67	1.08	6.31	4.36	1.21	0.3
2.00	0.82	2.00	126.53	1.14	3.04	2.11	0.56	0.4
2.00	1.4	2.00	63.72	0.64	4.99	3.7	0.75	0.6
1.00	0.45	2.00	56.59	1.03	7.4	5.16	0.79	0.4
2.00	0.77	2.00	121.03	1.07	6.59	5.26	0.56	0.3
2.00	0.35	1.00	38.04	1.25	2.71	1.5	0.93	0.32
1.00	0.34	1.00	41.2	1.19	4.81	3.14	0.87	0.4
2.00	2.78	3.00	74.44	1.69	4.23	3.79	0.2	0.5
1.00	0.55	2.00	32.32	1.19	2.47	1.6	0.71	0.9
2.00	0.17	1.00	310	0.72	8.42	7.2	0.79	0.1
1.00	0.61	2.00	62.86	1.14	1.71	1.3	0.23	0.3
3.00	4.29	3.00	305.71	0.71	10.18	8.29	0.79	0.3
2.00	1.63	3.00	128.57	1.14	4.06	2.98	0.73	0.8
1.00	0.35	1.00	10.04	0.78	2	1.3	0.6	0.8
2.00	1.39	2.00	519.44	2.5	13.36	11.76	1.04	0.5
2.00	0.67	2.00	129.22	1.27	7.44	5.04	1.69	0.6
1.00	0.83	2.00	117.78	0.73	2.39	1.9	0.33	0.3
1.00	0.32	1.00	73.23	1.73	7.02	6.41	0.47	0.2
2.00	3.57	3.00	324.29	0.48	13.62	12.51	0.51	0.5
1.00	0.38	1.00	63.46	1.16	3.78	3.09	0.44	0.4
2.00	0.18	1.00	145.03	2.28	5.26	2.45	2.01	0.3
1.00	0.81	2.00	55.27	1.71	2.98	2.37	0.36	0.6

2.00	0.5	2.00	192.5	1.84	8.84	7.5	0.99	0.2
3.00	1.82	3.00	536.36	1.34	5.39	3.92	0.84	0.2
2.00	0.5	2.00	66	1.32	3.19	2.53	0.41	0.2
2.00	2.78	3.00	297.78	1.05	12.66	10.65	0.67	0.5
2.00	0.41	1.00	200	1.14	7.57	5.48	1.53	0.2
2.00	1.2	2.00	170	4.05	5.05	2.53	1.43	0.6
2.00	0.97	2.00	98.61	0.98	13.01	10.62	1.58	0.7
2.00	0.82	2.00	108.16	1.14	5.48	4.1	0.87	0.4
1.00	0.17	1.00	24.94	2.19	3.23	2.37	0.51	0.3
2.00	0.48	2.00	35	0.98	3.75	2.15	1.03	0.5
1.00	0.18	1.00	71.01	0.74	2.08	1.31	0.49	0.2
2.00	0.6	2.00	342.39	0.87	11.84	10.6	0.92	0.4
3.00	3	3.00	978.57	0.68	10.57	9.37	0.6	0.42
2.00	0.38	1.00	61.74	1.79	4.06	2.19	1.35	0.44
1.00	0.8	2.00	44.09	1.55	1.42	0.8	0.38	0.7
2.00	0.37	1.00	622.22	1.52	14.31	12.04	1.09	0.1
2.00	0.47	2.00	290.63	1.67	14.25	12.29	1.41	0.3
2.00	4	3.00	435.71	0.4	5.97	4.62	0.45	0.56
2.00	0.45	2.00	120.45	1.8	4.49	2.14	1.63	0.4
1.00	0.41	1.00	144.59	1.27	6.2	5.4	0.6	0.3
1.00	0.11	1.00	129.47	0.95	6	4.7	0.8	0.1
2.00	0.21	1.00	162.55	0.71	8.32	5.63	1.96	0.1
2.00	0.98	2.00	41.57	1.81	2.42	1.2	0.83	0.5
1.00	0.38	1.00	73.46	0.76	3.22	2.68	0.27	0.4
2.00	0.34	1.00	108.62	1.08	3.54	2.61	0.57	0.2
1.00	0.43	2.00	63.44	1.8	2.88	2.1	0.27	0.4
1.00	1.34	2.00	48.2	2.39	2.57	1.92	0.37	0.82
1.00	0.23	1.00	33.41	0.91	1.65	1.09	0.46	0.2
2.00	0.89	2.00	179.29	0.89	13.79	10.9	1.88	0.5
2.00	0.61	2.00	378.37	1.14	7.2	6	0.7	0.3
2.00	0.5	2.00	240	1.37	5.81	4.18	1.11	0.4
2.00	0.26	1.00	20.51	1.48	5.6	3.77	1.2	0.2
2.00	0.82	2.00	157.14	1.14	4.1	2.85	0.88	0.4
1.00	0.56	2.00	22.7	0.98	4.83	4.33	0.3	0.5
1.00	0.61	2.00	35.51	1.14	1.66	1.1	0.39	0.3
1.00	0.59	2.00	26.14	1.69	4.33	3.07	0.77	0.6
2.00	0.13	1.00	163.64	2.33	7.86	5.74	0.86	0.1
1.00	0.22	1.00	68.25	2.48	10.23	7.89	1.04	0.3

1.00	0.09	1.00	45.14	1.2	6.7	5.2	1	0.1
1.00	0.88	2.00	66.25	1.95	2.9	2.2	0.5	0.7
2.00	0.2	1.00	53.4	1.36	12.73	11.2	0.97	0.2
1.00	0.73	2.00	30.41	6.15	4.26	2.66	0.99	0.9
1.00	0.18	1.00	48.3	1.39	7.83	5.33	1.21	0.3
3.00	1.02	2.00	120.41	1.14	18.93	15.01	2.17	0.5
1.00	0.22	1.00	37.8	0.68	1.68	1.24	0.26	0.2
2.00	3.08	3.00	730.77	0.83	5.41	4.52	0.39	0.4
3.00	1.08	2.00	618.92	1.48	17.9	12.3	4.19	0.4
3.00	4	3.00	1300	0.16	15.08	11.28	2.87	0.4
1.00	0.13	1.00	31.25	1.32	1.7	1.1	0.4	0.1
1.00	0.58	2.00	28.5	1.59	2.72	2.1	0.3	0.7
2.00	0.61	2.00	144.9	1.14	2.7	1.3	1.2	0.3
2.00	0.43	2.00	111.43	1.13	4.84	2.86	0.98	0.3
1.00	0.76	2.00	67.62	0.93	3.62	2.64	0.69	0.8
1.00	0.14	1.00	11.61	2.11	3.82	2.1	1.4	0.3
2.00	0.81	2.00	874.05	3.17	10.46	8.23	1.21	0.3
1.00	0.53	2.00	41.87	0.85	2.49	2.12	0.21	0.4
1.00	0.24	1.00	45.16	1.25	2.91	1.66	0.81	0.3
1.00	0.1	1.00	51.36	1.46	7.2	4.22	1.32	0.2
2.00	1.3	2.00	200	0.57	3	1.3	1	0.6
2.00	0.09	1.00	67.27	1.58	5.32	2.8	1.52	0.1
3.00	5.71	3.00	662.86	0.74	4.51	3.59	0.55	0.4
2.00	1.29	2.00	210.2	1.14	14.09	11.75	0.53	0.63
2.00	0.61	2.00	45.86	1.57	5.03	3.07	1.3	0.6
3.00	2.31	3.00	1133.85	1.11	7.69	4.55	2.38	0.3
2.00	0.53	2.00	125.09	1.47	7.54	5.26	1.21	0.3
3.00	2	3.00	730	0.47	7.81	5.84	1.48	0.4
2.00	0.92	2.00	138.46	2.43	4.5	2.9	1	0.6
1.00	0.63	2.00	83.17	1.55	9.78	8.18	0.6	0.4
1.00	0.59	2.00	135.29	1.63	5.96	4.93	0.55	0.4
1.00	0.82	2.00	96.73	1.14	2.37	1.82	0.32	0.4
2.00	0.73	2.00	149.09	2.14	5.17	3.24	1.15	0.4
1.00	0.37	1.00	63.3	0.99	2.8	1.9	0.5	0.4
1.00	0.41	1.00	49.8	1.31	1.34	0.88	0.29	0.2
1.00	0.71	2.00	70.57	0.92	5.3	4.47	0.54	0.5
3.00	1.08	2.00	145.95	1.8	5.28	3.09	1.82	0.4
2.00	0.24	1.00	144.71	1.68	4.44	1.9	1.92	0.2

2.00	0.82	2.00	181.63	1.14	5.57	3.62	1.48	0.4
1.00	0.41	1.00	48.37	1.15	3.73	2.86	0.36	0.4
1.00	0.33	1.00	74	0.9	5.03	4.73	0.21	0.2
3.00	4.25	3.00	2841.67	1.49	12.64	10.59	0.92	0.51
2.00	0.71	2.00	221.43	1.3	12.83	10.69	1.52	0.4
3.00	3.85	3.00	672.31	1.52	10.85	8.79	1.38	0.5
2.00	0.4	1.00	87.13	1.09	9.61	7.59	1.36	0.4
2.00	1.16	2.00	142.03	1.08	5.28	3.6	1.32	0.8
2.00	0.61	2.00	381.63	1.14	15.61	12.75	1.53	0.3
1.00	0.28	1.00	33.38	1.11	4.64	3.24	1.12	0.4
2.00	0.65	2.00	63.89	1.16	3.9	1.79	1.56	0.7
1.00	0.82	2.00	46.89	0.81	2.91	2.15	0.47	0.5
2.00	0.59	2.00	242.86	1.14	22.05	18.87	1.81	0.29
3.00	3.33	3.00	415.56	0.24	4.31	3.3	0.51	0.3
1.00	0.54	2.00	34.84	1.68	3.62	3.06	0.35	0.5
2.00	0.48	2.00	47.58	1.91	2.99	1.49	1.23	0.6
2.00	3.08	3.00	380	0.39	5.81	4.99	0.42	0.4
3.00	1.82	3.00	390.91	1.17	9.12	7.03	1.05	0.4
2.00	0.34	1.00	135.29	1.09	7.07	3.5	2.31	0.4
3.00	0.61	2.00	320.41	1.14	14.35	10.65	2.83	0.3
1.00	0.15	1.00	19.39	0.99	2.92	2	0.5	0.2
3.00	3.08	3.00	872.31	0.82	3.62	2.51	0.88	0.4
1.00	0.34	1.00	26.21	2.14	2.34	1.5	0.59	0.4
2.00	0.36	1.00	78.04	1.07	4.56	2.51	1.39	0.4
2.00	0.31	1.00	124.74	1.38	5.3	3.25	1.26	0.3
2.00	0.41	1.00	82.45	1.14	4.06	2.6	0.94	0.2
2.00	0.42	2.00	59.66	1.43	8.19	5.4	1.63	0.5
2.00	0.71	2.00	110	1.09	8.78	6.49	1.53	0.5
2.00	1.35	2.00	166.15	0.67	13.61	11.61	1.18	0.7
2.00	0.49	2.00	204.88	0.86	3.1	1.8	1	0.2
2.00	0.24	1.00	62.36	2.41	7.44	4.6	1.71	0.3
2.00	0.61	2.00	155.1	1.14	10.5	7.8	1.8	0.3
2.00	0.08	1.00	63.73	3.57	3.37	1.8	1.19	0.1
2.00	0.96	2.00	287.67	1.24	6.69	4.59	1.35	0.7
2.00	0.38	1.00	114.15	2.79	7.57	5.65	1.23	0.4
2.00	1.11	2.00	342.86	1.05	11.13	9.74	0.87	0.31
1.00	0.29	1.00	65	1.35	5.81	4.32	0.94	0.4
2.00	3.89	3.00	496.39	1.55	3.75	1.88	1.18	1.4

3.00	2	3.00	262.67	1.63	8.24	6.18	1.3	0.3
2.00	0.49	2.00	155.74	0.55	26.87	23.67	1.35	0.3
3.00	2.73	3.00	690.91	0.89	7.03	5.5	0.84	0.3
1.00	0.4	1.00	61.11	0.48	2.6	1.54	0.63	0.43
3.00	0.25	1.00	207.5	0.63	9.71	5.28	2.93	0.1
2.00	0.93	2.00	66.05	1.07	3.32	2.01	0.96	0.4
2.00	0.21	1.00	456.25	3.45	5.6	3.46	1.85	0.1
1.00	0.94	2.00	153.13	1.16	4.22	3	0.36	0.6
1.00	0.45	2.00	59.55	1.79	2.49	1.49	0.71	0.4
1.00	0.34	1.00	108.55	2.3	3.87	2.18	1.04	0.4
2.00	1.15	2.00	124.59	1.78	4.31	1.96	1.78	0.7
1.00	0.27	1.00	27.78	1.51	3.12	1.43	1.27	0.5
3.00	2.14	3.00	517.14	0.56	6.22	4.75	0.76	0.3
1.00	0.31	1.00	66.25	1.68	3.95	3.61	0.19	0.2
1.00	0.2	1.00	114	1.42	6.6	5.5	0.6	0.2
3.00	1.54	2.00	1092.31	0.68	7.54	6.34	0.94	0.2
1.00	0.11	1.00	45.26	1.2	4	2.9	0.8	0.2
2.00	0.27	1.00	336.49	2.49	7.8	5.9	1	0.2
1.00	1.11	2.00	73.65	1.68	8.97	8.01	0.54	0.7
1.00	0.08	1.00	26.34	1.84	3.53	2.81	0.51	0.1
2.00	1.39	2.00	1150	1.3	9.58	8.25	0.62	0.25
1.00	0.15	1.00	68.1	3.55	3.11	1.38	1.1	0.2
2.00	0.33	1.00	49.89	1.95	5.65	3.77	1.14	0.3
2.00	1.09	2.00	88	1.31	3.69	2.18	0.94	0.6
2.00	0.72	2.00	70.14	1.28	6.54	4.37	1.3	0.5
2.00	0.75	2.00	145.29	0.84	5.26	1.76	2.28	0.65
1.00	0.22	1.00	52.81	2.18	15.62	13.56	0.94	0.4
1.00	0.6	2.00	64.29	1.49	3.65	3.26	0.36	0.5
2.00	0.25	1.00	77.78	1.48	9.89	8.3	1.16	0.2
3.00	3.08	3.00	464.62	0.54	7.64	4.72	2.21	0.4
1.00	0.31	1.00	72.92	0.41	2.46	1.9	0.34	0.2
2.00	0.82	2.00	55.92	1.14	1.6	0.53	0.6	0.4
2.00	0.77	2.00	406.15	3.59	11.34	8.98	1.34	0.5
3.00	0.43	2.00	469.57	1.66	6.6	4.66	1.26	0.1
1.00	0.53	2.00	47.63	1.23	1.89	1.2	0.49	0.7
2.00	0.42	2.00	84.21	1.35	3.81	1.94	1.35	0.4
3.00	0.61	2.00	248.98	1.14	8.7	5.1	2.24	0.3
2.00	0.12	1.00	52.24	1.4	2.78	1.74	0.83	0.1

1.00	0.35	1.00	192.98	1.01	8.59	7.24	0.62	0.4
2.00	0.99	2.00	491.36	2.48	6.6	4.9	0.9	0.8
1.00	0.23	1.00	120.45	1.56	2.38	1.49	0.65	0.2
2.00	1.43	2.00	226.53	1.14	3.34	1.69	1.42	0.7
2.00	0.82	2.00	49.8	1.14	7.29	5.96	0.7	0.4
2.00	1.21	2.00	152.33	1.51	4.73	3.22	0.78	0.88
2.00	2.73	3.00	201.82	0.89	3.59	2.27	0.88	0.6
2.00	0.86	2.00	67.9	1.61	5.5	4.15	0.89	0.7
1.00	0.25	1.00	24.81	0.72	1.44	0.88	0.36	0.2
2.00	1.18	2.00	652.94	1.44	4.09	3.75	0.28	0.2
2.00	0.51	2.00	60	1.14	5.27	3.62	1.2	0.25
2.00	0.16	1.00	82.87	3.72	5.98	2.71	2.59	0.2
2.00	0.34	1.00	174.58	2.65	13.35	11.91	0.68	0.2
2.00	2	3.00	328.8	4.97	10.55	8.53	0.89	0.5
2.00	0.85	2.00	167.05	0.75	7.4	5.15	1.29	0.52
2.00	0.45	2.00	104.55	0.76	5.89	4.76	0.77	0.3
1.00	0.24	1.00	35	1.95	5.67	3.96	1.02	0.4
1.00	0.77	2.00	162.82	0.65	4.8	4.11	0.1	0.3
1.00	0.34	1.00	65.75	1.94	4.09	2.86	0.79	0.5
1.00	0.44	2.00	67.94	0.79	3.42	2.77	0.49	0.3
2.00	1.03	2.00	92.52	1.37	5.36	3.19	1.63	1.1
2.00	0.47	2.00	154.11	3.4	10.4	3.7	4.9	0.69
3.00	3.47	3.00	377.78	0.85	11.31	8.34	2.16	1.25
3.00	1.02	2.00	148.98	1.14	6.8	4.3	2.1	0.5
2.00	0.17	1.00	299.17	2.79	4.08	1	1.78	0.2
3.00	1.82	3.00	1572.73	0.45	13.08	10.37	1.46	0.2
1.00	0.27	1.00	46.36	1.73	2.9	2.1	0.6	0.3
1.00	0.33	1.00	41.32	1.36	2.09	1.05	0.85	0.4
2.00	0.45	2.00	77.53	1.62	4.17	2.62	0.98	0.4
2.00	0.75	2.00	114.61	2.28	4.4	2.6	1.2	0.67
1.00	0.27	1.00	34.97	1.42	3.52	2.65	0.48	0.4
3.00	2.67	3.00	726.67	1.07	5.18	3.35	1.27	0.4
1.00	0.33	1.00	78.28	1.68	3.87	2.06	0.96	0.4
3.00	0.52	2.00	254.02	1.5	14.81	10	4.3	0.45
2.00	0.41	1.00	100.82	1.14	6.44	5.19	0.75	0.2
1.00	0.26	1.00	66.67	1.45	6.8	4.5	0.9	0.3
1.00	0.2	1.00	40.4	1.62	4.02	2.78	0.9	0.3
2.00	0.24	1.00	201.81	4.77	6.43	3.08	2.66	0.2

3.00	5.56	3.00	1177.78	0.24	7.4	5.65	0.94	0.5
3.00	0.88	2.00	402.94	0.56	12.71	10.4	1.8	0.3
2.00	0.14	1.00	53.96	1.67	5.5	1.5	3.5	0.2
2.00	0.42	2.00	133.8	1.98	6.5	4.52	1.57	0.3
3.00	5.56	3.00	608.89	0.66	6.11	3.29	1.95	0.5
2.00	0.86	2.00	76.21	1.35	4.98	3.77	0.84	0.5
2.00	0.72	2.00	159.42	1.14	10.03	7.48	1.78	0.5
3.00	5.1	3.00	1820	0.57	12.96	9.8	1.66	0.51
1.00	0.38	1.00	64.04	1.1	2.89	1.89	0.62	0.43
2.00	0.59	2.00	147.06	0.71	12.52	9.4	1.64	0.3
2.00	0.77	2.00	89.23	1.44	3.75	2.67	0.88	0.5
2.00	0.33	1.00	191.67	2.14	5.5	3.69	1.45	0.2
2.00	1.02	2.00	133.67	1.14	9.6	7.42	1.01	0.5
2.00	0.61	2.00	153.47	1.14	5.59	4.23	0.87	0.3
2.00	0.56	2.00	625	0.54	10.71	9.4	0.7	0.2
3.00	5	3.00	850	0.82	5.18	3.3	1.25	0.7
1.00	0.22	1.00	35.39	1.47	3.27	1.94	0.73	0.34
2.00	0.21	1.00	192.71	1.47	7.49	5.74	1.42	0.2
2.00	0.68	2.00	72.65	1.53	11.27	9.16	1.17	0.8
1.00	0.51	2.00	24.05	0.57	2.99	1.95	0.66	0.4
1.00	0.41	2.00	42.98	1.6	6.3	4.9	0.8	0.5
3.00	1.54	2.00	114.63	0.72	8.73	4.94	2.87	0.63
2.00	0.29	1.00	71.94	1.5	4.97	1.85	2.12	0.3
2.00	0.34	1.00	83.9	2.31	7.28	3.77	2.24	0.4
2.00	1.9	3.00	976.19	1.01	14.29	12.7	0.73	0.4
2.00	0.27	1.00	67.57	2.16	8.35	6.39	1.6	0.3
1.00	0.13	1.00	58.8	1.97	4.66	1.97	1.94	0.3
2.00	0.87	2.00	297.39	1.42	5.46	4.34	0.61	0.4
2.00	0.48	2.00	60.32	0.87	7.07	5.19	1.21	0.3
1.00	0.51	2.00	114.55	1.78	4.68	3.53	0.64	0.5
1.00	0.54	2.00	45.74	1.22	9.17	7.22	0.8	0.7
1.00	0.35	1.00	45.97	1.89	6.42	4.39	1.56	0.8
2.00	1.21	2.00	118.79	1.18	6.09	4.75	0.77	0.4
2.00	1.27	2.00	120	1.3	5.4	4.1	0.9	0.7
1.00	0.68	2.00	113.39	0.25	1.5	0.83	0.5	0.4
1.00	0.26	1.00	83.85	1.43	3.12	1	1.4	0.5
3.00	1.54	2.00	861.54	1.29	5.88	4.1	0.83	0.2
1.00	0.05	1.00	24.17	1.55	2.42	1.7	0.5	0.1

3.00	0.61	2.00	128.57	1.14	6.1	3.5	2.1	0.3
2.00	0.82	2.00	21.36	1.27	3.02	2.19	0.56	0.36
2.00	0.37	1.00	73.33	1.91	5.13	3.66	0.92	0.3
3.00	5.38	3.00	549.23	0.52	34.02	27.59	4.05	0.7
2.00	0.44	2.00	51.11	1.06	10.4	9.1	1	0.4
1.00	1.15	2.00	62.31	2.07	3.32	3	0.2	0.3
2.00	0.85	2.00	162.03	1.08	7.8	5.9	1.2	0.5
1.00	0.59	2.00	70.59	2.2	2.3	1.32	0.55	0.5
1.00	0.62	2.00	77.24	2.02	6	4.6	0.6	0.9
2.00	0.75	2.00	295.52	1.25	6.98	5.08	1.45	0.5
2.00	0.38	1.00	263.75	1.27	18.11	15.3	1.4	0.3
1.00	0.38	1.00	63.21	1.23	5.4	4.1	0.8	0.4
1.00	0.41	1.00	59.59	1.14	2.82	2.1	0.4	0.2
2.00	0.15	1.00	226.87	2.52	9.6	6.9	2.1	0.2
3.00	0.38	1.00	163.46	1.32	10.7	7.6	2.5	0.2
2.00	0.82	2.00	142.86	1.14	4.2	2.6	1.3	0.4
2.00	0.25	1.00	87.5	0.91	8.3	6.5	1.2	0.22
2.00	1.02	2.00	46.94	0.53	12.4	9	1.4	1
1.00	0.38	1.00	53.46	1.8	3.52	2.02	0.98	0.5
2.00	0.48	2.00	95.24	1.79	6.6	5.4	0.7	0.2
2.00	0.46	2.00	46.81	1.62	4.3	3.1	1	0.43
1.00	0.36	1.00	22.29	1.51	5.4	4.4	0.7	0.5
3.00	1	2.00	162.5	1.04	6.7	3.6	2.5	0.4
2.00	0.25	1.00	108.47	3.09	3.8	1.6	1.7	0.3
2.00	0.19	1.00	45.37	0.75	6	4.3	1.5	0.2
2.00	0.33	1.00	125.2	2.28	3	1.2	1.4	0.4
1.00	0.8	2.00	88.57	1.14	7.87	6.93	0.44	0.39
3.00	4.67	3.00	680	0.85	10.08	8.94	0.79	0.7
2.00	0.37	1.00	151.02	1.14	18.13	15.31	1.07	0.18
2.00	0.7	2.00	231.69	1.44	10.84	7.78	2.33	0.5
3.00	3	3.00	264	0.27	13.12	10.35	1.32	0.3
2.00	1.02	2.00	62.04	1.14	13.63	12.07	0.78	0.5
2.00	0.82	2.00	189.8	1.14	19.7	16.8	1.3	0.4
3.00	4.17	3.00	841.67	0.2	10.42	7.22	1.48	0.5
2.00	1.43	2.00	124	0.73	5.74	3.76	0.9	0.5
2.00	1.02	2.00	70.2	1.14	5.26	4.13	0.63	0.5
2.00	0.75	2.00	225.85	0.85	3.82	2.67	0.8	0.4
3.00	7.5	3.00	637.5	0.07	8.57	6.75	1.24	0.6

2.00	2	3.00	427	2.81	10.87	9.44	0.83	0.4
2.00	0.54	2.00	136.43	0.59	17.71	16.63	0.63	0.3
2.00	1.72	3.00	114.48	0.61	7.4	6.5	0.6	0.5
3.00	2.31	3.00	995.38	0.59	8.87	7.09	0.76	0.3
2.00	0.61	2.00	106.12	1.14	4.76	3.65	0.69	0.3
3.00	3	3.00	1090	0.7	14.51	12.4	1	0.3
2.00	0.71	2.00	153.57	1.71	7.03	5.97	0.66	0.4
3.00	1.43	2.00	175.51	1.14	11.61	7.92	2.68	0.7
2.00	4.44	3.00	2811.11	0.4	21.17	20.18	0.35	0.4
3.00	2.93	3.00	569.33	1.45	7.18	4.92	1.68	0.44
3.00	3.14	3.00	1350	1.89	12.94	10.36	1.61	0.44
2.00	0.62	2.00	29.23	0.52	15.4	13.2	1.1	0.4
3.00	4.55	3.00	872.73	0.96	12.69	11.38	0.58	0.5
3.00	12.86	3.00	405.71	0.14	17.29	15.52	0.87	0.9
3.00	1.25	2.00	617.5	0.28	4.87	3.79	0.53	0.1
2.00	4.29	3.00	828.57	0.34	3.03	2.69	0.28	0.3
3.00	7.56	3.00	911.11	0.97	6.88	4.84	0.95	0.68
3.00	1.22	2.00	393.75	0.88	13.6	10.3	2.4	0.39
3.00	3.33	3.00	756.67	0.46	5.76	3.63	1.53	0.4
3.00	2.27	3.00	1554.55	0.75	29.23	25.54	1.91	0.5
3.00	7.14	3.00	1842.86	0.92	10.92	8.57	1.12	0.5
3.00	4	3.00	272	0.57	26.67	23.73	1.59	0.8
3.00	3.64	3.00	849.09	1.07	15.03	11.72	1.45	0.4
3.00	1.25	2.00	343.75	0.69	15.87	14.69	0.76	0.2
1.00	0.32	1.00	115.23	1.2	5.66	4.89	0.52	0.28
3.00	3.85	3.00	562.31	0.92	4.7	2.86	1.25	0.5
1.00	0.56	2.00	228	0.9	7.3	6.2	0.9	0.7
1.00	1.21	2.00	113.94	0.86	6.26	5.9	0.18	0.4
3.00	1.58	2.00	410.53	1.1	6.6	3.69	2	0.6
2.00	0.27	1.00	209.59	0.84	6.17	3.16	1.94	0.2
1.00	1.88	3.00	151.25	0.37	6.38	5.56	0.15	0.3
2.00	1.18	2.00	161.18	0.78	4.57	3.08	0.83	0.4
1.00	1.59	3.00	79.55	0.61	2.5	2	0.4	0.7
3.00	3	3.00	1091	0.48	7.08	4.83	1.25	0.3
2.00	0.15	1.00	134.33	0.99	9.38	5.12	2.63	0.1
2.00	0.97	2.00	168.75	0.41	6.8	5.3	1.2	0.31
2.00	1.02	2.00	236.73	1.14	9.27	6.82	1.52	0.5
3.00	1.47	2.00	503.33	0.6	17.83	14.98	1.73	0.44

3.00	1.33	2.00	1500	1.05	6.9	4.7	1.11	0.2
3.00	0.86	2.00	108.86	0.95	9.24	6.92	1.58	0.3
2.00	0.88	2.00	28.36	0.52	4.7	3.6	0.7	0.59
2.00	0.2	1.00	395.92	1.14	10.94	8.07	1.29	0.1
3.00	3.85	3.00	1118.46	0.67	6.18	4.62	1.06	0.5
2.00	0.88	2.00	145.29	0.6	8.76	6.55	0.95	0.3
3.00	3.33	3.00	1600	0.65	6.99	5.4	0.83	0.3
2.00	0.8	2.00	63.6	0.72	13.7	11.08	1.23	0.4
2.00	1.03	2.00	156.41	1.14	6.61	5.37	0.67	0.4
3.00	6.88	3.00	1026.25	2.13	11.97	8.43	1.82	1.1
3.00	1.82	3.00	137.58	0.93	21.57	18.17	1.99	0.6
1.00	0.65	2.00	57.41	2.03	1.84	1.14	0.58	0.7
1.00	1.11	2.00	69.52	2.2	1.94	1.1	0.58	0.7
	2.22		382.22	0.48	2.52	1.9	0.5	0.2
	0.67		256.00	0.43	6.56	2.2	3.22	0.1
	1.33		429.33	0.17	25.34	22.25	1.06	0.2
	0.61		77.96	1.14	3.82	3.6	0.1	0.3
	3.75		442.50	1.36	4.54	3.79	0.43	0.3
	1.18		1000.00	0.13	5.86	5.2	0.52	0.2
	0.82		94.69	1.14	7.22	6.17	0.67	0.4
	7.14		905.71	0.3	9.72	8.21	1.07	0.5
	0.25		57.79	0.76	4.73	2.22	1.12	0.3
	1.18		31.96	1.28	3.69	1.98	1.25	1.2
	0.38		201.33	1.74	2.72	2	0.5	0.4
	0.29		61.05	1.79	3.12	2.33	0.44	0.33
	2.50		1650.00	1.05	9.75	7.25	1.41	0.3
	0.16		50.00	1.27	5.6	4.1	1.2	0.2
	0.62		66.77	0.9	10.84	8.8	1.04	0.4
	0.32		104.84	1.65	3.42	3	0.3	0.2
	1.24		300.00	0.8	6.5	5.4	0.7	0.21
	0.38		53.03	1.09	1.72	1	0.6	0.34
	2.50		682.50	2.29	10.84	8.7	1.37	0.4
	0.58		85.51	3.1	6.45	4.67	1.07	0.4
	0.50		160.00	2.19	2.88	2.05	0.52	0.3
	1.67		48.75	0.61	1.19	0.73	0.38	0.8
	0.97		67.58	1.28	2.67	1.17	1.05	0.6
	0.31		52.81	1.35	4.16	2.18	1.32	0.3
	0.82		220.00	1.14	4.07	2.53	0.91	0.4

1.54		1292.31	1.61	5.28	3.06	1.45	0.2
0.67		42.44	0.53	1.9	0.92	0.76	0.6
5.11		1700.00	0.83	24.15	20.65	2.66	0.46
0.57		152.83	1.29	4.58	2.99	1.21	0.3
109.52		371.43	2.02	7.17	6	0.44	23
23.50		4220.00	0.02	10.32	5.27	2.91	0.47
0.31		54.59	0.79	2.29	1.45	0.48	0.26
3.85		626.15	0.73	13.91	11.3	1.02	0.5
0.61		109.49	1.35	5.97	3.6	1.84	0.6
0.96		86.27	1.95	2.07	1.2	0.69	0.49
0.87		51.30	1.46	2.12	1.82	0.13	0.6
0.61		140.98	0.72	3.5	2.33	0.8	0.37
1.67		288.89	1.7	2.03	1.24	0.58	0.3
0.65		70.20	1.14	3.48	1.82	1.03	0.32
0.58		88.35	0.83	18.39	16.38	0.7	0.6
0.61		163.27	1.14	5.03	4.29	0.53	0.3
0.95		18.95	0.85	2.2	1.53	0.37	0.9
0.22		66.45	0.64	3.43	2.3	0.64	0.2
0.36		153.95	1.57	7.56	2.78	3.07	0.31
0.45		77.61	1.46	2.21	1.27	0.68	0.3
0.42		96.67	1.92	4.26	3.53	0.26	0.2
2.73		201.82	0.33	3.88	3	0.59	0.3
0.49		57.07	1.23	3.23	2.36	0.63	0.2
2.14		1478.57	0.96	8.15	6.28	1.28	0.3
0.34		144.07	2.11	4.24	2.98	1	0.2
1.10		351.02	1.14	11.28	7.43	2.27	0.54
0.78		195.00	2.71	8.1	7.1	0.61	0.25
1.33		600.00	0.7	2.44	0.96	0.9	0.2
7.00		514.00	0.61	11.49	10.15	0.38	0.7
0.61		133.44	1.67	6.09	4.93	0.62	0.39
0.58		93.46	0.97	8.62	6.88	0.7	0.3
1.84		132.65	1.14	3.84	1.74	1.36	0.9
0.38		693.85	0.49	12.42	10.69	0.38	0.1
0.56		78.52	1.05	4.91	3.03	1.18	0.3
0.30		52.00	1.85	2.39	1.57	0.66	0.3
0.26		42.15	1.93	3.71	2.5	0.78	0.32
2.31		361.54	0.85	6.35	3.04	2.08	0.6
3.00		3210.00	0.41	14.45	12.84	0.54	0.3

0.45	101.80	4.08	7.15	4.3	1.32	0.6
1.35	66.76	1.75	5.08	4.33	0.35	1
0.20	214.29	1.14	4.4	3.42	0.51	0.1
0.61	90.61	1.14	6.86	5.25	1.12	0.3
4.67	1022.22	0.5	12.02	9.39	1.44	0.42
3.33	575.00	0.81	8.31	6.35	1	0.4
0.35	148.98	1.14	4.01	2.85	0.67	0.17
0.16	88.52	1.4	3.01	1.76	0.95	0.1
0.57	66.79	1.1	3.66	2.38	0.95	0.3
1.58	273.68	0.56	9.71	7.23	1.55	0.3
3.08	938.46	0.79	8.92	7.29	0.6	0.4
0.20	88.57	1.14	8.71	5.63	0.79	0.1
0.20	150.98	1.68	7.05	5.04	1.12	0.1
0.82	136.73	1.14	2.53	0.93	1.25	0.4
0.33	122.33	1	15.02	13.88	0.67	0.2
1.02	39.32	0.72	1.71	0.93	0.44	0.6
1.02	84.49	1.14	8.17	6.96	0.72	0.5
0.85	91.55	1.16	2.84	1.88	0.52	0.6
0.81	22.58	2.08	3.27	2.27	0.3	0.5
0.67	60.53	0.51	2.23	1.31	0.69	0.5
0.20	196.73	1.14	5.09	3.5	1.2	0.1
0.47	111.64	1.42	6.81	2.08	3.19	0.34
2.22	805.56	2.41	16.13	13.22	1.8	0.4
0.61	171.43	1.14	3.87	2.86	0.63	0.3
0.68	147.46	1.37	3.95	2.6	1.07	0.4
11.00	1310.00	1.05	7.17	5.3	0.45	1.1
0.29	66.47	1.68	4.43	2.41	1.28	0.3
0.75	688.33	10.23	6.67	4.02	1.96	0.36
0.43	104.49	1.2	4.06	2.55	0.91	0.3
1.33	230.00	0.54	4.66	3.04	0.91	0.4
0.41	184.49	1.14	17.91	16.99	0.51	0.2
1.11	111.11	0.53	4.28	3.36	0.66	0.3
0.41	102.04	1.14	5.03	3.91	0.82	0.2
0.61	273.47	1.29	6.28	2.36	2.65	0.3
7.78	855.56	0.9	4.36	3.17	0.57	0.7
1.52	239.39	1.24	9.46	8.55	0.66	0.5
0.95	235.71	2.22	17.4	14.8	1.2	0.4
0.30	72.03	1.44	10.27	9.21	0.4	0.4

0.65	23.23	1.35	2.27	1.72	0.41	0.4
0.61	148.98	1.14	12.83	10.63	1.36	0.3
0.54	242.86	1.42	4.88	2.94	1.45	0.38
1.07	87.14	0.75	1.27	0.85	0.26	0.3
3.57	435.71	0.73	9.1	5.5	2.1	0.5
2.00	166.00	0.45	5	4.4	0.3	0.4
3.08	216.92	0.64	4.1	3.52	0.38	0.4
0.45	172.73	1.89	3.4	1.6	1.5	0.5
3.33	1633.33	0.7	14.2	11.56	1.12	0.2
8.00	400.00	0.26	3.38	2.67	0.21	0.4
0.09	178.57	2.08	5.26	2.88	1.05	0.1
0.67	151.58	1.72	6.56	3.85	1.88	0.38
1.83	156.67	1.42	5.38	3.99	1	1.1
1.63	154.38	2.84	2.92	2.09	0.48	0.52
3.85	128.46	0.41	6.54	4.87	0.88	0.5
1.12	84.86	2.4	3.93	2.6	0.9	1.2
0.37	51.11	2.54	11.8	10.94	0.52	0.3
3.33	304.44	1.13	9.97	8.28	0.73	0.3
0.59	115.29	0.43	3.72	3	0.2	0.2
0.50	115.00	1.57	2.49	1.11	0.77	0.6
1.94	494.44	1.84	23	21.9	0.9	0.7
0.61	585.71	1.14	3.96	2.36	1.11	0.3
0.16	53.47	0.97	6.42	5.78	0.31	0.2
3.33	1058.33	0.97	6.58	5.23	0.92	0.4
1.58	128.16	0.13	3.62	2.83	0.31	0.6
0.32	145.16	0.82	8.16	4.05	3.7	0.3
0.82	148.98	1.14	5.37	4.45	0.76	0.4
2.22	1715.56	0.25	2.7	1.69	0.68	0.2
0.31	216.56	1.28	7.76	5.96	1.22	0.2
1.14	101.43	2.73	6.28	5.09	0.78	0.8
0.41	24.08	1.14	2.67	1.79	0.52	0.2
0.23	69.77	3.23	3.82	2.55	0.78	0.3
2.78	900.00	0.55	4.02	2.54	0.86	0.25
0.44	45.19	1.57	3.12	1.58	1.21	0.48
0.15	81.41	0.98	6.28	2.69	2.53	0.27
1.79	251.28	3.41	4.1	2.8	0.7	0.7
0.77	288.46	0.38	3.96	2.94	0.2	0.2
2.50	925.00	1.37	6.61	4.89	0.84	0.4

0.46	101.61	1.16	3.74	1.43	1.76	0.4
0.18	53.08	1.54	3.79	2.97	0.4	0.14
0.43	133.70	2.35	8.46	7.06	1.06	0.4
0.61	86.53	1.14	4.13	2.69	0.76	0.3
0.86	42.00	1.25	4.56	3.36	0.97	0.6
0.23	25.49	2.24	2.52	1.9	0.4	0.33
1.11	270.37	0.69	3.17	1.76	1.08	0.3
0.94	158.49	1.12	3.11	1.99	0.87	0.5
0.35	69.57	1.34	4.3	2.7	1.2	0.4
1.92	411.54	2.3	5.5	3.43	1.58	0.5
0.61	47.76	1.14	2.57	1.81	0.53	0.3
4.00	284.00	0.55	4.87	4.27	0.49	0.4
0.28	294.44	2.92	3.13	2.04	0.83	0.1
0.69	31.78	0.4	2.87	1.68	0.92	0.7
1.02	208.16	1.14	3.93	3.01	0.62	0.5
0.75	273.75	2.43	4.61	3.75	0.56	0.6
3.25	458.33	1.42	3.39	2.47	0.69	0.39
4.00	700.00	1.57	4.14	2.83	0.78	0.4
0.11	56.14	1.52	4.49	3.41	0.61	0.1
0.57	271.70	1.02	8.89	6.71	1.56	0.3
1.74	634.78	0.84	7.4	5	1.7	0.4
3.33	225.83	2.2	5.18	4.14	0.58	0.8
0.14	66.67	0.63	3.15	1.98	0.6	0.2
0.11	39.33	1.88	5.31	4.19	0.68	0.1
0.40	45.33	1.03	4.56	3.75	0.47	0.3
1.02	218.37	1.14	5.6	2.53	1.98	0.5
0.50	124.17	2.39	4.1	3.12	0.46	0.6
0.36	33.66	1.17	1.99	1.37	0.34	0.4
0.47	57.44	2	4.75	3.07	1.18	0.4
0.98	47.84	1.91	3.06	2.24	0.32	0.5
0.30	56.10	3.45	9.68	8.11	0.55	0.5
0.29	43.43	2.01	10.82	8.21	2.34	0.2
0.18	43.09	2.63	5.05	4.14	0.54	0.2
1.02	232.65	1.14	4.77	3.6	0.63	0.5
0.65	134.52	1.14	3.35	1.05	1.69	0.4
0.39	34.69	2.94	4.16	1.85	1.77	0.5
0.83	57.08	0.89	5.57	4.91	0.58	0.4
0.41	55.92	1.14	2.14	1.46	0.46	0.2

0.96	115.07	1.43	7.76	5.36	1.49	0.7
0.56	79.63	1.64	6.83	5.49	0.87	0.6
0.68	65.05	1.82	5.82	4.4	1.06	0.7
0.97	416.13	0.79	14.37	12.78	0.65	0.3
0.49	156.39	1.72	3.01	2.36	0.5	0.3
0.47	84.65	0.83	2.35	1.33	0.61	0.2
0.50	38.61	2.02	4.3	2.2	1.9	0.5
0.49	213.90	0.89	9.1	7.14	1.32	0.2
0.98	143.90	2.45	4.74	2.86	1.24	0.4
0.35	154.39	1.29	2.2	1.3	0.67	0.2
0.34	61.67	2.09	2.46	1.51	0.64	0.45
0.58	58.55	1.29	2.51	1.78	0.59	0.4
1.05	101.75	0.48	16.27	13.51	1.74	0.6
0.35	128.24	1.59	4.4	3.08	0.8	0.3
0.63	47.79	1.77	4.32	3.44	0.66	0.6
0.70	49.65	1.6	3.14	2.4	0.36	1
0.31	26.94	1.96	1.38	0.83	0.45	0.3
0.85	83.05	1.53	3.09	1.83	0.71	0.5
0.68	75.68	1.41	10.07	8.26	0.99	0.5
1.82	233.33	1.5	4.13	2.49	1.21	0.6
0.28	59.72	1.86	5.54	4.57	0.6	0.2
0.20	89.00	1.66	7.7	5.3	1.4	0.2
0.51	97.69	1.45	7.95	5.76	1.33	0.4
1.00	240.67	1.01	4.72	2.88	1.18	0.3
0.89	221.43	1.45	10.94	8.11	1.92	0.5
0.19	29.23	1.9	2.7	1.35	0.8	0.2
0.20	46.26	1.18	2.49	1.32	0.81	0.3
0.75	126.88	1.17	6.89	4.78	1.25	0.7
0.82	318.37	1.14	4.5	3.7	0.5	0.4
0.21	122.38	1.21	4	1.36	1.9	0.3
0.24	57.72	2.79	2.87	2.09	0.48	0.3
0.41	97.35	2.82	4.02	2.29	1.16	0.4
0.61	385.71	1.14	6.08	5.49	0.25	0.3
0.82	39.59	1.14	1.82	1	0.45	0.4
0.37	185.98	1.92	14.27	12.08	1.56	0.4
1.00	203.33	2.37	6.06	2.23	2.26	0.6
0.28	26.06	1.84	2.23	1.35	0.65	0.3
0.61	79.80	1.52	13.23	11.87	0.64	0.6

1.43	70.20	1.14	4.37	2.96	1.06	0.7
0.44	87.56	0.77	8.16	5.39	1.81	0.2
0.65	98.70	2.11	5.34	2.8	1.98	0.5
0.34	68.97	1.02	3.32	2	1.1	0.4
0.42	63.17	1.78	3.43	2.32	0.55	0.5
0.42	41.86	1.31	2.29	1.82	0.29	0.5
0.75	203.50	1.41	2.47	1.9	0.39	0.3
0.68	153.42	1.12	7.94	5.75	1.71	0.5
0.91	137.58	0.86	3	1.57	1.15	0.3
0.69	48.74	1.15	6.99	5.31	1.07	0.6
2.50	519.38	3.27	4.17	2.36	1.33	0.4
0.33	116.13	1.32	9.12	6.14	2.24	0.41
0.18	49.54	2.46	2.9	2.07	0.56	0.2
1.33	177.78	1.22	8.48	4.74	2.8	0.6
0.48	132.26	2	4.5	3.3	0.6	0.3
0.35	46.71	1.33	3.74	2.81	0.62	0.3
0.37	47.71	0.78	5.63	4.21	0.68	0.4
0.44	158.82	1.07	8.16	7.09	0.84	0.3
0.15	125.76	1.82	4.01	2.16	1.23	0.1
0.56	67.29	1.59	4.7	3.06	0.68	0.6
0.71	187.50	1.14	4.62	3.38	0.76	0.34
0.13	74.76	1.52	3.52	2.48	0.76	0.13
5.83	1420.00	1.12	9.35	8.03	0.84	0.7
0.44	56.67	1.09	12.95	11.27	0.53	0.29
0.48	108.33	1.83	8.52	7.54	0.94	0.4
0.60	101.20	1.67	5.13	4	0.9	0.5
0.51	53.08	0.88	6.02	4.71	0.96	0.4
0.39	91.81	2.33	5.28	1.87	2.06	0.6
0.11	270.21	1.91	8.54	6.3	1.29	0.1
0.63	94.74	1.17	4.01	2.17	1.33	0.36
0.79	183.42	0.7	5.33	3.94	1.03	0.6
0.67	26.37	1.08	6.66	5.88	0.27	0.9
0.24	29.59	2.14	3.51	2.46	0.47	0.3
0.53	79.79	1.77	10.15	9.29	0.6	0.5
0.94	336.73	1.14	8.53	7.04	1.1	0.46
10.91	1963.64	0.89	31.72	27.19	1.42	1.2
0.47	53.95	1.19	3.96	2.55	1.03	0.4
0.82	108.16	1.14	5.48	4.1	0.87	0.4

0.08	81.10	2.95	7.4	5.63	1.2	0.1
0.45	49.85	2.01	2.57	2.08	0.31	0.3
0.33	53.72	1.6	4.34	1.86	1.88	0.4
0.45	120.45	1.8	4.49	2.14	1.63	0.4
0.65	94.46	1.33	4.11	1.9	1.63	0.6
0.11	129.47	0.95	6	4.7	0.8	0.1
0.38	73.46	0.76	3.22	2.68	0.27	0.4
1.34	48.20	2.39	2.57	1.92	0.37	0.82
0.26	146.55	1.51	5.93	4.51	0.69	0.3
0.61	35.51	1.14	1.66	1.1	0.39	0.3
0.29	140.36	2.72	5.83	3.98	0.98	0.4
0.88	66.25	1.95	2.9	2.2	0.5	0.7
0.51	53.97	1.2	3.46	1.38	1.62	0.4
0.38	39.62	1.73	1.82	1.4	0.3	0.4
0.14	11.61	2.11	3.82	2.1	1.4	0.3
0.53	41.87	0.85	2.49	2.12	0.21	0.4
0.24	45.16	1.25	2.91	1.66	0.81	0.3
0.31	59.69	1.38	5.9	4.4	0.9	0.4
0.35	10.88	1.53	3.14	1.54	1.24	0.4
0.53	125.09	1.47	7.54	5.26	1.21	0.3
0.71	70.57	0.92	5.3	4.47	0.54	0.5
2.31	249.23	0.46	9.63	8.1	0.98	0.3
3.85	672.31	1.52	10.85	8.79	1.38	0.5
1.15	115.38	0.37	4.97	4.59	0.17	0.6
0.28	33.38	1.11	4.64	3.24	1.12	0.4
0.53	49.36	0.95	1.97	0.89	0.78	0.5
0.31	124.74	1.38	5.3	3.25	1.26	0.3
0.41	43.67	1.14	2.45	1.83	0.29	0.2
0.08	63.73	3.57	3.37	1.8	1.19	0.1
0.77	62.05	2.13	3.95	3.02	0.48	0.6
0.41	31.37	1.09	1.96	0.99	0.81	0.3
0.37	72.30	1.68	7.1	5.6	1.1	0.55
1.47	163.24	1.48	3.33	2.41	0.54	1
0.48	29.23	1.37	1.56	1.14	0.3	0.5
0.39	452.75	2.28	7.15	3.33	2.32	0.2
0.54	61.43	1.21	3.69	2.25	0.91	0.3
0.27	336.49	2.49	7.8	5.9	1	0.2
0.61	59.32	1.82	6.31	4.8	0.55	0.9

0.60	65.48	0.68	4.73	4.15	0.38	0.5
0.61	85.31	1.14	3.62	1.99	0.65	0.3
0.90	70.45	0.98	4.6	3	1.3	0.6
0.82	49.80	1.14	7.29	5.96	0.7	0.4
0.39	48.43	2.1	3.33	1.77	1.12	0.4
1.82	184.85	0.59	2.44	1.71	0.6	0.6
0.20	101.90	2.42	3.83	2.07	1.25	0.21
0.61	153.47	1.14	5.59	4.23	0.87	0.3
0.78	27.11	1.27	1.8	1.12	0.46	0.7
0.51	24.05	0.57	2.99	1.95	0.66	0.4
0.20	22.86	1.35	5.67	3.7	1.38	0.2
1.05	308.38	1.26	7.08	5.82	0.81	0.39
0.50	84.15	2.42	3.92	3	0.5	0.41
0.41	121.22	1.14	1.97	0.81	0.66	0.2
0.51	114.55	1.78	4.68	3.53	0.64	0.5
3.24	223.53	0.61	8.83	6.36	1.68	1.1
0.31	40.39	0.65	1.95	1.65	0.15	0.24
2.56	390.77	2.68	5.18	3.99	0.65	1
0.38	63.21	1.23	5.4	4.1	0.8	0.4
0.61	57.55	1.14	3.12	1.7	1.1	0.3
3.00	264.00	0.27	13.12	10.35	1.32	0.3
0.24	52.38	0.41	4	2.9	0.7	0.2
3.00	610.00	0.55	7.41	6.43	0.9	0.3
2.93	569.33	1.45	7.18	4.92	1.68	0.44
3.08	476.92	0.53	23.08	18.71	2.17	0.4
1.82	509.09	0.92	2.49	2.31	0.15	0.2
1.29	503.23	1.73	24	21.3	1.6	0.4
6.67	1862.22	0.26	9.74	7.53	1.08	0.6
2.27	1554.55	0.75	29.23	25.54	1.91	0.5
3.85	562.31	0.92	4.7	2.86	1.25	0.5
0.56	228.00	0.9	7.3	6.2	0.9	0.7
3.00	1091.00	0.48	7.08	4.83	1.25	0.3
4.12	1055.29	0.65	7.92	6	1.1	0.7
2.41	90.34	0.25	2.12	1.6	0.2	0.7
1.22	114.49	1.14	7.4	6.38	0.34	0.6
0.88	145.29	0.6	8.76	6.55	0.95	0.3
0.65	57.41	2.03	1.84	1.14	0.58	0.7

NLR	RBC	HGB	HCT	PLT	PT	PTA	INR	**Na**	**BUN**
10.00	4	104	28.8	190	17.2	49.5	1.39	138.7	10.87
6.13	3.48	96.2	31.2	78	15.1	60.6	1.23	133.4	8.43
6.43	2.42	79.2	23.62	112	16.5	52.8	1.42	143.5	14.41
0.68	3.13	109	36.9	38.4	23	30.7	1.97	150.3	33.7
1.94	0.8	25.2	7.72	75	14.6	65.2	1.26	143.5	9.84
6.87	1.83	64.2	20.22	79	23.3	30	2	130.2	11.59
5.73	0.77	25.2	7.22	40.4	30.7	19	2.62	129.3	54.1
11.96	1.21	35.2	11.02	148	23.7	30.2	2.03	137.8	16.61
3.48	1.33	38.2	12.22	86	16.2	55	1.39	139.7	26.7
6.13	2.94	103	29.1	40.4	22.4	33.1	1.92	132.3	3.61
2.03	2.64	100	28.2	19.4	51.8	8.8	4.4	117.4	12.12
3.92	3.4	105	29.4	201	53.3	8.4	4.53	144.9	4.94
28.61	2.86	102	28.4	59.4	33.4	17.6	2.85	125.6	10
4.56	2.86	93.4	24.62	102	18.5	51	1.71	138.4	17.33
13.72	1.05	36.2	11.12	57	37.6	22	3.48	124.4	25.4
2.72	1.79	67.2	19.92	139	27.6	32	2.56	136.5	15.63
15.42	3.97	123	34.7	146	14.2	70	1.31	137.5	9.74
17.66	4.2	138	38.7	56.4	42.3	18	3.92	137.4	13.33
36.00	1.57	66.2	18.62	38.2	27.9	23	2.21	132.9	15.13
10.67	2.71	81.2	24.42	58	39.8	13.1	3.11	128.7	21.66
4.00	1.7	65	18.1	33	33.1	17.9	2.61	129.8	18.71
8.86	3.25	92.2	28.5	161	26	28.3	2.22	132.7	27.82
32.78	2.56	102	29.5	54	18.7	43.9	1.6	148.7	22.99
18.50	3.58	108	31.3	21.4	15.9	56.7	1.37	129.1	14.49
8.34	3.24	111.4	28.8	124	24.4	28.9	2.09	118.8	28.09
10.00	2.98	94	28.4	170	18.5	50	1.61	137.1	19.03
5.75	3.31	109	31.5	86	24.8	28.2	2.12	142	21.34
9.21	2.86	87	27.6	46.4	20.9	36.9	1.79	150.5	7.68
28.61	2.99	102.4	27.2	40.4	28.7	30	2.66	128	16.74
9.71	1.48	56.2	16.42	42.2	27.4	30	2.51	118	10.08
4.61	1.71	67.2	19.42	64	29.7	28	2.61	139.5	8.17
1.32	3.26	95	27.4	54	21.2	40	1.96	123	11.64
3.71	4.25	129.3	37.46	54	46	13	3.57	141.3	1.66
17.92	2.27	78.7	22.82	22.6	26.7	24.7	2.12	129.8	19.07
1.84	3.23	113.7	32.72	40.8	17	50.4	1.38	143.6	6.88
0.75	3.34	109	30.4	49	25.3	20.5	2.05	134.9	4.61
1.94	4.21	126	37.4	69	17.5	48.2	1.41	136.2	3.75

4.38	3.43	116	32.6	34.2	35.5	15.7	2.79	132.7	4.37
3.00	3.7	122	35.1	140	17.2	49.5	1.39	140.9	7.28
6.87	4.21	132	36.3	81	23.7	30.1	2.03	127.2	8.25
5.33	2.95	80	22.7	55	18	46.1	1.45	139.1	6.71
13.57	2.33	91	23.6	99.6	23.2	30.8	1.85	124.7	29.84
3.29	3.07	78	22.7	83	18.7	43.4	1.51	141.8	3.18
21.18	3.89	117	33.3	115	35.4	18.3	3.02	128.1	9.79
26.33	2.17	61.2	18.82	47.2	27.2	24	2.16	120.6	28.25
5.14	3.81	116.4	30.9	198	17.5	48.3	1.5	135.8	4.08
17.43	3.63	122	35.8	48.4	15.5	59	1.33	133.3	9.47
11.33	1.7	66.4	18.58	56.3	30.2	20.3	2.39	126.5	10.11
3.00	2.58	81	22.2	88	17.2	49.5	1.39	109.6	17.15
15.44	3	108.2	31.1	78	22.5	32.4	1.8	137.7	7.31
7.75	3.36	122	35.5	42.2	18.4	47	1.58	135	21.22
9.29	2.73	88.2	24.62	45.2	46.7	10.5	3.63	129.9	10.25
3.56	2.91	101	26.9	26	25.3	27.2	2.01	128.4	13.79
24.81	3.23	125.3	34.88	19.6	48.9	15	3.79	137.7	7.42
17.00	2.25	84.2	22.32	40.2	26.4	25.5	2.1	126.3	21.7
1.35	3.75	130	37.6	17.2	18	53	1.4	132.3	12.66
20.11	2.13	81.1	23.54	26.7	39.9	13.4	3.12	125.2	14.67
24.00	4.02	125	36.4	49.2	18	46.1	1.45	126	5.84
3.42	3.36	123	35.2	64	14.5	64.5	1.18	129.4	14.71
21.18	0.91	35.5	9.98	24.7	18.4	47	1.58	122.9	29.74
10.00	4.03	113	33.9	65	14.9	61.8	1.21	135.8	5.51
9.00	3.76	115	35.1	14.2	25.9	26.2	2.06	129.7	10.09
3.83	3.02	96.2	28.6	50	18.7	43.5	1.51	128.2	9.41
11.73	1.93	69.2	20.52	81	35.5	16.1	2.75	131.5	43.72
9.20	4.23	127	37.4	37.2	21.9	34	1.75	135.1	4.64
4.00	1.25	40	12.7	9	26.1	25.9	2.08	128.2	27.74
4.60	2.91	78.2	24.52	59	17.2	49.5	1.39	136	7.47
3.67	2.75	81.2	26	45.2	14.3	76	1.09	137.2	7.29
11.36	3.84	122	35	224	19.6	40.4	1.68	126.4	10.15
6.21	1.46	57	16.8	88	20.5	41	1.79	130.2	10.95
3.31	4.72	138	41.2	97	28.6	22.5	2.44	139	2.77
31.80	1.92	60.2	19.02	45.2	22.5	32.6	1.93	139.8	18.64
14.81	2.85	86	27	146	15.7	58.1	1.35	127.1	13.15
2.43	3.55	124.9	35.82	60.9	15	67	1.13	155	4.31
7.83	3.88	127	36.4	120	22.9	39	1.58	133.7	4.07

3.88	3.22	114.2	34.06	61.3	12	93	0.94	143.9	3.75
4.36	3.99	121	36.3	59	15.5	59.2	1.33	141.3	6.62
6.92	4.44	140.4	37.9	172	26.6	27.4	2.27	128.2	3.58
3.78	3.81	99	31.6	95.4	16.6	53.7	1.43	136.1	3.18
4.53	2.47	56.2	19.42	131	17.9	48.2	1.54	127.3	7.17
23.13	2.22	76.2	22.32	42.2	25.8	34	1.74	126.1	19.71
3.58	2.87	89.4	25.55	42.4	20.2	40.6	1.73	139.6	4.93
6.93	3.82	123	36.2	44.2	14.9	62.6	1.28	139.2	9.82
8.40	3.18	104	29.5	62	30.2	19.5	2.58	135.7	7.38
2.78	2.97	91.9	26.83	107.8	17.8	54	1.29	129.7	7.51
9.59	2.02	59.2	18.12	75	20.1	38.4	1.72	142	9.86
1.88	3.46	119.4	32.5	44.4	17.5	48.3	1.5	141.2	4.67
21.30	3.4	117	32.3	29.4	26.9	23.7	2.3	119.5	24.91
2.00	2.43	67.2	19.08	78.1	17.3	49.2	1.49	143.6	11.97
9.21	3.7	118.4	34.14	51.8	23.3	30	2	139.9	9.01
13.80	3.03	105	30	33.4	18.2	45.2	1.56	134.3	6.75
2.70	3.29	122.8	32.31	112.3	49.4	16	2.93	140	2.16
3.47	3.82	121	34	68.4	54.1	7.4	4.59	132.9	5.2
4.53	4.65	147.4	39.9	55.4	19.9	39	1.71	136.3	4.96
4.37	2.72	91	26.2	27.4	26.8	32	2.08	140.9	5.38
3.86	3.87	94	30.2	32.4	15.2	61	1.31	139.2	5.48
4.77	4.75	157	45.3	154	40.1	12.2	3.41	134.1	5.18
7.94	2.54	96	27.5	52	37.7	13.5	3.21	125.6	28.75
5.60	2.9	81	23.52	22.4	18.1	45.7	1.55	132.7	13.45
14.03	1.41	52.2	16.02	164	23.5	29.6	3.01	143.1	20.36
4.33	2.94	111	33.3	78	15	62.3	1.29	137.2	11.24
8.16	4.81	166	46.2	53.4	28.6	21.4	2.44	130.6	11.09
7.88	4.32	125	35.3	84	14.9	63	1.28	129.3	7.31
3.30	3.64	113	32.3	46.4	20.6	36.8	1.77	133.2	3.13
6.43	2.59	88	26.2	54	16.9	51.2	1.45	150.2	18.16
1.05	3.32	119	34	106	18.4	47	1.58	140.5	6.44
5.39	2.81	107	31.2	39.4	20.7	36.5	1.77	135.8	9.42
2.55	3.13	103.4	28	79.4	19.8	39.3	1.7	131.5	12.89
13.64	4.85	163	44.9	78	30.3	20.5	2.59	136.6	6.21
3.85	2.88	85	25.02	119	18.1	46.2	1.55	140.9	6.4
1.77	3.16	113	31.1	55	34	24	2.17	129.5	6.68
1.81	2.13	82	23.62	84.4	14.5	65.5	1.25	135.2	9.52
1.76	3.47	120.9	35.15	48.8	29	29	1.91	139	4.36

1.44	3.35	65.2	21.62	135.4	19.5	48	1.39	142.3	6.15
1.28	2.78	102.4	27.5	56	18.5	44.7	1.59	123.6	8.14
2.81	2.27	85	24.52	25.4	23.1	31.5	1.98	129.9	34.15
3.61	4.28	102	31.1	155.4	15.5	59	1.33	135.8	5.96
2.03	3.77	129.2	35.76	59.7	27.1	31	1.99	135.3	3.06
3.02	2.52	90	25.72	46.4	17.6	53	1.41	132.7	6.61
11.08	4.62	143	39.4	81.4	16	56.1	1.38	137.2	14.03
5.30	2.17	83	24.12	27.4	26.3	25.7	2.25	132.3	6.88
2.33	5.28	156	45.9	116	16.5	53.5	1.42	137.9	4.6
24.75	3.65	127	35.6	26.4	17.3	49.6	1.49	128.4	6.8
1.52	3.72	129.3	38.25	47	14.9	54	1.23	138.4	2.6
4.62	2.51	74.7	23.4	196.1	14.1	68.5	1.21	143.1	14.12
1.66	2.47	86	25.42	72.4	12.8	79.7	1.1	133.3	6.98
1.77	2.01	58.2	18.02	34.4	13.8	70.8	1.19	144	5.47
5.18	3.31	109	32.4	33.4	28.2	23	2.41	140.2	6.83
3.41	1.32	36.2	12.92	64	30.7	20.1	2.62	142.1	21.47
2.11	4.28	146	41.6	113.4	14.9	62.8	1.28	132.1	3.37
1.74	2.98	113.9	34.55	74.5	17.5	43	1.4	128.2	6.33
2.30	3	108	31.8	51	15	62.1	1.29	142.4	5.87
1.37	4.12	135	38.4	48.4	18.6	44.3	1.6	139.6	4.23
6.61	2.65	104.4	28.3	102	23.7	30.2	2.03	125.4	5.49
1.11	3.08	99	30.1	152	10.9	106	0.94	136.9	16.25
2.54	1.95	72.2	20.92	57	36.7	15.2	3.13	127.2	5.4
5.09	4.61	147	41.8	139	21.2	36	1.82	139.5	4.57
1.08	3.27	115	33.2	43.4	22.5	32.8	1.93	140.1	3.34
2.27	2.39	82	23.42	36.4	15.9	56.7	1.37	129.8	3.53
3.25	3.92	137.3	39.25	108.4	46.1	17	4.09	131	4.22
1.20	3.4	120	34.7	38.2	15	66	1.3	141.6	3.45
3.64	2.42	89	24.92	38.4	17.7	47.9	1.52	126	5.43
6.30	4.31	133	38	34.4	31.2	19.6	2.66	135.7	3.46
9.54	1.68	60.5	17.34	36.3	19.4	47	1.69	136.8	6.56
19.27	4.86	147	44.5	155	24.3	29.1	2.08	142.4	7.35
11.08	2.52	105	30.1	64	19.9	39.8	1.71	128.9	8.88
9.33	3.08	109	32.3	28.4	25.3	27.3	2.16	121.4	14.28
8.71	3.07	94	28.1	13.4	22.7	32.4	1.94	135.4	25.11
5.14	2.15	72.2	21.92	69	19.2	42.1	1.65	128.4	15.78
1.87	1.93	83	23.42	52	14.6	64.8	1.26	133	6.84
6.85	4.2	135.4	36	101.4	20.6	37.7	1.77	140.5	3.39

13.58	3.26	114	33.6	46.4	17.5	48.7	1.5	137.4	7.23
5.08	3.87	117.3	35.43	22.2	29.1	21.9	2.49	131.4	16.94
4.28	2.87	91.7	26.17	56.3	16.2	61	1.39	138.1	5.39
18.32	4.52	148	42.3	59	37.6	14.6	3.2	136.1	5.14
3.91	3.79	133	36.4	62.4	51.9	8.8	4.41	133.8	20.84
7.85	2.23	78.2	23.29	150.7	14.2	72	1.22	139.8	7.17
2.21	4.09	126.8	37.88	210.7	13	82	1.12	140.6	6.53
1.47	3.52	133	37.2	39.4	17.4	49.2	1.49	133.5	7.54
4.23	2.13	67.2	19.92	96	14.9	62.8	1.28	145.3	11.73
3.67	1.83	62.3	18.32	42.4	23	37	1.93	139.7	7.36
2.43	3.47	120.4	32.8	34.4	18.2	50	1.54	138.5	5.18
3.27	2.17	67.2	21.12	172	14.7	64.1	1.26	154.3	16.17
1.49	3.96	121	35.7	150	19.7	40.5	1.69	131.5	4.52
2.01	3.17	106	32.4	82	36	15.7	3.07	133.4	15.32
13.44	1.94	68.2	18.82	58	17.5	48.7	1.5	127.1	15.37
5.35	3.26	113	32.9	47.4	23.3	31.1	2	142.9	7.72
11.64	3.05	94	29.2	62.4	13.3	75.1	1.15	131.5	13.52
11.84	3.55	117	34.3	39.4	14.2	63	1.31	141.5	3.05
0.89	2.19	84.9	25.94	44.8	12.7	75	1.17	144.3	8.49
5.03	4.21	142.6	42.93	90.4	40.3	17	3.63	138.2	9.06
1.77	3.08	114.6	33.45	30.2	15.2	57	1.4	137.8	6.87
1.07	2.87	95	29.9	90	19.6	40.8	1.68	143.5	2.54
8.34	3.96	127.4	33	266	20.8	37.1	1.78	130.7	15.88
3.50	2.4	70.2	20.82	7.4	14.6	61	1.34	130.9	13.76
26.71	4.63	141	38.7	51.4	19.9	39.8	1.71	129.7	5
7.24	1.82	62.2	19.52	88	16.5	53.5	1.42	133.7	26.48
3.97	4.08	138.4	37.3	113	38.2	14.3	3.25	130.8	3.44
5.63	1.6	61.2	18.04	58	40.9	12.8	3.48	130.9	18.78
7.95	3.61	91	28	85.4	17.8	47.5	1.53	139.7	4.72
9.20	3.75	116	33.1	133	23.8	30	2.04	139.2	3.75
4.61	2.27	88	25.52	19.4	22.8	34	2.08	142.6	2.94
5.92	1.75	68.3	20.06	43.9	18	46.6	1.55	129.2	9.42
4.43	2.19	96.4	25.82	41.4	23.6	30.4	2.02	133.3	26.56
7.34	3.26	105.4	28.7	90	22.7	34	2.07	135.6	12.32
3.71	1.34	50.2	14.32	36.4	19.5	41.1	1.67	133.4	10.83
10.20	4.13	151.4	41.2	79	19.1	42	1.75	133.7	9.48
9.83	1.73	39.4	12.86	48.6	20	39.5	1.72	138.3	17.47
6.16	3.24	82	25.62	54.4	23.6	30.4	2.03	133.7	10.58

3.58	3.44	117	33.4	119	19	42.8	1.63	133.6	5.88
1.59	0.93	50.2	13.82	63	20.7	37.4	1.77	128.1	16.67
2.14	2.34	81.1	24.18	21.8	14.3	63	1.32	144.8	21.65
4.26	3.96	125.2	37.41	82.7	41.5	12.5	3.53	126.9	6.42
4.16	2.96	106	29.4	63	19	42.8	1.63	131.1	6.69
15.77	2.82	89	25.62	36.4	24.1	29.5	2.06	148.2	15.14
24.91	3.33	122	34.5	62	22.8	34	2.08	129.1	7.15
2.75	4.47	142	39.5	329.4	17.4	52	1.61	129.6	6.34
1.28	2.34	74.2	22.52	65	17.9	47	1.54	140.2	5.19
1.45	4.71	147	43.1	45.4	14	69.3	1.21	138.4	4.04
1.91	2.72	102	29.1	64	17.1	53	1.58	151.7	4.22
3.05	4.16	130	38.7	90.6	45	17	4.17	138.8	1.78
5.02	1.85	69.2	19.62	93	18.3	45.4	1.57	124.3	22.4
5.39	2.32	91	25.42	29.4	20.5	38	1.76	127.5	5.39
2.38	3.36	110	33.1	52	14	69.3	1.21	145.2	9.88
1.46	3.53	114.4	30.4	94	44.2	18	4.09	139	2.94
23.78	1.99	73.2	20.72	321	18.5	44.7	1.59	129.3	11.52
31.10	1.93	78.2	23.72	48.4	21	36.6	1.8	132	20.56
7.27	4.69	152	41.8	107	13.4	74.2	1.15	127.7	4.25
5.05	1.79	50.2	15.22	21.4	14.1	68.5	1.21	138.5	53.65
24.32	2.75	96.4	25.82	37.4	21.8	34.5	1.87	103.7	6.31
12.37	3.54	105	31.7	49.4	18.4	47	1.58	132.6	14.23
5.99	1.98	70.2	20.12	65	18.3	45.4	1.57	136.4	15.82
2.66	3.98	128.4	34.9	33.4	25.5	33	2.36	129.2	12.37
6.71	2.25	54.2	17.02	105	14.5	65.5	1.25	138.6	9.27
2.95	4.91	149	41.9	86	24.1	29.5	2.06	134.7	5.92
0.65	1.79	73.2	20.12	76	16.9	51.5	1.45	140.9	4.35
0.94	4.21	143	40.7	80	16.3	56	1.51	141.6	2.89
6.40	1.86	69.4	18.52	24.4	44.3	17	4.1	123.8	4.33
9.94	2.59	83	23.72	55	22.9	31.9	1.96	122	8.46
11.14	2.51	83	23.52	214	14.6	64.8	1.26	140.1	21.74
4.25	3.59	120	34.3	133	29	22	2.48	137.3	6.48
8.20	2.04	66.4	17.02	66	27.7	23.7	2.37	128.1	10.94
2.00	3.87	114	37.2	20	17.8	50	1.65	141.2	5.74
26.02	3.18	119.4	32.1	155	23.5	36	2.18	137.9	4.42
11.05	1.67	55.2	16.02	65	19	48	1.76	132.1	17.85
14.55	2.21	78.4	21.32	84	16.4	60	1.52	145.9	20.85
2.71	2.64	71	21.9	205	14.3	67	1.32	130.5	7.88

10.05	3.52	105	30.3	37.4	30.2	29	2.8	130.3	12.71
5.67	2.28	83	25.12	32.4	22.1	41	2.05	136.5	22.03
7.40	1.17	47.2	14.62	18.4	31.8	27	2.94	135.9	6.36
1.51	1.38	46.2	14.22	44.4	42.1	20	3.9	139	6.23
2.40	2.91	102.4	27.4	70	50.5	16	4.68	136.5	6.51
3.67	4.75	163.4	43.8	141	37.9	22	3.51	137.2	3.68
4.19	2.66	99.4	26.5	72	28.5	31	2.64	138.7	4.18
4.29	5.05	162.4	44.3	127	33.2	24	3.04	139.2	4.3
1.43	2.7	65.2	19.52	134	14.8	69	1.37	144	7.33
7.13	1.74	66.4	17.72	43.4	36.7	23	3.4	131.5	32.96
6.54	3	117.4	30.2	212	29.7	29	2.75	129.1	6.34
4.50	5.61	187	53.6	77	21.4	41	1.97	139.4	7.85
3.70	3.43	110	29.9	67	23.8	38	2.2	133.7	7.25
5.51	3.27	114	31.7	84	21.1	44	1.95	130.9	2.29
3.04	3.83	133	36.8	28.4	21	45	1.94	137.2	2.51
20.72	2.65	84	23.62	73.4	15.2	66	1.33	134.5	9.73
4.35	4.59	152	43.1	123	26.1	34	2.42	136.5	8.46
8.71	2.67	95	27	70	20.6	46	1.91	133	6.91
4.06	3.4	104	29.5	119	17.2	58	1.59	135.2	4.02
2.11	1.79	61.2	18.62	23.2	28.5	31	2.64	140.2	5.73
3.32	2.29	74.2	21.52	48.1	28.8	31	2.67	142.8	3.84
1.03	2.95	108	30.5	64	19.6	46	1.71	139	5.39
27.92	1.94	77.4	20.32	125	48.8	17	4.52	118.5	41.21
9.67	2.11	71.2	20.62	41.4	16.1	64	1.49	137.6	15
11.33	1.82	52	16.7	260	20.4	44	1.78	126.5	7.11
3.45	3.85	137	37.7	131	25.5	33	2.24	135	8.61
3.94	3.07	112	32.2	74	20.3	47	1.88	137.1	7.98
5.39	4.06	143	39.2	64.4	11.9	99	1.1	137.1	10.51
4.35	4.17	134	37.3	70.4	66.1	11	5.85	133.3	3.94
19.20	4.15	143	42.8	34	29.2	28	2.56	137.2	5.22
17.33	2.19	86	24.52	264	15.8	65	1.46	133.4	7.88
22.77	1.58	50.2	14.82	108	21.8	43	2.02	136.6	14.29
10.85	1.99	81	23.3	41	24.6	35	2.16	133.2	13.97
8.84	3.32	127	35.5	27.4	32	27	2.96	140.8	6.44
5.84	1.62	57.1	15.32	130.6	19.3	47	1.69	147.9	21.05
3.81	4.71	149	41	81.4	56.9	14	5.27	140.4	9.85
15.81	4.08	128	37.1	124.4	17.4	57	1.61	128.6	10.08
2.78	2.16	47.2	15.52	27.4	19.5	49	1.81	140.2	13.95

4.13	2.88	104	30	45.4	12.9	82	1.19	131.6	2.44
3.35	3.87	131	38	148	22.7	37	2.1	134.9	5.5
8.02	2.98	108	31.9	55	21.1	39	1.85	127.5	8.24
3.85	4.59	143	40.5	98.4	26.3	31	2.44	139.6	2.79
4.34	3.24	101	29.3	86.4	14.7	67	1.36	128.8	11.45
2.43	2.02	60.2	19.02	87	14.7	67	1.36	141.7	7.57
1.88	3.59	120.9	35.14	67.8	15	64	1.31	137.1	5.25
3.37	4.8	146	42.2	106	20.9	40	1.83	130.7	20.78
8.80	2.43	81	23.72	51	35.6	22	3.3	130.5	13.94
1.10	3.06	92	26.2	34.4	21	41	1.94	142.5	1.61
33.49	1.99	54.2	18.02	97	26.4	31	2.44	137.6	5.18
11.38	3	92.4	25.02	43.4	29.6	27	2.74	125.3	3.11
17.75	1.84	66.2	20.82	30	51.5	14	4.77	137.5	4.06
1.62	2.32	81	23.8	41	33.7	22	2.96	135.9	7.02
8.87	2.35	66.2	21.32	71	27.2	33	2.52	134.5	4.3
1.75	3.5	110.4	29.3	201	14.4	73	1.33	125.3	25.06
3.34	4.26	152	42.9	69	19.4	50	1.8	134.2	8.8
3.54	4.12	126	36.3	74	16	63	1.48	132.4	13.99
33.31	3.62	133	38.7	90.4	23.9	38	2.21	133.5	8.16
2.03	3.86	136	37.8	46	19.1	50	1.77	134.7	3.59
7.57	2.84	90	26.6	193	13.7	78	1.27	144.4	22.42
5.09	3.77	127	36	30	22.7	41	2.1	136.5	13.73
0.88	3.03	70	23.2	24.2	13.5	78	1.17	133.3	11.29
7.89	2.72	81	23.9	96	17.5	56	1.62	141.4	11.49
3.05	1.49	45	13.7	75	22.8	41	2.11	138.4	9.55
12.35	3.85	99	29.6	127	14.3	74	1.34	122.7	19.16
3.02	3.21	74.2	24.52	80.4	18.6	48	1.72	137.6	6.82
3.41	1.29	49.2	14.02	52	43.9	18	3.72	120	11.24
2.41	1.34	50.2	14.75	61.6	17.2	55	1.48	145	22.49
1.24	3.31	105	30.1	109	42	18	3.89	139	3.45
0.36	3.8	93	28.7	273	24.4	34	2.26	144	4.71
5.22	2.61	94	27	95	23.7	35	2.19	133.9	7.3
6.50	6	199.1	56.91	24.4	19.6	45	1.81	136.4	14.24
0.76	3.27	122	34.8	29	15.3	65	1.32	138	5.87
5.56	2.9	103.4	27.3	77	26.7	32	2.28	118.7	3.97
7.86	3.38	103	30.4	244	11	108	0.96	135.5	15.13
4.05	3.47	137	38.4	49.4	21.9	39	2.03	130.7	4.36
4.10	1.37	57.2	16.22	86	19.4	41.4	1.66	128.9	4.44

30.86	2.1	72.2	20.82	49	21.4	35.5	1.83	129.2	35.98
10.19	1.38	39.2	12.92	37.4	19.8	40	1.81	151.1	15.84
6.20	3.05	98	27.3	57	19.8	39.8	1.59	129.9	13.93
7.82	4.29	155.4	41.5	73	15.3	60.2	1.32	119.2	9.24
7.91	1.88	68.4	18.62	70	18.4	45	1.58	116.3	30.26
5.25	4.08	124	34.9	159	15	64	1.31	135.8	8.7
2.03	2.49	76.2	22.82	170	14.8	66	1.37	141	12.32
5.90	3.41	75.2	23.8	47.6	26.4	25.1	2.1	130.9	8.91
5.31	5.03	162	48.4	106	27.4	24.1	2.34	132.6	3.84
2.62	3.43	125	36.5	61	26	32	2.41	148.4	14.28
10.55	2.18	56.2	17.22	60	14.9	66	1.38	126.2	36.45
4.20	1.12	34.2	10.32	8.4	20.1	39.2	1.72	131	21.88
3.00	4.06	98	30.4	30.4	17.1	54	1.58	143.6	2.95
2.65	2.34	74.1	21.51	107	13.5	80	1.25	136.6	18.38
8.38	2.23	86	24.7	57	30.4	29	2.81	132.3	4.4
24.80	2.44	91.2	26.62	30.2	24.2	28.9	1.93	126.4	36.35
1.94	3.7	128	36.5	56	20.6	37.7	1.77	136.4	3.28
9.26	2.43	90	26.92	28.2	39	20	3.5	132.3	6.84
10.91	2.49	71.2	21.32	264	14.7	67	1.36	143.5	5.12
8.18	1.65	65.2	19.32	20.4	20.1	39.2	1.72	136.4	8.07
18.22	2.72	96.4	26.1	138	22.2	38	2.06	128	10.17
5.05	1.87	76	22.22	163	18.6	44.3	1.6	129.2	4.45
1.75	2.53	82.2	24.52	51	18.3	45	0.48	130.2	6.58
0.95	4.19	137.5	38.93	143.9	32.4	17.4	2.77	138.5	2.58
2.74	2.77	102	28.6	200	16.4	62	1.52	137.6	5.2
2.05	3.58	119.5	35.45	86.4	20.5	37.1	1.76	137.9	9.37
3.63	3.75	124.4	33	101.4	20.8	37.1	1.78	125.8	7.47
8.31	1.82	79.2	22.52	111	15	62.1	1.29	137.1	6.87
1.70	3.25	102	30.5	83.4	18.4	45	1.58	141.7	3.3
3.89	4.35	142.4	37.8	72.4	51.9	16	4.81	140.6	2.93
3.99	2.23	71.2	20.12	94	16.3	59	1.42	138.6	8.05
4.49	1.99	53	17.4	55	27.7	32	2.56	142.2	9.13
2.10	2.3	75	22.26	48.8	19.5	45	1.65	130.1	6.22
2.89	2.34	84	25.8	57	21.8	38	1.91	140.5	5.55
2.15	4.42	137.7	40.63	121	19.3	48	1.66	141.8	4.91
15.33	3.13	113	32.6	57	22.3	33.3	1.92	137.9	3.73
5.53	2.69	105.7	31.4	16.7	42.1	19	2.58	139.8	11.85
3.80	4.89	164.8	47.3	87.3	17.6	52	1.49	139.8	4.4

4.94	3.08	82	25.72	57.4	14.4	66.2	1.24	139.2	6.76
1.85	4	120	36.3	45	14.3	67	1.32	140.2	5.16
4.44	2.31	80	22.32	56.4	13.6	81	1.26	139.7	18.13
4.71	4.59	141	38.7	113	25.1	33	2.32	138.1	2.71
3.14	3.52	120	33	42.4	25.3	27.3	2.16	140.9	4.03
8.67	2.48	94.2	28	12.4	23.7	29.8	1.89	137	10.14
2.22	2.3	99.2	26.5	48.2	20.2	38.6	1.62	128.9	5.26
6.17	3.63	128	35.6	98	17.4	49.2	1.49	129	18.19
12.72	4.53	138.4	37.7	207	27.3	24.2	2.33	127.9	2.06
6.86	1.42	35.2	12.22	62	15.1	62	1.4	138.3	18.36
8.66	3.63	84	25.42	54.4	20.4	43	1.88	137	5.42
11.34	3.41	123.4	33	27.4	22.9	31.9	1.96	122.1	25.69
8.58	3.14	104	29.6	145	15.8	57.2	1.36	128.6	11.78
2.19	1.67	60	17.9	61	14.4	71	1.25	141.2	6.49
3.00	3.2	96	25.8	61	17.8	46.9	1.44	134.3	2.86
8.77	2.79	104.4	31.26	109	16.1	54.9	1.31	138.5	4.9
4.88	1.66	53.8	16.56	68.1	41.3	12.7	3.22	124.5	33.43
9.59	2.49	93.3	27.35	64.2	17.9	46.5	1.44	116.9	22.87
4.83	3.89	118	32.3	52.3	14	74	1.07	136.6	10.18
2.03	3.4	113	33.8	70	15.4	59.7	1.32	134.9	6.17
1.91	3.29	112	30.9	42	16.9	57	1.45	140.5	2.48
15.00	3.45	118	35.2	39.2	19.6	40.4	1.58	128.5	4.57
5.03	3.38	91	28.6	32.4	17.7	54	1.29	141.7	8.1
15.21	2.89	110	30.8	79	25.3	27.3	2.16	136.4	11.48
5.42	3.63	120.6	35.3	101.4	39.4	13.7	3.08	131.7	9.98
20.38	2.54	107.5	30.03	79.2	14.9	68	1.12	120.4	13.07
4.33	2.14	81	23.02	91	25.8	25.4	2.21	126	6.28
4.64	3.34	89.5	27.37	60.8	22.2	40	1.54	133.1	2.54
3.91	3.74	117.4	30.8	52.4	35.3	21	3.1	131.3	6.56
3.05	1.64	65	18.4	79	25.5	33	2.36	137.7	7.24
1.09	2.65	94.2	27.1	91.4	16.3	47.2	0.31	135.7	12.14
5.02	3.32	125.4	32.4	57	21.4	35.5	1.83	133.1	11.54
8.13	2.25	80	20.9	88	17.6	40.8	1.42	140.5	4.88
2.36	2.31	83	24.32	73	25.3	26.2	2.16	134.1	13.03
14.10	2.99	102	28	148	23.2	40	2.15	131.6	8.4
2.43	3.15	106.2	31.8	41.2	19.8	39.8	1.59	131.7	3.98
24.33	3.7	100	28.4	178	14.2	66.6	1.16	128	23.15
2.08	3.09	118	34.3	61	19.1	42.1	1.54	136.7	5.56

13.26	4.05	136.6	38.84	84.4	28.1	31	1.86	122.4	8.85
3.31	3.35	63.1	20.89	40.7	14.7	64.1	1.26	137.4	9.08
2.13	2.99	67.2	22.12	287	14.1	72	1.31	133.8	7.51
4.19	3.21	95	28.7	172	18.7	48	1.73	137.1	6.91
6.49	1.49	62.2	17.89	45.7	54.4	8	4.2	125.8	12.67
2.22	3.95	107.2	34.8	75	23.6	30.3	1.88	134.5	4.51
3.92	2.8	111.4	30.2	76	17.9	47	1.54	130.7	13.75
13.31	2.1	46.2	15.82	55.4	22.4	38	2.07	141.7	15.49
2.53	4.1	129.3	36.2	52.8	26.6	24.1	2.27	131.7	3.78
5.50	2.92	89	26.4	23	14.7	68	1.36	129.5	6.5
11.34	2.74	100	28.5	268	28.7	30	2.66	130.7	2.77
1.49	1.47	47.2	14.82	93	22	34	1.88	135.4	9.09
3.43	4.17	128	35.2	182	30.6	28	2.83	136.5	3.53
19.36	2.91	90.4	24.42	47.4	15.9	56.7	1.37	133.8	35.93
2.79	3.54	123.4	32.9	107	45.4	17	4.2	134.3	3.51
6.30	3.4	117	32.46	75.4	29.7	20.1	2.54	128.1	6.1
1.09	3.91	135	38.4	135	16.9	51.2	1.45	133.4	4.21
1.87	2.9	98	28	86	16.6	53	1.43	139	4.5
3.78	1.54	60.2	17.02	53	30.2	24	2.34	125.1	13.77
5.00	3.99	123	35.3	115	20.8	36.7	1.67	136.1	5.61
13.85	1.14	48.2	13.45	42.3	29.2	21.8	2.49	131.6	18.52
2.64	2.65	87.6	26.25	29.2	23.5	30.7	2.01	133.2	10.52
4.85	3.98	131.4	34.2	107.4	29.8	27	2.76	128.8	4.24
2.84	2.5	104	28.7	67	32.7	25	2.88	126.7	8.97
3.00	2.04	53.5	17.38	45.6	15.3	60.2	1.32	138.8	7.67
3.47	2.2	74.3	21.98	61.3	25.3	30	2.3	131.1	6.48
3.38	3.72	114	33.7	40.2	27.4	23.7	2.17	136.7	2.92
4.63	1.54	64.2	19.12	178	15.7	57	1.27	134.7	9.81
2.53	2.82	97	27.3	35.4	28	22.1	2.39	127.4	7.62
4.89	1.76	54.6	15.92	138.6	13.4	74.2	1.15	141	11.12
7.30	2.92	98	27	16.4	27.1	30	2.51	133.4	6.98
1.18	3.62	85	28.1	54.4	16.1	55.4	1.38	141	6.19
5.63	1.45	47.2	13.62	54	23.1	35	2.02	144.6	25.24
10.52	1.6	50.2	14.22	64	21.9	39	2.03	143.1	7.66
1.42	3.46	84	26.6	59.4	15.1	64	1.4	139.8	6.34
9.04	1.67	65.2	18.62	26.4	41.7	19	2.8	132.4	7.2
2.47	3.02	105.1	31.51	41.6	18.3	44.8	1.57	143.2	11.23
11.89	2.5	88	26.1	24.7	16.2	55	1.39	140.4	4.86

1.71	3.17	105.2	30.2	51	20.2	38.4	1.62	143.2	3.74
3.44	2.6	96.7	26.95	11.8	24.8	28.1	1.98	126.3	4.73
5.75	3.52	118	33.6	29.2	14.9	62.1	1.21	139.7	7.53
4.21	4.57	104	32.7	67.4	16.3	54.5	1.4	135.1	5.25
26.29	1.92	78.5	22.57	25.1	27.2	24.3	2.16	126.5	5.77
2.95	3.5	113	32	81	23.3	33.1	2	134.3	2.67
1.85	3.52	123.4	32.3	54	16.4	53.8	1.41	127.4	7.29
1.31	3.55	101	33.96	48.8	15.2	57	1.4	144.9	3.9
8.63	2.86	112	31.8	54	23.5	30.5	2.01	130.2	25.6
5.66	4.53	134	39.1	79.4	21.8	39	2.02	133.1	5.08
1.06	2.74	97.6	28.91	150.6	11.2	104	1.04	135.7	3.91
1.95	4.52	158.4	42.8	58	15.1	66	1.32	127	16.56
0.50	2.91	101	28.8	149	18	54	1.67	139.6	2.3
1.27	4.08	130	35.2	59	30.2	20.3	2.39	137.2	1.77
14.70	2.12	76.9	22.24	75	25.8	33	2	127.3	6.57
2.01	2.95	107	30.8	65	22.5	40	1.93	137.3	4.03
1.92	4.93	155	43.5	98	34.7	24	3.21	144.7	2.45
2.34	3.04	109	30.6	76	27.1	31	2.51	136.8	6.01
2.94	4.73	139	41.3	96	35.1	16	2.76	135.7	2.45
0.81	3.47	124	35.7	88.4	15	62.1	1.29	140.3	5.35
9.43	2.82	101.2	28.4	159	20	39	1.61	138	4.47
0.66	2.72	107.4	29.1	50	23.3	39	2.16	136	11.37
7.46	2.08	84.4	21.32	56	21	44	1.94	113	22.91
7.43	2.82	87	25.32	41.4	14.1	68.5	1.21	135.9	11.18
3.14	3.48	122.4	35.74	61.2	14.5	68	1.34	142.6	4.31
6.36	3.43	107	28.7	93	26.8	18.4	2.18	133.3	3.85
5.54	1.76	46	15.5	51	15.6	63	1.36	143.7	9.35
1.74	3.96	141.7	40.59	120.1	12.4	88	0.97	137.7	4.12
6.66	3.44	108	31.6	123	13.4	74.2	1.15	132.2	24.78
1.35	3.18	115	34.4	63	18.9	49	1.65	138.6	3.83
21.96	4.55	130	38.7	94	14.6	64.8	1.26	134.7	13.29
4.04	1.58	64.2	18.22	35.2	30.5	26	2.82	135.4	15.7
3.54	3.07	114	33.5	42.4	23.6	30.4	2.02	128.2	7.85
1.93	3.02	107	30.6	56	19.6	45	1.81	141.6	4.34
2.25	2.79	92.2	27	80	15.3	59.4	1.24	134.8	10.5
1.56	3.19	112	31.8	42.4	23.5	30.7	2.01	137.1	4.61
4.43	0.95	31.6	9.19	46.2	22.2	40	1.69	139	23.03
4.33	2.2	62	18.7	13	25.5	33	2.36	130.3	7.1

7.55	1.53	51.2	15.52	49.4	18.5	44.7	1.59	142	6.06
4.76	2.8	110.4	28.8	96	15.7	57.8	1.35	112.1	6.45
4.76	2.8	110.4	28.8	96	15.7	57.8	1.35	112.1	6.45
1.46	3.18	110.5	33.17	131.5	14	72	1.21	137.7	17.01
3.18	1.88	68.2	20.12	109	14.6	61	1.34	138.5	14.69
7.12	4.16	143	39.8	51	18.7	48	1.73	141.1	7.02
3.38	3.41	120	34.9	88	14.6	64.8	1.26	144.8	6.49
1.83	4.05	146.6	41.72	32.1	16.6	52.7	1.43	141	2.51
3.81	4.41	139	40.1	51	10.2	128	0.94	142.3	5.35
3.35	3.12	118.4	32.1	87	23.9	33	2.09	132.6	4.88
2.96	3.15	115.2	33.74	122.9	15.6	66	1.21	144.7	3.3
4.90	2.3	78.2	23.52	36.4	18.5	49	1.71	139.8	8.53
7.50	3.77	117	33.2	211	16.9	59	1.56	137.9	8.26
2.75	2.7	87	25.5	69	15.7	62	1.36	136.9	7.66
3.67	3.27	105.2	31.8	86	19.3	41.4	1.66	129.3	16.36
6.75	2.88	107	29.9	24	20.7	42	1.92	135.4	4.63
4.18	2.72	108.2	29.8	23.2	20.9	36.6	1.68	130.5	7.66
0.92	4.85	156	45	213	12.2	90	1.13	149.7	3.99
5.63	2.53	88	25.12	131	23.6	35	2.19	139.5	3.16
2.88	1.58	54	17.3	33	18.1	49	1.68	151.3	34.54
3.63	2.89	95.4	25.82	70	17.2	57	1.59	129.7	10.6
2.36	4.51	144.6	43.09	72	13.7	76	1.19	143.6	4.45
5.25	2.7	98.9	29.06	78.8	22.9	31.9	1.96	133.7	4.61
8.70	2.78	65.2	21.12	110	15.7	62	1.45	125.5	4.49
4.57	3.03	107	30.2	99	21.6	40	2	130.5	8.59
2.70	2.71	106	29.7	74	15.3	63	1.42	134.7	6.47
2.68	2.91	97	27.4	47.4	23.6	30.4	2.02	129.3	17.11
5.59	3.09	102	28.5	49.4	14.5	69	1.34	132.2	16.19
6.00	2.65	102.4	27.4	55	22.4	33.1	1.92	127.9	13.74
4.30	2.22	64.2	19.72	144	16.4	57	1.52	145.5	9.86
7.14	3.4	84.9	26.77	54.2	17.3	49.6	1.49	130.4	10.32
13.16	2.81	74.2	21.72	91.4	20.9	42	1.94	138.1	30.26
14.50	4.3	128	35.8	195	28.7	22.4	2.45	134.8	3.15
3.30	3.01	94.6	27.8	94	13.5	76	1.25	136.9	9.67
6.16	4.37	134	38.5	35.4	15.2	63	1.41	136.7	4.85
3.25	2.31	75.2	21.12	65.4	27.3	23.1	2.33	132.4	2.58
7.13	2.14	58	19.1	51	16.5	58	1.53	131.5	20.66
3.88	3.44	126	32.2	55	24.6	28.1	1.96	132.6	3.01

7.45	2.43	84	24.92	68	13.1	76.9	1.13	141.1	9.14
1.46	1.72	42	13.7	60	14.2	71	1.31	135.7	5.1
4.81	3.31	115	33.5	75	13.1	76.9	1.13	133.6	5.39
12.49	2.96	111.8	32.09	140.7	22.2	33.1	1.78	132.1	9.89
6.78	3.19	115	33.7	149	13.2	85	1.22	142.4	10.34
11.68	1.64	58.2	17.32	71	19	42.1	1.63	137.6	7.46
30.18	2.95	95.7	29.36	30.7	23	31	1.74	155.2	10.51
2.65	2.81	98	28.7	46	22.6	36	1.98	141.7	5.7
12.00	1.23	44.2	13.52	62	22.3	40	1.58	128.8	25.02
2.60	3.1	102	28.2	49.4	15.6	58.4	1.34	134.5	10.47
13.87	2.65	96	29.1	20.4	32.1	21	2.74	135.7	12.12
11.00	2.27	77.2	23.32	47.2	21	36.3	1.68	135.9	11.78
1.79	2.69	96	27.9	81	22.2	37	1.94	139.4	4.48
2.90	3.39	104	31.3	38.4	13.3	70	1.23	133.9	13.46
7.00	1.75	65.2	17.82	24.4	24.1	35	2.23	127.6	8.84
0.78	4.7	148.7	41.92	122.3	13.6	73.3	1.17	139.2	3.08
11.28	1.93	48.1	15.7	94.3	16.7	52.5	1.43	141.6	16.38
1.86	2.33	92.4	22.82	88	18.9	47	1.74	132.7	4.12
5.58	1.92	76.7	21.9	22.8	39.7	20	3.37	128.4	18.04
2.25	1.79	63	18	31	21.2	35.6	1.7	140.4	10.66
3.53	4.78	169	46.8	43.4	16.9	59	1.56	141.1	5.66
1.65	5.09	129	39.3	85	15.3	6.2	1.32	137.6	1.49
1.82	1.9	56	18.3	71	17.2	49.5	1.39	135.8	3.61
2.01	3.53	129	37	72	15.5	62	1.44	138.5	9.19
2.95	2.36	64.2	20.22	43.4	15.6	61	1.44	141.8	14.27
2.24	3.08	107	30.74	36.6	13.2	75.2	1.08	136.9	7.66
1.05	3.29	119	34.1	44.4	14.3	69	1.32	134.9	10.19
2.81	3.35	81	27.1	59.4	16	61	1.38	143	8.86
3.78	4.4	141	39.6	137	29	31	2.69	135.1	4.1
8.47	2.63	76.2	23.42	27.4	19.7	44	1.82	142.3	15.8
3.38	2.13	63.3	18.28	75.1	14	74	1.21	140.7	6.14
16.50	2.65	96	26.3	59	18.5	44.7	1.59	127.7	20.15
0.73	3.48	126	35.3	17.4	15.4	61	1.43	143.1	2.79
4.94	3.42	91	28	72.4	17.3	53	1.6	143.8	5.99
6.31	3.25	85	25.72	86	13.3	77	1.23	130.3	6.87
5.37	2.16	52.9	16.54	32.5	15.7	61	1.45	145.7	6.7
5.63	2.47	94	26.4	47	28.8	22.3	2.28	133	6.55
4.15	3.86	127	35	67	13.6	75	1.26	146.8	8.46

1.36	2.87	101	27.8	49	26.6	24.8	2.11	139.7	2.82
10.94	2.75	83	23.42	292	16.1	55.6	1.38	120.2	27.4
2.11	3.32	127.8	37.11	37.5	15.7	63	1.37	143.6	3.38
19.66	2.02	45.2	15.92	129	18.3	51	1.58	134.3	12.61
2.06	3.26	89	26.2	61	15.2	67	1.41	139.7	11.06
2.93	3.1	110	31.3	52	13.6	72.5	1.17	142	3.25
4.72	3.92	101	33.1	95.4	14.4	57	1.2	140.3	5.43
2.02	4.08	132	36.8	112	32.8	25	2.79	134.8	5.47
4.68	2.92	104	28.9	20.4	15.2	60.8	1.31	138.9	16.44
2.18	2.6	72	21.5	160	15.7	63	1.36	137.5	4.57
4.17	3.22	113	32	16.2	16.1	55	1.25	135.8	2.55
1.57	2.74	80	23.9	132	18.4	47	1.58	130.2	8.48
3.63	2.02	57	16.6	76	13.6	71.7	1.11	135.6	12.31
28.67	3.1	122	32.2	147	19.4	41.1	1.56	135.9	3.17
5.00	3.35	112	33.9	58.4	15.8	57.2	1.36	127.3	5.53
8.56	1.93	50.2	16.22	60	15.8	57.2	1.36	136.9	12.85
2.76	3.88	140	39.2	90	13.3	80	1.15	137.2	2.81
2.54	3.01	89.5	26.08	36.9	14.2	70	1.24	145.9	4.32
1.62	2.65	56.2	19.72	47.4	20.7	42	1.92	141.6	9.02
5.41	2.11	66.7	19.84	87.7	13.5	76	1.25	140.3	15.88
2.63	1.97	55.2	17.52	69	17.5	47	1.6	138.4	6.25
7.82	2.55	98.4	24.92	58	17.5	57	1.62	117.6	17.63
5.44	2.57	90	26.5	216	14.5	74	1.34	135.1	19.37
3.47	1.71	45.2	14.26	84.2	17	51	1.46	136.5	13.9
1.05	3.33	97	27.2	72	17.6	48	1.36	129	3.81
2.31	2.22	92	26.8	59	17.4	48	1.6	138.3	6.23
4.85	2.95	113.4	30.2	46.4	14.6	69	1.35	132.6	1.74
2.57	3.51	96	29	35.4	15.7	61	1.45	136.5	3.51
17.11	3.72	70.2	24.72	128.4	15.2	63	1.41	131.2	7.39
2.62	2.88	106	30.3	46	15.4	64	1.34	140.2	6.47
0.99	3.45	110	31.9	106	10.4	117	0.96	127.1	14
2.36	3.32	86.5	25.11	81.4	17.7	54	1.29	137.2	5.34
11.31	1.94	63.2	18.92	187	15.5	62	1.44	131.6	39.4
4.98	1.85	56.2	15.82	33.4	16.4	60	1.52	141.3	13.6
3.09	3.49	118	33.3	147	15.4	63	1.43	133.1	10.51
1.63	2.47	91.8	25.97	147	15.8	62	1.29	142.1	5.08
2.77	3.16	102	29.9	34.4	39.2	22	3.63	142.3	3.46
7.76	2.59	106.4	28.6	58	19.4	41.4	1.66	137.2	11.37

5.84	2.87	73.2	24.42	72	17.5	55	1.62	134.5	4.9
5.87	1.73	45.2	13.82	80	15	69	1.39	133.8	17.11
3.47	2.74	95.9	26.84	75.4	37.1	21	3.44	139.9	4.26
3.49	3.68	133	38.1	48.4	18	55	1.67	133.3	6.36
3.29	3.14	105	30.5	111	16.4	61	1.52	140.7	21.39
1.18	1.45	49	14.8	28	15.2	66	1.33	132.9	17.94
4.95	3.91	119	34	103.4	16.7	60	1.55	135.2	4.53
5.88	4.08	148.4	40.3	99	15.5	59	1.33	120.6	10.15
8.38	3.02	87	26.1	67	14.1	77	1.31	142.6	9.8
2.27	3.04	93	28.4	61.4	17.2	50.1	1.48	132.6	7.95
2.58	2.96	113	32	49	16.7	58	1.44	144.3	4.64
5.65	2.7	105	29.6	34.4	27.1	33	2.51	137.6	13.84
3.50	3.82	94	29.6	43.4	27.3	30	2.53	134.1	4.45
7.39	3.32	110	31.6	46.4	16.8	54	1.47	138	4.42
2.06	1.86	59.2	17.72	77	13.8	75	1.2	140.8	3.32
11.75	4.31	126.9	37.34	54.4	13.1	82	1.14	145.2	8.21
1.73	3.41	112	33.2	46.4	18.8	50	1.62	134.7	4.12
3.79	3.7	110	31.7	89	16.4	54	1.41	135.5	6.34
1.29	2.21	69.4	21.15	144.9	13.5	76	1.18	133.9	3.12
4.33	3.11	94	28.4	50.4	18.4	47	1.58	143.7	10.41
5.30	2.25	65	20.2	69	18.2	52	1.57	142	15.23
5.79	4.6	145	41.2	194	29.3	21.7	2.5	137	3.32
6.92	2.48	93.4	25.22	76	17.6	56	1.63	137.2	10.69
11.90	2.09	82.6	23.68	50.9	22.5	32.8	1.93	137.1	18.09
1.86	2.15	79.8	24.06	27.7	31.7	26	2.9	140.5	4.14
6.05	2.95	107	30.8	32.4	18.6	43.6	1.6	133.7	2.09
2.44	3.29	115.8	32.94	81.6	16	62	1.19	133.3	16.61
2.91	2.46	80.1	23.92	92.1	12.6	88	1.17	145.1	13.86
2.43	2.03	81.2	23.82	52	21.3	37.6	1.83	131.3	5.25
5.34	2.26	65.2	19.42	50	14.8	67	1.37	138.9	8.27
1.69	3.48	122.4	33.2	30.4	21	45	1.94	125.4	4.46
1.63	2.67	94	27.9	68	15.2	68	1.41	141.2	5.36
24.65	4.57	159	45.6	63	25.3	36	2.34	134.7	7.77
4.71	1.98	83.2	23.62	123	19.6	33.3	1.58	142.2	13.3
3.63	3.31	116.4	30.8	95	13	87	1.2	127.3	5.74
1.35	4.32	150	43.1	45.4	17.1	54	1.58	139.5	4.57
5.70	4.62	137	37.7	39.4	13	84	1.2	140.6	8.86
2.54	2.4	86	26.6	71	16.3	54.5	1.4	142.4	2.86

2.96	2.21	85	23.42	78	20.1	47	1.86	134.5	6.08
1.24	4.07	128	36.3	51	15.1	60.6	1.23	137.8	5.15
4.35	3.23	99	29.1	71	15.7	61	1.45	143.2	5.36
1.97	3.37	97	29.2	95.4	16.2	54.9	1.39	138.6	5.7
3.86	4.46	144	41.1	200	16.2	58	1.5	139.7	17.62
15.08	1.98	62.2	19.52	59	17.5	41.2	1.41	138.3	13.48
2.85	2.26	73.6	21.43	15.3	16.5	61	1.28	142.1	8.44
3.01	3.1	103	29.1	71	14.1	68.5	1.21	134.1	11.29
1.40	3.53	124.5	36.35	68.9	15.9	58	1.39	138.8	4.19
6.04	3.43	115	34.4	129	11.6	99	1.07	137.4	29.81
4.32	2.91	109	32.34	46.9	16.8	51.3	1.36	134.7	9.74
4.67	3.81	107.2	35.2	47.2	13.6	77	1.04	138	9.6
7.74	3.1	101	30.3	199	16.3	58	1.51	146.2	7.73
2.31	2.94	58	20.7	81	14.3	72	1.25	135.7	5.25
3.85	3.57	120	34.6	68	17.2	58	1.59	135.3	8.4
5.38	2.22	72	21.7	97.7	15.5	64	1.35	146.3	14.61
2.28	4	139	39.7	26.4	25.1	26.5	2.15	127.8	3.76
2.54	3.86	123	35.2	77	25.8	30	2.26	135.1	4.15
2.01	3.73	138	38.2	89	16	59	1.48	142.3	3.75
1.73	2.83	68.2	21.02	380	12	92	1.11	139.9	10.81
0.99	5.39	171	51.29	122	15.2	60.8	1.31	136.6	7.71
33.55	2.31	84	24.82	25.4	21.2	36	1.82	136.6	10.83
1.46	3.07	108.9	31.96	26.1	15.9	56	1.29	143.2	2.94
1.36	2.76	103	26	22	16.9	58	1.31	136	5.21
18.55	2.42	80	23.92	79	20.3	38.6	1.74	135.4	9.47
0.99	2.35	94	26.16	48.9	20.3	41	1.78	136	6.55
2.97	3.03	108.2	31.4	103	17.5	48.2	1.5	133.8	7.87
2.79	2.64	94	25.82	34.4	13.6	81	1.26	133.2	14.42
1.82	2.79	120	34.6	80	21.8	34.3	1.87	137	11.62
2.54	5.13	150	42.6	110.4	24.3	35	2.13	139.2	4.91
0.97	3.27	109	32.6	253	14.3	67	1.23	145.7	5.03
2.91	2.85	111	32.7	50	18	46.6	1.55	134.2	6.99
2.21	3.63	83	27.9	60.4	24	35	2.22	144.8	5.3
4.44	2.99	75.2	22.82	258	12.4	89	1.15	124.6	4.1
2.88	2.49	92.2	26.2	18.2	20.2	38.6	1.62	136.3	4.3
3.25	3.28	113	33.1	114	18.7	43.9	1.6	139	8.06
0.54	4.3	148.6	43.85	78.9	16	58	1.4	144.8	3.64
3.01	4.83	129	38.4	205	12.1	91	1.12	139.5	10.59

4.88	1.86	45.2	15.22	76.4	17.5	52	1.62	141.1	5.46
10.89	5.14	165.4	43.7	146	26.2	25.8	2.24	123.6	9.28
6.21	4.45	154	44	111	13.3	75.1	1.15	133.2	4.47
1.45	2.95	107	30.4	48	17.7	53	1.55	133.2	6.24
3.06	2.89	102.7	29.93	41.7	22.9	35	2.01	143.2	2.72
1.65	3	102	29.8	126	13	81	1.13	138.4	5.94
4.99	2.98	99	27.1	155	16	62	1.48	121.9	4.2
1.51	3.8	129	37	33	12.8	78	1.19	142.1	4.42
14.69	3.42	99	29.4	30.4	19	42.8	1.63	131.5	14.43
2.71	3.29	112	32.1	62	15.7	61	1.45	142	4.48
2.29	5.63	185.1	52.51	116	14.4	69	1.33	134.8	4.68
2.75	3.28	106	30.3	153	15.9	60	1.47	141.3	9.19
4.30	2.5	66.2	21.32	123	14.8	67	1.37	134.8	7.12
1.32	3.12	97	29.1	36	20.8	43	1.82	140.5	4.14
4.59	3.36	110	32.1	49	17.8	51	1.64	135.9	5.49
4.04	2.55	62.2	20.52	110	16	58	1.48	144.8	9.36
1.49	3.49	122	34.9	39	25.2	31	2.21	141.4	3.36
3.96	2.45	73.2	22.32	59	20.7	37.4	1.77	135.6	3.24
4.90	1.61	49.2	15.02	62	14.4	68	1.25	141	8.13
7.87	2.94	103	28.7	42.4	37.1	21	3.44	127.8	8.81
2.19	3.68	123	35.5	39.4	17	50.7	1.46	132.2	7.5
6.22	3.34	85	26.6	105	14.6	64.8	1.26	137.4	3.3
3.18	3.39	100.4	29.46	43.3	16	56	1.24	141.7	6.11
3.00	3.33	108.2	31.9	109	17.5	48.2	1.41	142	3.84
2.06	4.38	155.4	42.2	133	13.1	80	1.21	142.2	6.34
6.23	2.16	42.2	14.52	185	16.7	52.5	1.43	140.3	8.7
4.18	3.02	112	32.2	48.4	17.5	48.7	1.5	138.5	8.37
3.52	1.2	103	28.3	133	15	61.2	1.22	135.9	7.66
3.24	3.3	121.4	32.1	112	12.6	87	1.17	130.4	12.97
4.40	2.53	106.2	31	89	15.9	57	1.23	143.1	2.61
2.54	5.18	156	43	28.4	15.4	63	1.43	137.8	5.78
1.55	3.69	97	30.3	30.4	16.5	53.5	1.42	141.2	3.72
4.69	3.31	61.2	22.22	137.4	16	64	1.48	135.6	3.16
5.40	2.53	91.2	25.82	38.2	17.6	47.8	1.42	122.3	9.84
8.75	2.62	88	25.12	58	29.6	30	2.74	142.7	7.91
2.96	2.19	65.2	18.92	82	17.1	59	1.58	140.4	4.57
13.22	2.91	98	29	59	20.1	39.2	1.72	142.5	15.34
3.70	3.21	101	29.2	54	13.2	76	1.14	146.7	6.45

1.81	3.52	129	39.17	61.7	18.9	47	1.75	137.2	4.2
5.61	2.46	83.6	24.09	159.2	18.2	45.8	1.56	131.9	11.32
12.70	2.79	100	29.3	40.4	21.7	34.7	1.86	124.7	13.39
5.63	2.11	65.2	19.62	68	15.9	65	1.47	139.5	13.98
4.50	2.39	86	24.62	53.4	18	46	1.55	137.9	7.88
7.67	3.42	119	35.3	59	15.8	57.2	1.36	140.5	3.66
3.59	4.12	138.6	41.2	61	38.1	14.3	3.25	131.7	11.69
1.69	2.7	111.4	30.1	80	19.3	41.8	1.66	138.1	6.52
5.50	2.39	73	21.5	82	13.6	77	1.18	139.1	4.73
4.53	3.29	86.7	26.93	39.7	16.2	58	1.5	138.8	8.36
5.50	2.85	104.2	30.4	50	16.2	62	1.26	138.7	6.57
6.93	0.99	30.2	9.02	65	17.6	54	1.54	136.5	16.18
31.93	4.11	95	30.7	42	15.2	63	1.33	133.2	8.66
7.44	2.59	98	27.5	28.4	20.8	45	1.93	132.6	10.24
4.20	3.09	108	31.3	52	16.4	54	1.41	138.9	6.02
8.44	2.13	58.2	18.32	108	15.4	68	1.43	145.9	9.75
6.66	2.24	72.2	22.02	60	27.5	29	2.55	130.2	7.07
1.76	3.09	117	32.4	83	16	56.1	1.38	142.6	7.27
46.86	1.92	65.2	22.22	51	26.9	27	2.3	128	9.98
4.11	2.08	79	21	79	20.2	38.4	1.62	137.1	15.38
6.71	2.78	110.4	30	42.4	23.3	39	2.16	141.3	8.6
3.09	4.36	138	42.1	46.4	16.5	53.5	1.42	141	5.81
3.30	3.3	110	32	95	15.6	58.4	1.34	136.4	11.35
9.56	2.27	77	20.1	62	20.9	36.6	1.68	125.9	15.76
3.80	3.66	128	36.9	43.4	15.4	59.7	1.32	139.6	6.61
5.76	3.04	102	29.9	208	11.7	102	1.08	137.4	13.99
5.68	1.87	68	19.9	93	16.5	58	1.53	143.3	15.27
4.45	2.34	72.2	22.92	90	14.1	74	1.31	134.6	15.04
1.74	4.75	161.4	43.3	182	13.5	78	1.25	138.7	1.04
3.53	4.74	152	44.3	120	12.2	95	1.13	140.4	6.53
3.49	2.36	74.2	22.42	74	19.7	49	1.82	141.4	10.82
4.26	2.69	107.3	31.73	43.2	18.2	47	1.41	135.9	4.49
2.88	3.22	114	33	47.4	19.8	44	1.83	144.1	4.94
6.13	2.71	65.2	21.42	125	14.6	64.8	1.26	133.4	10.93
3.96	3.22	112	32.6	62	20.6	37.7	1.77	136.8	7.94
3.78	2.28	64.2	19.22	79	18.2	54	1.69	139.7	12.42
6.89	3.47	119	33.4	76	22.3	42	2.06	131.5	25.14
5.47	2.82	63.8	20.47	54.1	13.4	73	1.24	145.5	7.81

6.70	2.67	79.2	24.42	62	15	62.1	1.29	136.7	18.82
1.22	3.92	136	40.4	107	16	56.1	1.38	141.8	3.94
3.14	3.05	110	32	57.4	25.1	34	2.2	139.6	3.15
2.66	4.5	148	42.2	100	13.1	86	1.21	143	3.24
2.40	2.1	84	23.22	106	20.2	46	1.87	144.4	1.59
4.67	2.33	60	17.7	86	18.4	44.5	1.48	138.6	12.92
3.91	3.44	100.6	29.73	39.8	16.6	58	1.45	139.4	8.99
9.38	3.8	126	37.7	97	11.6	99	1.01	131.8	5.56
31.96	3.59	116	34.4	24	16.8	56	1.56	137.2	10.42
5.83	2.25	63.2	19.42	140	14.2	76	1.31	141.7	11.13
4.88	3.26	98	28.9	254	14.1	68.5	1.21	136.9	12.09
2.23	4.52	134	40.8	98	21	45	1.94	140.9	2.87
1.93	4.56	134.1	40.6	113.5	19.5	47	1.7	141.4	2.13
4.86	3.52	127	36.2	144	15.2	60.2	1.23	136.9	6.63
3.20	3.67	116	32.9	241	12.4	82	1.15	135.9	8.16
4.18	3.35	109	31.1	77	17.1	56	1.25	129.3	12.5
3.60	4.51	145.4	37.7	209	32.7	26	3.03	135.7	2.38
3.77	4	126	39	62	14	78	1.3	142.9	6.42
4.93	2.25	77.2	23.02	27.4	18.7	43.9	1.6	132.6	14.67
6.53	2.47	108.2	32.45	49.8	20.3	38.6	1.74	129.8	6.93
9.39	2.83	94	29.4	47.2	20.6	44	1.8	149.1	5.94
1.61	3.92	134	38	35	17.1	54	1.58	141.9	4.18
3.61	2.76	98	28.2	48.2	19.4	43	1.66	139.2	3.71
18.95	4.03	154	43.2	13.4	15.5	64	1.35	133.3	5.12
2.25	3.86	135	39.1	53	14.9	62.8	1.28	140.5	6.8
9.11	2.68	88	24.92	186	16.2	61	1.5	143.5	12.13
5.65	1.27	32.6	10.24	30.8	15.8	63	1.18	138.5	11.78
10.49	2.86	110.4	29.9	21.4	33.9	25	3.14	132.7	11.08
4.08	3.33	112	31.1	63	14.5	74	1.34	135.3	8.96
2.17	2.96	105	29.2	23	17.8	47	1.31	140.2	9.7
11.31	3.32	109	33	187	17.3	49.6	1.49	148.9	16.33
2.98	3.21	125.2	36.8	116.3	13.6	71.2	1.11	133.6	13.41
5.76	1.16	27.2	9.82	42.4	16.3	59	1.42	141.6	15.4
13.64	3.37	109	30.4	45.4	20.3	47	1.88	139.1	4.97
24.53	4.17	129	37.8	45.4	47.9	17	4.44	135.4	6.11
7.02	2.96	90	27.1	66	13.2	76	1.14	132.2	16.55
1.22	4.23	105	32.1	248	11.4	106	1.06	139.2	8.29
6.58	3.2	79.3	25.61	40.9	13.6	72.5	1.17	140.7	8.91

7.58	1.85	62.2	17.52	77	19.9	45	1.85	135.6	12.23
4.67	3.65	129.4	34	59	23	39	2.13	139.6	4.67
6.17	3.05	90	27	26.4	18.7	51	1.73	144.5	7.98
15.90	3.55	131.8	39.69	53.6	21.1	37	1.93	140.8	9.19
3.58	2.07	68.2	19.32	98	16.4	60	1.52	140.2	18.84
1.77	3.73	135	39.3	85	14.2	68	1.31	135.3	3.86
6.72	3.17	110	32	71	19	42.1	1.63	136.7	27.42
4.71	3.37	111	31.3	53	15.5	59	1.33	136.9	6.15
4.65	3.18	119.4	32.4	44.4	13.3	80	1.23	141.2	7.07
2.09	4.28	108	34.3	36.4	16.9	51.2	1.45	141.7	5.93
2.67	3.38	76.2	23.82	77.4	15.3	60.2	1.32	136.5	8.7
11.52	3.46	71.2	23.42	229.4	14	74	1.3	137.6	32.79
15.62	2.72	91	25.82	137	16.1	55.6	1.38	140.2	2.82
1.62	4.68	160.4	43.7	71	14.6	64.8	1.26	136.5	3.07
2.11	3.04	105.6	29.66	38.8	37.7	22	2.36	145	5.9
11.05	3.13	117.4	30.9	168	13.2	81	1.22	132.4	16.19
8.72	3.48	121	37.2	186	21.3	37	1.95	131.7	4.36
10.27	1.35	53.2	15.92	61	32	18.9	2.73	134.8	10.88
1.31	4.14	132.7	39.52	107.2	16.9	50	1.55	141.6	5.09
9.00	2.11	71.2	20.82	107	15.8	56.6	1.28	148	16.59
5.88	2.35	68.2	21.82	123	13.1	75.5	1.07	140.1	10.49
2.87	2.2	50.2	16.02	76.4	21.6	42	2	136.2	13.27
1.45	2.49	91.9	26.78	21.2	17.4	49.2	1.49	136	5.03
9.93	3.79	89	29.3	76.4	15.9	56.7	1.37	133.4	7.34
4.58	2.52	81	23.22	63	15.3	66	1.42	140.9	16.17
7.78	2.96	98	28.6	59	14	74	1.07	129	5.91
5.19	2.16	78.2	21.82	29.4	14.1	68.5	1.21	139.7	10.49
2.37	3.42	112	34.9	29.4	16.4	54	1.41	135.1	7.19
5.80	2.27	82	23.42	100.4	15.7	62	1.45	140.4	27.48
8.57	1.8	37.2	13.04	185.4	18.2	45.4	1.47	135.5	10.52
3.77	2.08	64.2	19.42	192	17.1	50.6	1.47	129.1	8.46
3.14	2.16	82	22.7	16	20.6	44	1.91	125.5	6.58
3.24	2.93	98	28	77	14.5	65.5	1.25	142.5	13.95
14.43	2.79	97	27.4	20.2	18.9	47	1.74	136.7	11.5
2.82	4.43	107	32.9	17.4	17.9	47	1.54	135.7	7.16
3.99	4.58	134	39.1	26.4	14.6	70	1.35	140.2	5.75
6.67	3.04	93	27.2	126	14.1	68.5	1.21	133.9	20.48
7.59	3.57	125.7	37.66	93.5	12.4	78	1.14	135.1	7.86

5.20	3.52	121	34	49.2	17.5	49	1.36	127.1	8.9
4.40	3.29	66	22.1	53	16.1	54.9	1.31	139.8	6.78
11.55	4.46	122	36.1	53.4	18.7	47	1.73	136	5.81
2.69	3.71	84	27	37.4	16.5	53.5	1.42	135.3	3.73
4.40	1.15	44.7	12.47	79.7	19.4	40.9	1.56	131.4	13.36
6.92	1.53	57.2	16.42	59	50.3	15	4.66	133.6	8.09
4.77	3.12	113	31.1	34.4	16.5	53.5	1.42	139.3	3.81
11.59	4.51	150	41.6	95	24.9	28	2.13	134.5	2.99
2.94	1.54	38.2	12.62	229	14.9	67	1.38	144.2	9.76
3.93	1.94	60.2	17.42	130	19.8	40.1	1.7	142.9	10.59
2.75	3.01	99	28.4	25	24.4	34	2.26	141.4	6.25
7.00	3.1	100.2	29.7	34.2	19.4	40.9	1.56	138.5	12.39
1.08	3.38	119	33.3	71	19	42.3	1.53	139.7	4.44
2.92	2.63	102	28.2	78	16.2	55	1.39	135.5	8.46
3.83	3.24	78.2	27	71	13.2	75	1.22	138.4	5.94
1.50	4.19	146	41.6	25.2	13.6	71.2	1.11	140	6.41
6.80	4.68	145	39.9	323.4	18.4	51	1.33	133.5	5.95
10.10	2.62	71.2	21.74	31.4	14.6	65	1.35	130.6	8.24
2.05	3.19	107	32.5	56	17.4	52	1.61	142.5	4.26
3.20	3.32	126.1	36.59	101.7	16.5	53.5	1.42	146.5	5.19
1.30	3.55	71	21.6	92	18	39.1	1.45	135.1	4.58
1.84	3.01	96	28.8	74	14.5	66	1.34	136.1	5.93
6.53	2.49	99.4	26.3	46.4	17.9	47	1.54	120.8	18.4
22.17	3.23	112	32.1	103	19.7	40.5	1.69	129	17.02
2.36	3.71	133	38	45.4	16.1	55.6	1.38	134.7	7.92
1.91	4.26	141.3	41.94	147.4	22	35	2.01	141.9	3.31
4.35	1.96	47	15.08	71.3	13.6	72.5	1.17	142.3	7.31
3.95	1.77	56.2	16.92	146	20.3	38.6	1.74	141.2	14.33
2.90	3.34	118	33	90	18.2	45.4	1.47	135.5	12.4
13.63	3.91	125	34.4	52.4	15	62.1	1.29	129.2	5.29
8.96	2.17	68.2	20.72	92	22.1	39	2.05	138.1	5.95
5.69	3.52	84	27.2	47.4	16	56.1	1.38	139.7	5.81
2.82	3.69	126	36.8	82	16.5	53.2	1.42	134.8	4.6
3.80	3.03	97	27.5	69	13.2	75	1.22	139.6	9.39
3.03	2.43	81	23.12	24.4	16.7	52.5	1.43	124.6	8.82
8.28	2.8	89	26.7	49.4	13.6	72.5	1.17	140.2	13.84
1.70	2.68	105	30.4	54	13	81	1.12	134.1	7.81
0.99	4.11	148	36	123	13.9	70	1.2	142.3	4.24

2.45	2.15	69.2	20.02	89	16.3	54.5	1.4	132.2	10.54
7.94	2.7	89	25.22	47.4	16.1	55.6	1.38	134.7	10.46
22.52	2.91	85	24.72	44.4	15	62.1	1.29	136.8	13.01
11.51	2.61	85	24.42	341	19.4	45	1.8	131.6	3.68
7.03	2.44	60.2	19.22	124	14.6	64.8	1.26	141.2	17.24
6.37	3.63	111	31.8	87.4	21.9	34.2	1.88	139.5	7.18
5.58	2.5	54.2	18.22	88	16.4	54	1.41	136.9	22.66
2.73	1.86	63.2	17.42	98	15.1	62	1.4	136.3	12.72
8.33	1.58	50.2	16.22	187	20.4	38.3	1.75	136	24.89
2.89	3.43	125	34.4	48.4	13.9	70	1.2	137.4	6.13
1.15	4.19	138	39.3	69	16.3	54.3	1.4	135.5	4.24
4.57	3.21	101.5	29.9	28.6	18.4	47	1.58	138	7.81
10.43	4.42	121	40.9	119	16	56.1	1.38	133.3	1.78
6.47	1.69	74.2	21.02	37.4	21.4	35.5	1.83	130.4	10.24
8.74	4.28	132	36.5	32.4	14.4	66.2	1.24	123.6	11.66
1.21	3.76	129	38.5	59	12.6	76	1.16	143.6	6.11
11.88	2.14	70.2	20.12	49.4	28.5	30	2.41	137.6	14.33
6.70	1.41	48.2	14.22	86	21.3	43	1.49	135.3	10.03
1.52	4	139.4	38	161	17.5	55	1.28	135.4	16.2
3.76	2.15	71.2	20.72	157	14.9	62.8	1.28	140.9	18.38
4.00	3.19	93.2	28.5	25.4	18.2	46	1.41	137.3	3.17
2.85	3.71	80	26.2	113.4	17.3	49.6	1.49	138.6	9.04
2.54	3.74	99	30.7	30.4	19	42.8	1.63	142.2	3.94
1.81	4.07	139.2	41.54	87.4	14.1	64	1.3	142.3	2.93
2.58	4.47	153	43.5	121	15.5	59	1.33	136.8	6.29
2.77	3.14	107	29.4	40.4	17.9	47	1.54	134.4	7.53
3.31	3.48	121	36	71	25.3	27.3	2.16	141.8	4.13
4.24	3.17	100	29	77	16.5	53.5	1.42	145.7	10.59
9.84	1.73	60.2	18.42	86.4	19.8	40	1.81	146.7	19.68
1.80	3.19	110	29.9	84	25	30	1.94	138.2	6.18
2.69	2.7	93.1	27.98	76.7	12.7	75	1.17	141.1	5.83
4.33	3.45	119	32.5	76	16.2	54.3	1.31	134.6	12.38
1.51	3.34	86.4	27.49	75.2	11.6	86	1.07	137.6	9.91
3.40	2.56	61.2	19.42	210	13	77.8	1.12	137.5	7.3
4.59	4.44	133	38.9	121	10.9	96	1.01	139.8	6.07
11.20	4.2	145	40.6	96	16.7	52.5	1.43	136.2	5.15
4.60	2.85	89	26	91	16.2	54.9	1.39	130.4	19.56
1.59	4.03	128.1	39.12	178.7	12	82	1.11	137.8	4.18

4.75	3.48	130	37.5	39.4	18.3	45.4	1.57	137.8	6.09
17.53	2.33	77.2	22.82	95	17	51	1.46	135.4	15.57
6.55	3.86	120	33.3	76	23.8	30	2.04	137.6	5.22
2.44	3.65	118	35.3	66	18.5	44.7	1.59	139.1	7.18
1.80	2.48	84	24.02	83	20.7	36.5	1.77	137.5	5.84
2.09	3.9	132.4	35.6	28.4	20	39.5	1.72	136.2	8.89
1.87	3.55	134	38.3	219	12.1	87.1	1.04	133.5	3.76
8.33	2.35	65.2	20.82	98	48.7	14	4.35	135.5	8.33
2.10	4.05	126	36.3	53	14.1	68.5	1.21	142.8	6.64
2.10	4.26	127	37	127	13.6	71.3	1.17	139.6	6.47
1.10	3.37	127.7	35.57	76	14.6	67	1.25	141.7	4.95
1.13	3.56	125.8	35.98	51.4	15.6	57	1.21	145.5	4.32
6.25	4.14	133	37.9	72.4	29.2	21.8	2.49	137.8	2.94
19.00	1.68	52.2	15.72	42.4	14.8	63.5	1.27	138.3	12.79
9.17	3.12	118	33.4	114	18.9	42.8	1.52	134.5	5.64
6.74	4.03	124.4	36.6	142	24.4	36	1.67	134.9	3.58
3.63	2.94	70	21.1	86	15.3	59.6	1.24	136	12.71
5.90	2.45	65.2	20.42	249	14	68.1	1.14	135.4	8.23
14.83	2.86	89	26.3	46.4	19.6	40.8	1.68	144.5	5.88
5.51	3.2	91	29.1	32.4	12.9	73	1.19	136.5	3.88
13.31	4.93	155	44.6	207	40.4	13.1	3.44	138	2.77
1.25	4.13	126.3	38.22	93.3	13.7	67	1.26	139.5	3.52
3.31	3.27	121	34.6	45.4	15.7	57.8	1.35	137.4	7.96
2.32	4.13	141	39.1	48.4	17.3	49.6	1.49	131.2	5.88
3.36	4.02	139	40.5	48.4	19.4	41.4	1.66	142.7	2.78
0.77	3.81	125	35.2	126.4	15	62.1	1.29	142.3	5.15
14.43	3.73	121	36.1	94	12.7	80.7	1.09	131.7	6.89
9.06	3.72	120	34.2	54	15.5	56	1.42	133.5	7.39
7.16	3.39	114	33.2	63	13.8	66	1.27	138.6	16.63
2.14	4.04	137	37.8	60.4	32.3	22	2.93	133.5	2.43
5.59	2.85	70.2	23.62	47.4	16	56.1	1.38	133.9	8.35
0.88	3.53	93	28.28	27.4	18.4	45	1.58	136.2	7.58
6.70	3.17	111	33	264	11	85	0.97	137.7	3.3
3.70	3.73	123	34.3	108	25.8	26.5	2.21	140.3	3.42
2.45	4.12	102	32.5	62.4	18.6	45.7	1.6	144.5	5.84
1.44	3.38	113.4	30.6	80	17.6	49.4	1.51	138.3	4.41
2.28	3.27	118	34.1	122	16.4	53.3	1.33	137.4	3.66
2.10	3.98	126	36.2	44.4	15.9	56.7	1.37	135.5	2.96

11.68	3.44	84	25.72	220	13.6	77	1.17	132.5	14.31
5.44	4.4	143	41.2	398	15	61.2	1.22	134.4	3.21
2.29	2.5	52.2	17.72	106	13.2	76	1.14	139.8	4.2
1.19	2.85	92	26.4	111	19.9	39.8	1.71	138.4	6.35
8.51	2.1	68.2	21.22	24.4	17	51	1.46	140.5	10.36
4.13	2.57	77.2	23.54	111.2	14.1	68.5	1.21	136.7	11.4
2.58	1.27	42.2	12.42	44.4	22.1	41	1.88	134.4	8.8
4.66	3.9	127	35.5	55	14	69.3	1.21	140.6	6.24
2.44	3.09	101.3	30.57	19.6	19.4	46	1.64	140.6	5.97
13.39	3.96	125	36	111	19.4	41.4	1.66	133.4	3.9
3.02	1.65	58.2	16.82	29.4	22.9	39	1.95	146.9	5.71
1.05	3.75	126.4	37.81	106.9	13.2	81	1.02	143.1	10.91
17.51	3.26	87	25.22	103	15.4	59.6	1.32	126.7	24.55
9.58	3.15	108.8	32.33	82.2	15	61	1.16	127.7	2.69
3.99	2.01	51.5	16.27	101.9	24	37	1.64	122.5	12.54
6.18	2.99	102	30.5	69	15.1	61.5	1.3	137.3	7.08
3.88	3.32	132.7	38	59.5	13	82	1.01	131.4	8.24
41.10	1.25	31	9.99	63.5	23	31.7	1.97	135.6	11.87
3.62	4.8	149	41.9	96	14.2	67.7	1.22	142.8	3.86
5.65	2.42	44.1	14.74	46.2	17.2	56	1.26	139.7	12.31
1.96	3.81	119	34.9	99	16.4	54	1.41	137.4	4.2
0.76	3.08	100	29.8	225	14	68.5	1.14	145.1	9.04
3.86	1.82	58.2	17.52	136	15.8	57.2	1.36	128.1	12.25
2.05	2.58	83.2	24.42	73	17.3	49.1	1.4	139.5	14.54
0.56	4.21	110	35	359	15.9	56.6	1.37	145.3	4.26
7.10	3.64	121	33.2	173	35.6	15.9	3.04	134.7	6.36
3.50	3.4	111	30	51	15.5	58.3	1.26	129.6	10.06
1.24	2.93	98	28.8	50	17.2	50.1	1.48	143	4.25
2.67	4.58	142	41.4	69	14.6	64.8	1.26	139.1	2.6
2.17	2.87	92.2	27.7	102	14	79	1.07	141.3	13.1
5.52	5.01	113	36.3	51.4	16.4	53.8	1.41	141.4	6.13
2.64	4.34	133	37.8	109	21	35.7	1.8	135.2	5.03
2.15	2.26	83.1	24.93	95.5	14.4	65.5	1.17	135.8	11.16
2.33	1.79	52.2	16.02	221	20.4	38	1.64	135	14.83
6.92	4.03	135	38.4	49.4	18.4	47	1.58	144.7	4.22
5.00	3.24	98	26.3	78	19.8	32.6	1.6	137.9	5.12
3.09	4.13	141	40.2	61	16	56.1	1.38	147.7	4.83
1.16	3.92	129	40.19	167.5	11	105	0.95	123.1	22.61

6.01	1.24	34.2	11.12	106	22.5	32.8	1.93	127.1	13.67
5.78	2.26	85.2	23.92	137	27.8	17.2	2.26	121.3	5.68
0.43	2.94	108.2	30.6	75	15.6	57.6	1.27	136.4	7.84
2.88	2.55	84	24.82	95	16.4	54	1.41	139	8.86
1.69	2.52	79.3	24.42	54.8	22.6	38	1.97	143.5	11.84
4.49	3.07	78.2	24.82	44.2	14.5	65.1	1.25	142.7	8.05
4.20	2.58	71.2	22.12	110	15.2	60.8	1.31	141	9.7
5.90	4.32	135	37.2	182	31.3	19.5	2.67	130.2	3.84
3.05	2.64	98.4	26.8	73	13.1	76.9	1.13	133.7	6.41
5.73	1.86	59.2	16.52	75	24.2	28.2	2.07	128.2	24.88
3.03	2.8	81	24.32	58	14.4	66.7	1.24	138.4	7.15
2.54	1.06	34.2	9.62	115	16.9	51.5	1.45	133.9	12.6
7.35	1.99	53.9	16.52	65.5	17.8	47.5	1.53	138	11.59
4.86	2.94	94.2	26.88	75.2	14.8	63.5	1.27	132.2	12.49
13.43	3.37	116	33.1	225	18	47.8	1.55	138.9	7.6
2.64	4.79	161	46	119	16.5	54.1	1.42	137.5	6.16
2.66	3.58	124.6	35.73	53.8	15.8	63	1.18	143.3	3.06
4.04	3.13	102	30.9	185	14.6	64.8	1.26	139.7	15.01
7.83	2.96	107.4	29.1	85	13	82	1.01	134.2	10.1
2.95	2.09	68.3	19.96	19	19	49	1.38	145	7.78
6.13	4.44	112	35.5	52	15	61.4	1.22	134.9	15.03
1.72	2.74	96.8	29.19	47	19.5	48	1.39	141.1	5.17
0.87	3.45	121.8	35.56	74.1	13.5	78	1.04	143.9	5.83
1.68	3.77	128	35.6	99	16	56.1	1.38	140.6	9.68
17.40	4.41	142	40.7	205	33.7	16.3	2.87	139.2	2.2
3.99	4.31	121	37.1	75	13.3	76.1	1.15	136.5	7.79
1.02	3.22	123	35.2	137	11.2	94	0.97	143.9	4.29
7.11	2.02	71.6	21.38	136.8	17.5	48.3	1.5	134.4	8.57
4.29	3.22	96	29.4	37.4	16.6	52.7	1.43	137.2	9.87
5.52	4.22	98	31	113.4	14.2	68.2	1.22	136.9	6.57
9.03	4	135	39.1	59	15.7	57.8	1.35	130	4.12
2.81	3.49	141	10.02	103.9	17.1	115	0.85	140.5	8.29
6.17	3.23	108	31.3	39.2	17.7	49	1.52	130	11.54
4.56	3.04	106.2	29.7	66	19.3	41.4	1.55	126.2	8.6
1.66	2.23	69.2	20.28	66.9	23	31.6	1.84	129.9	5.24
0.71	3.38	112	32.4	161	12.6	94	0.98	142.1	5.69
4.94	4.93	159	44.3	112	24	28.6	2.05	137.2	4.08
3.40	4.29	137	39.3	45.2	18.4	44.6	1.48	141.5	3.36

1.67	3.62	142	38	63	16.1	56.1	1.38	122.1	5.04
3.91	2.28	81.4	22.64	9.4	20.4	40	1.75	136.5	8.32
3.98	3.85	125	37.4	59.4	14.5	71	1.1	142.4	8.58
6.81	3.09	93	28.2	71.4	36.8	17.3	3.14	127.4	15.69
9.10	1.82	59	16.3	46	24.3	29	1.94	132.6	17.41
15.00	3.02	111	34.3	16.2	17.9	46.5	1.44	130.7	5.92
4.92	1.56	50.2	15.82	95.6	19.7	47	1.43	141	12.94
2.40	4.44	108	34.6	60	15.5	59	1.33	130.8	5.83
7.67	3.16	106.2	32.3	112	17.5	48.2	1.41	135.9	11.89
3.50	3.97	119	35.4	198	16.5	57	1.53	137.9	10.68
10.93	2.78	74.2	24.32	211	16.2	54.3	1.31	141.6	9.2
5.13	2.22	65.2	19.32	67	15.7	57	1.27	141.8	7.39
5.25	3.04	103.2	30.1	29.2	19.1	49	1.39	134.2	6.29
3.29	2.71	59.2	20.12	304	15.6	57.6	1.27	143.2	12.24
3.04	1.98	66	17.4	85	16.1	54.8	1.31	138.2	10.36
2.00	4.69	147	44.6	70	19.3	41.4	1.55	132.6	5.15
5.42	2.29	71	18.7	77	19.9	39.5	1.6	135.2	9.6
6.43	2.33	76	21.2	46	21.4	35.3	1.72	145.3	5.63
2.06	2.7	91.6	26.89	69.5	17.7	47.4	1.43	140.1	8.2
7.71	3.28	125	33.4	40	16.9	50.9	1.37	132	3.01
3.10	2.9	82.2	24.82	44	15	61.4	1.22	142.4	8.43
6.29	3.11	98.2	29.1	31.2	17.1	50	1.38	136.5	8.52
1.44	3.09	108.2	30.9	65	17.9	46.5	1.44	128	4.21
0.94	4.85	140	38.7	128	13.2	75.2	1.08	143.2	7.17
2.87	2.6	72	21.1	49	20.6	37.2	1.65	141.7	10.47
0.86	3.12	97	26.4	154	17.3	49	1.34	140.8	4.12
15.75	3.22	110	31.4	43.4	16	56.1	1.38	134.9	17.25
11.32	2.74	89	28.3	102	29.8	21.1	2.55	122.8	9.99
14.31	3.37	109.4	28.4	74	21.7	34.7	1.86	122.6	9.69
3.34	2.13	59.4	18.49	164.5	12.7	80.7	1.09	130.9	10.77
7.84	2.65	90	26.6	26.4	21.4	35.5	1.83	142.4	24.6
15.47	2.55	88	26.9	30.4	37.3	21	3.45	133.4	26.52
12.92	4.7	147	38.7	93	23.3	30.9	1.86	130.6	7.07
4.88	2.81	87	25.82	101	35.6	18.1	3.04	133.8	9.67
4.18	1.36	54.2	15.72	43.4	25.8	26.5	2.21	120.3	14.1
6.56	2.52	85	25.02	34.4	21.9	34.2	1.88	146	24.84
3.34	2.51	59.3	18.54	119.7	21.4	37.4	1.83	138.8	13.5
5.44	3.93	124	34.4	51	71	5.4	6.01	131.2	3.87

11.37	3.08	91.4	26.3	85.4	40.1	19	3.71	128.9	12.43
26.40	3.08	85	25.92	76.4	18.7	48	1.73	129.8	8
10.83	2.06	76.2	22.62	33.2	29.8	21.1	2.36	126.2	14.34
9.33	4.87	149	41.8	129.4	40	20	3.66	134.2	7.78
5.29	2.11	75.2	21.82	52	32	27	2.96	143.3	25.6
12.40	5.21	158	47	109	20.4	38	1.64	123.4	10.47
9.05	0.87	33.2	10.82	86	15.5	59.2	1.33	135.7	16.21
2.96	1.37	50.2	15.22	86	29	20.9	2.48	140.2	28.29
57.66	2.68	91.4	24.82	253	21.6	35	1.85	124.3	9.95
2.93	3.41	127	35.2	85.4	17.6	48.3	1.51	128.2	16.37
6.43	4.32	133	38.8	189	45	17	3.97	134.4	11.05
12.00	2.7	84	22.4	19	28.3	16.6	2.3	139.6	7.57
19.62	2.21	87	24.52	96	26.8	27.1	2.29	130.8	30.46
17.84	2.38	83	23.12	28.4	30.9	19.9	2.64	112.6	30.25
7.15	2.55	93	26.5	49.4	23.1	31.5	1.98	132.5	5.63
9.61	2.1	73.2	21.72	58	31.7	27	2.94	127.2	16.36
5.09	2.37	79	22.9	82	22.1	40	1.93	131.9	10.2
4.29	5.28	149	42.5	126	20.4	38	1.64	128.2	7.2
2.37	5.16	166.3	48.62	90.8	30	28	1.96	134.6	3.75
13.37	3.74	127	36.4	342	16.9	55	1.56	136.3	21.43
7.65	1.9	77.4	20.92	129	28.8	31	2.67	135	11.23
14.92	1.88	64.2	18.62	54.4	22.4	39	1.7	124.9	26.16
8.08	3.78	120.4	31.3	93.4	37	21	3.43	120	8.09
19.33	2.31	85.4	21.62	55	20	39.5	1.72	103.9	8.25
9.40	3.79	131	36	101.4	15.6	51	1.28	138.2	20.73
2.29	3.95	132.3	40.22	73.1	26.7	25.1	2.28	137.5	4.87
6.89	4.66	104.2	34.5	285	48.8	5.9	4.01	137.3	0.56
32.78	2.14	81.6	24.28	37.6	24.9	27.6	1.98	131.2	20.11
1.85	4.96	132	41	156	18.8	43.5	1.61	135.3	3.61
1.63	3.78	145	41.1	153	12.6	81.7	1.09	132.7	8.01
37.07	2.34	95.7	26.59	24.2	16.2	61	1.2	120.8	9.32
3.71	1.8	75	20.67	54.8	23.9	36	1.8	129.4	3.67
5.00	2.78	110	28.6	35	29.5	21.5	2.33	119.8	18.04
3.86	4.41	137.6	39.49	109.1	24.1	37	1.65	143.7	6.26
1.95	2.46	89	24.82	90	19.1	42.5	1.64	135.6	7.7
4.42	2.05	83.2	23.42	54	28.3	22.5	2.24	125.5	17.7
4.49	1.48	37.2	12.22	116	25.2	33	2.11	137.7	21.89
8.66	2.52	70.2	23.72	151	17.3	49.6	1.49	136.4	11.51

4.23	5.13	167	46.3	225	21.4	35.5	1.83	124.8	3.6
4.38	1.68	59.9	17.69	38.1	27.9	31	2.36	126.5	30.29
5.14	3.63	129	33.8	19	23.2	31.2	1.85	132.6	4.42
6.26	3.07	131.4	34.3	194	27	33	2.5	142.7	19.08
4.36	3.57	126.6	35.49	145.4	19.9	39.5	1.6	137.8	2.5
6.89	2.7	98	28.1	49.4	19.4	41.4	1.66	126	16.87
6.51	4.32	135	39.5	144	28.1	23.1	2.4	140.6	5.3
9.01	2.35	87.3	26.69	31.8	25.5	33	1.98	137.1	20.97
8.01	2.65	93	26.5	61	20.2	38.9	1.73	128.3	4.93
4.63	5.05	152.4	46.61	164.2	23.5	33	2.14	138.9	3.47
9.13	1.72	48.2	15.32	45.4	18.5	44.7	1.59	151.1	15.84
1.97	3.91	106	31.6	62	12.7	80	1.18	142	3.09
1.90	4.18	133	38.91	43.8	13.4	79	1.03	142.1	5.41
3.80	1.38	44.2	12.92	34.4	17.2	49.5	1.39	136.3	11.31
0.68	3.13	109	36.9	38.4	23	30.7	1.97	150.3	33.7
20.99	3.09	70.2	22.62	64.4	24.3	28	2.08	128.4	25.49
36.00	1.57	66.2	18.62	38.2	27.9	23	2.21	132.9	15.13
8.81	1.24	51.2	14.92	35.4	18.5	44	1.59	132	29.65
10.00	2.98	94	28.4	170	18.5	50	1.61	137.1	19.03
9.21	2.86	87	27.6	46.4	20.9	36.9	1.79	150.5	7.68
7.67	2.62	92	25.62	63.4	23.6	30.4	2.02	132.5	8.4
1.98	3.84	126.6	38.22	70.5	16.5	53	1.28	133.3	5.69
1.58	3.87	127.8	37.63	32.6	17	50.4	1.38	142.4	6.15
4.00	3.09	89.2	28.1	211.4	19.4	40.9	1.56	139.3	10.11
5.30	2.52	88.2	26.09	69.6	12.2	87	0.95	140.5	4.91
5.14	3.81	116.4	30.9	198	17.5	48.3	1.5	135.8	4.08
3.42	3.36	123	35.2	64	14.5	64.5	1.18	129.4	14.71
8.46	2.65	94.4	25.82	43.4	23.5	30.5	1.88	130.4	16.19
10.00	4.03	113	33.9	65	14.9	61.8	1.21	135.8	5.51
7.71	3.18	101.2	30.3	51	19.5	40.8	1.57	130.6	5.91
1.67	5.22	174.1	50.31	47.2	15.4	58.8	1.25	139.1	5.27
6.35	3.22	106.3	31.5	109.2	15.7	57	1.35	136.8	8.05
4.36	3.99	121	36.3	59	15.5	59.2	1.33	141.3	6.62
3.94	2.88	95	27.8	96	15	62	1.29	134.7	17.69
1.92	1.13	35.2	12.22	23.4	21.4	37.4	1.83	135	3.71
1.11	2.95	99.1	28.34	41.9	13.4	79	1.03	143.8	4.12
1.65	2.84	106.9	30.88	50.7	17.3	50.6	1.49	139.7	11.21
2.78	2.97	91.9	26.83	107.8	17.8	54	1.29	129.7	7.51

2.11	3.7	118.4	31.7	168	21.7	33.8	1.86	139.2	5.18
1.21	2.73	101	28.34	38.2	19.9	39	1.71	145.8	2.97
7.76	1.87	58.2	16.72	153	16.4	53.8	1.41	133.7	13.29
2.47	3.38	132	36.9	81	17.1	50.6	1.47	137.2	3.75
13.64	4.85	163	44.9	78	30.3	20.5	2.59	136.6	6.21
1.81	2.13	82	23.62	84.4	14.5	65.5	1.25	135.2	9.52
3.02	2.52	90	25.72	46.4	17.6	53	1.41	132.7	6.61
11.08	4.62	143	39.4	81.4	16	56.1	1.38	137.2	14.03
1.96	4.1	106	34.4	108.4	12.8	79.7	1.1	132.3	7.29
1.74	2.43	77.5	22.71	44	14	69.3	1.21	136.6	7.11
14.00	3.14	103	30	35.4	15.6	58.4	1.34	131.9	10.37
2.91	1.74	59.2	18.82	86	15.3	60.2	1.32	131	8.91
2.14	3.35	122	36	52	14.1	68.5	1.21	142.1	3.87
1.77	2.01	58.2	18.02	34.4	13.8	70.8	1.19	144	5.47
23.40	2.82	101	30	91	17	51	1.46	143.6	13.89
8.09	1.53	52.2	17.22	80	13.7	71.7	1.18	127.4	25.55
4.14	2.53	83.9	24.6	18	16.5	58	1.43	138	7.15
3.59	2.26	58	18.19	61.8	14.8	67	1.28	132.7	15.19
0.91	3.28	113.9	35.05	132.4	16.1	60	1.4	136.4	7.42
1.87	1.93	83	23.42	52	14.6	64.8	1.26	133	6.84
13.58	3.26	114	33.6	46.4	17.5	48.7	1.5	137.4	7.23
5.08	3.87	117.3	35.43	22.2	29.1	21.9	2.49	131.4	16.94
3.75	2.94	119	32.8	23.4	17.1	50.6	1.47	134.8	16.31
4.91	4.18	142	41.2	207	15.2	60.8	1.31	145.4	9.74
2.98	2.01	69.2	20.42	85	13.9	70	1.2	138	9.39
3.27	2.17	67.2	21.12	172	14.7	64.1	1.26	154.3	16.17
11.64	3.05	94	29.2	62.4	13.3	75.1	1.15	131.5	13.52
1.07	2.87	95	29.9	90	19.6	40.8	1.68	143.5	2.54
26.71	4.63	141	38.7	51.4	19.9	39.8	1.71	129.7	5
7.95	3.61	91	28	85.4	17.8	47.5	1.53	139.7	4.72
9.83	1.73	39.4	12.86	48.6	20	39.5	1.72	138.3	17.47
1.28	2.34	74.2	22.52	65	17.9	47	1.54	140.2	5.19
28.13	3.31	99.4	27	180.4	18	52	1.67	122.5	12.57
2.57	2.22	77.5	23.63	42.4	15.1	61.5	1.3	134	8.16
2.38	3.36	110	33.1	52	14	69.3	1.21	145.2	9.88
3.21	3.13	110	32.1	51	15	62.1	1.29	136.7	5.01
1.46	3.53	114.4	30.4	94	44.2	18	4.09	139	2.94
23.78	1.99	73.2	20.72	321	18.5	44.7	1.59	129.3	11.52

3.26	3.52	120.9	37.05	135.4	13.7	71	1.27	138.1	7.14
12.37	3.54	105	31.7	49.4	18.4	47	1.58	132.6	14.23
6.71	2.25	54.2	17.02	105	14.5	65.5	1.25	138.6	9.27
4.69	2.21	60.2	18.02	44.4	15	62.1	1.29	131.1	34.23
6.52	2.67	91.4	24.32	92	17.5	48.7	1.5	134.6	27.22
6.35	3.74	123	36.6	69	29.2	28	2.7	146.5	2.88
4.25	1.52	52.4	13.92	73	31.4	27	2.91	131	6.3
1.85	3.31	110	32.5	54	20.8	44	1.93	141.6	4.6
2.51	2.5	89	25.22	35.4	24.3	37	2.25	136.1	3.43
4.66	3.02	111	31	52	28.6	30	2.65	138.5	3.49
12.15	4.12	135.4	36.9	122	62.8	13	5.81	124.4	5.44
7.13	1.74	66.4	17.72	43.4	36.7	23	3.4	131.5	32.96
4.50	5.61	187	53.6	77	21.4	41	1.97	139.4	7.85
0.74	3.83	122	35.5	67	19.6	48	1.81	143.4	5.02
20.72	2.65	84	23.62	73.4	15.2	66	1.33	134.5	9.73
2.11	1.79	61.2	18.62	23.2	28.5	31	2.64	140.2	5.73
9.67	2.11	71.2	20.62	41.4	16.1	64	1.49	137.6	15
3.62	2.49	91	26.6	65	19.5	49	1.81	137.5	5.85
7.57	2.94	96	25.96	14	18.8	52	1.74	137.8	6.8
1.90	2.41	93	26	45.4	18.4	53	1.7	136.8	4.51
2.92	2.42	56.2	17.72	96.4	16.8	60	1.56	142.9	11.39
0.65	2.35	88.6	25.83	81.5	21.9	40	1.92	146.1	5.13
7.34	1.19	37.2	11.52	145	15.2	69	1.41	140.5	14.88
4.54	2.65	91	26	84	17.6	56	1.63	142.5	17.03
2.43	2.02	60.2	19.02	87	14.7	67	1.36	141.7	7.57
11.78	3.24	113	31.3	131	15.9	60	1.47	121.6	7.44
1.88	3.59	120.9	35.14	67.8	15	64	1.31	137.1	5.25
2.05	3.76	114	34.9	330.4	10	132	0.87	135.3	5.06
2.80	2.71	86.9	25.27	72.1	16.5	57	1.53	141.8	8.97
3.34	4.26	152	42.9	69	19.4	50	1.8	134.2	8.8
33.31	3.62	133	38.7	90.4	23.9	38	2.21	133.5	8.16
5.09	3.77	127	36	30	22.7	41	2.1	136.5	13.73
4.77	1.83	68.2	19.22	50	29.1	31	2.69	125.3	29.88
0.89	2.55	89	25.02	134	22.4	42	2.07	136.3	8.25
5.56	2.9	103.4	27.3	77	26.7	32	2.28	118.7	3.97
12.95	3.5	103	35	79	16	59	1.48	154.5	41.62
12.33	3.22	117	30.7	99	15.5	58.3	1.26	118.4	11.39
23.03	3.84	96.5	31.53	95.8	16.2	57	1.49	145.7	6.58

4.20	1.87	65.2	17.82	14.4	21.9	42	2.03	135.5	18.29
7.82	4.29	155.4	41.5	73	15.3	60.2	1.32	119.2	9.24
2.03	2.49	76.2	22.82	170	14.8	66	1.37	141	12.32
3.27	2.64	71.2	22.92	24.4	21.4	35.5	1.83	139	5.17
2.62	3.43	125	36.5	61	26	32	2.41	148.4	14.28
14.67	2.34	90.2	24.02	33.2	21.5	42	1.67	127.5	15.28
9.26	2.43	90	26.92	28.2	39	20	3.5	132.3	6.84
1.07	3.26	106	28.8	190	15	61.2	1.22	138.1	5.88
10.32	2.46	87	24.82	98	16.8	51.7	1.44	129.8	28.26
12.71	1.55	57	16.8	20	29.6	29	2.53	126.7	22.02
2.74	2.77	102	28.6	200	16.4	62	1.52	137.6	5.2
2.05	3.58	119.5	35.45	86.4	20.5	37.1	1.76	137.9	9.37
3.99	2.23	71.2	20.12	94	16.3	59	1.42	138.6	8.05
4.35	2.89	92	31.9	49.4	15.9	59	1.47	144.9	8.05
5.53	2.69	105.7	31.4	16.7	42.1	19	2.58	139.8	11.85
2.89	3.44	96.1	28.94	90.8	15.5	58.3	1.26	137.8	9.29
21.04	3	112	31.7	41.4	14.5	65.9	1.25	125.9	10.74
11.34	3.41	123.4	33	27.4	22.9	31.9	1.96	122.1	25.69
15.00	3.45	118	35.2	39.2	19.6	40.4	1.58	128.5	4.57
1.44	3.96	96	29.7	138	15.5	61	1.35	135.5	3.29
24.33	3.7	100	28.4	178	14.2	66.6	1.16	128	23.15
2.13	2.99	67.2	22.12	287	14.1	72	1.31	133.8	7.51
18.65	2.74	87.1	27.23	66.3	13.3	74	1.23	142.4	11.49
5.68	3.69	127	35.5	127	24.3	29.1	2.08	135.7	5.09
9.13	2.08	59.7	17.31	48.7	14.2	73	1.23	143	3
1.09	3.91	135	38.4	135	16.9	51.2	1.45	133.4	4.21
5.86	2.87	104	29.4	73	17.1	54	1.58	132.7	11.22
2.49	4.61	103	31.1	154.4	16.3	54.5	1.4	138.4	4.94
4.89	1.76	54.6	15.92	138.6	13.4	74.2	1.15	141	11.12
6.53	3.06	97	28.1	71	14.1	71	1.31	146.4	17.71
3.44	2.6	96.7	26.95	11.8	24.8	28.1	1.98	126.3	4.73
3.27	3.2	108	32.2	90	14.6	64.8	1.26	139.3	3.6
2.95	3.5	113	32	81	23.3	33.1	2	134.3	2.67
1.31	3.55	101	33.96	48.8	15.2	57	1.4	144.9	3.9
1.06	2.74	97.6	28.91	150.6	11.2	104	1.04	135.7	3.91
4.00	2.92	96	24.7	98	14.8	56.6	1.19	133.3	4.22
14.70	2.12	76.9	22.24	75	25.8	33	2	127.3	6.57
5.82	4.59	137.4	35.7	148	40.3	21	3.73	135.2	2.21

0.81	3.47	124	35.7	88.4	15	62.1	1.29	140.3	5.35
7.43	2.82	87	25.32	41.4	14.1	68.5	1.21	135.9	11.18
6.66	3.44	108	31.6	123	13.4	74.2	1.15	132.2	24.78
3.54	3.07	114	33.5	42.4	23.6	30.4	2.02	128.2	7.85
3.46	3.12	106	29.6	29.4	21.1	36.3	1.81	138.8	11.89
4.75	2.93	69.2	22.92	36.2	20.7	37.2	1.66	137.7	13.26
1.63	4.01	138	40.2	73	16.3	54.5	1.4	142.7	9.02
2.29	2.13	62.2	18.42	84	18.4	47	1.58	142.9	8.35
2.25	2.79	92.2	27	80	15.3	59.4	1.24	134.8	10.5
2.17	3.32	119	33.7	107	20.9	44	1.94	139.9	2.97
3.42	2.3	73.2	23.12	23.4	19.9	39.8	1.72	127.1	27.29
8.71	3.02	113	32.8	28.4	24.7	36	2.29	134.7	9.61
2.46	2.2	50.2	19.52	106	14.1	68.5	1.21	138.5	9.47
1.83	4.05	146.6	41.72	32.1	16.6	52.7	1.43	141	2.51
4.85	3.13	112	32.9	102	17.8	47.5	1.53	138.2	5.18
6.70	2.72	96	30	219	14.5	65.5	1.25	137.7	11.76
3.58	2.56	96	28.1	55	20.1	39.2	1.72	138.7	6.04
3.63	2.89	95.4	25.82	70	17.2	57	1.59	129.7	10.6
5.59	3.09	102	28.5	49.4	14.5	69	1.34	132.2	16.19
4.30	2.22	64.2	19.72	144	16.4	57	1.52	145.5	9.86
2.94	2.93	106.2	28.9	146	23.5	30.5	1.88	124.4	3.6
7.14	3.4	84.9	26.77	54.2	17.3	49.6	1.49	130.4	10.32
3.30	3.01	94.6	27.8	94	13.5	76	1.25	136.9	9.67
6.16	4.37	134	38.5	35.4	15.2	63	1.41	136.7	4.85
7.98	2.06	65	19.3	34	17.5	54	1.51	139.9	7.79
1.28	3.3	113	32.6	107	17.2	57	1.59	137.1	8.13
6.78	3.19	115	33.7	149	13.2	85	1.22	142.4	10.34
4.03	3.11	107.3	30.22	37.7	14.4	71	1.09	140.6	4.66
2.60	3.1	102	28.2	49.4	15.6	58.4	1.34	134.5	10.47
7.00	1.75	65.2	17.82	24.4	24.1	35	2.23	127.6	8.84
14.75	4.38	151	43.4	92	15	69	1.39	135.4	13.56
3.51	2.39	87.4	23.62	30.4	16.1	55.6	1.38	128.6	13.6
7.67	3.36	107	30.8	47.4	15.6	51	1.28	133.9	11.64
5.71	2.4	83	24.22	114	13.1	76.9	1.13	133.6	10.59
0.62	1.66	59.2	17.52	83.4	15.4	61	1.43	134.2	5.59
1.05	3.29	119	34.1	44.4	14.3	69	1.32	134.9	10.19
8.47	2.63	76.2	23.42	27.4	19.7	44	1.82	142.3	15.8
3.17	3.27	78.2	26.9	27.4	17.5	48.7	1.5	137.9	9.18

3.60	3.17	102	30.3	84	16.4	57	1.52	142.4	5.24
6.31	3.25	85	25.72	86	13.3	77	1.23	130.3	6.87
4.15	3.86	127	35	67	13.6	75	1.26	146.8	8.46
19.66	2.02	45.2	15.92	129	18.3	51	1.58	134.3	12.61
4.72	3.92	101	33.1	95.4	14.4	57	1.2	140.3	5.43
2.18	1.59	55.2	16.92	36.4	20.7	36.5	1.77	136.1	6.76
1.16	2.57	79	22.5	39	13.4	74	1.04	134.8	7.32
5.41	2.11	66.7	19.84	87.7	13.5	76	1.25	140.3	15.88
2.31	2.22	92	26.8	59	17.4	48	1.6	138.3	6.23
1.94	1.83	47.2	16.82	88	14.7	64.1	1.26	138.7	10.19
2.36	3.32	86.5	25.11	81.4	17.7	54	1.29	137.2	5.34
3.02	2.45	51.2	18.02	40.4	19.1	50	1.77	142.8	9.66
7.76	2.59	106.4	28.6	58	19.4	41.4	1.66	137.2	11.37
3.85	2.93	95	28.5	109	13.8	76	1.2	147.2	13.27
5.21	3.72	128	37.2	45.4	15.8	60	1.46	140.3	4.17
6.67	3.04	93.3	28.95	71	11.5	88	1.06	133.4	16.25
1.84	3.34	87	26.3	26.4	17.6	54	1.52	136	5.7
2.58	2.96	113	32	49	16.7	58	1.44	144.3	4.64
8.34	2.12	77.2	22.22	56	14.4	66.7	1.24	129.5	11.99
2.06	1.86	59.2	17.72	77	13.8	75	1.2	140.8	3.32
7.62	3.46	124	34.9	42.4	16.1	60	1.4	139.7	5.41
3.79	3.7	110	31.7	89	16.4	54	1.41	135.5	6.34
4.33	3.78	110.9	32.64	76.2	14.1	75	1.31	143.2	8.66
2.44	1.38	50.4	14.62	72.2	24.7	32	2.16	140.6	15.39
4.22	2.76	77.2	25.52	124	15.7	61	1.45	141.8	10.61
1.69	3.48	122.4	33.2	30.4	21	45	1.94	125.4	4.46
1.63	2.67	94	27.9	68	15.2	68	1.41	141.2	5.36
3.82	2.82	73	24.1	118	14.8	66	1.37	134.7	5.21
7.40	2.73	74.2	25.12	156	12.9	77.3	1.11	140.4	8.39
0.72	2.95	88	26.1	175	26.7	30	2.47	139.7	5.77
4.35	3.23	99	29.1	71	15.7	61	1.45	143.2	5.36
1.97	3.37	97	29.2	95.4	16.2	54.9	1.39	138.6	5.7
21.96	2.49	69.2	21.92	189	12.5	86	1.16	142.3	15.96
2.22	2.64	93	29	19.4	17.3	49.2	1.49	146.1	6.82
7.74	3.1	101	30.3	199	16.3	58	1.51	146.2	7.73
0.99	5.39	171	51.29	122	15.2	60.8	1.31	136.6	7.71
2.08	2.66	84	23.22	28.4	13.2	79	1.22	137.3	10.69
18.55	2.42	80	23.92	79	20.3	38.6	1.74	135.4	9.47

2.79	2.64	94	25.82	34.4	13.6	81	1.26	133.2	14.42
2.98	3.62	72.2	23.32	39.4	16.9	60	1.56	141.7	14.14
1.41	2.99	110	31.4	76	13.3	75.1	1.15	142.4	11.75
1.82	2.79	120	34.6	80	21.8	34.3	1.87	137	11.62
4.22	2.59	65.3	20.4	75.8	15.9	56	1.23	129.1	10.57
6.28	2.68	101	28.9	49.4	13.9	70	1.2	132.5	8.15
4.87	2.91	69.2	23.82	81.4	16.3	58	1.51	144.7	7.73
3.36	3.65	107	32.8	112	14.8	65	1.29	137.1	12.72
1.37	2.59	86	24.82	45.4	20.8	42	1.93	147.7	3.73
4.96	4.43	147	42.6	42.4	15	62.1	1.29	143.9	8.76
1.77	3.2	104.5	29.99	83.1	12.4	89	1.06	144.2	6.95
2.74	2.17	73.2	21.02	144	13.1	86	1.21	142.5	12.96
3.70	3.21	101	29.2	54	13.2	76	1.14	146.7	6.45
1.69	2.7	111.4	30.1	80	19.3	41.8	1.66	138.1	6.52
5.50	2.39	73	21.5	82	13.6	77	1.18	139.1	4.73
4.53	3.29	86.7	26.93	39.7	16.2	58	1.5	138.8	8.36
6.19	3.61	124	35.6	52	15	62.1	1.29	134.8	3.74
8.44	2.13	58.2	18.32	108	15.4	68	1.43	145.9	9.75
1.76	3.09	117	32.4	83	16	56.1	1.38	142.6	7.27
4.50	3.23	105	30.6	72	13	77.8	1.12	140.1	12.82
4.45	2.34	72.2	22.92	90	14.1	74	1.31	134.6	15.04
3.26	3.62	115	34.1	77	16	56	1.38	139.8	8.25
9.56	4.15	134.4	35.8	170.4	25.6	35	2.37	136.4	4.07
21.26	2.92	86	25.72	37.4	15.3	68	1.42	141.8	9.04
8.02	2.2	61	19.2	91	14.7	69	1.28	140.8	11.22
4.44	3.18	79.2	25.42	84	15.1	61.5	1.3	138.2	11.4
4.91	2.71	96	26.8	41.4	16.2	54.9	1.39	141.3	9.78
0.91	3.25	98.6	29.58	142.3	13.4	79	1.17	146.1	3.6
4.88	3.26	98	28.9	254	14.1	68.5	1.21	136.9	12.09
1.63	4.1	133.7	40.03	54	14.9	63	1.38	144.1	3.66
3.83	3.75	86	28.8	139.4	13.4	83	1.24	137.5	9.12
21.78	2.32	80.1	23.06	35.6	15.8	63	1.18	129.8	8.3
5.23	3.36	107	32.5	36.4	17.6	47.8	1.51	143.3	6.13
15.48	3.07	97	28.3	75	15.4	59.7	1.32	139.6	11.32
6.40	3.18	107	29.7	165	17.3	58	1.6	133.9	11.65
19.15	3.33	111.4	29.9	216	18.1	53	1.68	137.8	22.32
2.48	3.86	138	39.1	46.4	16.2	63	1.5	143.9	5.72
4.71	3.37	111	31.3	53	15.5	59	1.33	136.9	6.15

4.69	4.34	152	42.1	103	14.7	64.1	1.26	135.7	10.07
6.71	3.23	84	24.82	33.4	11.7	97	1.08	138.7	12.01
0.99	3.78	124	37.7	65	14.3	72	1.32	143.2	1.47
1.31	4.14	132.7	39.52	107.2	16.9	50	1.55	141.6	5.09
1.17	3.23	117.2	35.4	86.9	13.6	68	1.25	142.8	6.52
5.88	2.35	68.2	21.82	123	13.1	75.5	1.07	140.1	10.49
9.93	3.79	89	29.3	76.4	15.9	56.7	1.37	133.4	7.34
5.19	2.16	78.2	21.82	29.4	14.1	68.5	1.21	139.7	10.49
6.54	4.18	124	36.6	170	13.6	71.3	1.17	120.5	11.78
2.82	4.43	107	32.9	17.4	17.9	47	1.54	135.7	7.16
4.06	4.17	98	31.31	195.1	12.1	88	0.94	137.5	5.41
4.40	3.29	66	22.1	53	16.1	54.9	1.31	139.8	6.78
0.85	3	105.4	30.97	42.1	13.9	75	1.06	134.3	7.12
4.67	3.42	111	32.7	41.2	13.2	76	1.02	127.7	3.63
1.50	4.19	146	41.6	25.2	13.6	71.2	1.11	140	6.41
10.10	2.62	71.2	21.74	31.4	14.6	65	1.35	130.6	8.24
2.05	3.19	107	32.5	56	17.4	52	1.61	142.5	4.26
4.89	4.08	142	41.9	77	18.8	38	1.46	132.6	7.32
1.24	3.74	129	37.3	12.4	16.1	54.8	1.31	134.1	1.48
4.35	1.96	47	15.08	71.3	13.6	72.5	1.17	142.3	7.31
8.28	2.8	89	26.7	49.4	13.6	72.5	1.17	140.2	13.84
8.27	3.19	97	29.8	32.4	18.5	44.7	1.59	135.5	7.52
6.37	3.63	111	31.8	87.4	21.9	34.2	1.88	139.5	7.18
27.00	2.2	55.2	17.22	60	24.5	34	2.05	136.4	8.15
2.89	3.43	125	34.4	48.4	13.9	70	1.2	137.4	6.13
1.14	2.49	81	24.52	46.4	14.7	64.1	1.26	139.9	3.49
2.58	4.47	153	43.5	121	15.5	59	1.33	136.8	6.29
6.31	2.19	78.2	22.92	21.4	23.5	30.7	2.01	141	8.35
1.51	3.34	86.4	27.49	75.2	11.6	86	1.07	137.6	9.91
6.29	2.44	79.2	22.72	48.4	13.4	69	1.24	140.3	8.92
1.22	2.75	95.4	26.92	22.9	14	74	1.07	141.2	6.3
5.09	3.36	108	29.1	107	15.2	60	1.23	136.3	13.36
4.46	2.59	83	23.92	111	15.9	56.7	1.37	138.2	7.08
3.80	2.6	77.2	23.02	30.4	14.6	64.8	1.26	142	4.23
1.44	2.43	87.7	27.66	230.9	15	62.1	1.29	138.3	7.16
2.47	2.81	99	29.4	34.4	20.7	37.4	1.77	137.6	4.55
5.90	2.45	65.2	20.42	249	14	68.1	1.14	135.4	8.23
8.73	3.31	101.5	30.16	87.8	14	68.5	1.14	131.4	8.29

10.92	2.18	75.2	21.62	55	15.3	60.2	1.32	142.1	7.08
3.06	2.91	98.8	29.19	41.8	16.3	52	1.5	143.6	4.79
2.31	3.25	104.2	29.3	47.2	13.6	71.2	1.11	139.1	4.88
8.51	2.1	68.2	21.22	24.4	17	51	1.46	140.5	10.36
1.58	3.95	130	37.5	49.4	15.8	50	1.29	139.9	4.36
2.85	1.05	29.2	8.42	61	15.4	59.6	1.32	122.5	7.95
1.66	3.58	121	34.8	107	13.6	73.3	1.17	133.1	5.96
4.86	2.94	94.2	26.88	75.2	14.8	63.5	1.27	132.2	12.49
2.43	3.08	76.2	25.02	24.4	14.4	66.2	1.24	138.6	5.44
2.95	2.09	68.3	19.96	19	19	49	1.38	145	7.78
2.68	3.84	130	37.3	22.4	15.1	61.5	1.3	140.9	4.38
7.19	3.07	66.4	21.29	114.1	17	57	1.25	134.6	13.29
6.00	3.6	107.2	30.6	69	14.3	65.9	1.16	130.2	8.06
1.23	3.97	97	30.4	59.4	16	56	1.38	135.1	3.66
5.52	4.22	98	31	113.4	14.2	68.2	1.22	136.9	6.57
3.79	1.92	53.2	18.22	76	19.6	40	1.68	142.1	17.56
11.00	3.1	103.9	30.95	31.1	15.1	67	1.13	141.9	5.34
6.14	3.13	115.7	32.79	152.4	17	50.4	1.38	102.6	8.27
5.13	2.22	65.2	19.32	67	15.7	57	1.27	141.8	7.39
1.55	4.07	133	38.2	28.2	17.5	48.2	1.41	137.1	5.11
7.84	2.65	90	26.6	26.4	21.4	35.5	1.83	142.4	24.6
4.14	1.48	52	13.2	44	44.3	11.4	3.45	120.2	16.5
7.14	2.74	97	27.7	61	21.4	34.6	1.83	125.4	53.35
2.93	3.41	127	35.2	85.4	17.6	48.3	1.51	128.2	16.37
8.62	1.91	63.2	17.62	62	39.5	12.5	3.36	120.3	39.15
15.40	1.37	58.2	17.52	56	17.2	50.1	1.48	141.8	7.97
13.31	3.01	106	27.9	156	19	42.3	1.53	123.4	18.81
6.97	1.6	60.1	16.76	167.6	65.2	12	3.66	135.8	5.83
13.37	3.74	127	36.4	342	16.9	55	1.56	136.3	21.43
2.29	3.95	132.3	40.22	73.1	26.7	25.1	2.28	137.5	4.87
6.89	4.66	104.2	34.5	285	48.8	5.9	4.01	137.3	0.56
3.86	4.41	137.6	39.49	109.1	24.1	37	1.65	143.7	6.26
5.45	1.87	64.4	17.32	179.4	14.8	63.5	1.27	129.2	20.21
8.00	0.95	40.2	11.32	26.2	30.2	20.7	2.39	119.8	8.19
18.76	2.39	74.7	22.85	56.1	18	50	1.67	121	8.48
6.89	2.7	98	28.1	49.4	19.4	41.4	1.66	126	16.87
1.97	3.91	106	31.6	62	12.7	80	1.18	142	3.09

Cr	GLU	NH4	MELD
102.2	8.79	70	10.72
96	5.84	24	6.61
78.3	10.48	29	4.63
218.3	3.59	131	20.59
61	10.91	23	2.59
155.8	8.26	125	19.31
538.2	8.19	42	38.01
67.1	20.45	59	8.15
184.8	7.07	44	10.77
207.4	18.3	96	21.07
218.4	9.42	84.3	35.7
174.5	25.35	179	34.68
150.4	3.39	27	25.72
48.9	21.24	43	8.06
200.1	6.04	43	33.13
107.9	7.02	26	20.36
32.4	22.27	41	3.02
105.7	9.41	70	28.73
80	6.77	33	21.44
73.4	9.4	75	21.74
129.2	6.42	43	18.5
97.7	5.99	31	15.95
96.8	17.69	26	17.25
157.4	10.28	7	9.06
199.4	4.39	34	24.44
265.2	8.13	72	15.88
125.9	11.26	26	24.76
37.6	22.23	34	17.04
135.8	9.25	76	23.53
115	5.84	13	22.79
103	12.52	13	19.89
71.8	4.852	42	18.47
66.3	6.17	6	27.07
91.3	12.52	93	18.12
55	5.3	35	4.65
40.3	8.53	8.7	10.78
69.3	10.07	19	15.26

63.2	5.24	24.5	24.08
112.4	4.16	4	12.04
62	11.86	37	20.34
74.2	5.9	46	5.81
198.9	5.94	82	19.18
55.7	4.64	46	11.14
131.1	6.41	36	29.21
344.4	5.79	29	34.55
72.1	7.77	68	16.83
68.6	11.57	28	7.04
145.6	7.71	40	27.77
122.6	6.71	86	11.12
95.5	5.49	35	15.35
280	11.48	98	29.32
63.1	5.69	30	26.96
93	8.91	56	16.11
57	3.3	70	26.59
374.7	6.07	34	35.12
118.3	13.91	41	10.91
214	2.2	37	33.3
55.8	8.58	92	12.9
98.3	7.8	57	6.03
259.2	4.3	27	27.73
50.5	9.96	46	7.77
97.7	6.93	38	18.2
149.3	7.14	19	11.9
509	7.29	23	41.37
55.6	6.21	30	21.56
197	13.49	5	27.77
62.4	21.33	54	6.74
106	5.55	86	4.92
147.8	6.04	17	23.99
161.3	5.88	43	22.68
35.6	9	37	23.18
62.9	8.07	17	10.96
521.4	7.62	15	20.4
76	5.27	22	6.21
54	6.65	14	17.16

39	5.67	36	2.79
56.3	13.67	44	5.97
27.2	11.63	54	19.9
67.9	6.57	53	6.03
57.2	8.89	19	6.46
69.6	36.94	83	9.72
38	6.39	66	9.43
84.8	11.79	71	2.9
113.1	5.62	19	26.52
99.6	6.51	7	9.56
104.5	15.11	36	9.27
53.3	14.26	56	10.74
109.3	30.24	37	25.27
31.4	7.84	42	6.81
92	5.53	69	16.92
57.8	7.02	25	7.82
59	3.94	58	22.34
100.3	7.65	26	31.65
82	6.47	13	20.3
57.5	9.31	21	13.56
63.4	6.02	37	3.8
70.9	8.67	68	26.38
252	2.79	20	36.78
158.8	19.91	36	18.06
112.3	6.74	79	19.17
64.6	7.68	53	6.62
173.3	1.73	58	23.26
58.9	7.36	27	7.33
28.7	7.37	27	16.65
78.9	22.59	63	5.56
76.5	4.91	46	7.68
79.9	7.79	29	14.11
61.6	16.4	23	10.1
64.7	6.66	43	22
89	6.64	14	11.83
93	5.96	27	17.31
68.1	6.32	36	17.24
70	3.31	22	17.57

53	4.05	9	8
64.5	5.61	21	9.89
162.8	6.26	40	19.39
58	7.97	33	3.19
68	6.95	24	19.69
89.9	11.33	36	7.58
159.5	10.89	44	22.02
66	7.1	11	22.91
65.1	6	17	11.13
37.7	20.53	20	11
56	10.94	29	5.8
93	12.99	243	2.62
72	5.99	19	1.53
35.2	10.87	30	1.95
58.9	11.43	34	23.44
65.2	16.1	57	10.79
49	19.26	63	3.44
46	6.08	47	7.61
47.7	13.76	47	9.12
55.3	11.2	55	9.45
102.2	5.22	25	19.16
108	4.95	12	1.92
55.5	11.27	44	25.14
65.4	6.44	29	19.31
51.4	6.76	16	14.41
49	5.34	24	8.11
58	4.25	62	27.51
57	4.73	56	3.83
51.9	5.26	23	7.36
66.9	7.67	28	24.99
79	13.66	40	8.01
59.5	5.89	51	19.44
167.9	7.7	24	19.36
252.8	7.93	59.8	25.96
122	20.77	37	18.45
91.1	7.89	45.9	14.28
116	6.88	37	8.96
52.6	4.34	51	18.82

119.7	6.65	6	11.86
175	7.51	34	29.98
64	7	33	3.69
68.1	5.29	36	24.86
290	9.29	29.7	39.62
77	7.51	35	2.23
47	8.15	72	4.86
86	5.92	26	10.51
369.3	20.3	18	16.49
84	5.56	40	9.9
62.5	5.99	40.5	7.78
130.5	6.81	44	6.33
44.3	21.45	64.2	7.2
200	5.27	62	27.27
47	6.12	38.2	14.02
50	18.65	42	20.14
129.8	9.98	31.7	6.82
55	5.53	49.9	4.9
81	5.68	60	1.76
222	15.73	71	34.65
87	10.37	38	6.32
238.9	8.67	960	15.35
142.9	7.19	64.5	21.67
113	7.02	21	5.63
61.8	5.3	39	13.8
187	7.53	29	11.5
48.7	10.5	53	24.81
105	6.75	38	24.57
65.5	5.31	20.2	7.46
70.5	5.81	34.7	20.91
60	13.66	55	18.3
90	6.01	23	13.53
281.2	6.93	10	28.46
90	19.54	44	11.95
74.6	6.1	15.89	12.3
65	7.69	44	21.04
162.7	35.41	65	12.08
86	14.29	26	14.39

69.8	7.67	37.6	9.47
116	7.92	34	17.75
194	31.32	28	14.15
70.7	9.16	51	26.08
80	7.55	26	14.49
66.4	13.14	46.3	17.03
94.6	9.24	42.9	14.4
66	7.36	46	11.45
40	8.22	13.8	4.84
51.7	9.02	39.4	4.69
34	5.98	52	12.84
52	4.74	37	27.95
209.8	9.49	55.5	18.87
74.9	8.98	28.9	13.67
68.1	6.69	22.9	5.82
52	5.89	44	27.77
147.7	15.62	43	12.81
288.4	11.05	70	27.63
62.3	11.67	28	8.18
471.9	6.17	51	18.21
66.5	6.38	80	19.04
78.4	5.13	8	5.12
59.7	10.12	88	9.23
118	11.49	87	20.34
113	10.48	21	4.86
88.9	5.23	36	18.06
58.7	14.6	14	6.47
59	6.02	33	8.15
47.1	14.65	20	28.7
65.3	10.39	13	17.64
95.7	7.65	25	5.89
94.8	7.63	15	20.58
132.9	8.74	27	26.33
42	7.65	22	7.48
58	8.09	20	20.29
85	12.45	48	6.85
78.1	8.14	54	4.84
74	18.93	39	3.11

112	4.88	31	20.7
418	3.66	38	34.35
133.9	2.88	48	24.24
64.6	14.57	36	23.13
135.5	3.9	33	33.02
64.1	14.62	48	26.27
60.5	5.36	25	22.97
55.1	8.54	27	23.75
42.6	10.32	69	3.53
253.5	4.44	20	33.7
90.5	6.01	32	24.04
124	4.64	28	17.12
58	5.38	14	18.22
41.5	8.83	21	14.91
57.9	7.12	40	16.4
65.4	11.71	19	5.59
74.9	22.12	39	16.67
48	6.07	39	10.94
61.5	7.99	42	11.93
56.5	7.75	41	18.06
35	8.52	39	21.18
70	4.3	47	11.12
884	7.25	44	52.23
71.5	13.12	58	7.34
59	41.38	52	9.37
61	9	77	17.98
58.1	13.63	45	12.75
153	24.3	33	8.15
58.3	11.9	32	29.99
71	7.89	22	20.12
55.1	6.64	21	8.78
139.9	25.38	21	13.1
333	7.76	41	34.03
33.4	7.97	49	19.76
98	9.86	92	9.73
142	5.91	48	29.89
61.3	6.86	53	19.13
124.6	8.52	15	9.94

42.5	4.47	49	9.68
59.9	15.86	48	11.9
75	6.09	29	13.33
64	4	49	19.21
100.2	13.03	31	8.26
54.5	7.59	27	3.44
53.3	5.64	33	4.98
147.4	9.32	36	22.32
53.6	9.87	26	26.63
62.8	5.46	27	15.98
84.4	9.85	15	10.37
15	10.78	15	19.89
201.5	7.02	38	33.41
107.1	3.72	36	18.64
67.3	10.86	34	17.99
201.4	5.47	32	16.18
89.6	5.53	39	10.3
148.1	6.91	31	11.93
142.6	2.88	32	22.76
59.3	17.72	45	12.61
209.3	7.93	57	12.24
87.4	8.68	19	12.47
89.4	8.96	39	1.93
76	8.36	39	5.78
56.1	11.66	40	8.62
133.2	12.14	40	8.96
53.6	6.72	41	8.09
71.6	7.36	16	28.3
90.7	11.91	30	4.73
66.8	9.96	28	22.6
63.4	5.16	27	9.13
70.5	7.07	30	17.11
120.5	4.91	26	16.99
53.3	5.92	39	9.6
60.9	4.84	13	18.06
123.8	6.93	40	3.23
78.5	5.89	15	19.17
55.7	8.04	63.4	13.01

301.5	14.9	47.5	22.94
84.4	8.45	20.6	7.05
136	5.42	37	23.68
94.9	7.95	27	10.23
159	8.65	30	14.38
61.1	18.51	31	6.19
67.1	5.76	33	3.53
105.9	6.52	7.2	16.37
55.4	7.56	45	18.95
69	4.77	29	21.25
163.1	8.85	26	9.49
724	5.28	12	29.41
63	5.28	12	7.5
104.7	4.85	23	4.12
64.8	4.83	24	20.39
201.4	7.93	97	27.5
147.6	13.37	28.6	17.46
52.5	8.23	26	19.11
65.8	8.36	115	3.44
78	6.18	18	16.41
209.2	5.92	38	29.06
59.6	13.21	23	10.13
79.6	7.43	70	6.56
46.7	17.7	32	20.4
72.1	6.93	48	6.76
83	12.45	12	12.67
67.3	5.91	26	14.27
55.8	15.97	19	7.23
65	6.42	33	14.93
56.6	8.07	35	26.69
47.4	19.6	32	7.41
58	5.54	77	17.45
76	5.03	18	10.93
82	6.76	39	12.43
60.6	4.57	33	14.01
68.4	8.48	48	15.38
82	4.89	10	22.15
50	4.04	39	15.22

30.7	7.72	50.3	2.41
77	4.12	29	3.67
122.9	11.58	54	15.28
49.9	7.18	26	20.62
48	9.1	42	20.49
234.2	4.1	104	20.63
55.7	10.7	35	15.29
175.3	9.62	59	18.76
36.4	7.61	55.3	20.61
67.6	21.43	42.9	4.93
71.6	9.5	57	12.81
184.5	20.35	20	23.13
42.2	6.71	48.5	5.46
71	11.21	24	2.5
59.6	9.81	41	16.9
60	8.54	67	12.38
318.2	9.38	45	31.33
152	7.25	15	10.99
152.6	11.32	20	19.62
35	32.33	76	6.31
54.5	3.7	36	13.03
74.9	7.44	18	13.02
89	5.38	21	6.57
409.9	7	34	36.48
120	5.17	70	28.13
95	10.59	22	9.65
53	12.21	31	18.46
46	6.07	54	15.31
68.1	5.65	52	25.85
75	9.15	25	14.72
76.7	8.59	13.7	3.26
156.7	7.2	35	22.72
94.9	10.17	51.2	7.6
137.6	5.96	15	21.23
82.7	12.42	66	19.11
37.2	5.49	41	11.15
246.4	17.05	25	14.04
53.8	6.69	27	10.7

87	7.4	18	19.49
81.6	5.96	40	3.22
136.6	8.31	20	7.2
53	7.16	45	10.83
150.7	5.91	45	33.24
44.1	4.96	24	18.69
124.7	8.73	49	18.2
105	15.56	6	13.44
72	6.31	41	21.18
80	17.78	42	3.7
54.5	5.54	17	19.89
78.2	8.11	70	13.41
44.6	6.65	27	22.62
668.3	30.14	67	23.18
62.4	8.7	27	28.62
81.2	10.54	42	23.45
47.7	14.81	63	7.42
72.7	6.8	37.7	8.76
96.7	10.21	18.7	17.37
76	5.4	138	18.94
153	7.27	10	27.42
62.9	11.59	32	13.22
52.4	5.93	37	24.85
85.6	7.61	12	23.14
70.3	10.09	37	6.04
130.5	10.63	64.7	24.77
45.3	10.6	6	20.23
96	7.25	42	12.08
52.7	5.82	24	19.81
79	11.04	24.5	1.57
80	6.54	21	17.16
65.8	6.99	30	5.59
95	27.84	46	8.93
45.7	26.25	21	7.93
41.9	7.81	24	3.77
92.5	5.4	18	21.23
76	5.23	17	7.62
34.8	13.23	41	8.2

51.7	15.22	67	10.24
51	9.32	27	17.4
73.3	11.98	68	8.36
47.8	8.23	44.3	7.99
94.9	6.19	48	24.36
75.4	5.66	11	18.95
77.6	21.5	38	7.6
52	6.66	38	8.64
376.2	9.3	23	34.96
56.7	20.6	35	20.12
74.3	5.17	46	4.1
82.9	7.56	24	5.83
54.6	7.13	97	10.51
46.6	6.15	22	20.96
96	4.14	39	16.83
75.4	4.64	36	13.9
53	7.43	41	22.75
66.3	6.41	17	16.7
46	6.31	43	24.89
90.4	7.03	33	5.52
52	4.79	35	13.16
81.3	5.23	16	14.73
172.4	8.53	27	23.56
203	12.74	26	10.41
73.4	5.57	49	3.28
65.1	3.88	5	20.88
69	7.33	15	3.44
56	5.99	28	3.43
106	15.42	71.9	3.83
62	4.99	25	11.09
125.7	9.31	14.1	6.6
187.2	7.93	29	30.66
55.6	8.31	50	20.15
46.1	5.66	30	11.53
119	9.95	30	6.31
63.8	9.58	30	13.91
164	11.39	31	12.92
55.7	13.19	31	17.62

45.7	9.86	63	5.19
81	8.63	22	3.36
81	8.63	22	7.38
101.5	5.03	38	9.25
210	6.32	30	11.58
54.5	9.26	27	12.01
45.9	4.52	48	7.01
51	6.98	58	10.16
85.3	5.2	19	0
58.2	5.33	34	16.52
56	10.86	26	9.07
96.8	7.09	30	9.87
61.6	8.35	30	7.78
75.2	7.6	36	4.95
140.3	5.35	24	18.44
62	6.5	37	11.89
71	18.72	54	14.9
72.2	5.52	44	1.37
89.1	8.8	20	18.35
314	7.16	32	18.02
73.5	11.77	37	13.82
64	6.88	31	4.92
70	10.96	37	21.45
65.2	7.6	21	5.17
71.1	5.7	36	13.46
69.5	4.72	33	10.04
121.4	28.93	39	22.99
196.1	6.45	28	13.4
98.2	9.43	27	17.17
24.5	11.17	28	4.69
115	5.79	29	10.01
97.9	18	97	8.4
69.2	18.35	45.2	22.79
118	6.01	32	6.46
57.8	10.02	32	7.99
48.2	7.44	24	21.04
99	7.15	19	6.45
63.2	5.22	59	19.83

58.1	7.95	28.1	3.26
60.1	5.62	11	4.75
68.7	8.5	52	9.84
112	6.34	21	22.46
147	5.96	30	8.05
65.9	5.93	46	8.58
47.8	11.75	20	14.28
85.4	8.91	52	13.01
155.8	7.99	20	20.15
151.7	7.15	64.9	10.18
107	5.85	18.2	23.99
58.6	10.93	47	11.06
73.5	5.05	27	14.88
163.8	10.01	36	10.55
67	5.02	20	16.33
56.6	4.8	29	3.94
96	4.8	32	5.65
82	5.02	25	12.63
120.4	6.55	23	26.04
136.5	5.92	60	15.51
75.9	9.62	37	8.63
56.3	6.48	18	4.49
52.5	7.52	13	8.03
61	12.49	69	7.92
112.5	5.28	21	7.32
60	4.57	61	0.88
110.6	10.99	42	6.7
78.3	6.76	37	6.12
52.5	7.32	27	22.74
54.1	12.28	52	6.71
79.1	8.17	37	2.13
187.8	27.15	69.1	14.82
54	5.1	28	8.85
53.7	9.9	67	7.44
46.8	6.18	36	2.32
46.4	13.77	36	4.16
66.9	9.64	26	19.87
86.6	6.03	13	5.36

47.2	7.42	33	14.82
328.3	9.28	72	22.42
55	30	30	9.03
82.5	8.22	33	6.06
20.2	10.23	60	3.85
73.2	6.5	60	4.88
48	8.5	16	2.04
93.2	3.67	33	19.87
102.8	7.94	43	7.9
52.2	20.47	43	3.44
40.3	10.6	36	8.21
88.1	6.93	58	6.66
24.1	26.15	17	1.17
84.7	6.32	51	11.9
87.8	5.88	42	7.9
72.3	21.21	8	3.44
39.7	4.75	50	10.35
55.9	6.75	32	2.41
64.3	9.9	41	8.72
90.2	18.01	33	2.69
118	7.53	37	13.08
169.2	8.01	24	18.57
185.2	6.44	33	13.01
86.8	18.05	29	4.24
51.2	12.58	66	8.99
42	7.15	45	9.15
69.4	16.82	12	8.95
66.1	10.12	52	6.68
71.3	18.54	32	3.98
88	5.93	14	9.63
117.1	6.23	39	2.7
44	5.52	24	8.71
207	12.27	21	21.77
183.9	5.65	26	13.26
94.9	7.84	34	16.72
84	2.98	12	4.11
36.9	8.8	29	21.54
292.3	5.41	52	20.51

67.3	7.97	44	6.13
197	10.3	14	11.86
82	5.03	31	22.91
69.7	8.74	37	9.81
114.9	9.92	37	7.21
173	5.18	20	14.53
66.9	7.58	36	6.63
133.8	12.5	56	14.09
65.8	12.19	49	3.02
122	12.83	43.8	13.69
82.2	4.72	28	8.52
103	6.87	21	21.33
58.5	23.95	53	16.69
81.2	6.02	10	9.92
71.5	13.63	48	2.04
99.3	9.73	26	4.12
35.6	5.64	49	9.03
61	5.66	33	3.91
71.5	9.57	28	3.19
73.7	5.1	44	9.87
113.7	6.35	36	8.9
73.7	7.51	40	22.54
44.8	16.91	28	6.45
189	6.6	22	20.87
71	5.42	54	19.18
56.9	13.14	45	16.29
84	6.9	24	8.38
72.7	20.02	40	1.82
66	23.54	38	12.25
58	11.65	32	3.53
61.1	6.73	48	13.79
67	5.28	41	7.2
145.6	5.18	67	20.92
81.3	7.31	2.4	9.58
78	14.91	26	5.04
69.3	6.95	38	7.73
106	5.56	25	4.17
79.7	9.55	48	8.59

50.1	8.82	27	15.91
53.6	7.75	61	7.18
66.7	9.52	39	4.27
67.8	6.42	35	7.57
180	5.76	41	16.37
48.3	11.99	35.3	5.5
64	5.19	32	3.04
99.4	6.88	17.2	5.27
53	4.87	36	6
197.3	4.78	18	8.47
85	4.92	19	8.83
60.6	6.5	10	4.09
51.8	11.31	27	6.62
60	8.8	11	3.46
96	9.02	46	9.04
86	14.24	59	3.36
49	7.24	40	17.66
46.1	20.96	47	18.18
60.3	4.99	55	7.85
60.4	14.57	29	1.17
78	3.67	43	7.82
69.3	6.82	36	16.83
71	3.84	34	6
67	8.38	45	8.14
47	12.09	35	11.48
84.5	4.18	48	13.53
55.8	6.72	34	16.26
121.9	22.62	37	9.39
66	6.87	30	8.87
49	6.46	53	20.13
59.8	9.99	48	2.7
66.2	6.91	50	11.11
50	7.97	33	11.65
32.6	7.41	71	5.81
54.8	5.78	41	14.01
67.6	6.83	36.2	10.46
57	4.18	47	7.07
88.4	13.9	24	2.59

49.6	7.64	27	6.58
81.1	7.66	33.6	19.66
72.7	9.65	52.7	6.87
89	3.42	26	8.58
63.1	5.08	32	15.88
65.3	7.94	39	5.5
41.5	5.67	11	6
64	5.5	26	3.01
113.6	8.61	18	11.71
62.1	8.97	30	5.78
48.4	14.16	33	4.68
61.2	6.71	48	6.84
71	8.4	40	3.53
60	5.67	24	13.48
51	17.72	59	8.81
58.1	9	60	6.29
89.5	4.22	22	16.71
59	6.05	17	15.3
66.6	12.5	18	2.5
106.1	9.79	44	23.43
66.9	6.84	35	7.8
50.5	6.55	33	2.59
71	4.54	47	4.36
45.3	7.47	37	8.05
57.1	6.63	21	5.15
57.1	9.41	55	4.01
64	8.53	48	4.54
60.7	7.74	63	6.16
143.9	45.57	41	9.49
73	11.54	48	9.31
53.7	14.93	56	6.79
66.3	5.88	48.8	6.65
53	8.93	52	4.87
68.9	33.12	56	7.01
74.2	15.33	65	15.93
71.1	4.85	23	5.17
90.3	7.77	58	8.11
61	7.97	40	3.22

85	5.42	52	13.24
53.3	9.52	44	9.67
181.5	4.64	25.8	18.44
28.8	11.94	14	4.31
65.7	11.08	30	11.19
56.9	10.15	33	8.76
145	4.95	42	29.39
82.1	7.19	40	10.9
60	7.64	19	3.28
67.9	8.3	44	4.54
103.6	6.19	33	8.47
49.2	41.75	48	4.84
69	12.38	37	11.01
113.3	8.01	44	13.33
66.6	7.83	57	7.58
56.2	8.51	23	4.01
66.7	5.16	16	15.96
119.6	5.32	15	9.71
69	22	31	12.81
63.8	10.82	19	9.08
70.4	8.4	25	17.01
79	8.13	62	5.77
75	11.38	52	8.64
82.5	23.4	38	15.86
78	9.01	33	9.13
484.8	3.91	17	17.2
67.2	9.15	53	6.18
109.8	9.27	32	5.11
51.1	10.58	52	8.93
74.3	5.94	17	1.37
30.1	7.9	37	8.33
50	4.88	35	9.21
76	5.64	55	11.44
17.2	7.63	35.3	2.59
66.3	6.46	54.66	15.68
49.7	5.09	21	6.99
206.9	6.78	44	22.37
80	7.32	35	3.81

60.5	14.87	15	5.11
77	4.6	34	9.19
63	4.68	10	14.84
62.9	6.14	29	5.9
44.6	9.32	25	11.93
23.7	16.83	68	8.68
61	16.36	35	6.16
82	3.98	18	1.63
69	5.34	45	8.46
73.6	7.72	40	3.02
164.3	7.62	27	8.36
67	5.1	42	16.5
72	4.28	14	14.49
49.1	6.08	43	6.64
94.3	7.4	61.3	5.25
146.6	11.07	50	12.05
59.4	10.41	52	23.75
59.1	6.37	31	2.94
76.2	7.66	41.1	9.78
60.7	9.51	67	15.06
37.5	15.96	70	13.43
52	5.16	48	9.05
79	6.91	25	12.05
157.8	7.16	42	19.19
56.2	8.52	37	5.12
52.4	10.55	75	4.54
75	10.27	12	1.85
94.5	11.61	19	21.91
95.3	10.91	36	6.32
75.4	7.53	14	4.05
104	8.18	19	8.26
127	5.23	20	8.84
61.3	17.32	22	3.93
74	7.59	51	9.99
126.7	5.67	71	31.9
147.5	9.12	52.7	6.6
86.3	6.45	18	1.01
84	8.44	7.1	1.76

70.5	18.29	41	9.81
67	5.93	18	15.57
61.3	12.59	36	11.97
154	4.68	45	19.94
52.8	7.79	41	6.06
45	4.62	36	8.56
174.8	7.48	32	15.54
65.8	10.36	27	6
67.8	10.38	33	6.3
47.5	5.27	36	5.56
76.8	9.28	44	4.24
170	24.07	49	9.22
87.2	8.4	48	15.65
46.8	21.68	43	4.05
64	4.71	25	14.96
136	29.08	40	11.23
41	8.94	46	11.36
138.1	5.82	60	22.88
69	5.52	15	11.27
92.2	10.48	125	3.38
64.9	9.19	34	0.76
28.6	14.72	30	7.76
57.5	8.25	28	10.1
95.6	7.91	34.1	8.14
73	8.43	25	3.93
59.3	14.01	29	4.51
176.3	10.83	75	14.16
154.3	19.68	46	11.73
124.9	11.17	56	8.59
73.7	7.4	31	4.7
47.5	9.48	34.3	6.81
57	5.99	11	14.67
58.1	7.89	55.5	4.29
26	11.53	31	11.34
56.8	8.89	46	8.94
74.7	6.28	41	6.75
123.1	6.71	5	5.31
69	5.81	33	4.2

62.2	10.56	49	8.25
49.4	5.3	25	3.16
57.1	7.54	50	6.14
54	6.02	40	11.07
75	7.46	129	11.26
60.2	8.16	18	27.38
47.1	6.83	42	8.74
61.7	8.1	60.8	20.85
72	16.14	11	3.61
87	9	59.4	12.19
53	5.24	33	11.15
149.3	4.76	62	14.27
52.8	6.5	68	9.42
77	6.21	44	9.16
29	6.41	36	4.51
64.3	5.68	19	2.9
61.1	7.86	24	10.75
77.9	5.3	16	7.19
39	4.09	29	10.69
52	7.17	51	7.61
83.5	8.21	21.3	10.1
93	5.18	33	5.21
160.8	10.41	14	16.71
106.6	7.16	37.8	11.89
89.9	8.68	22	9.39
66	4.89	34	18.8
49.3	7.39	87.1	1.76
75.4	7.46	71.3	10.34
96.7	8.26	78	10.27
39.8	21.81	22.9	5.15
61.1	26.03	92.7	13.7
64.2	8.47	42	5.98
49	19.46	48	11.66
128	6.35	47	5.78
72.8	15.59	41	7.9
140.7	9.24	34.1	6.22
78.8	8.61	24	5.14
46.1	5.31	40	3.1

46.6	10.55	49	5.97
90	6.78	48	7.43
53.7	17.22	53	2.85
63.8	10.27	13.4	16.59
60.1	18.97	44.2	2.59
93.7	9.12	67.7	16.44
125.8	7.17	26	8.99
109.6	2.73	65	8.15
95.5	14.04	44.6	8.87
50.9	17.67	19	5.2
75.5	7.21	60	6.75
60	16.96	43	10.63
82	3.57	92.7	9.71
76.8	12.94	19	14.91
86.2	9.79	42	5.22
81.5	8.95	61.2	4.54
102.2	8.71	28	19.32
124.5	8.81	49	9.83
89.6	8.23	21	7.16
102.8	7.8	47.3	4.87
47.5	8.48	82	9.88
62	19.38	84	8.79
62.9	10.24	74.4	9.58
54	10.26	32	5.61
65.7	6.5	37	5.77
99.5	5.08	71.4	18.61
75.9	15.52	48	11.37
64.2	9.61	46.74	5.32
105	9.47	49	10.7
90	7.68	15.3	14.72
81	5.71	33	8.3
79.6	4.88	27	10.01
53	16.67	56	0.76
84	6.38	48	1.27
58.3	5.8	36	0.11
94.5	8.31	33.6	13.8
86.9	12.33	65	6.43
56	6	10	1.17

72.6	5.64	51.8	12.51
41	12.61	42	5.31
81.4	9.1	57.1	18.47
70.9	8.94	36	9.18
68	11.17	48	6.59
68.9	3.91	42.1	11.74
61.8	10.09	48	0.44
86	4.93	34	23.42
66.5	6.97	56.3	3.42
88.1	15.39	57	3.49
51	12.7	53	8.12
69	4.81	35	6.41
54.3	6.72	37.8	22
95.9	8.1	13	3.46
124	6.92	43	21.66
65.4	12.75	43	15.33
73.6	8.4	31	4.24
84	7.83	52	1.93
46	13.06	51	12.32
53.3	17.73	91.3	1.95
46.7	21.29	76	25.51
44	4.16	54	5.24
105	8.74	41	7.66
88	10.05	17.1	10.72
39.7	12.77	100	12.54
85.8	6.91	26	3.76
52.5	17.2	59.5	2.22
46.2	11.03	46	6.06
44.3	11.68	52.7	4.04
47.6	7.02	55.2	22.15
45.3	8.91	60	5.71
95	6.29	33	15.48
42.8	6.4	20	2.02
77.2	6.13	21.7	19.25
48	5.83	59	8.93
36.8	8.26	75	9.81
61.2	11.34	32	11.46
57.4	5.35	36	5.28

100.8	6.44	44.3	3.02
49.6	13.2	74	4.11
69	13.43	34	1.47
86.7	7.54	36	8.68
67.8	8.67	54	4.24
58	17.12	36	2.13
102.7	9.65	24.2	10.71
56.2	9.68	36	4.56
75	4.9	30	9.66
56.8	25.09	64	16.74
58	13.26	15.7	11.54
117	4.9	44	3.55
67.9	24.37	33.1	5.32
38	8.37	107	12.7
85	3.07	15	11.84
35.6	16.46	53	3.72
107	4.32	15	5.02
100	9.68	32	8.78
65.9	5.9	34	3.34
69	10.67	34	2.59
51	13.32	42	6.08
106	4.81	39	3.21
63	6.42	148	8.58
86.8	5.59	49	4.09
51.2	5.81	48	5.39
52.3	6.97	21	24.1
104	10.19	38.1	9.7
46	4.39	35	7.32
44.4	5.53	27	4.44
60.3	6.76	37	3.53
67.4	6.54	32	10.3
52.8	13.66	48	16.4
92	4.66	16	5.14
82.5	7.26	62	7.37
73.3	6.64	60	10.1
69	4.97	34.4	10.29
69.2	7.1	68	5.98
102.5	21.26	22	5.68

107.1	13.5	69	13.83
55.5	5.13	24	21.58
65.5	5.41	19	7.46
55	11.31	62	5.64
62	14.21	31	16.45
63.5	13.8	24.1	2.5
81.6	14.04	62	3.93
64.8	11.25	42	21.81
67.7	7.22	50	1.69
80.6	13.05	65	13.96
67	9.08	43	2.41
39.9	6.94	22	6.94
89.7	40.59	30	7.47
60	23.83	36	2.68
51.2	11.59	52	6.23
68.5	5.8	13	16.18
59	6.1	34	6.49
63.7	9.47	49	3.81
128.5	7.72	33	5.95
57	7.58	20	7.98
88	7.42	64	5.03
50	4.3	30	9.24
53	5.17	35	4.08
54.4	5.98	13	6.2
55.8	5.33	71	21.53
51.9	6.48	22	1.57
51.7	6.53	39	0
52	6.93	53	8.93
65.8	9.89	66	6.07
61.3	15.77	23	2.48
53.1	8.48	19	7.98
64	7.89	24	6.31
101.3	9.92	24	13.81
76	9.53	31	14.81
45	4.74	20	15.57
69	4.26	48	0
64	4.7	41	19.01
54	4.83	50	9.52

86.9	9.21	44	7.71
47.5	10.64	27	10.06
52.1	5.63	63	1.79
185.9	13.29	63	25.94
98	10.27	55	8.87
112.6	3.54	43	18.52
57.3	14.17	20	6.08
63.9	21.32	30	6.23
99.3	11.61	54	8.35
51.2	7.8	37	4.76
76.1	7.66	26	3.02
37.6	7.75	44	2.68
47.8	17.29	24	11.48
54	8.54	38	2.68
72.1	7.73	18	3.02
68.7	10.35	25	15.15
61.6	7.65	34	5.26
49.8	8.74	40	9.41
60.5	7.28	73	7.22
56.5	7.69	44	7.51
58.1	8.67	26	2.23
72.7	7.65	99	5.55
38.7	5.62	10.3	10.11
80	4.74	28	0.86
16.6	7.71	71	5.61
50.2	5.28	33.5	4.32
103	10.22	9	5.07
179.3	9.33	74	23.22
81.1	10.59	56	18.72
78	15.23	61	0.97
176	3.02	52	23.86
425.7	6.54	53	34.02
84.6	6.64	48	20.58
133	6.43	13	28.74
79.4	7.1	20	19.27
119.4	16.4	35.1	20.06
55.1	13.97	35	7.76
61.6	5.27	68	30.9

215	3.09	36	32.82
98.3	7.88	40	9.93
273.5	6.66	46	33.31
116.9	5.69	106	29.51
137.5	7.27	45	24.63
59.9	13.79	69	20.4
83.7	7.91	26	5.39
272.8	8.85	47	25.79
109	19.11	20	19.79
202.8	7.13	66.2	22.54
109.2	9.42	19	26.67
316.3	7.36	157	28.85
542.9	6.68	22	38.35
275	15.14	49	34.51
61.2	6.58	45	19.87
126.9	4.71	26	25.29
60	5.89	36	17.58
44.1	11.88	35	19.5
56	6.66	41	19.82
172.2	6.08	34	12.32
59.8	8.41	49	20.05
196.5	7.61	42	18.66
58	3.4	20	23.83
60.1	8.74	24	17.65
99	7.35	114	8.11
87	6.24	38	19.92
43.8	11.42	30.5	26.4
116	18.3	99	20.08
38.9	7.92	36	9.22
80.7	6.33	44	4.41
82	5.94	20	10.34
40	6.55	29	14.57
175	4.6	122	24.85
96	5.04	30	18.56
68.2	13.76	39	13.72
136.4	9.67	2.8	23.19
128	6.53	108.8	11.94
74.3	8.67	15	5.67

56.8	6.46	44	19.99
200.6	10.23	41.8	22.96
53	11.55	32	21.02
207.8	7.04	36	31.47
52	6.49	34	19.76
115.7	29.31	58	12.96
115	4.52	34.7	18.11
168	7.81	15	27.38
41.8	8.44	19	13.26
55	4.07	16	19.7
84.4	8.45	20.6	5.59
48	5.47	20	1.85
59	6	23	0.33
68.3	8.87	19	10.94
218.3	3.59	131	20.59
180.8	5.44	25	23.31
80	6.77	33	21.44
98.7	27.05	48	13.78
265.2	8.13	72	15.88
37.6	22.23	34	17.04
52	27.1	58	18.1
60	4.04	43	7.25
55	4.08	28	7.32
63	7.57	15	9.21
62	5.2	40	2.16
72.1	7.77	68	16.83
98.3	7.8	57	6.03
139.4	6.65	316	19.86
50.5	9.96	46	7.77
61.1	8.59	10	18.31
55.6	6.67	40	8.9
66	6.85	38	12.76
56.3	13.67	44	5.97
165.4	6.63	57	9.44
52.9	8.83	25	6.86
60	5.29	20	1.49
103	5.55	16	10.17
99.6	6.51	7	9.56

89	4.25	16	17.84
58	6.92	22	10.05
50.6	9.92	53	13.29
56.8	7.9	22	8.77
64.7	6.66	43	22
68.1	6.32	36	17.24
89.9	11.33	36	7.58
159.5	10.89	44	22.02
83.9	6	67	4.36
56	13.67	48	2.13
88.6	8.19	22	4.27
89.3	16.91	52	5.6
58.1	6.62	36	9.43
35.2	10.87	30	1.95
685.7	5.89	32	25.95
206.5	8.17	24	12.94
59	5.7	29	6.63
119	8.89	29	6.71
52	4.94	47	3.77
116	6.88	37	8.96
119.7	6.65	6	11.86
175	7.51	34	29.98
79.5	8.13	25	11.22
111	7.15	38.7	13.36
70.6	9.78	77	3.31
130.5	6.81	44	6.33
129.8	9.98	31.7	6.82
238.9	8.67	960	15.35
61.8	5.3	39	13.8
65.5	5.31	20.2	7.46
162.7	35.41	65	12.08
40	8.22	13.8	4.84
102	5.33	23	13.81
82.8	37	37	6.76
68.1	6.69	22.9	5.82
63.7	17.24	34.7	6.73
52	5.89	44	27.77
147.7	15.62	43	12.81

61.8	8.38	23	5.07
78.4	5.13	8	5.12
113	10.48	21	4.86
326	11.82	7	22.34
198.1	4.89	42	21.63
74.6	7	20	17.14
45.6	11.36	37	15.72
35.1	8.51	51	12.62
57.5	6.49	39	15.29
42	6.84	26	14.85
65.3	12.42	55	30.93
253.5	4.44	20	33.7
124	4.64	28	17.12
89.5	12.1	30	11.68
65.4	11.71	19	5.59
56.5	7.75	41	18.06
71.5	13.12	58	7.34
56.2	5.57	34	10.66
42	7.1	11	6.2
40.9	21.04	66	12.72
43.4	6.34	36	5
58	5.17	21	9.5
45.4	20.49	22	11.38
81	5.61	25	13.58
54.5	7.59	27	3.44
83	7.72	32	15.46
53.3	5.64	33	4.98
43.9	15.37	61	6.71
74.5	9.49	13	4.76
89.6	5.53	39	10.3
142.6	2.88	32	22.76
87.4	8.68	19	12.47
154.6	28.49	38	21.95
45.1	12.59	17	11.94
60.9	4.84	13	18.06
238.2	11.47	54	14.04
80.6	5.22	37	15.58
30	5.66	39	9.22

133.4	7.16	19	13.15
94.9	7.95	27	10.23
67.1	5.76	33	3.53
66.2	6.78	39.4	13.49
69	4.77	29	21.25
116.4	5.37	34	16.54
52.5	8.23	26	19.11
60.7	12.6	50	3.88
329	4.99	28	23.95
199.9	9.99	12	31.21
72.1	6.93	48	6.76
83	12.45	12	12.67
47.4	19.6	32	7.41
62	5.61	12	12.21
82	4.89	10	22.15
136	5.53	26	11.76
53	8.05	20	3.82
184.5	20.35	20	23.13
74.9	7.44	18	13.02
63.9	1.7	61	4.86
246.4	17.05	25	14.04
136.6	8.31	20	7.2
72	9.11	24	3.34
81	5.77	35	20.52
88.7	14.03	23	3.06
47.7	14.81	63	7.42
51.3	10.1	47	11.05
33.7	6.2	25	15.12
79	11.04	24.5	1.57
129.9	10.72	20	8.59
51	9.32	27	17.4
54.3	5.93	20	6.26
75.4	5.66	11	18.95
52	6.66	38	8.64
74.3	5.17	46	4.1
63.5	8.14	0.6	14.83
96	4.14	39	16.83
52	5.01	82	27.03

90.4	7.03	33	5.52
203	12.74	26	10.41
106	15.42	71.9	3.83
55.6	8.31	50	20.15
102.1	7.13	32	15.42
50.6	5.96	99	9.63
62.6	7.89	58.9	6.34
33.7	9.82	21	9.76
119	9.95	30	6.31
40.4	5.22	17	14
232.1	7.66	22.8	19.94
111	4.48	34	17.27
114.4	7.37	48	11.43
51	6.98	58	10.16
68.9	5.56	22.8	13.34
98	7.63	65	4.81
66.2	5.96	58	15.47
73.5	11.77	37	13.82
196.1	6.45	28	13.4
24.5	11.17	28	4.69
54	7.09	40	18.04
115	5.79	29	10.01
118	6.01	32	6.46
57.8	10.02	32	7.99
52.6	7.31	51	5.11
49	4.17	36	7.52
147	5.96	30	8.05
71	5.48	19	2.62
151.7	7.15	64.9	10.18
67	5.02	20	16.33
93	7.83	41	9.2
101.4	9.33	25	12.59
72.4	15.89	46	7.99
97.3	19.69	23	5.86
62	4.91	52	7.07
110.6	10.99	42	6.7
54.1	12.28	52	6.71
55.4	9.18	20.6	8.8

51.7	8.62	25	8.99
46.8	6.18	36	2.32
86.6	6.03	13	5.36
82.5	8.22	33	6.06
48	8.5	16	2.04
51.9	7.54	57	10.87
43.8	14.87	42	6.48
90.2	18.01	33	2.69
42	7.15	45	9.15
154.6	6.68	21	7.95
44	5.52	24	8.71
70.3	7.98	39	6.39
292.3	5.41	52	20.51
92.9	5.25	30	3.65
70	11.74	24	9.11
230	6.15	35	11.97
29.7	6.87	55	5.54
82.2	4.72	28	8.52
99.7	8.13	30	10.45
71.5	13.63	48	2.04
66.6	6.49	13	9.66
61	5.66	33	3.91
89.4	9.22	22	6.34
66	5.38	25	11.95
71.8	11.81	22	4.16
61.1	6.73	48	13.79
67	5.28	41	7.2
70	5.02	30	6.97
79.7	7.09	21	4.22
66.1	7.07	26	10.13
66.7	9.52	39	4.27
67.8	6.42	35	7.57
70.9	9.02	25	1.66
48.7	9.88	92	7.06
51.8	11.31	27	6.62
78	3.67	43	7.82
90.8	10.57	65	3.26
47	12.09	35	11.48

121.9	22.62	37	9.39
63.5	13.13	37	5.8
113	7.71	64	7.68
66	6.87	30	8.87
64	4.35	28	5.13
64	4.27	34	4.23
86.2	9.73	22	4.96
49.4	9.34	24	4.6
54.2	4.92	27	13.88
68.6	8.76	55	6.76
84	4.35	32	8.86
35.3	5.85	23	2.13
61	7.97	40	3.22
82.1	7.19	40	10.9
60	7.64	19	3.28
67.9	8.3	44	4.54
63	7.53	42.6	8.04
56.2	8.51	23	4.01
119.6	5.32	15	9.71
141.1	5.71	54.3	5.76
109.8	9.27	32	5.11
100.7	9.86	26	9.84
63.7	10.68	33	20.56
91.5	8.07	23	5.28
32	20.23	18	2.76
48.2	11.18	63.4	2.94
36.8	9.1	29	7.83
68	5.08	34	1.76
164.3	7.62	27	8.36
52	7.57	39	5.21
53.8	9.09	77	2.41
67	6.88	22	7.81
68.5	6.27	38	8.62
58	8.69	46	6.03
92.7	11.55	36	8.26
72.9	7.99	40	16.01
45	6.63	50	6.83
65.8	10.36	27	6

71.7	5.57	48.5	9.08
139.3	9.05	19	5.23
38	5.09	55	5.37
69	5.52	15	11.27
40	6.03	47	4.14
64.9	9.19	34	0.76
95.6	7.91	34.1	8.14
176.3	10.83	75	14.16
74.6	8.2	30	5.37
56.8	8.89	46	8.94
68	6.31	30	2.56
49.4	5.3	25	3.16
81	5.03	27	1.27
46	8.99	11.8	4.11
64.3	5.68	19	2.9
77.9	5.3	16	7.19
39	4.09	29	10.69
72.5	10.77	15	11.59
28.2	6.94	35	8.33
49.3	7.39	87.1	1.76
140.7	9.24	34.1	6.22
78.9	12.03	36.2	9.6
93.7	9.12	67.7	16.44
51.7	28.82	54.2	8.04
50.9	17.67	19	5.2
57.5	16.09	25.9	4.54
65.7	6.5	37	5.77
85.3	4.6	21	14.71
53	16.67	56	0.76
158	6.76	31.5	9.44
68	5.14	19	4.73
43.8	8.06	43	4.23
47.5	6.57	28	7.64
84.4	6.72	78.3	2.59
60	4.76	25	2.85
79	4.32	25	12.25
84	7.83	52	1.93
84	6.28	43	3.95

91	7.33	26	3.93
62	5.37	36	4.54
49.2	9.76	24	3.51
67.8	8.67	54	4.24
51.8	5.16	71	8.54
39.3	13.54	16	3.11
62.9	6.78	43	2.12
60	23.83	36	2.68
66.1	12.37	26	2.41
57	7.58	20	7.98
74.4	6.48	37	7.61
76.2	16.15	34	3.77
74.5	8.82	47	2.98
52	5.91	32	4.22
61.3	15.77	23	2.48
20.2	8.31	24	8.08
58	5.77	17	5.77
66	4.77	35	12.23
37.6	7.75	44	2.68
64	3.66	42	5.38
176	3.02	52	23.86
141.8	7.85	33	29.81
523.5	9.49	17	30.79
202.8	7.13	66.2	22.54
316	8.79	28	34.26
79.9	14.4	28	15.1
87.4	5.44	35	16.91
44	6	28	27.9
172.2	6.08	34	12.32
87	6.24	38	19.92
43.8	11.42	30.5	26.4
96	5.04	30	18.56
150.6	5.73	26.1	22.05
77.3	9.84	15	22.31
90.3	4.31	26	5.95
115.7	29.31	58	12.96
48	5.47	20	1.85
